# Supplementary material for: Structural and functional investigations of syn-copalyl diphosphate synthase from Oryza sativa
Source: Commun Chem. 2023 Nov 6;6:240. doi: 10.1038/s42004-023-01042-w (PMC10628199; doi:10.1038/s42004-023-01042-w)
Supplement: Supplementary file 2 — Supplementary Information [file 42004_2023_1042_MOESM2_ESM.pdf]

Supplemental Information for

Structural and functional investigations of *syn*-copalyl diphosphate synthase from *Oryza sativa*

Xiaoli Ma[1,5,*], Haifeng Xu[1,2,5], Yuru Tong[3], Yunfeng Luo[4], Qinghua Dong[1], Tao Jiang[1,2,*]

[1]National Laboratory of Biomacromolecules, Institute of Biophysics, Chinese Academy of Sciences, Beijing, China.

[2]University of Chinese Academy of Sciences, Beijing, China.

[3]School of Pharmaceutical Sciences, Capital Medical University, Beijing, China.

[4]School of Traditional Chinese Medicine, Capital Medical University, Beijing, China.

[5]These authors contributed equally.

[*]Corresponding Author: Xiaoli Ma (maxiaoli@ibp.ac.cn), Tao Jiang (tjiang@ibp.ac.cn).

Contents

Tables **4**

Supplementary Table 1. Crystal structure data collection and refinement statistics. 4
Supplementary Table 2. Cryo-EM data collection, refinement and validation. 5
Supplementary Table 3. No. of particles that contribute to the cryo-EM map 6
Supplementary Table 4. The distances between the substrate and relative amino acids (Å) 6
Supplementary Table 5. List of Constructs for Relative Enzyme Activity Assays. 6
Supplementary Table 6. List of Mutants for New Product Identification. 7

Supplementary Figures **8**

Supplementary Figure 1. The protein properties of OsCyc1 and OsCyc1$^{69-767}$..................8
Supplementary Figure 2. Extracted ion chromatograms (m/z=275) obtained from GC-MS analysis of the products formed by OsCyc1, OsCyc1$^{69-767}$ and OsCyc1 at different magnesium ion concentrations...9
Supplementary Figure 3. Supplementary data for cryo-EM structures of OsCyc1 and OsCyc1^{D367A}. 10
Supplementary Figure 4. Structure alignment and relative enzyme activity of OsCyc1 .. 11
Supplementary Figure 5. Density maps depict the density details of relative amino acids in Figure 2b 12
Supplementary Figure 6. Density maps depict the density details of relative amino acids in Figure 2c (a, b, c) and 2d (d) 13
Supplementary Figure 7. Density maps depict the density details of relative amino acids in Figure 2d (a), 2e (b, c) and 2g (b, d) 14
Supplementary Figure 8. Density maps depict the density details of relative amino acids in Figure 2h (a, b) and 2i (c, d) 15
Supplementary Figure 9. Density maps depict the density details of relative amino acids in Figure 2j 16
Supplementary Figure 10. Extracted ion chromatograms (m/z=275) obtained from GC-MS analysis of the products formed by OsCyc1 and various mutants related to interface of OsCyc1 oligomers...17
Supplementary Figure 11. Extracted ion chromatograms (m/z=275) obtained from GC-MS analysis of the products formed by OsCyc1 and various mutants related to surface-exposed positive residues and active pocket residues...19
Supplementary Figure 12. The magnified views of Figure 4..21
Supplementary Figure 13. Electro potential map surface of OsCyc1^{D367A}, AtCPS (PDB: 3PYA) and SmMDS (PDB: 4Y47) 22
Supplementary Figure 14. Density maps depict the density details of relative amino acids in active site 23
Supplementary Figure 15: Sequence alignment of OsCyc1 and its homologs. 24
Supplementary Figure 16. Sequence alignment of class II diterpene synthases that produce 8-hydroxycopalyl diphosphate...26
Supplementary Figure 17: Extracted ion chromatograms (m/z=275) obtained from GC-MS analysis of the products formed by OsCyc1 and various mutants related to active pocket

residues 27
Supplementary Figure 18: Extracted ion chromatograms (m/z=275) obtained from GC-MS analysis of the products formed by various mutants..29
Supplementary Figure 19: Extracted ion chromatograms (m/z=272) obtained from GC-MS analysis of the products formed by related enzymes 30
Supplementary Figure 20: Molecular docking and cyclization mechanism of GGPP to form various intermediates 31
Supplementary Figure 21: The phylogenetic tree of *syn*-copalyl diphosphate synthases for sequence analysis..32
Supplementary Figure 22: The phylogenetic tree of *ent*-copalyl diphosphate synthases for sequence analysis..33
Supplementary Figure 23: The phylogenetic tree of (+)-copalyl diphosphate synthases for sequence analysis 34
Supplementary References **34**

Tables

Supplementary Table 1: Crystal structure data collection and refinement statistics.

**Data collection**	**8KBW**
Space group	$P2_12_12_1$
Wavelength	0.97853
Resolution range	87.17-3.494 (3.619-3.494)
Unit cell	129.70 174.34 296.55 90 90 90
Total reflections	1154850 (118972)
Unique reflections	85822 (8453)
Multiplicity	13.5 (14.0)
Completeness (%)	96.51 (91.20)
Mean I/sigma(I)	10.14 (1.50)
Wilson B-factor	86.54
R-pim	0.1111 (0.7547)
$CC_{1/2}$	0.995 (0.511)
**Refinement**	
Reflections used in refinement	82868 (7722)
Reflections used for R-free	1947 (187)
R-work	0.2014 (0.3130)
R-free	0.2630 (0.3723)
Number of non-hydrogen atoms	
macromolecules	32467
Protein residues	4058
RMS(bonds)	0.011
RMS(angles)	1.40
Ramachandran favored (%)	91.35
Ramachandran allowed (%)	7.26
Ramachandran outliers (%)	1.39
Rotamer outliers (%)	0.09
Clashscore	19.13
Average B-factor	
macromolecules	81.33

Statistics for the highest-resolution shell are shown in parentheses.

Supplementary Table 2. Cryo-EM data collection, refinement and validation statistics

	Dimer	Tetramer	Hexamer	OsCyc1^{D367A}
	EMD-35207<br>PDB: 8I6U	EMD-35202<br>PDB: 8I6P	EMD-35206<br>PDB: 8I6T	EMD-35440<br>PDB: 8IH5
**Data collection and processing**				
Magnification	22500X	22500X	22500X	22500X
Voltage (kV)	300	300	300	300
Camera	Gatan K3	Gatan K3	Gatan K3	Gatan K3
Electron exposure (e/Å^2)	60	60	60	60
Defocus range (μm)	-1.5~-2.3	-1.5~-2.3	-1.5~-2.3	-1.5~-2.3
Pixel size (Å)	1.07	1.07	1.07	1.07
Symmetry imposed	C1	C2	C1	C1
Micrographs collected (No.)	3287	3287	3287	3939
Particles used (No.)	27984	149837	97919	735747
Resolution (global Å)	7.88	3.52	3.65	4.0
FSC 0.5 (unmasked/masked)	9.23/8.14	4.45/4.04	6.35/4.31	4.20/3.99
FSC 0.143 (unmasked/masked)	7.08/6.18	3.67/3.36	3.97/3.49	3.79/3.72
Map sharpening B factor (Å^2)	-781	-60	-44	-237
**Model composition**				
Protein (aa)	1376	2752	4122	4119
Ligands (No.)	0	0	0	GRG: 6
**Model refinement**				
B factors (Å^2)				
Protein residues	-	178.82	196.60	5.84
Ligands	-		-	19.56
R.m.s. deviations from ideal values				
Bond lengths (Å)	0.004	0.005	0.005	0.003
Bond angles (°)	0.917	0.749	0.978	0.610
**validation**				
Molprobity score	3.23	3.12	1.80	1.67
Clashscore	21.62	21.34	6.85	7.33
Poor rotamers (%)	16.42	15.58	0.59	0.34
Ramachandran plot				
Favored (%)	92.35	94.28	93.65	96.05
Allowed (%)	7.65	5.72	6.35	3.95
Disallowed (%)	0	0	0.02	0

Model statistics generated using Phenix comprehensive validation (cryo-EM)[1].

Supplementary Table 3. No. of particles that contribute to the cryo-EM map

	dimer	tetramer	hexamer
OsCyc1	27984	149837	97919
OsCyc1^{D367A}	-	-	735747

Supplementary Table 4. The distances between the substrate and relative amino acids (Å)

	H251	C310	I311
OsCyc1	3.7	6.9	3.7
AtCPS	2.6	4.6	4.2
SmMDS	3.5	6.3	3.6

Supplementary Table 5. List of Constructs for Relative Enzyme Activity Assays

Constructs	Position of Relevant Amino Acids
OsCyc1	
OsCyc1^{D367A}	
Q639A	Interface of OsCyc1 oligomers
H661A/T664A	Interface of OsCyc1 oligomers
Q678A/S681A	Interface of OsCyc1 oligomers
S674A/E677A	Interface of OsCyc1 oligomers
R114A	Interface of OsCyc1 oligomers
L115A/D116A	Interface of OsCyc1 oligomers
Q291A	Interface of OsCyc1 oligomers
D298A/K302A	Interface of OsCyc1 oligomers
H334A	Surface-exposed positive residues
R535A	Surface-exposed positive residues
R733A	Surface-exposed positive residues
V196I	Active pocket amino acids
V196A	Active pocket amino acids
H275L	Active pocket amino acids
I311V	Active pocket amino acids
I311A	Active pocket amino acids
L314F	Active pocket amino acids
L314A	Active pocket amino acids
L314V	Active pocket amino acids
Y317F	Active pocket amino acids
Y317A	Active pocket amino acids
H357W	Active pocket amino acids
H357A	Active pocket amino acids
L400F	Active pocket amino acids
L400A	Active pocket amino acids

Supplementary Table 6. List of Mutants for New Product Identification

	Mutants	Results
1	H275L/H357W	No detectable products
2	Y317F/H357W	No detectable products
3	H275L/Y317F	No detectable products
4	H275L/Y317F/H357W	*syn*-CPP
5	H275L/H357W/L400F	*syn*-CPP.
6	H275L/Y317F/H357W/L400F	*syn*-CPP.
7	H275L/I311V/Y317F	No detectable products
8	H275L/C310D/I311V/Y317F	No detectable products
9	L314V/Y317F/H357W	Trace amounts of *syn*-CPP
10	I311V/Y317F/H357W	No detectable products
11	I311V/L314V/Y317F/H357W	No detectable products
12	L314V/Y317F/H357W/L400F	No detectable products
13	I311V/Y317F/H357W/L400F	No detectable products
14	I311V/L314V/Y317F/H357W/L400F	No detectable products
15	H275L/I311V/L314V/Y317F/H357W (OsCyc1^{5Mu})	*syn*-CPP and another CPP
16	H275L/I311V/L314V/Y317F/H357W/L400F	*syn*-CPP and another CPP
17	Y317F/H357W/L400F	Protein aggregation

Supplementary Figures

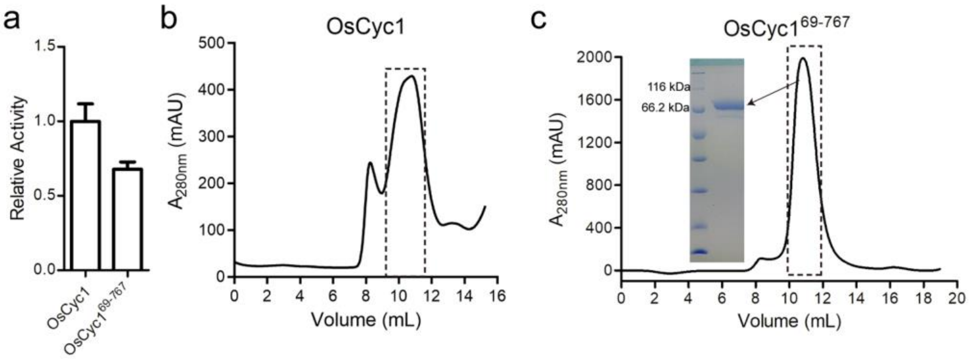

Supplementary Figure 1. The protein properties of OsCyc1 and OsCyc1$^{69\text{-}767}$. (a) Relative enzyme activity of OsCyc1$^{69\text{-}767}$ compared to that of WT OsCyc1. The error bar represents the mean with SEM. (b, c) Size exclusion chromatography of OsCyc1 and OsCyc1$^{69\text{-}767}$. Protein samples were loaded on a Superdex 200 HR 10/300 column for gel filtration assays.

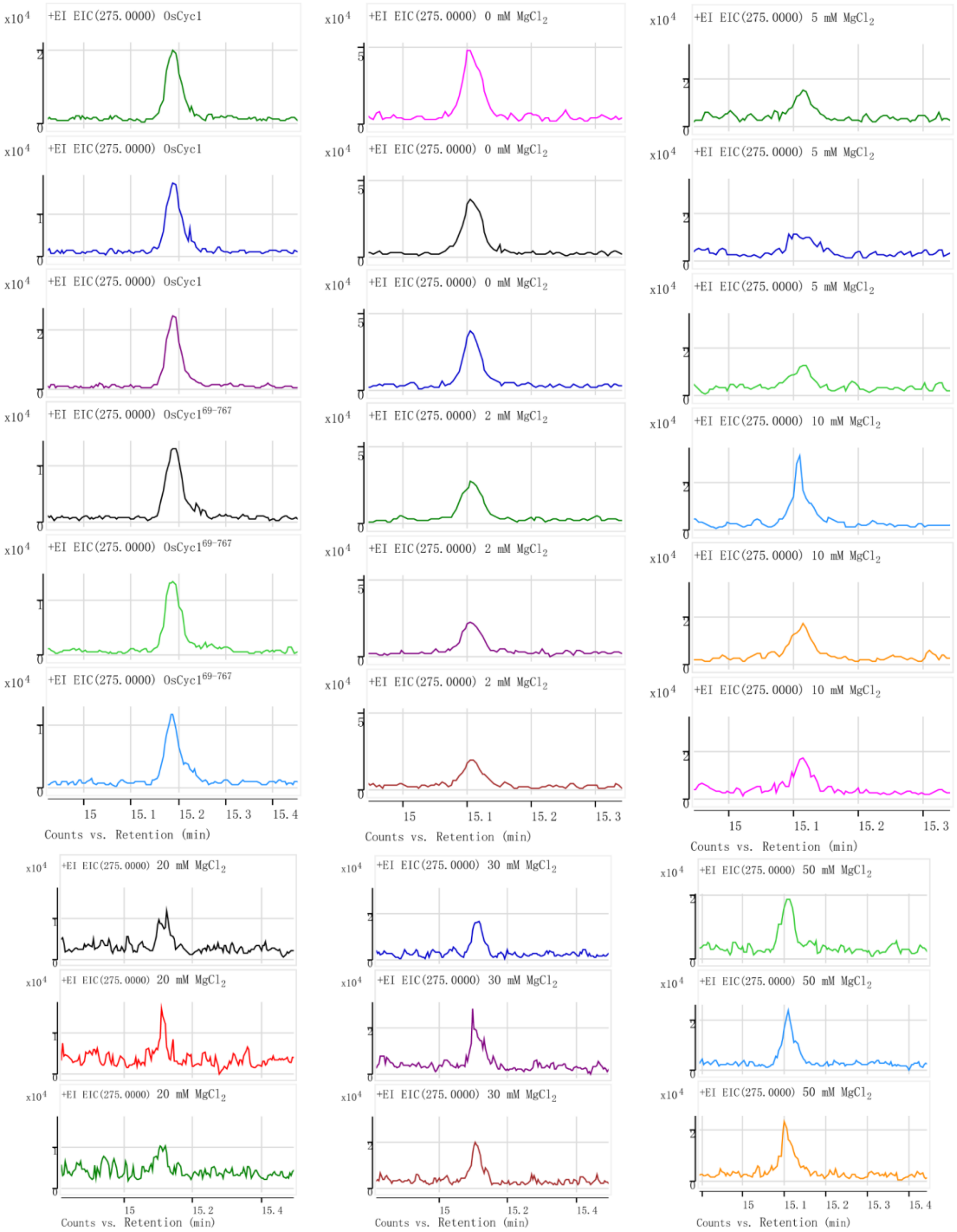

Supplementary Figure 2. Extracted ion chromatograms (m/z=275) obtained from GC-MS analysis of the products formed by OsCyc1, OsCyc1$^{69\text{-}767}$ and OsCyc1 at different magnesium ion concentrations. In each batch of GC-MS experiments, the product catalyzed by OsCyc1 was utilized as the standard reference. The experiments were conducted with three replicates each.

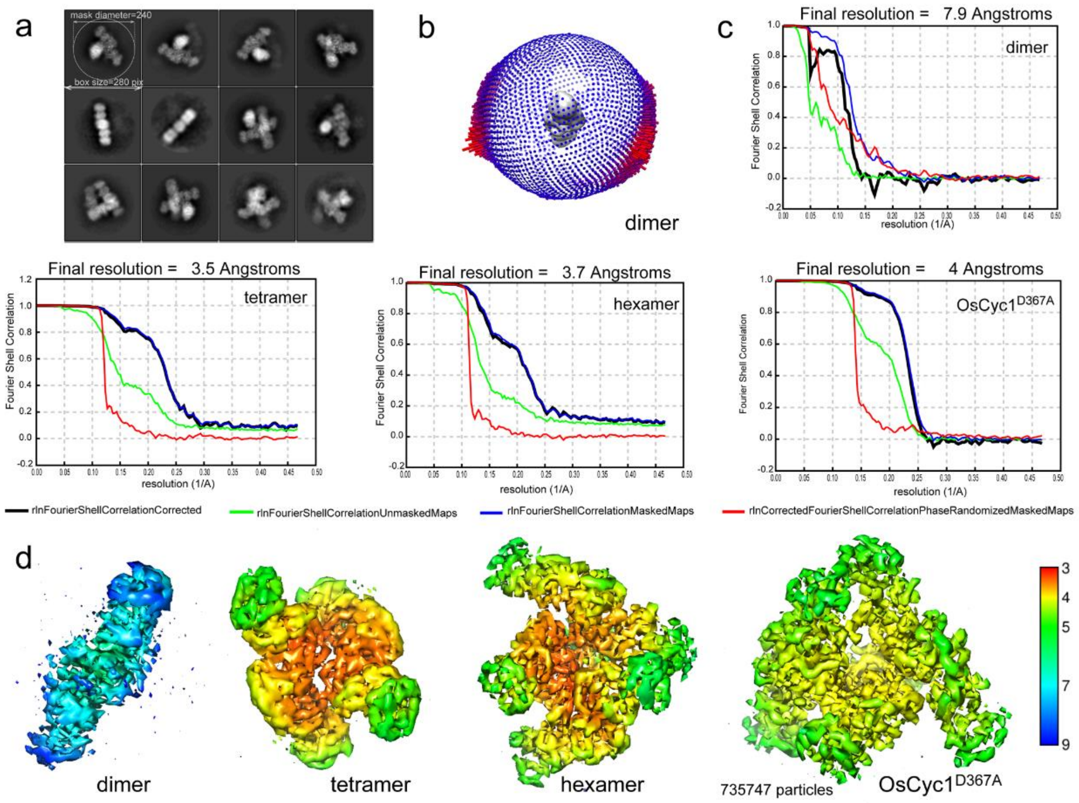

Supplementary Figure 3. Supplementary data for cryo-EM structures of OsCyc1 and OsCyc1^{D367A}. (a) Representative 2D class averages of the cryo-EM particles from OsCyc1. The box size is 280 pixels, and the mask diameter is 240 angstroms. (b) Angular distribution of particle orientations that go into the final class for dimer reconstruction. (c, d) Fourier shell correlation graphs and cryo-EM density maps used in model building. The maps are colored by local resolution.

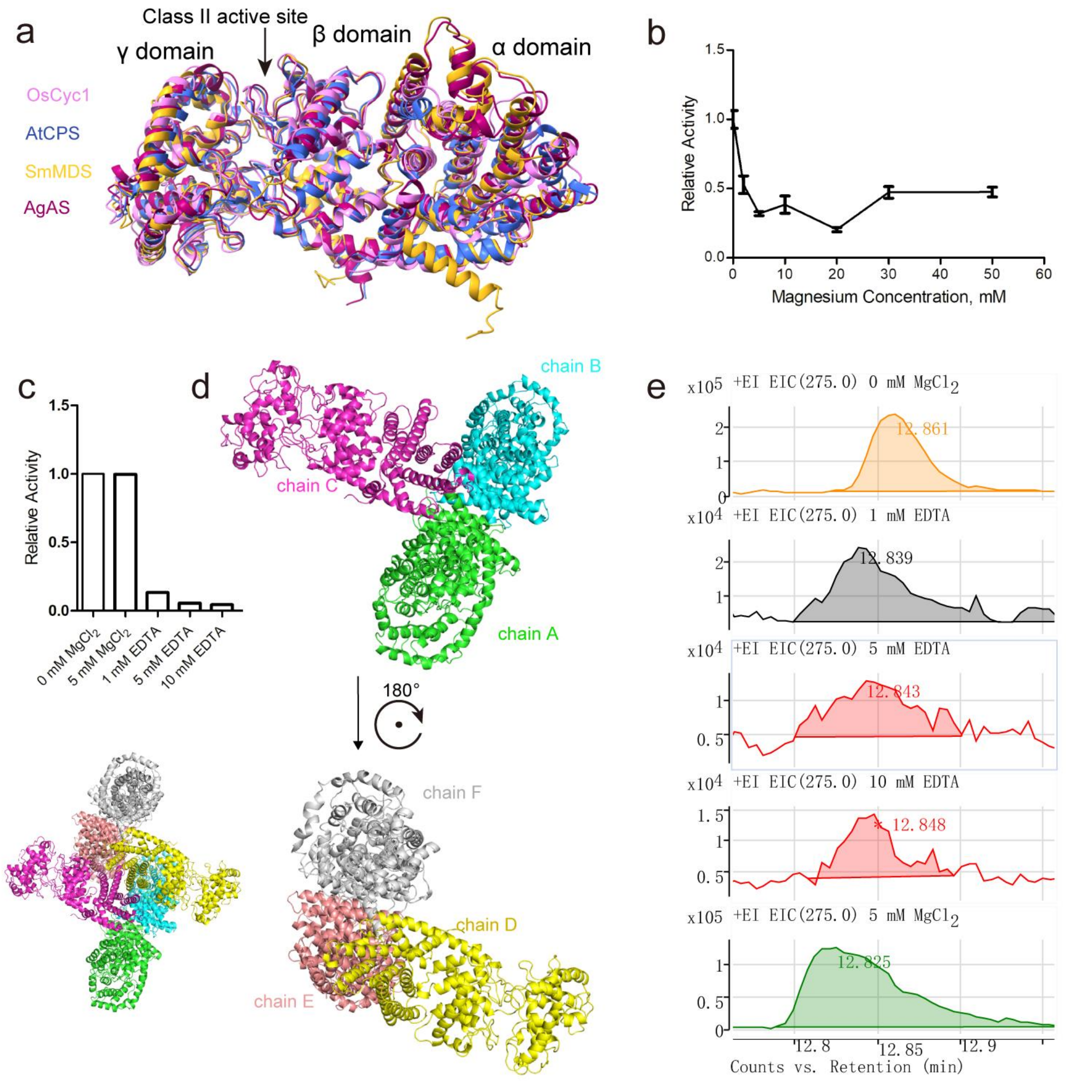

Supplementary Figure 4. Structure alignment and relative enzyme activity of OsCyc1. (a) Structure alignment of OsCyc1 (violet), AtCPS (PDB: 3PYA, royal blue), SmMDS (PDB: 4Y47, goldenrod) and AgAS (PDB: 3S9V, red-violet). (b) Relative enzyme activity of OsCyc1 at different magnesium ion concentrations. The experiments were conducted with three replicates each. The error bar represents the mean with SEM. (c) Relative enzyme activity of OsCyc1 at different ethylenediaminetetraacetic acid tetrasodium salt (EDTA) concentrations. (d) Structure relationship between chain A/B/C and chain D/E/F in the hexamer structure. (e) Extracted ion chromatograms (m/z=275) obtained from GC-MS analysis of the products formed by OsCyc1 at different EDTA concentrations. The experiments were conducted with one replicate each.

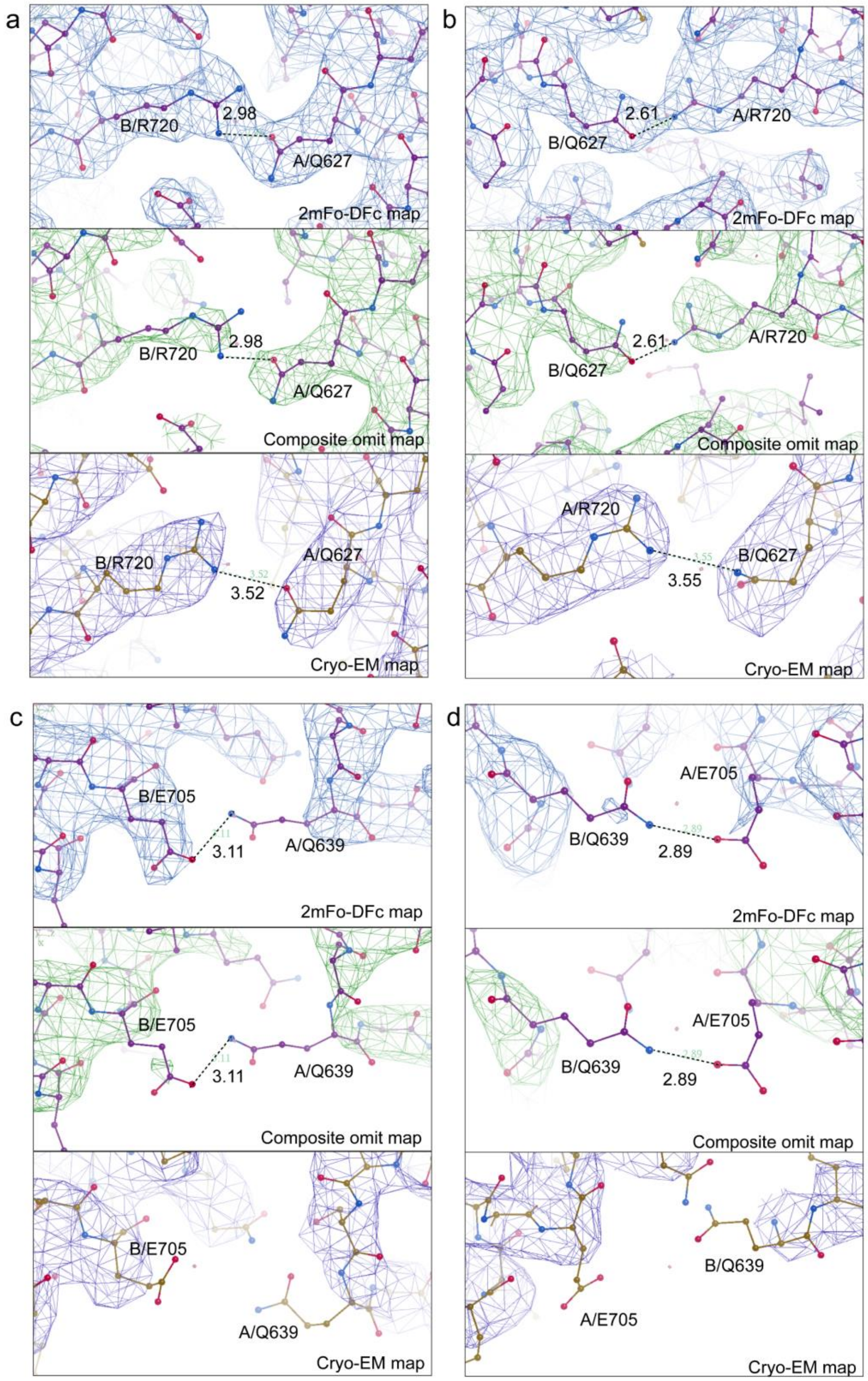

Supplementary Figure 5. Density maps depict the density details of relative amino acids in Figure 2b. The 2mFo-DFc map (contoured at 1.01 σ) and composite omit map (contoured at 2.0 σ) originate from the crystal structure. The Cryo-EM map (contoured at 5.00 σ) is derived from OsCyc1^{D367A}. The crystal structure of OsCyc1 is represented in purple, while the structure of OsCyc1^{D367A} is depicted in yellow. The images were captured using COOT.

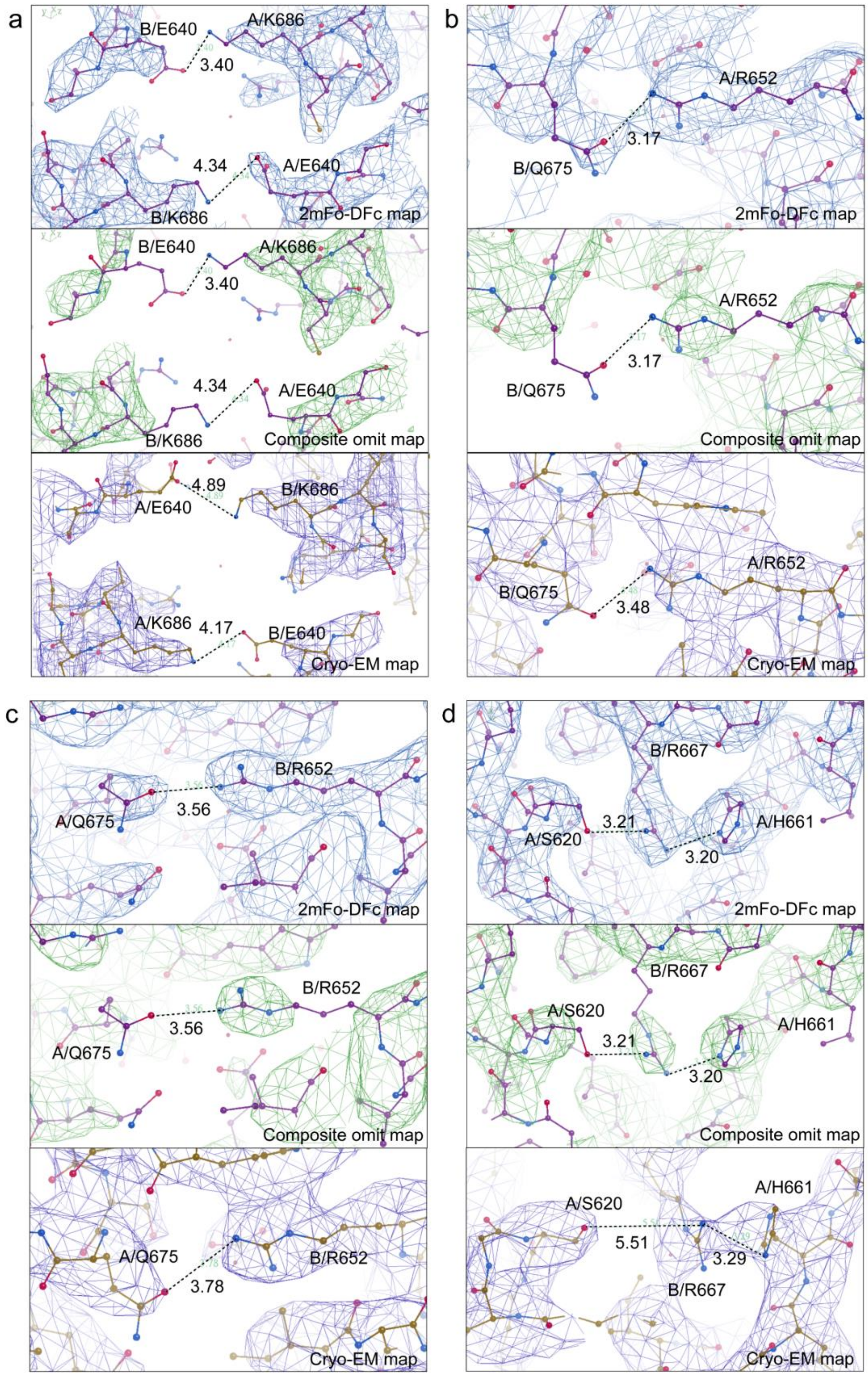

Supplementary Figure 6. Density maps depict the density details of relative amino acids in Figure 2c (a, b, c) and 2d (d). The 2mFo-DFc map (contoured at 1.01 σ) and composite omit map (contoured at 2.0 σ) originate from the crystal structure. The Cryo-EM map (contoured at 5.00 σ) is derived from OsCyc1^{D367A}. The images were captured using COOT.

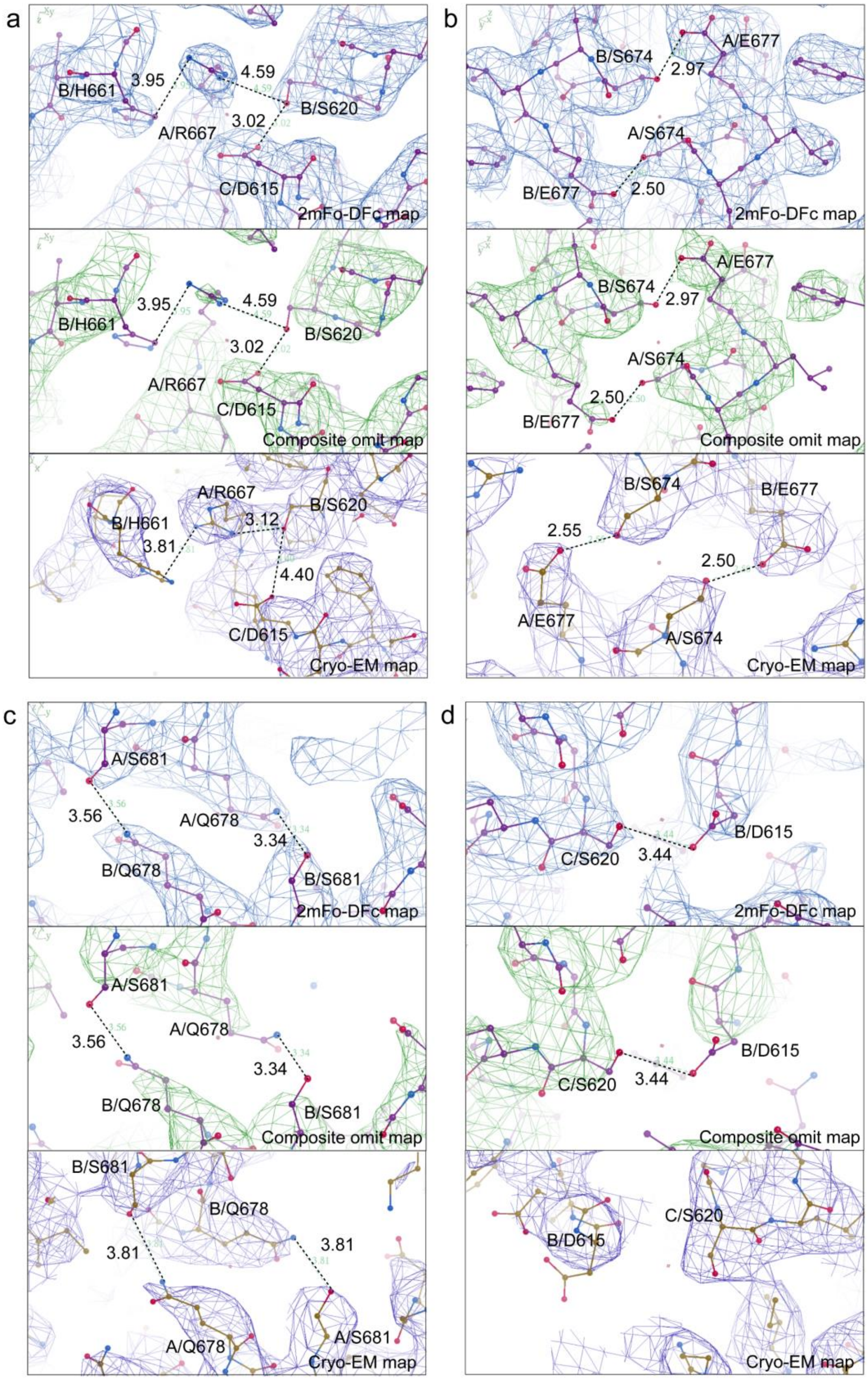

Supplementary Figure 7. Density maps depict the density details of relative amino acids in Figure 2d (a), 2e (b, c) and 2g (b, d). The 2mFo-DFc map (contoured at 1.01 σ) and composite omit map (contoured at 2.0 σ) originate from the crystal structure. The Cryo-EM map (contoured at 5.00 σ) is derived from OsCyc1^{D367A}. The images were captured using COOT.

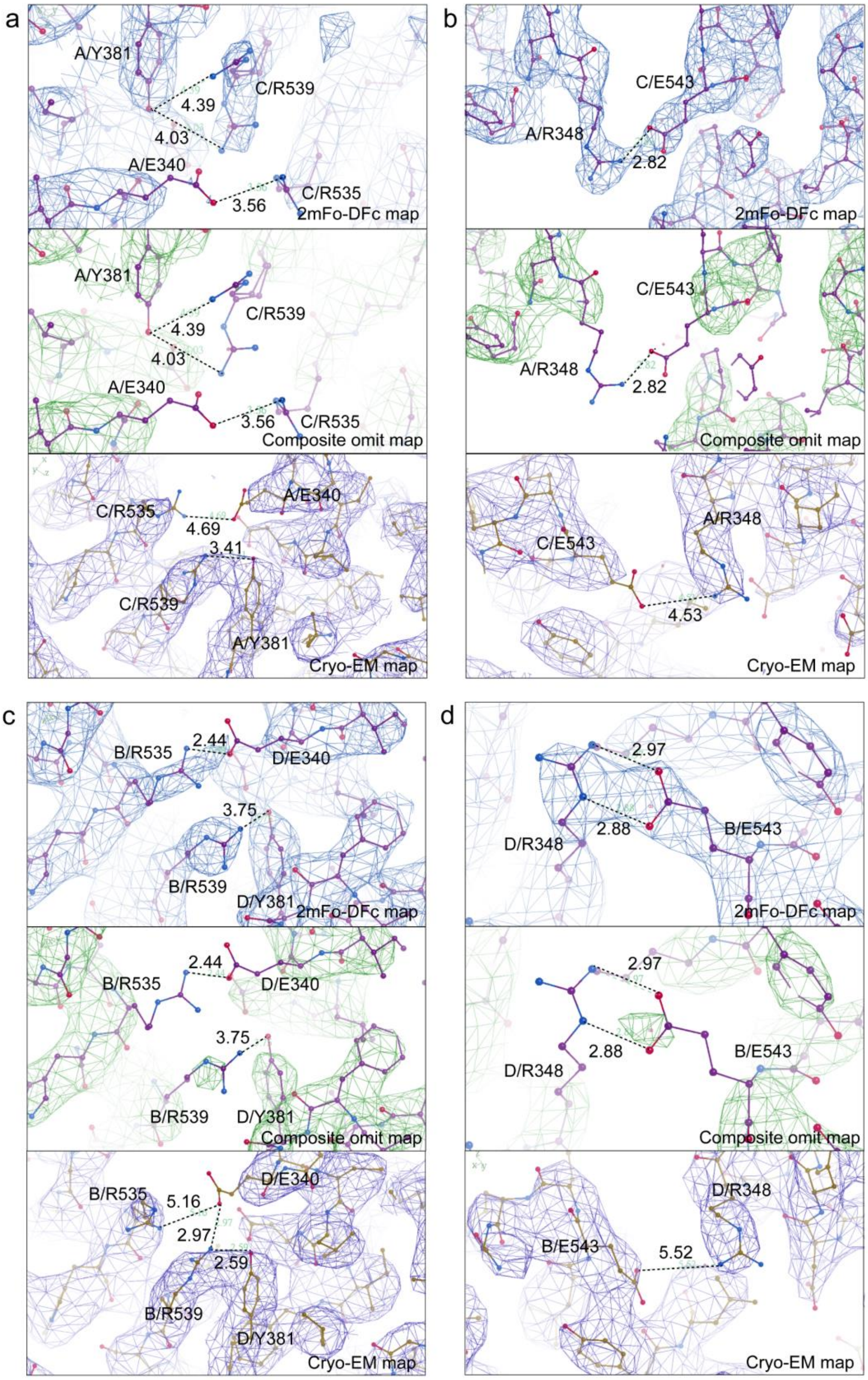

Supplementary Figure 8. Density maps depict the density details of relative amino acids in Figure 2h (a, b) and 2i (c, d). The 2mFo-DFc map (contoured at 1.01 σ) and composite omit map (contoured at 2.0 σ) originate from the crystal structure. The Cryo-EM map (contoured at 5.00 σ) is derived from OsCyc1^{D367A}. The images were captured using COOT.

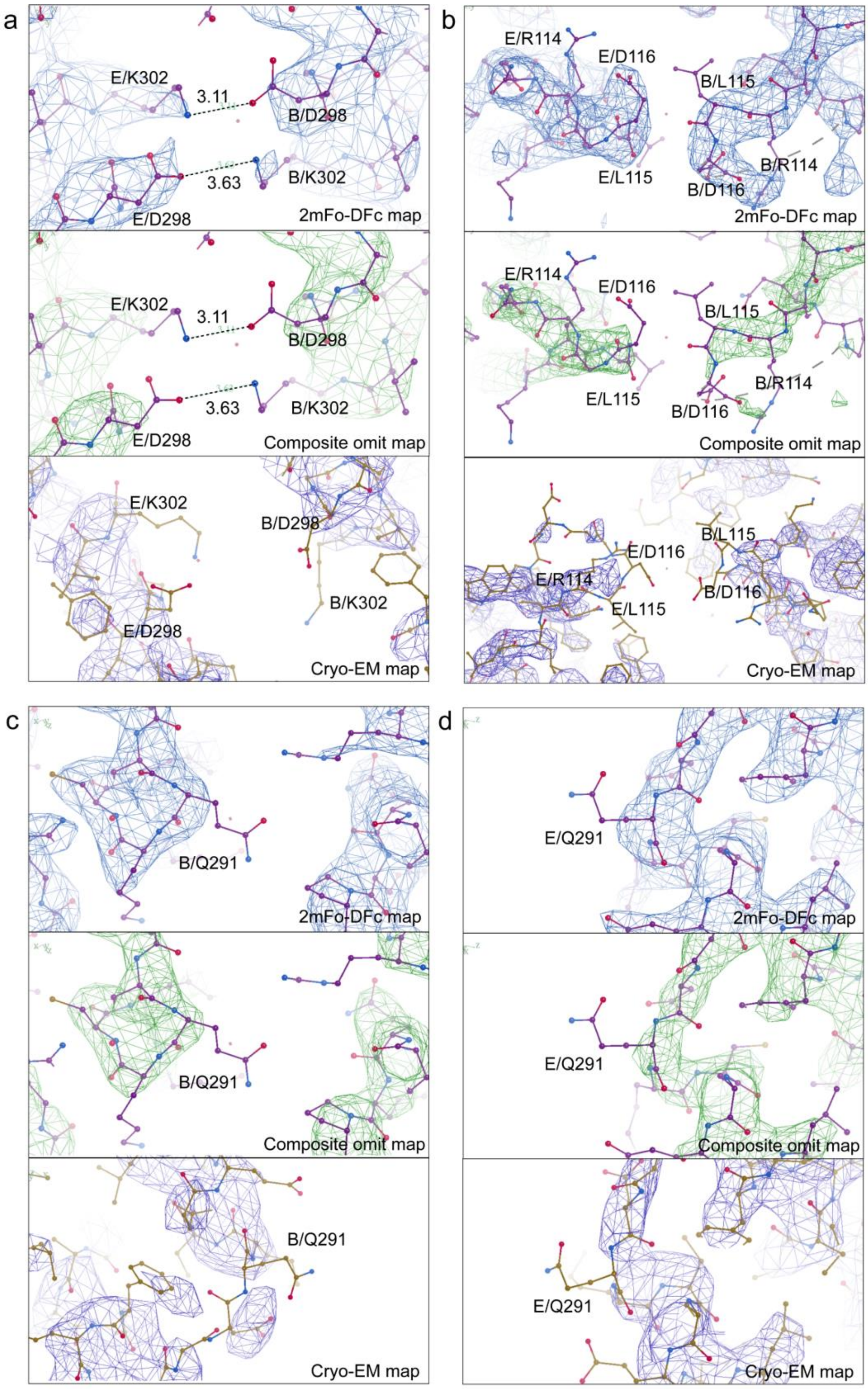

Supplementary Figure 9. Density maps depict the density details of relative amino acids in Figure 2j. The 2mFo-DFc map (contoured at 1.01 σ) and composite omit map (contoured at 2.0 σ) originate from the crystal structure. The Cryo-EM map (contoured at 5.00 σ) is derived from OsCyc1^{D367A}. The images were captured using COOT.

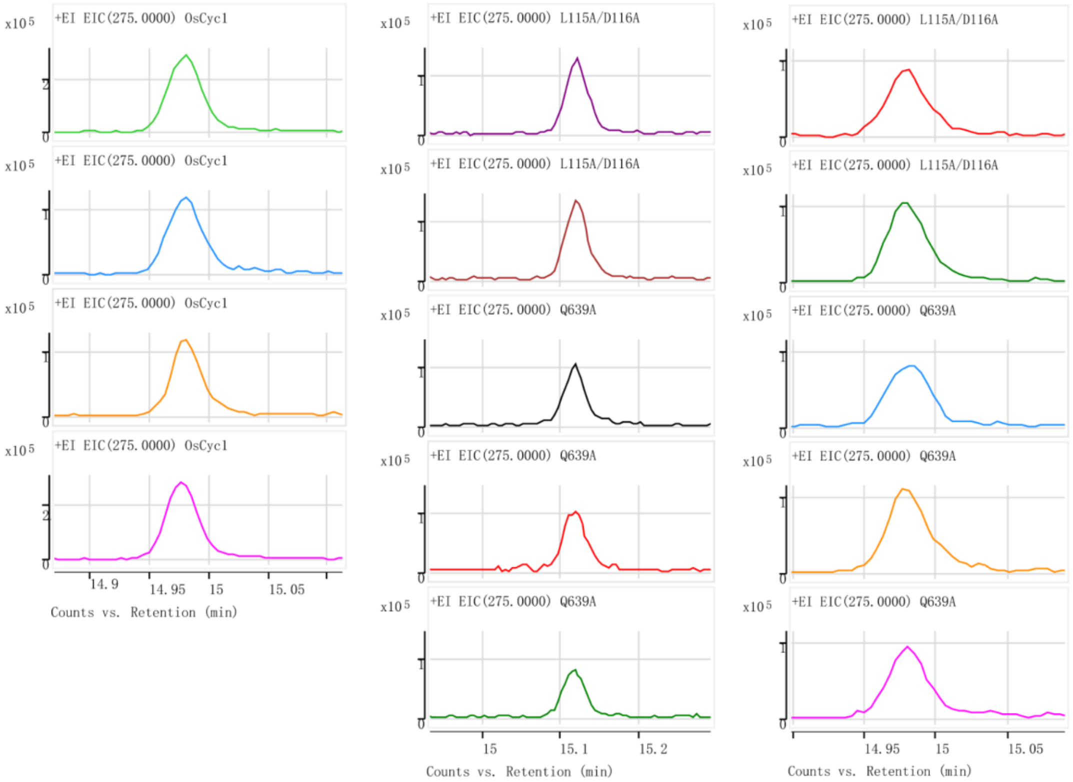
x10 5
+EI EIC(275.0000) OsCyc1
+EI EIC(275.0000) OsCyc1
+EI EIC(275.0000) OsCyc1
+EI EIC(275.0000) OsCyc1
14.9
14.95
15
15.05
Counts vs. Retention (min)
+EI EIC(275.0000) L115A/D116A
+EI EIC(275.0000) L115A/D116A
+EI EIC(275.0000) Q639A
+EI EIC(275.0000) Q639A
+EI EIC(275.0000) Q639A
15
15.1
15.2
Counts vs. Retention (min)
+EI EIC(275.0000) L115A/D116A
+EI EIC(275.0000) L115A/D116A
+EI EIC(275.0000) Q639A
+EI EIC(275.0000) Q639A
+EI EIC(275.0000) Q639A
14.95
15
15.05
Counts vs. Retention (min)

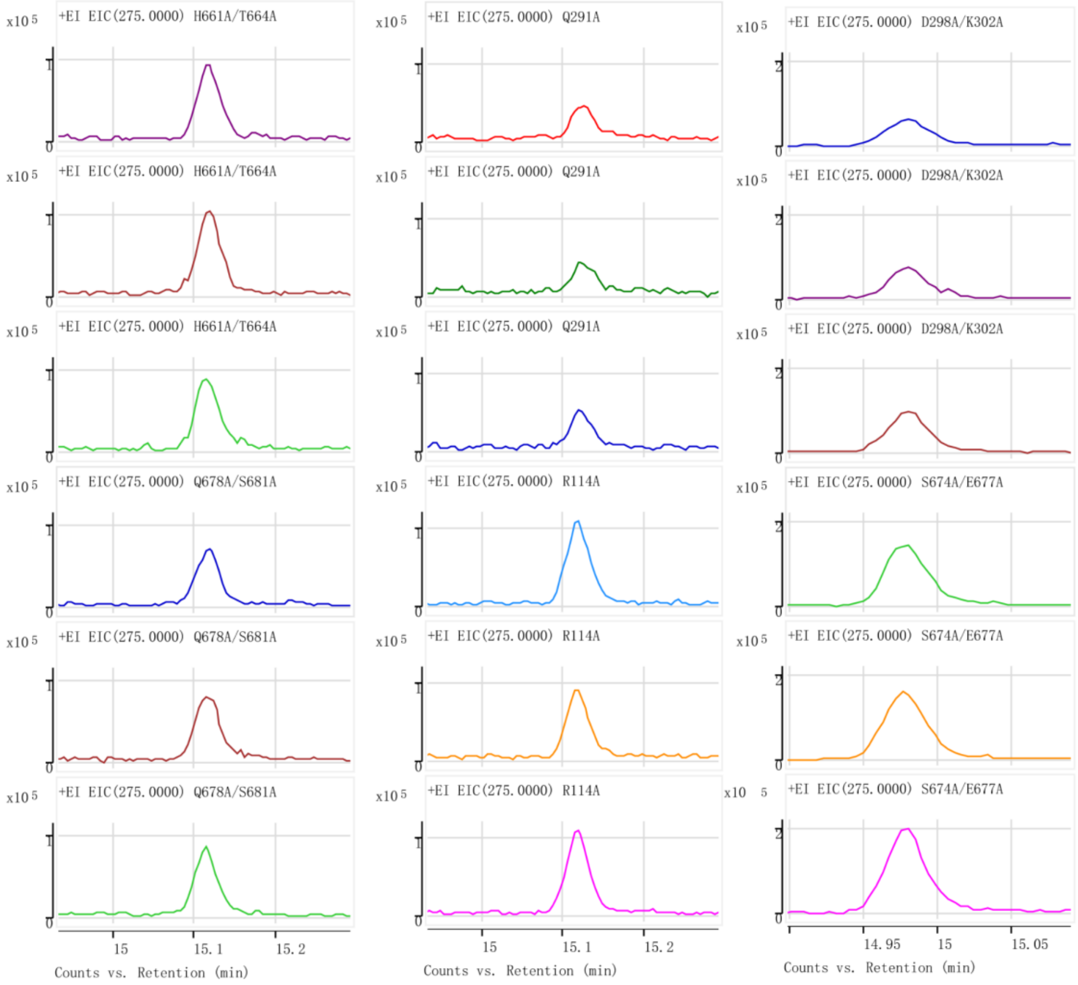
x10 5
+EI EIC(275.0000) H661A/T664A
+EI EIC(275.0000) H661A/T664A
+EI EIC(275.0000) H661A/T664A
+EI EIC(275.0000) Q678A/S681A
+EI EIC(275.0000) Q678A/S681A
+EI EIC(275.0000) Q678A/S681A
15
15.1
15.2
Counts vs. Retention (min)
+EI EIC(275.0000) Q291A
+EI EIC(275.0000) Q291A
+EI EIC(275.0000) Q291A
+EI EIC(275.0000) R114A
+EI EIC(275.0000) R114A
+EI EIC(275.0000) R114A
15
15.1
15.2
Counts vs. Retention (min)
+EI EIC(275.0000) D298A/K302A
+EI EIC(275.0000) D298A/K302A
+EI EIC(275.0000) D298A/K302A
+EI EIC(275.0000) S674A/E677A
+EI EIC(275.0000) S674A/E677A
+EI EIC(275.0000) S674A/E677A
14.95
15
15.05
Counts vs. Retention (min)

Supplementary Figure 10. Extracted ion chromatograms (m/z=275) obtained from GC-MS analysis of the products formed by OsCyc1 and various mutants related to interface of OsCyc1 oligomers. The GC-MS experiments were conducted in two separate runs, and due to differences in the length of the DB-5MS column in each batch, there were slight differences in the peak positions of *syn*-CPP. Both L115A/D116A and Q639A were replicated in both batches of experiments. Each GC-MS experiment of the products catalyzed by each mutant was performed with at least three replicates. Specifically, L115A/D116A had four replicates, while Q639A had six replicates.

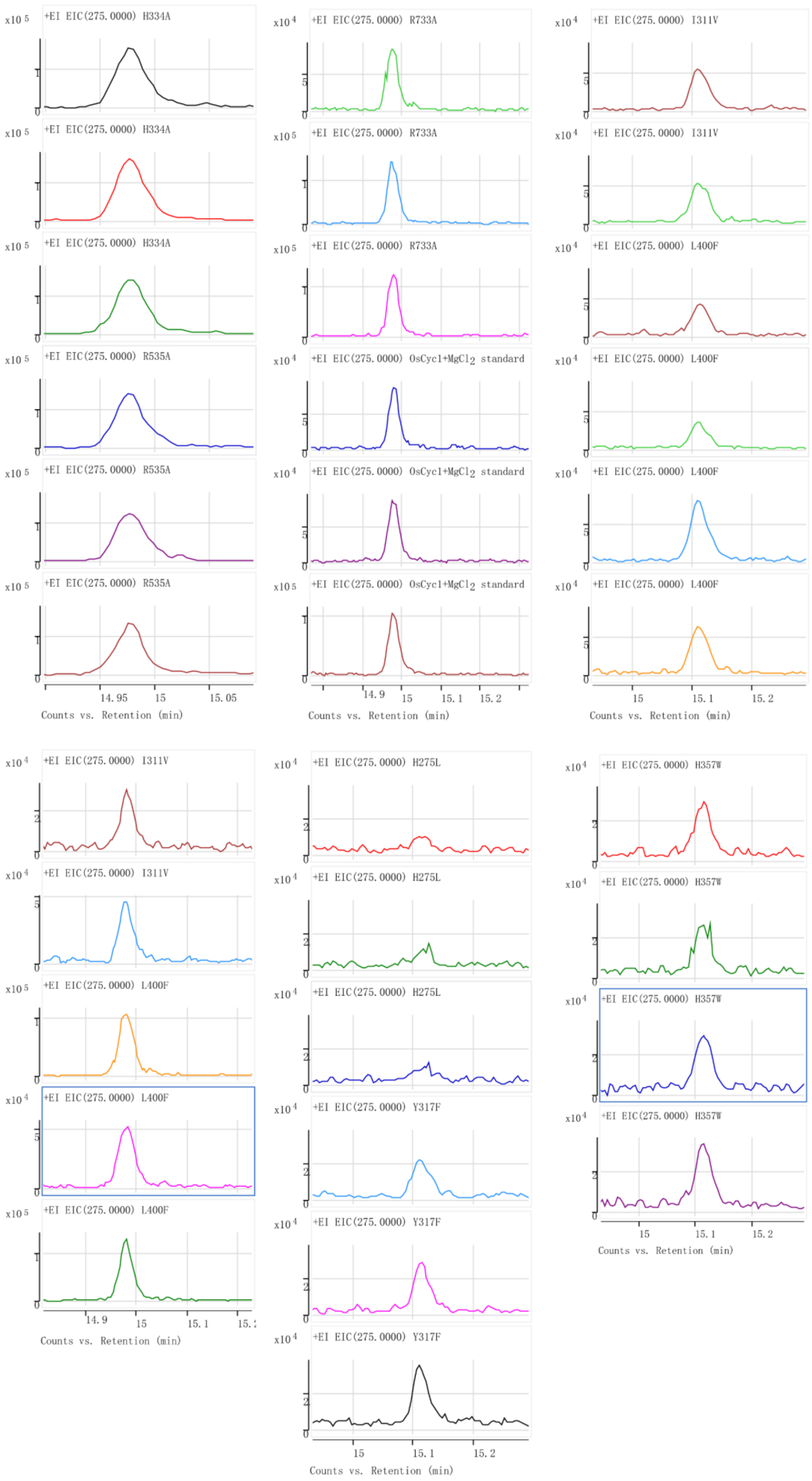
+EI EIC(275.0000) H334A
+EI EIC(275.0000) R535A
+EI EIC(275.0000) R733A
+EI EIC(275.0000) OsCyc1+MgCl2 standard
+EI EIC(275.0000) I311V
+EI EIC(275.0000) L400F
+EI EIC(275.0000) H275L
+EI EIC(275.0000) Y317F
+EI EIC(275.0000) H357W
x10 5
x10 4
14.95
15
15.05
14.9
15.1
15.2
Counts vs. Retention (min)

Supplementary Figure 11. Extracted ion chromatograms (m/z=275) obtained from GC-MS analysis of the products formed by OsCyc1 and various mutants related to surface-exposed positive residues and active pocket residues. The GC-MS experiments were conducted in two separate runs, and due to differences in the length of the DB-5MS column in each batch, there were slight variations in the peak positions of *syn*-CPP. The products produced by OsCyc1 with magnesium buffer were used as standard reference for H275L, I311V, Y317F, H357W and L400F. Both I311V and L400F were replicated in both batches of experiments. Each GC-MS experiment of the products catalyzed by each mutant was performed with at least three replicates. Specifically, both I311V and H357W had four replicates, while L400F had seven replicates.

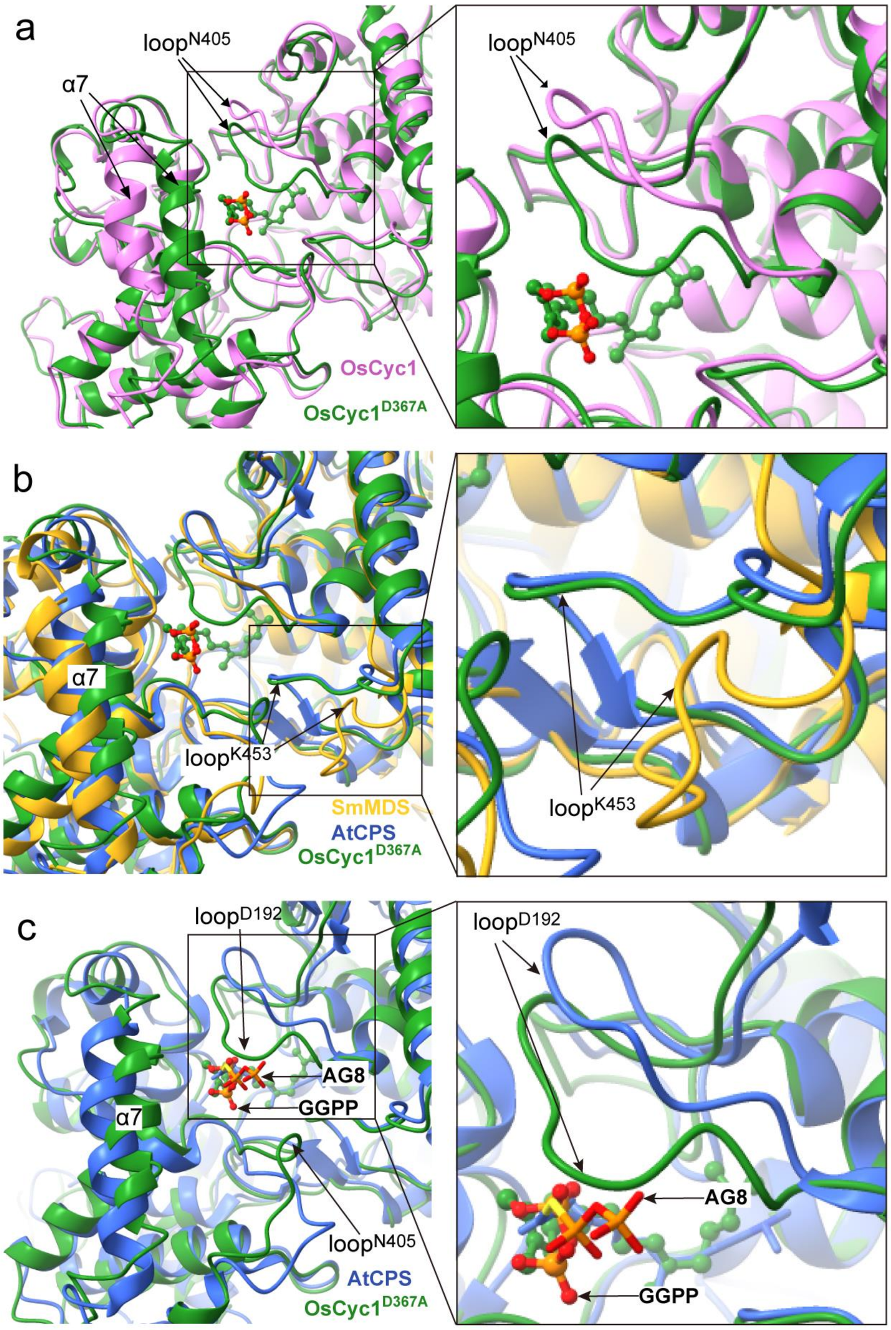

Supplementary Figure 12. The magnified views of Figure 4. (a) The magnified view of Figure 4a. Active pocket comparison of OsCyc1 (orchid) and OsCyc1D367A (forest green). (b) The magnified view of Figure 4c. Comparison of the structures in their ligand binding states of SmMDS (goldenrod, PDB ID: 4Y47), OsCyc1D367A (forest green) and AtCPS (royal blue, PDB ID: 3PYA). (c) The magnified view of Figure 4d. Comparison of active site pocket structures of OsCyc1D367A (forest green) and AtCPS (royal blue, PDB ID: 3PYA).

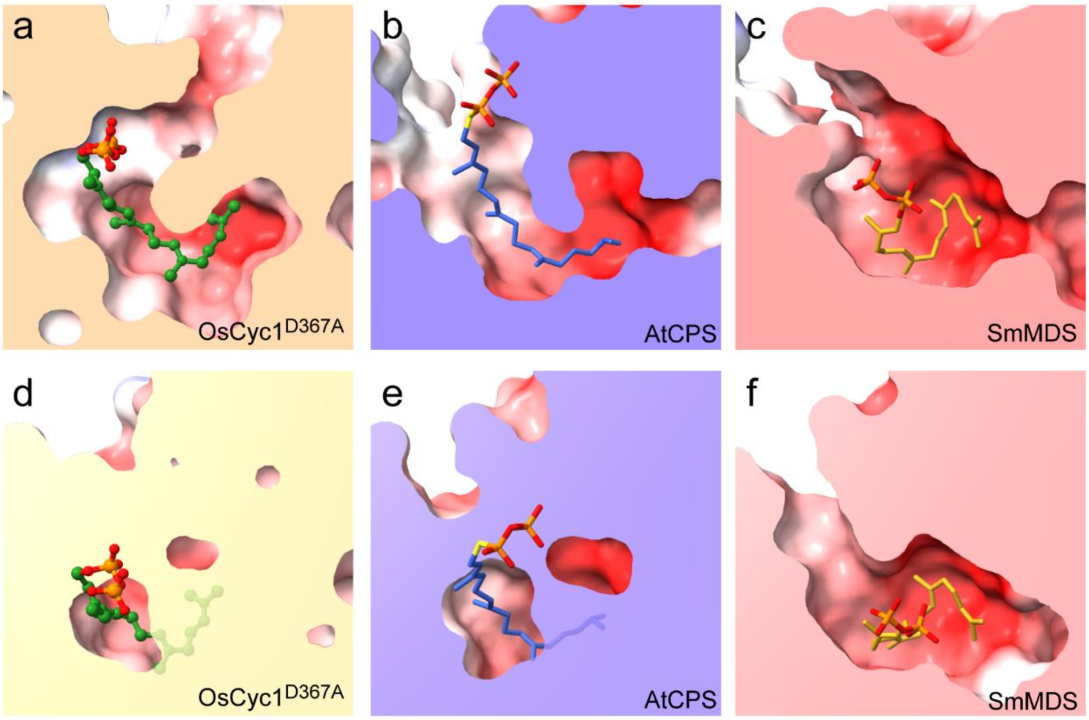

Supplementary Figure 13. Electro potential map surface of OsCyc1D367A, AtCPS (PDB: 3PYA) and SmMDS (PDB: 4Y47). (a, b, c) Section views of active pockets. (d, e, f) Top views of active pockets. The red is for negative potential, white at zero, and blue is for positive in the electro potential map.

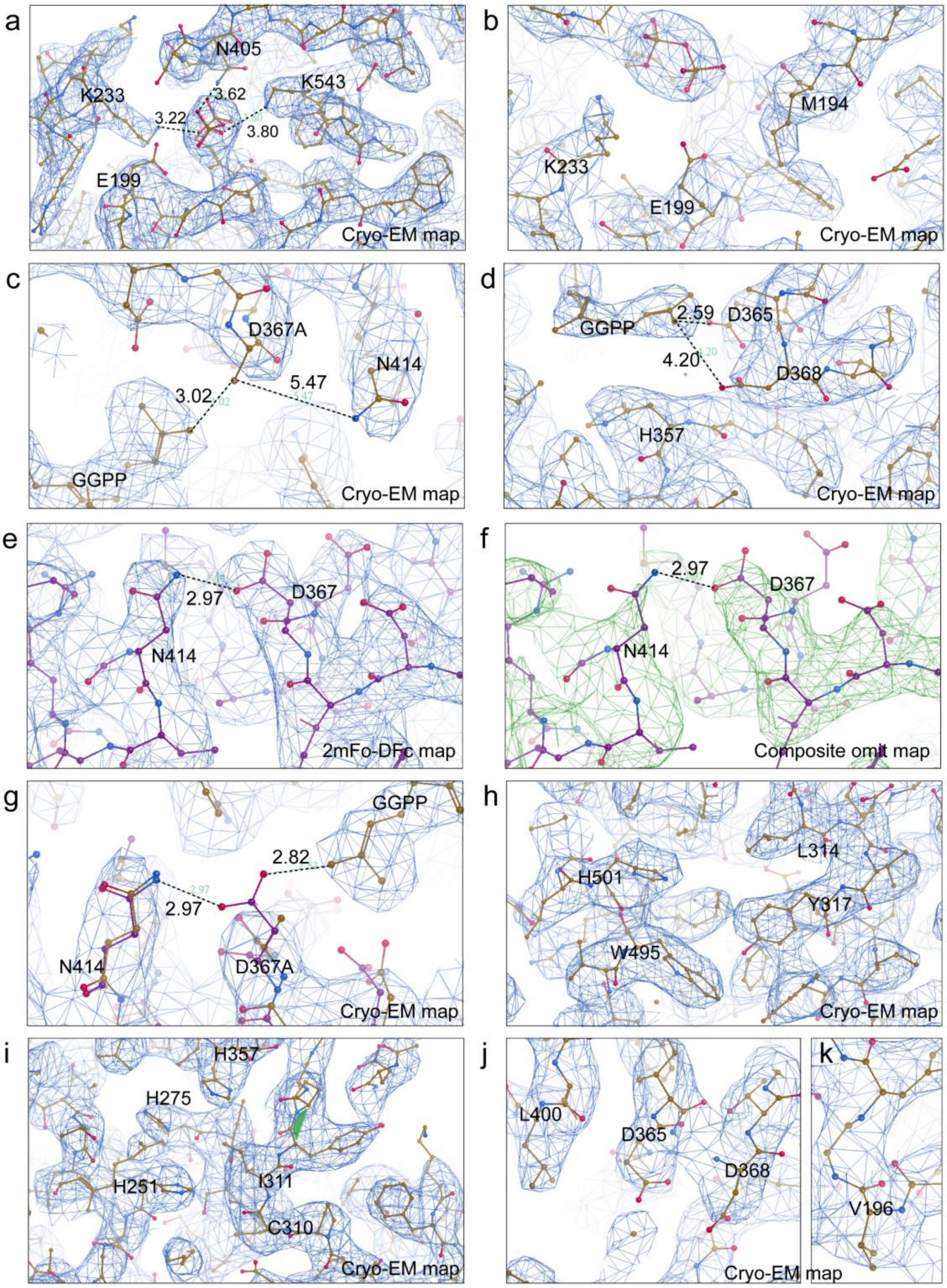

Supplementary Figure 14. Density maps depict the density details of relative amino acids in active site. The 2mFo-DFc map (contoured at 1.01 σ) and composite omit map (contoured at 2.0 σ) originate from the chain B of crystal structure. The Cryo-EM map (contoured at 5.00 σ) is derived from chain D of OsCyc1^{D367A}. The crystal structure of OsCyc1 is represented in purple, while the structure of OsCyc1^{D367A} is depicted in yellow. Images were captured using COOT.

a

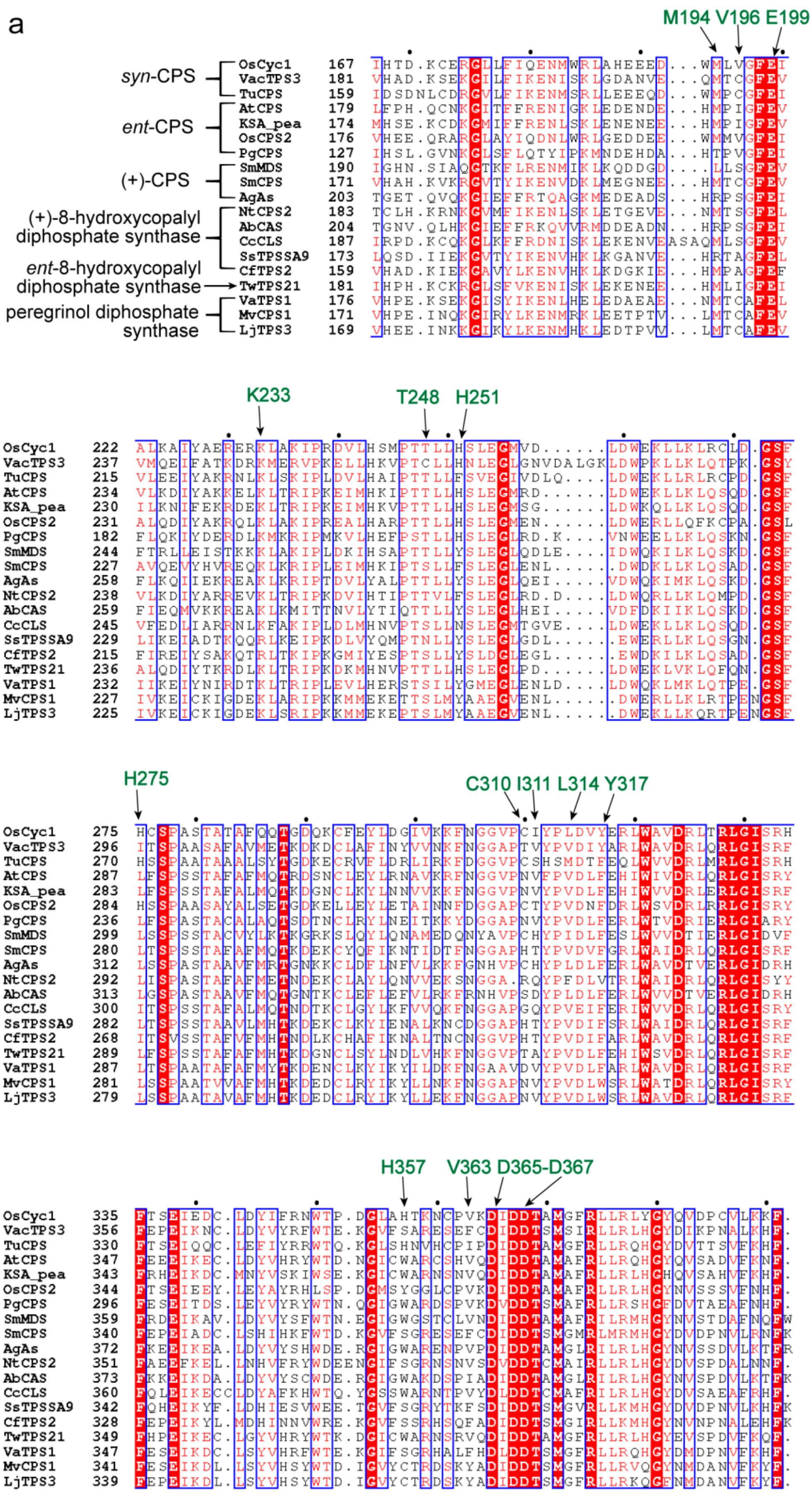
syn-CPS
ent-CPS
(+)-CPS
(+)-8-hydroxycopalyl diphosphate synthase
ent-8-hydroxycopalyl diphosphate synthase
peregrinol diphosphate synthase
OsCyc1
VacTPS3
TuCPS
AtCPS
KSA_pea
OsCPS2
PgCPS
SmMDS
SmCPS
AgAs
NtCPS2
AbCAS
CcCLS
SsTPSSA9
CfTPS2
TwTPS21
VaTPS1
MvCPS1
LjTPS3
M194 V196 E199
K233
T248
H251
H275
C310 I311 L314 Y317
H357
V363 D365-D367

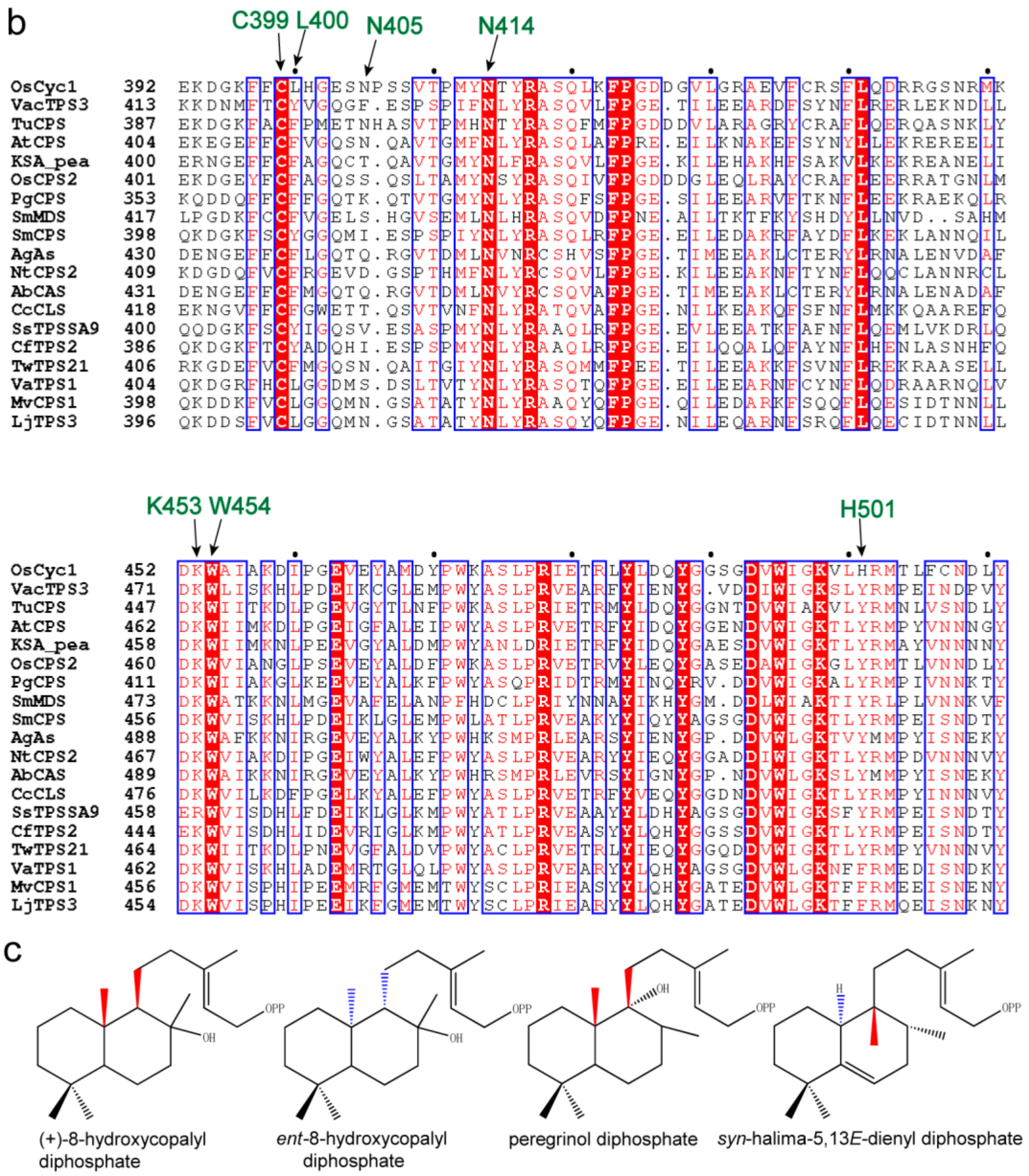

Supplementary Figure 15. Sequence alignment of OsCyc1 and its homologs. (a, b) The sequence alignment of *syn*-CPSs, *ent*-CPSs, (+)-CPSs, (+)-8-hydroxycopalyl diphosphate synthases, *ent*-8-hydroxycopalyl diphosphate synthase and peregrinol diphosphate synthases. **VacTPS3** (*syn*-copalyl diphosphate synthases from *Vitex agnus-castus*. UniProtKB: A0A2K9RFZ8) and TuCPS (*syn*-copalyl diphosphate synthases from *Triticum urartu*. UniProtKB: A0A8R7RGM7) are *syn*-copalyl diphosphate synthases. ***At*CPS**, KSA_pea (*Ent*-copalyl diphosphate synthase from *Pisum sativum*, UniProtKB: O04408), *Os*CPS2 (*Ent*-copalyl diphosphate synthase 2 from *Oryza sativa subsp. Japonica*, UniProtKB: Q6Z5I0) and *Pg*CPS (*Ent*-copalyl diphosphate synthase from *Picea glauca*, UniProtKB: D2X8G0) are monofunctional *ent*-copalyl diphosphate synthases. **SmCPS** is copalyl diphosphate synthase and comes from *Salvia miltiorrhiza*. **NtCPS2** (UniProtKB: G3CCC0), AbCAS (UniProtKB: H8ZM73), CcCLS (UniProtKB: E2IHE0), SsTPSSA9 (UniProtKB: G8GJ96), CfTPS2 (UniProtKB: X4ZWN5) are (+)-8-hydroxycopalyl diphosphate synthases. **TwTPS21** (UniProtKB: A0A0M4MZ71) is *ent*-8-hydroxycopalyl diphosphate synthase. VaTPS1 (UniProtKB: A0A2K9RFZ7), MvCPS1 (UniProtKB: A0A075FAK4) and LjTPS3 (UniProtKB: A0A976XVV4) are peregrinol diphosphate synthases. Sequence alignment was generated using ESPript3.0[2] and key

residues are indicated. The η symbol refers to a 3_{10}-helix. α-Helices, 3_{10}-helices and π-helices are displayed as medium, small and large squiggles, respectively. β-strands are rendered as arrows, strict β-turns as TT letters and strict α-turns as TTT. Every 10th residue is indicated with a dot placed above the sequences. (c) The molecular structures of (+)-8-hydroxycopalyl diphosphate, *ent*-8-hydroxycopalyl diphosphate, peregrinol diphosphate and *syn*-halima-5,13*E*-dienyl diphosphate.

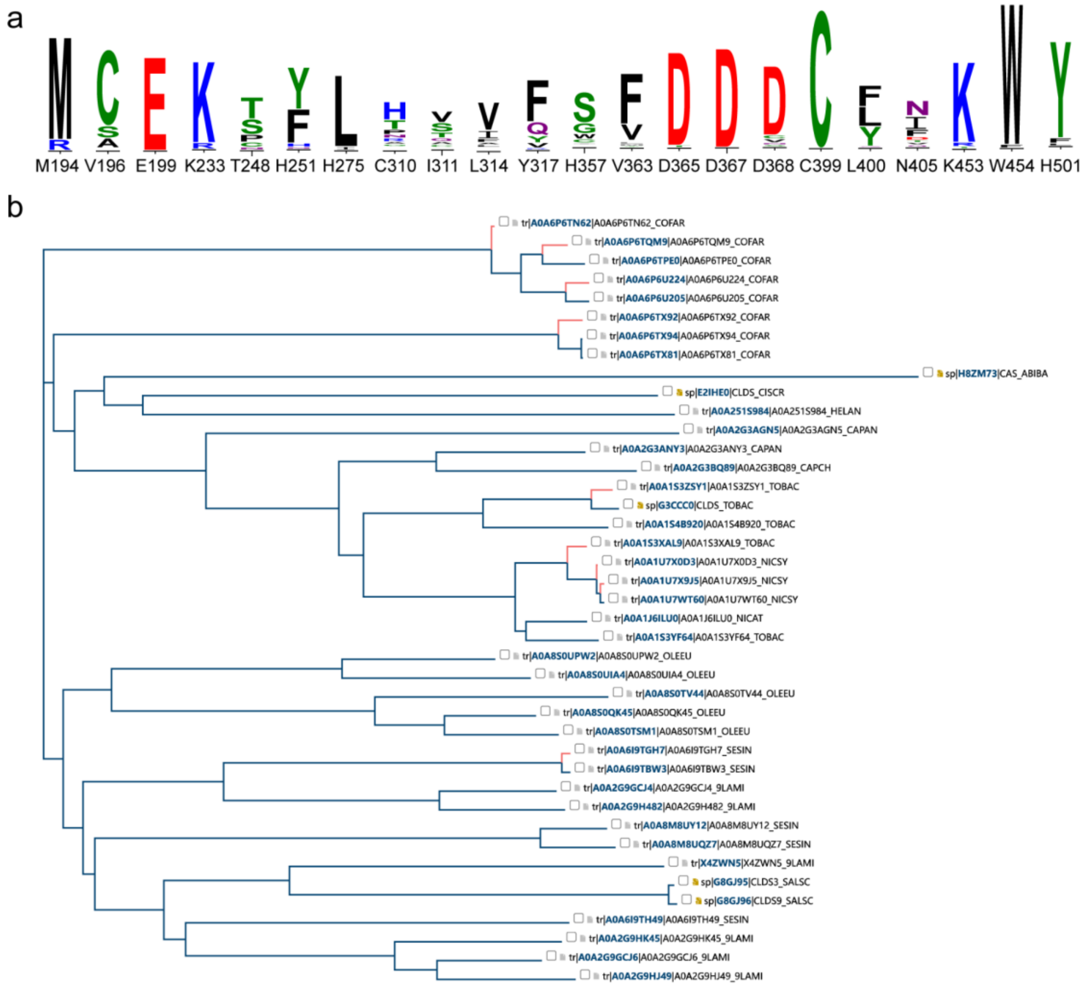

Supplementary Figure 16. Sequence alignment of class II diterpene synthases that produce 8-hydroxycopalyl diphosphate. (a) Sequence logos highlighting the conservation of amino acids corresponding to the residues around the active site of OsCyc1. (b) The phylogenetic tree of 8-hydroxycopalyl diphosphate synthases for sequence analysis. This is a neighbour-joining tree without distance corrections and is drawn on Uniprot website (https://www.uniprot.org/align).

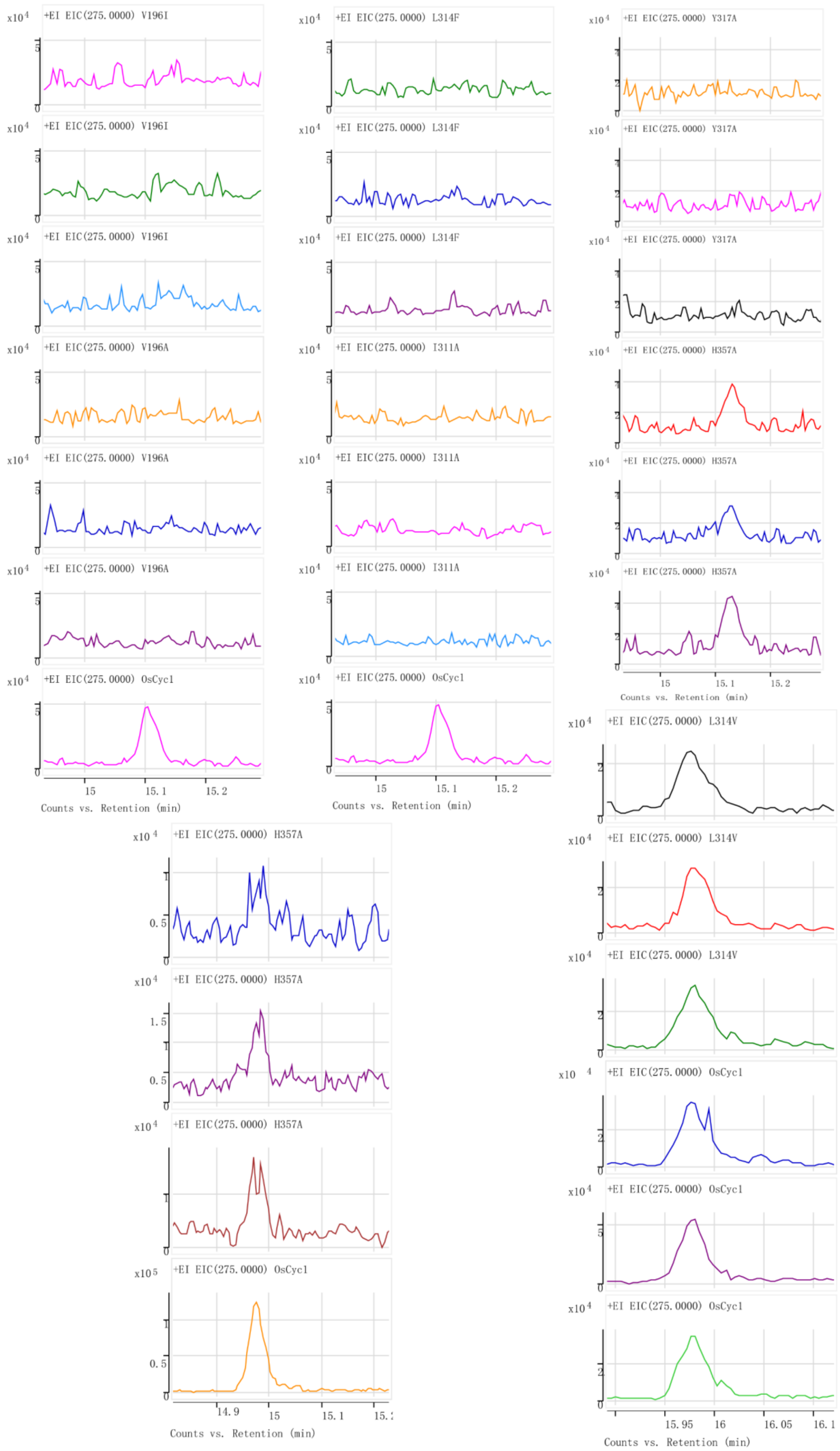
x10⁴ +EI EIC(275.0000) V196I
x10⁴ +EI EIC(275.0000) V196I
x10⁴ +EI EIC(275.0000) V196I
x10⁴ +EI EIC(275.0000) V196A
x10⁴ +EI EIC(275.0000) V196A
x10⁴ +EI EIC(275.0000) V196A
x10⁴ +EI EIC(275.0000) OsCyc1
15 15.1 15.2
Counts vs. Retention (min)
x10⁴ +EI EIC(275.0000) L314F
x10⁴ +EI EIC(275.0000) L314F
x10⁴ +EI EIC(275.0000) L314F
x10⁴ +EI EIC(275.0000) I311A
x10⁴ +EI EIC(275.0000) I311A
x10⁴ +EI EIC(275.0000) I311A
x10⁴ +EI EIC(275.0000) OsCyc1
15 15.1 15.2
Counts vs. Retention (min)
x10⁴ +EI EIC(275.0000) Y317A
x10⁴ +EI EIC(275.0000) Y317A
x10⁴ +EI EIC(275.0000) Y317A
x10⁴ +EI EIC(275.0000) H357A
x10⁴ +EI EIC(275.0000) H357A
x10⁴ +EI EIC(275.0000) H357A
15 15.1 15.2
Counts vs. Retention (min)
x10⁴ +EI EIC(275.0000) H357A
x10⁴ +EI EIC(275.0000) H357A
x10⁴ +EI EIC(275.0000) H357A
x10⁵ +EI EIC(275.0000) OsCyc1
14.9 15 15.1
Counts vs. Retention (min)
x10⁴ +EI EIC(275.0000) L314V
x10⁴ +EI EIC(275.0000) L314V
x10⁴ +EI EIC(275.0000) L314V
x10⁴ +EI EIC(275.0000) OsCyc1
x10⁴ +EI EIC(275.0000) OsCyc1
x10⁴ +EI EIC(275.0000) OsCyc1
15.95 16 16.05 16.1
Counts vs. Retention (min)

Supplementary Figure 17. Extracted ion chromatograms (m/z=275) obtained from GC-MS analysis of the products formed by OsCyc1 and various mutants related to active pocket residues. The GC-MS experiments were conducted in three separate runs, and due to differences in the length of the DB-5MS column in each batch, there were slight variations in the peak positions of *syn*-CPP. Each GC-MS experiment of the products catalyzed by each mutant was performed with at least three replicates. H357A was replicated in two batches of experiments and had six replicates.

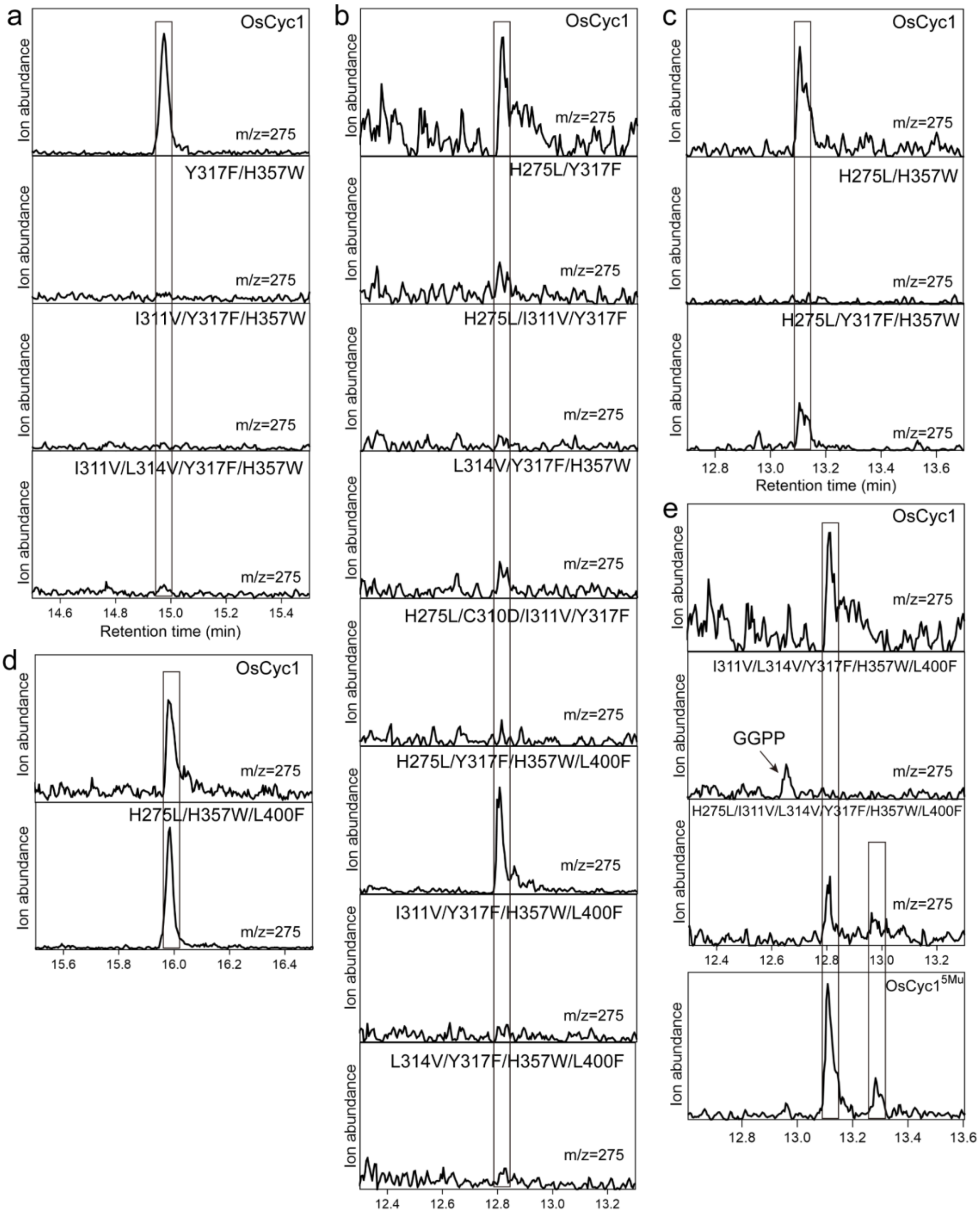

Supplementary Figure 18. Extracted ion chromatograms (m/z=275) obtained from GC-MS analysis of the products formed by various mutants. The GC-MS experiments were conducted in different separate runs, and due to differences in the length of the DB-5MS column in each batch, there were slight differences in the peak positions of *syn*-CPP. In each batch of GC-MS experiments, the product catalyzed by OsCyc1 was utilized as the standard reference.

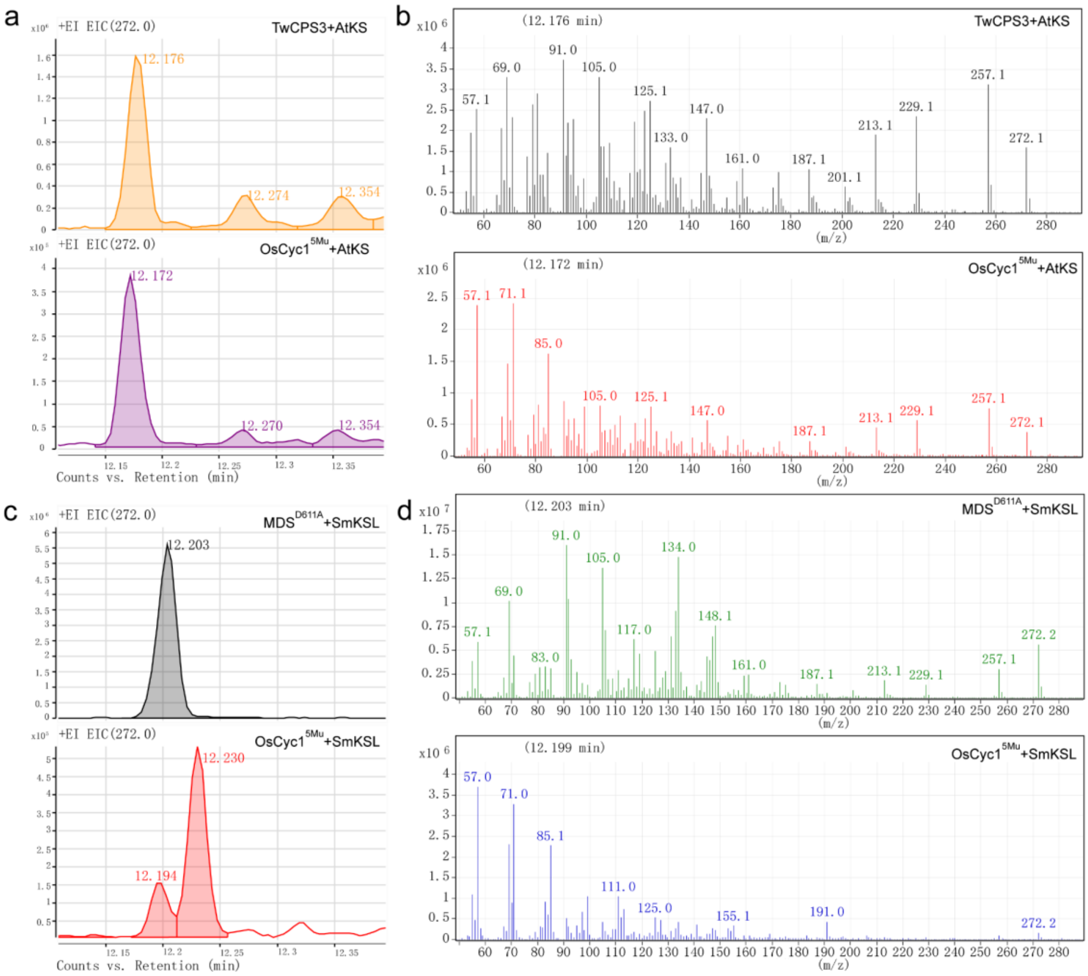

Supplementary Figure 19. Extracted ion chromatograms (m/z=272) obtained from GC-MS analysis of the products formed by related enzymes. (a) GC-MS analysis (272 m/z extracted ion chromatographs) of the products from TwCPS3 and AtKS and OsCyc1^{5Mu} and AtKS. (b) Mass spectrum of the GC-MS 272 m/z chromatograph peak. The background mass spectrometry was subtracted from the mass spectrometry. (c) GC-MS analysis (272 m/z extracted ion chromatographs) of the products from MDS^{D611A} and SmKSL, and OsCyc1^{5Mu} and SmKSL. (d) Mass spectrum of the GC-MS 272 m/z chromatograph peak. The background mass spectrometry was subtracted from the mass spectrometry.

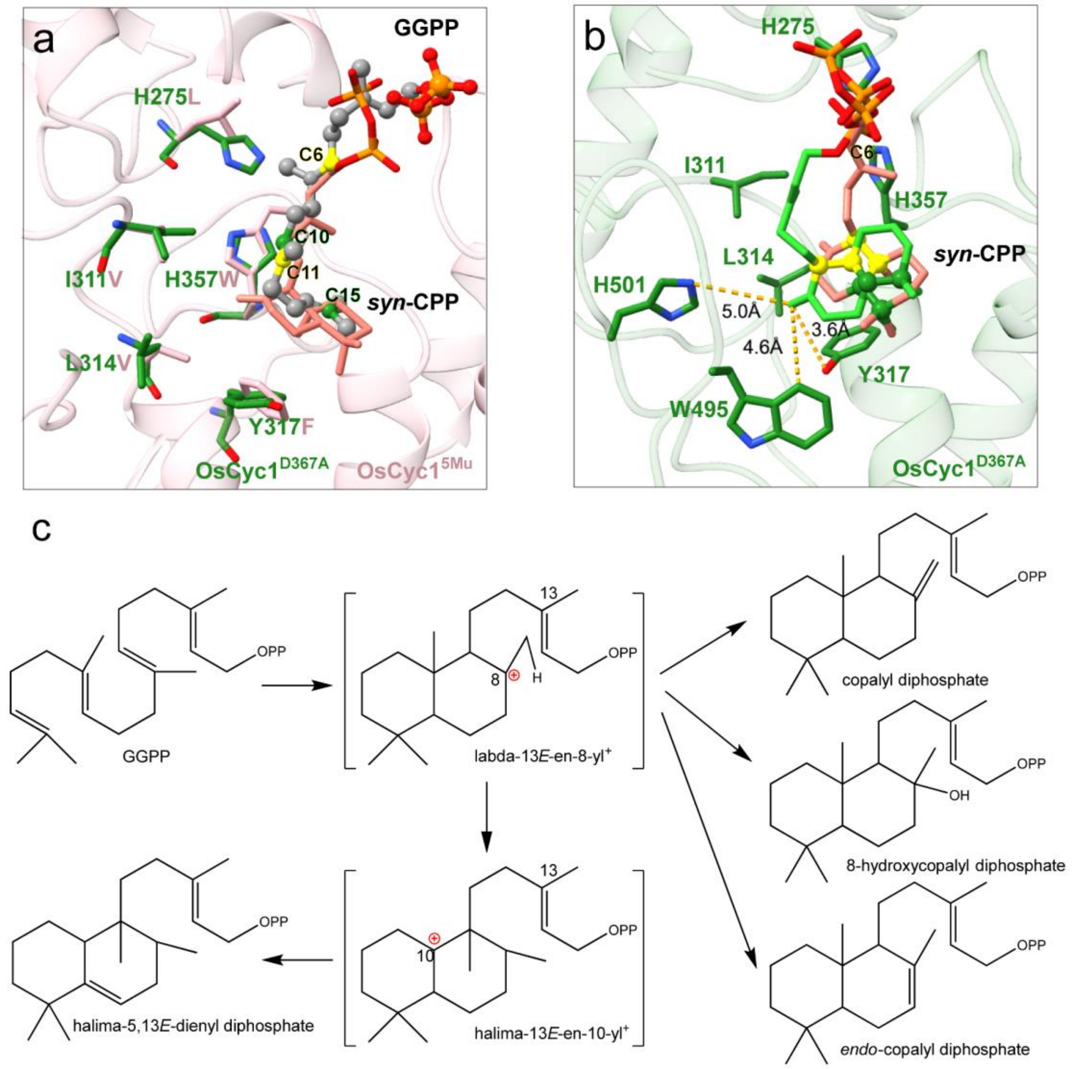

Supplementary Figure 20. Molecular docking and cyclization mechanism of GGPP to form various intermediates. (a) A superposition diagram showing the relative positions of GGPP (from the structure of OsCyc1^{D367A}) and *syn*-CPP (from the docking result with OsCyc1^{5Mu} as input). Atoms forming the new bonds are highlighted in yellow (C6 and C11) and forest green (C10 and C15). (b) Superposition of *syn*-CPP in the docking results with OsCyc1^{5Mu} and OsCyc1^{D367A} as inputs. In the docking result with OsCyc1^{5Mu}, *syn*-CPP is represented in salmon, while in the docking result with OsCyc1^{D367A}, *syn*-CPP is shown in lime green. (c) A schematic diagram illustrating the process of diterpene synthase catalyzing GGPP to form different intermediates. The intermediate labda-13*E*-en-8-yl$^+$ can be quenched to produce copalyl diphosphate, 8-hydroxycopalyl diphosphate, and *endo*-copalyl diphosphate. If the labda-13*E*-en-8-yl$^+$ intermediate is not quenched, the carbon cation continues to transfer, leading to the formation of the intermediate halima-13*E*-en-10-yl$^+$, which subsequently quenches to generate halima-5,13*E*-dienyl diphosphate.

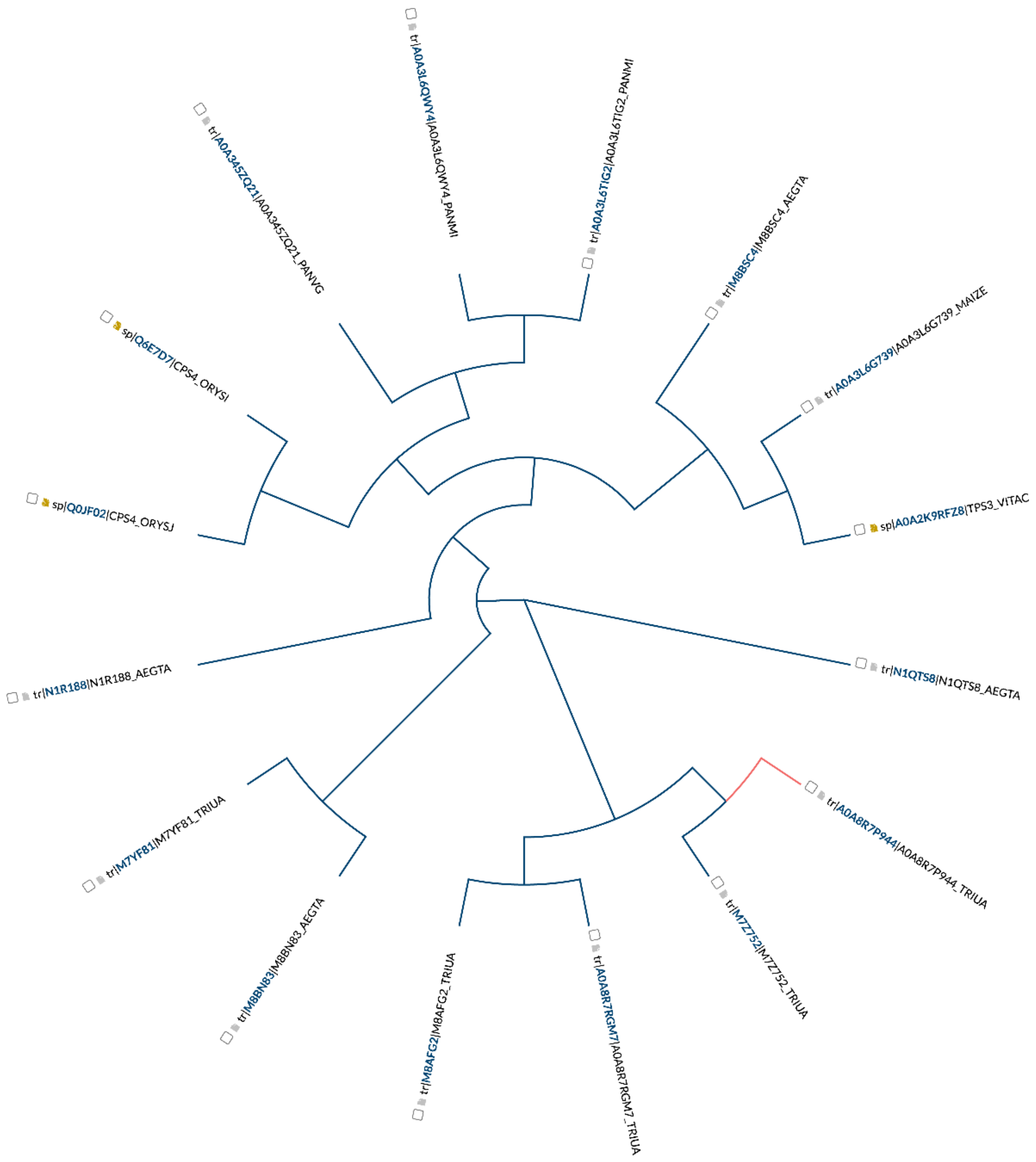

Supplementary Figure 21. The phylogenetic tree of syn-copalyl diphosphate synthases for sequence analysis. The phylogenetic tree consists of 16 protein sequences. This is a neighbour-joining tree without distance corrections and is drawn on Uniprot website (https://www.uniprot.org/align).

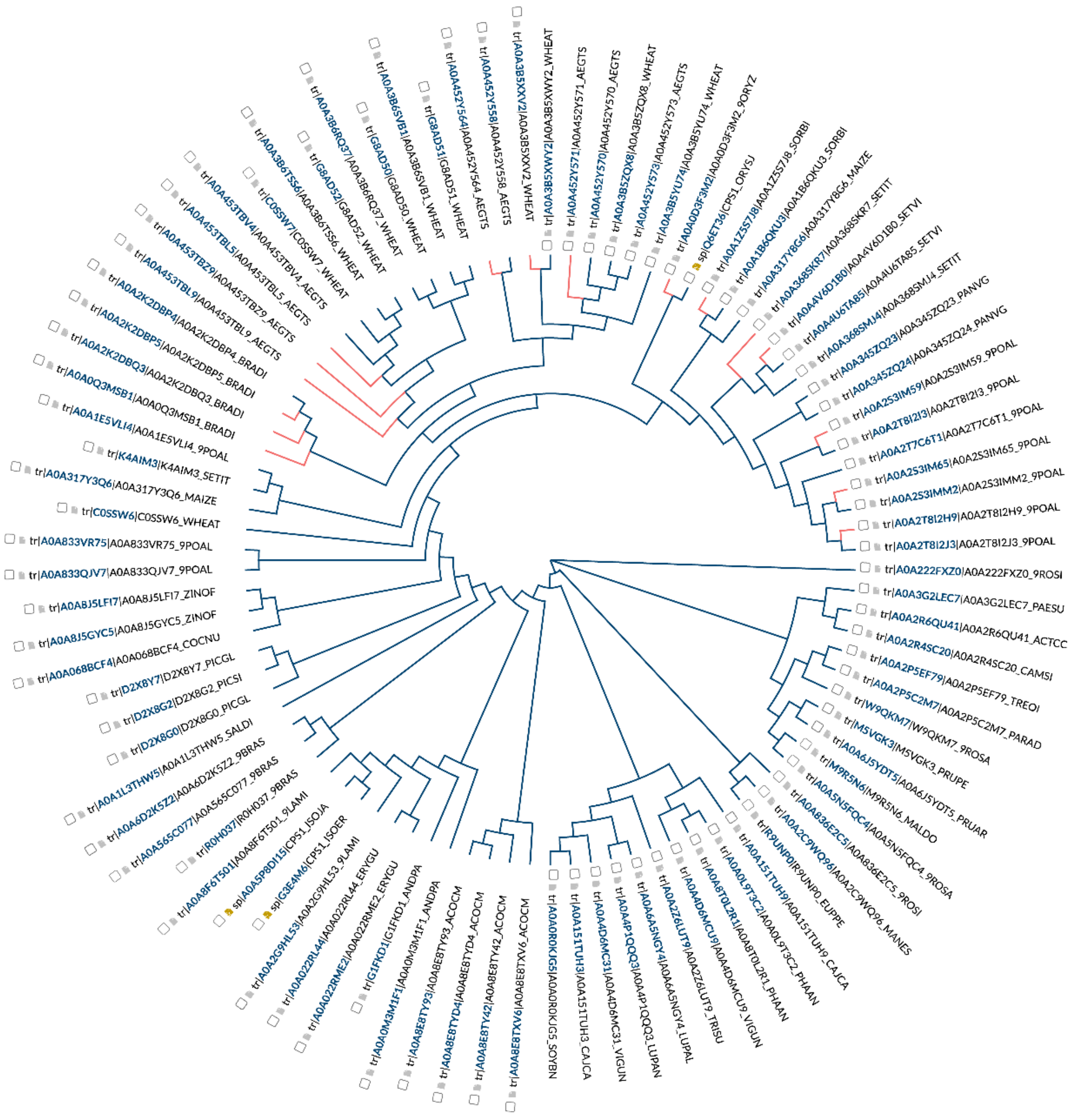

Supplementary Figure 22. The phylogenetic tree of ent-copalyl diphosphate synthases for sequence analysis. The phylogenetic tree consists of 94 protein sequences. This is a neighbour-joining tree without distance corrections and is drawn on the UniProt website (https://www.uniprot.org/align).

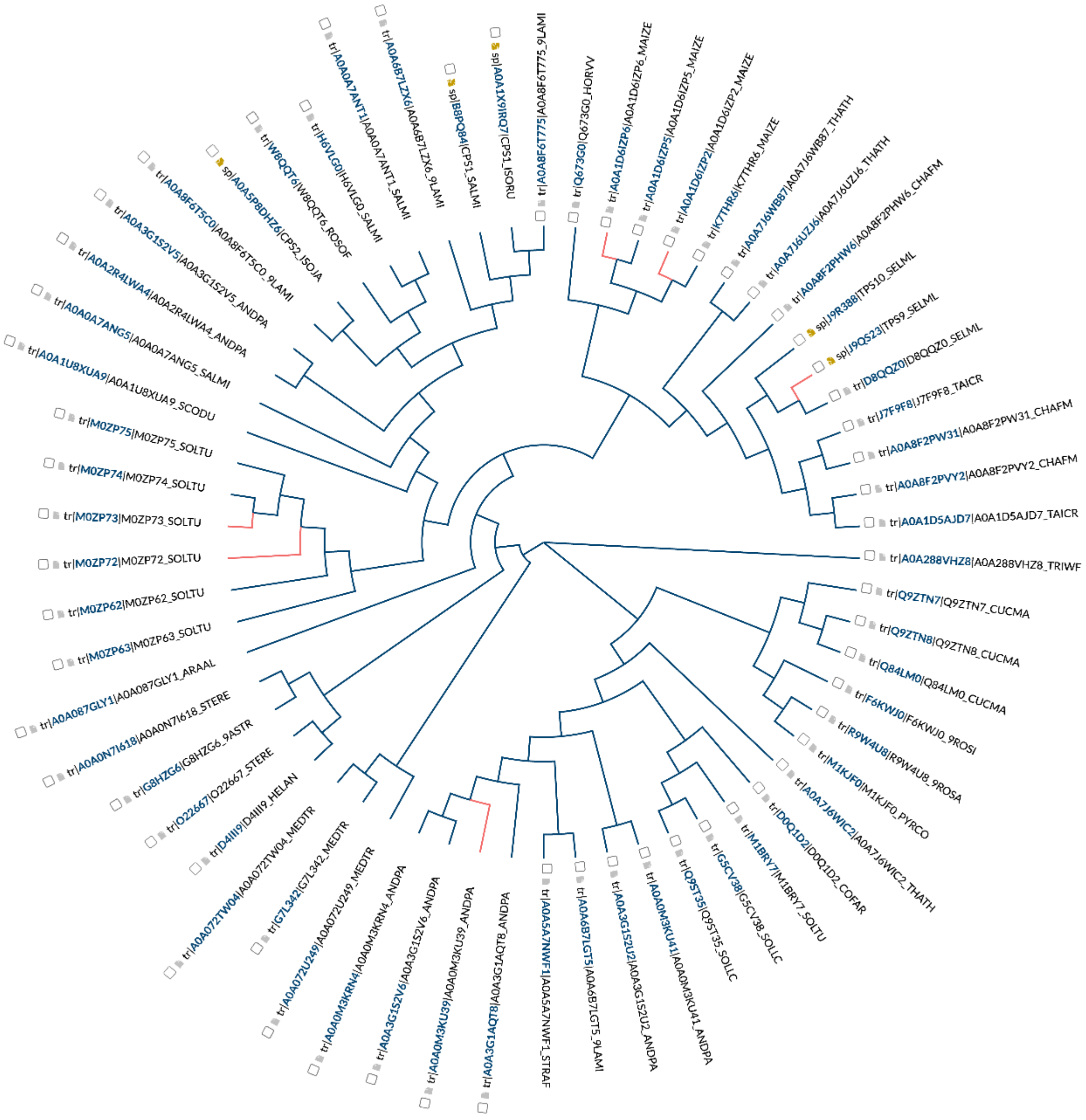

Supplementary Figure 23. The phylogenetic tree of (+)-copalyl diphosphate synthases for sequence analysis. The phylogenetic tree consists of 62 protein sequences. This is a neighbour-joining tree without distance corrections and is drawn on the UniProt website (https://www.uniprot.org/align).

Supplementary References

1. Afonine, P.V. *et al.* Real-space refinement in PHENIX for cryo-EM and crystallography. *Acta crystallographica. Section D, Structural biology* **74**, 531-544 (2018).
2. Robert, X. & Gouet, P. Deciphering key features in protein structures with the new ENDscript server. *Nucleic Acids Research* **42**, W320-W324 (2014).
