# Supplementary material for: Structural and functional investigations of syn-copalyl diphosphate synthase from Oryza sativa
Source: Commun Chem. 2023 Nov 6;6:240. doi: 10.1038/s42004-023-01042-w (PMC10628199; doi:10.1038/s42004-023-01042-w)
Supplement: Supplementary file 3 — Description of additional supplementary file [file 42004_2023_1042_MOESM3_ESM.pdf]

Description of Additional Supplementary Files

File name: Supplementary Data 1

Description: PDB file of crystal structure

File name: Supplementary Data 2

Description: The validation report for the crystal structure， 8KBW.

File name: Supplementary Data 3

Description: The validation report for the cryo-EM structure of the dimer.

File name: Supplementary Data 4

Description: The validation report for the cryo-EM structure of the tetramer.

File name: Supplementary Data 5

Description: The validation report for the cryo-EM structure of the hexamer.

File name: Supplementary Data 6

Description: The validation report for the cryo-EM structure of the $OsCyc1^{D367A}$.

File name: Supplementary Data 7

Description: The source data underlying the graphs and charts.
