# Supplementary material for: Structural and functional investigations of syn-copalyl diphosphate synthase from Oryza sativa
Source: Commun Chem. 2023 Nov 6;6:240. doi: 10.1038/s42004-023-01042-w (PMC10628199; doi:10.1038/s42004-023-01042-w)
Supplement: Supplementary file 5 — Supplementary Data 2 [file 42004_2023_1042_MOESM5_ESM.pdf]

Full wwPDB X-ray Structure Validation Report (i)

Aug 17, 2023 – 08:02 PM JST

PDB ID	:	8KBW
Title	:	The crystal structure of syn-copalyl diphosphate synthase from Oryza sativa
Deposited on	:	2023-08-04
Resolution	:	3.49 Å(reported)

This wwPDB validation report is for manuscript review

This is a Full wwPDB X-ray Structure Validation Report.

This report is produced by the wwPDB biocuration pipeline after annotation of the structure.

We welcome your comments at *validation@mail.wwpdb.org*
A user guide is available at
https://www.wwpdb.org/validation/2017/XrayValidationReportHelp
with specific help available everywhere you see the (i) symbol.

The types of validation reports are described at
http://www.wwpdb.org/validation/2017/FAQs#types.

The following versions of software and data (see references (i)) were used in the production of this report:

MolProbity	:	4.02b-467
Xtriage (Phenix)	:	1.13
EDS	:	2.35
Percentile statistics	:	20191225.v01 (using entries in the PDB archive December 25th 2019)
Refmac	:	5.8.0158
CCP4	:	7.0.044 (Gargrove)
Ideal geometry (proteins)	:	Engh & Huber (2001)
Ideal geometry (DNA, RNA)	:	Parkinson et al. (1996)
Validation Pipeline (wwPDB-VP)	:	2.35

For Manuscript Review

1 Overall quality at a glance (i)

The following experimental techniques were used to determine the structure:
X-RAY DIFFRACTION

The reported resolution of this entry is 3.49 Å.

Percentile scores (ranging between 0-100) for global validation metrics of the entry are shown in the following graphic. The table shows the number of entries on which the scores are based.

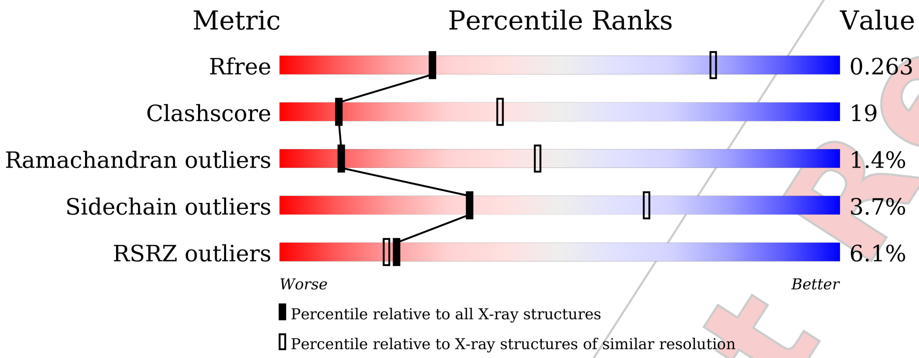

Metric	Whole archive (#Entries)	Similar resolution (#Entries, resolution range(Å))
R_{free}	130704	1659 (3.60-3.40)
Clashscore	141614	1036 (3.58-3.42)
Ramachandran outliers	138981	1005 (3.58-3.42)
Sidechain outliers	138945	1006 (3.58-3.42)
RSRZ outliers	127900	1559 (3.60-3.40)

The table below summarises the geometric issues observed across the polymeric chains and their fit to the electron density. The red, orange, yellow and green segments of the lower bar indicate the fraction of residues that contain outliers for >=3, 2, 1 and 0 types of geometric quality criteria respectively. A grey segment represents the fraction of residues that are not modelled. The numeric value for each fraction is indicated below the corresponding segment, with a dot representing fractions <=5% The upper red bar (where present) indicates the fraction of residues that have poor fit to the electron density. The numeric value is given above the bar.

Mol	Chain	Length	Quality of chain
1	A	775	51% 35% • 11%
1	B	775	5%<br>54% 31% • 14%
1	C	775	9%<br>51% 30% • 16%
1	D	775	8%<br>54% 33% • 11%
1	E	775	57% 30% • 11%
1	F	775	9%<br>52% 33% • 13%

For Manuscript Review

2 Entry composition (i)

There is only 1 type of molecule in this entry. The entry contains 32467 atoms, of which 0 are hydrogens and 0 are deuteriums.

In the tables below, the ZeroOcc column contains the number of atoms modelled with zero occupancy, the AltConf column contains the number of residues with at least one atom in alternate conformation and the Trace column contains the number of residues modelled with at most 2 atoms.

- Molecule 1 is a protein called Syn-copalyl diphosphate synthase, chloroplastic.

Mol	Chain	Residues	Atoms					ZeroOcc	AltConf	Trace
1	B	668	Total 5341	C 3391	N 916	O 1000	S 34	0	0	0
1	C	648	Total 5186	C 3297	N 892	O 962	S 35	0	1	0
1	E	688	Total 5504	C 3492	N 944	O 1033	S 35	0	0	0
1	D	688	Total 5504	C 3492	N 944	O 1033	S 35	0	0	0
1	F	677	Total 5421	C 3439	N 928	O 1019	S 35	0	0	0
1	A	689	Total 5511	C 3496	N 945	O 1035	S 35	0	0	0

There are 48 discrepancies between the modelled and reference sequences:

Chain	Residue	Modelled	Actual	Comment	Reference
B	768	GLU	-	expression tag	UNP Q0JF02
B	769	PHE	-	expression tag	UNP Q0JF02
B	770	HIS	-	expression tag	UNP Q0JF02
B	771	HIS	-	expression tag	UNP Q0JF02
B	772	HIS	-	expression tag	UNP Q0JF02
B	773	HIS	-	expression tag	UNP Q0JF02
B	774	HIS	-	expression tag	UNP Q0JF02
B	775	HIS	-	expression tag	UNP Q0JF02
C	768	GLU	-	expression tag	UNP Q0JF02
C	769	PHE	-	expression tag	UNP Q0JF02
C	770	HIS	-	expression tag	UNP Q0JF02
C	771	HIS	-	expression tag	UNP Q0JF02
C	772	HIS	-	expression tag	UNP Q0JF02
C	773	HIS	-	expression tag	UNP Q0JF02
C	774	HIS	-	expression tag	UNP Q0JF02
C	775	HIS	-	expression tag	UNP Q0JF02
E	768	GLU	-	expression tag	UNP Q0JF02

Continued on next page...

For Manuscript Review

Continued from previous page...

Chain	Residue	Modelled	Actual	Comment	Reference
E	769	PHE	-	expression tag	UNP Q0JF02
E	770	HIS	-	expression tag	UNP Q0JF02
E	771	HIS	-	expression tag	UNP Q0JF02
E	772	HIS	-	expression tag	UNP Q0JF02
E	773	HIS	-	expression tag	UNP Q0JF02
E	774	HIS	-	expression tag	UNP Q0JF02
E	775	HIS	-	expression tag	UNP Q0JF02
D	768	GLU	-	expression tag	UNP Q0JF02
D	769	PHE	-	expression tag	UNP Q0JF02
D	770	HIS	-	expression tag	UNP Q0JF02
D	771	HIS	-	expression tag	UNP Q0JF02
D	772	HIS	-	expression tag	UNP Q0JF02
D	773	HIS	-	expression tag	UNP Q0JF02
D	774	HIS	-	expression tag	UNP Q0JF02
D	775	HIS	-	expression tag	UNP Q0JF02
F	768	GLU	-	expression tag	UNP Q0JF02
F	769	PHE	-	expression tag	UNP Q0JF02
F	770	HIS	-	expression tag	UNP Q0JF02
F	771	HIS	-	expression tag	UNP Q0JF02
F	772	HIS	-	expression tag	UNP Q0JF02
F	773	HIS	-	expression tag	UNP Q0JF02
F	774	HIS	-	expression tag	UNP Q0JF02
F	775	HIS	-	expression tag	UNP Q0JF02
A	768	GLU	-	expression tag	UNP Q0JF02
A	769	PHE	-	expression tag	UNP Q0JF02
A	770	HIS	-	expression tag	UNP Q0JF02
A	771	HIS	-	expression tag	UNP Q0JF02
A	772	HIS	-	expression tag	UNP Q0JF02
A	773	HIS	-	expression tag	UNP Q0JF02
A	774	HIS	-	expression tag	UNP Q0JF02
A	775	HIS	-	expression tag	UNP Q0JF02

For Manuscript Review

3 Residue-property plots (i)

These plots are drawn for all protein, RNA, DNA and oligosaccharide chains in the entry. The first graphic for a chain summarises the proportions of the various outlier classes displayed in the second graphic. The second graphic shows the sequence view annotated by issues in geometry and electron density. Residues are color-coded according to the number of geometric quality criteria for which they contain at least one outlier: green = 0, yellow = 1, orange = 2 and red = 3 or more. A red dot above a residue indicates a poor fit to the electron density (RSRZ > 2). Stretches of 2 or more consecutive residues without any outlier are shown as a green connector. Residues present in the sample, but not in the model, are shown in grey.

- Molecule 1: Syn-copalyl diphosphate synthase, chloroplastic

Chain B:

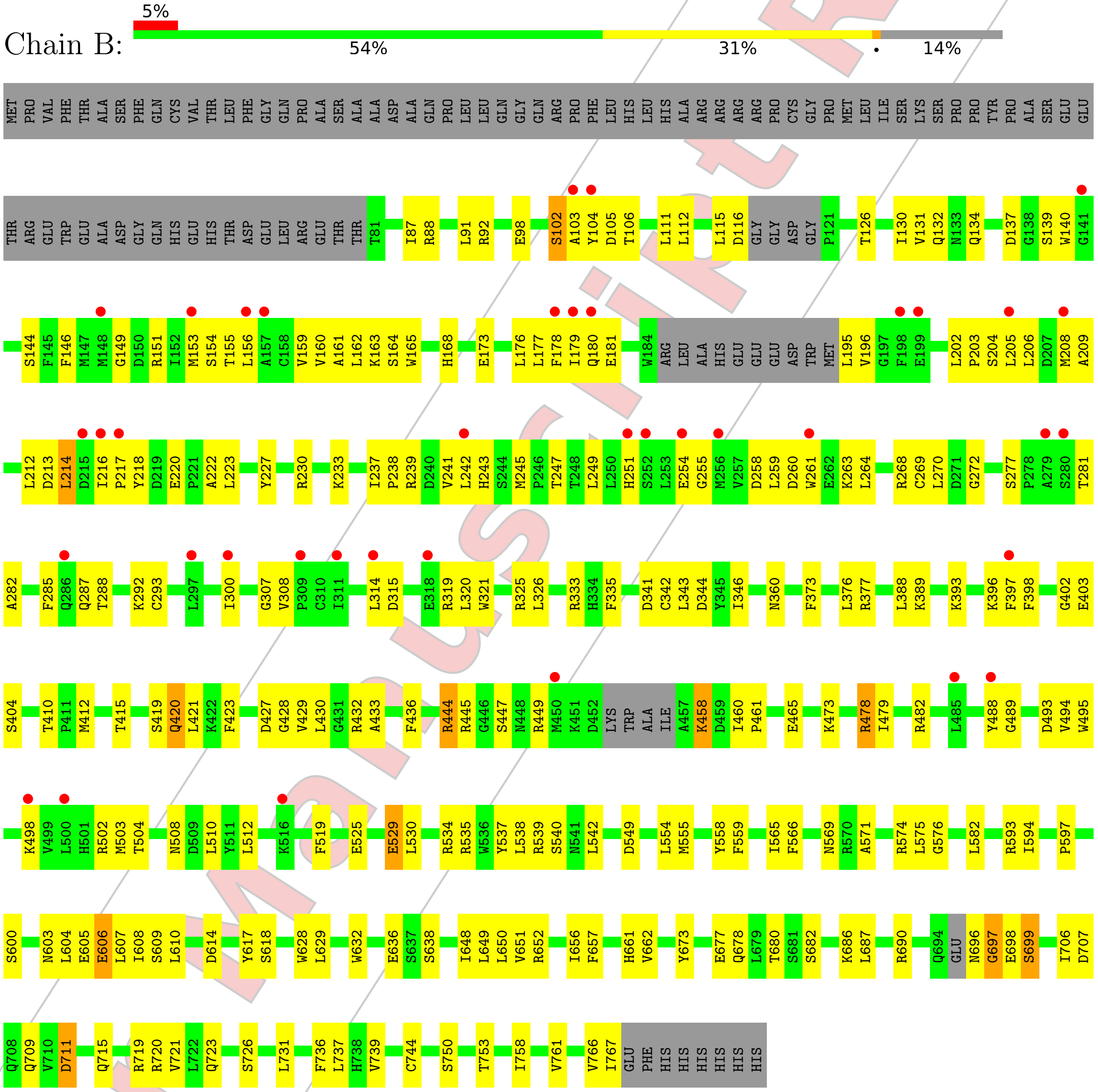

- Molecule 1: Syn-copalyl diphosphate synthase, chloroplastic

Chain C:

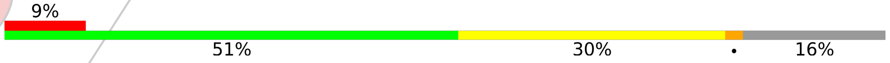

For Manuscript Review

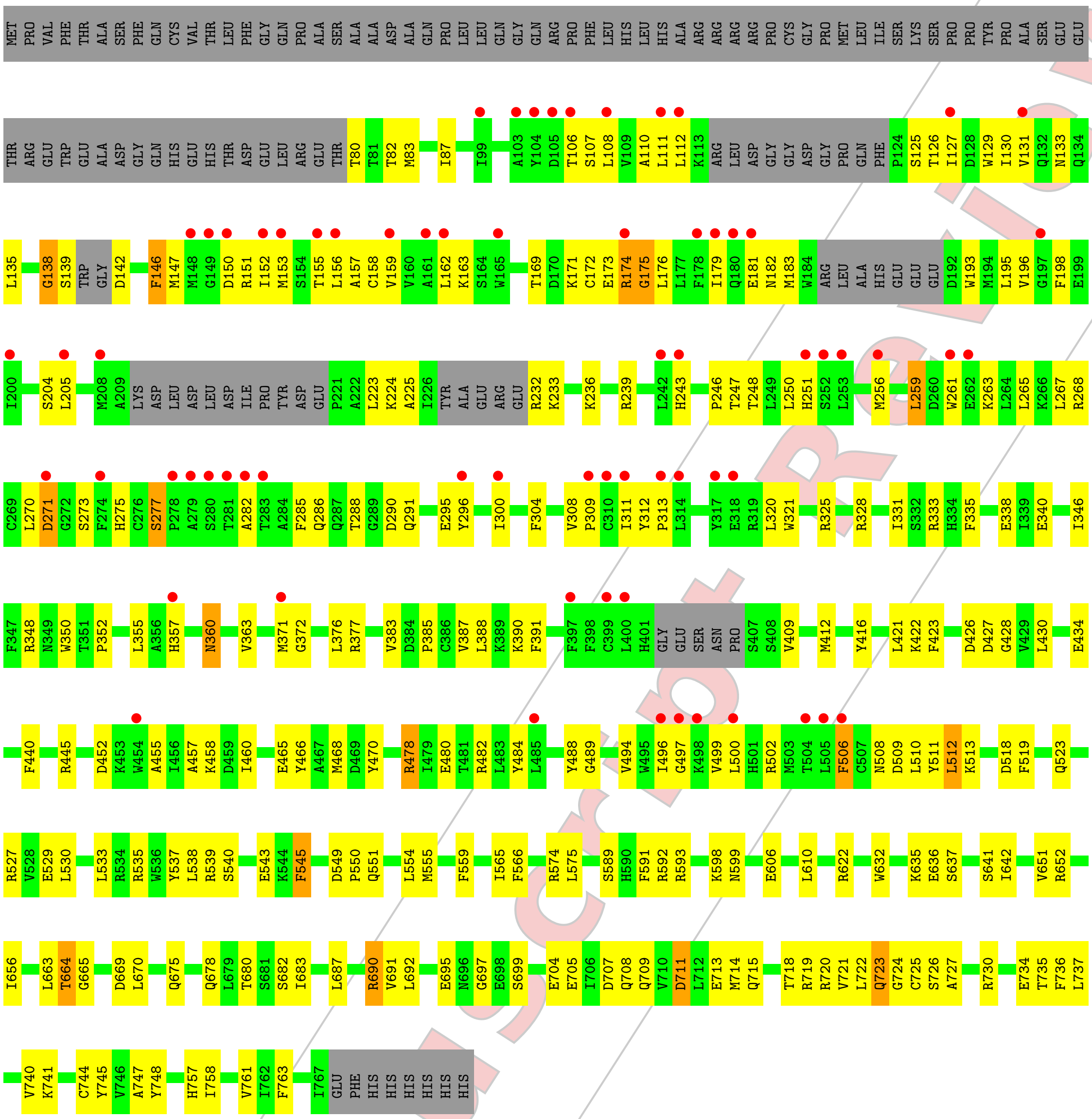

• Molecule 1: Syn-copalyl diphosphate synthase, chloroplastic

Chain E:

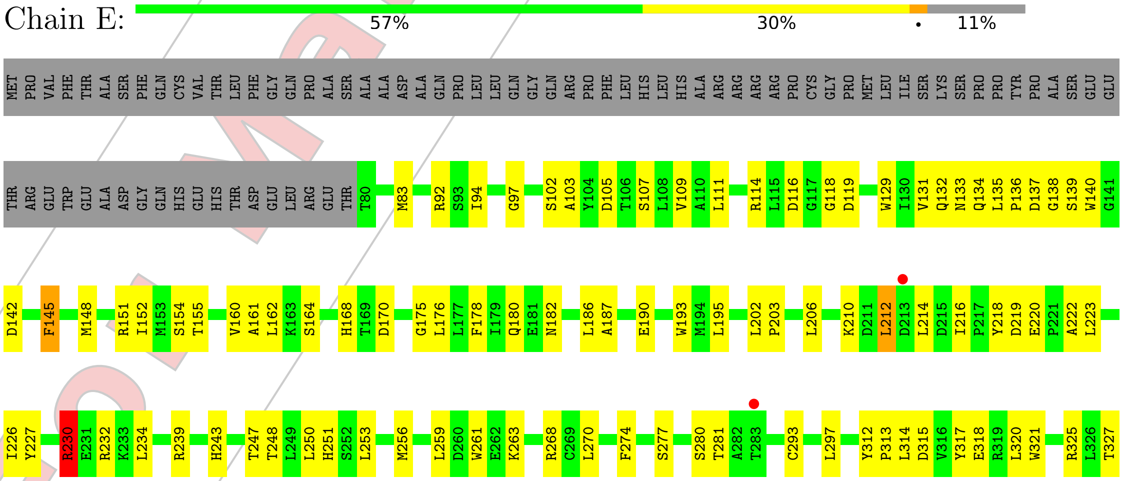

For Manuscript Review

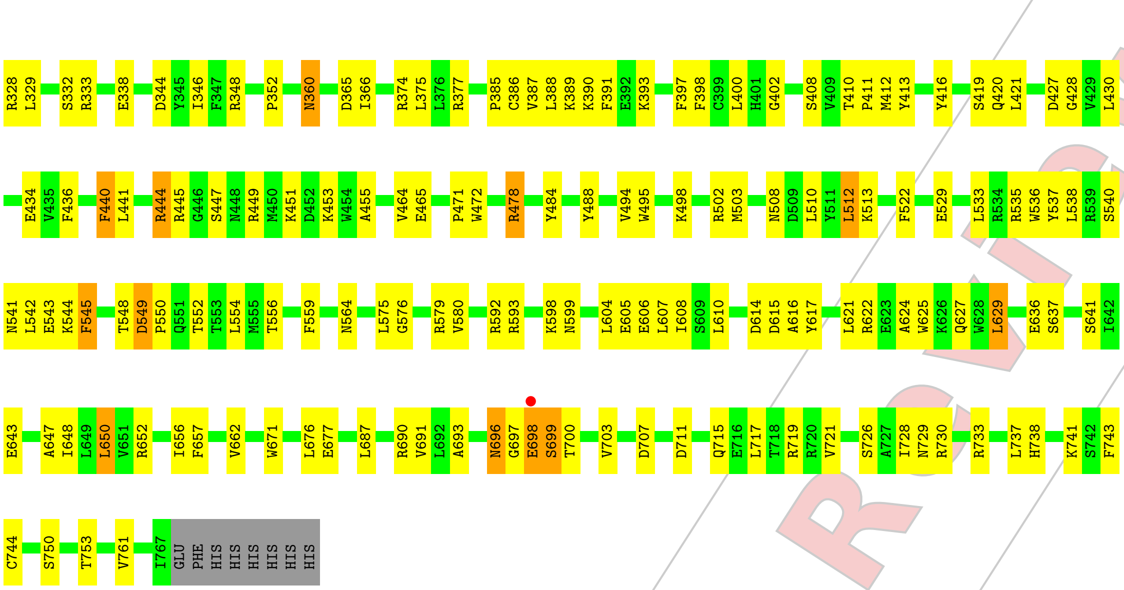

• Molecule 1: Syn-copalyl diphosphate synthase, chloroplastic

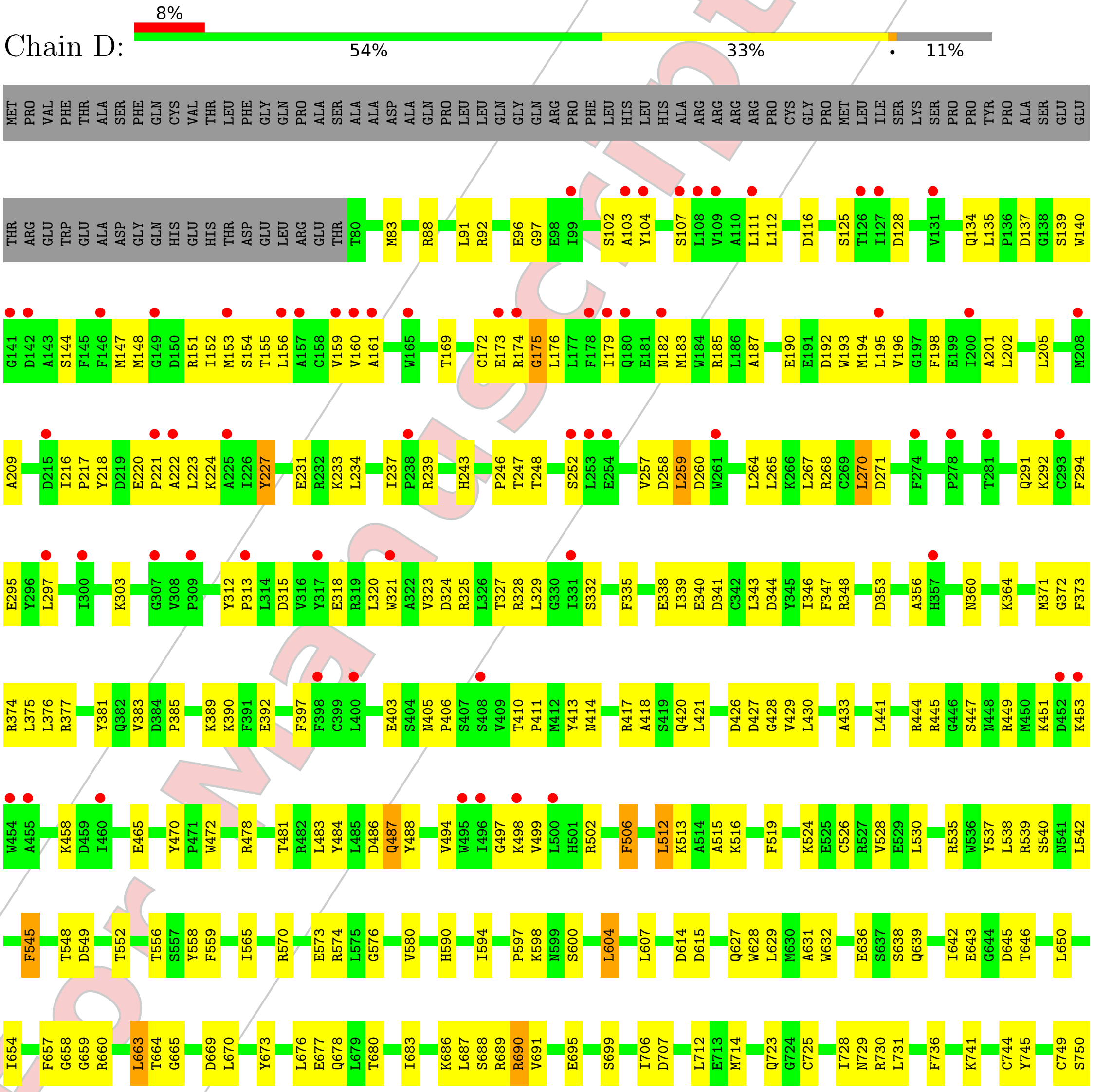

For Manuscript Review

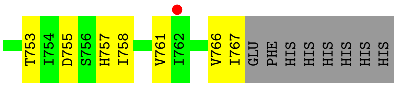

• Molecule 1: Syn-copalyl diphosphate synthase, chloroplastic

Chain F:

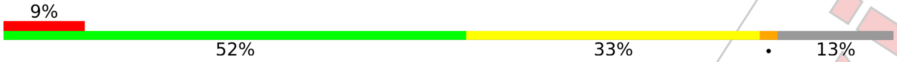

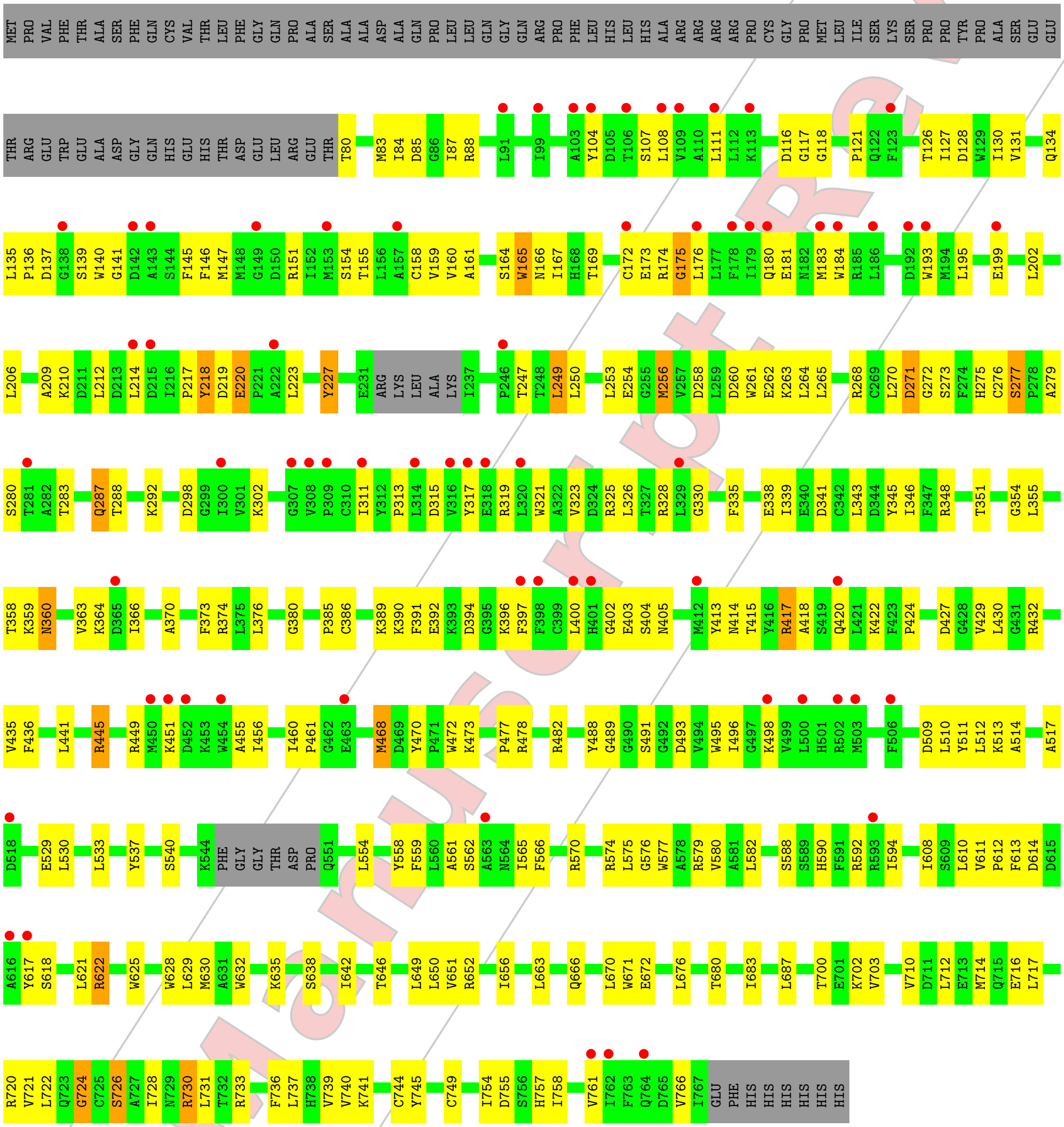

• Molecule 1: Syn-copalyl diphosphate synthase, chloroplastic

Chain A:

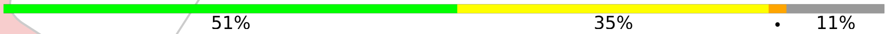

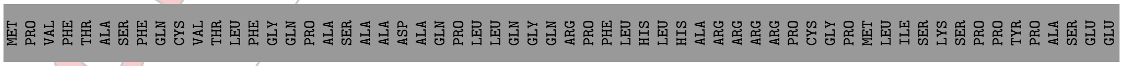

For Manuscript Review

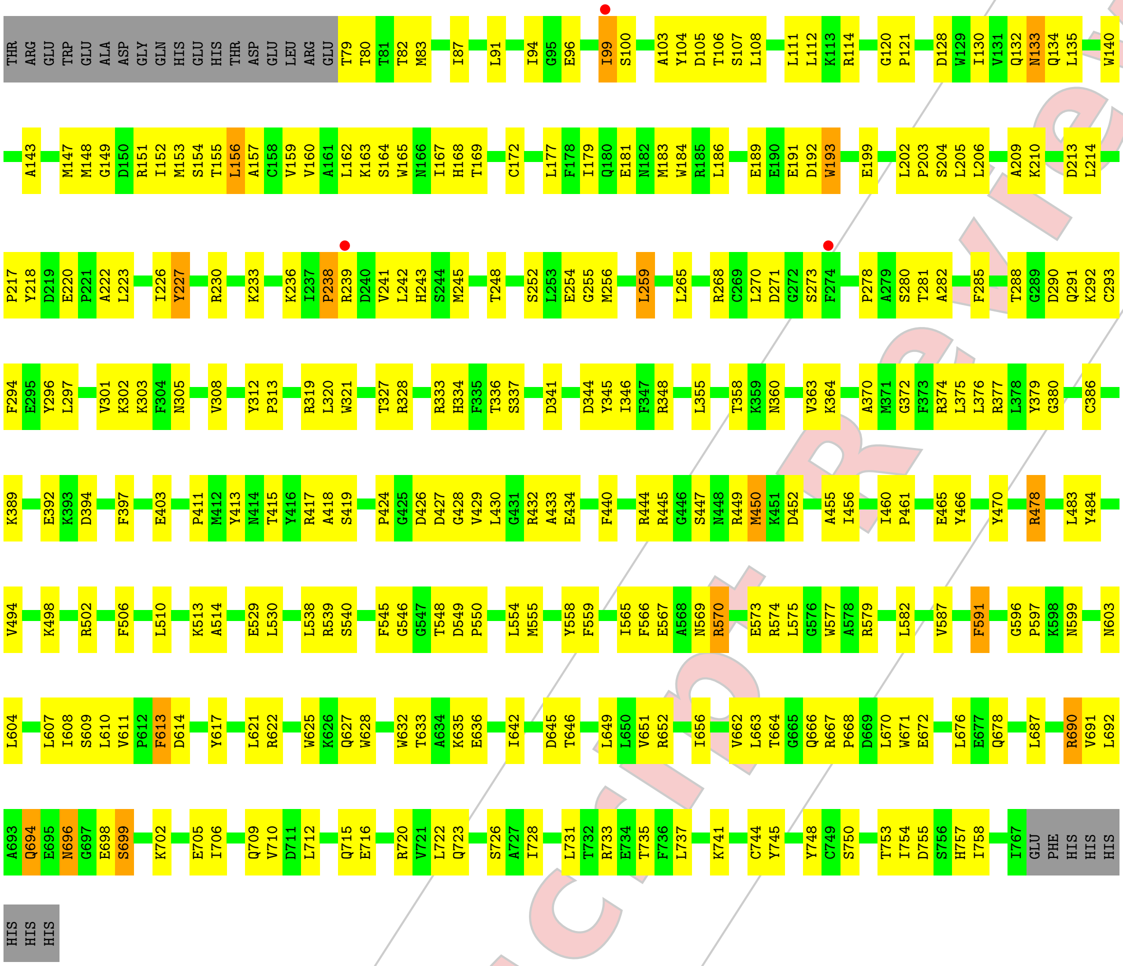

For Manuscript Review

4 Data and refinement statistics

Property	Value	Source
Space group	P 21 21 21	Depositor
Cell constants<br>a, b, c, α, β, γ	129.70Å 174.34Å 296.55Å<br>90.00° 90.00° 90.00°	Depositor
Resolution (Å)	97.62 – 3.49<br>98.19 – 3.49	Depositor<br>EDS
% Data completeness<br>(in resolution range)	96.5 (97.62-3.49)<br>95.4 (98.19-3.49)	Depositor<br>EDS
R_{merge}	(Not available)	Depositor
R_{sym}	(Not available)	Depositor
$< I/\sigma(I) >$ [1]	1.63 (at 3.49Å)	Xtriage
Refinement program	PHENIX 1.17.1_3660	Depositor
R, R_{free}	0.201 , 0.263<br>0.201 , 0.263	Depositor<br>DCC
R_{free} test set	2000 reflections (2.33%)	wwPDB-VP
Wilson B-factor (Å^2)	86.5	Xtriage
Anisotropy	0.366	Xtriage
Bulk solvent k_{sol}(e/Å^3), B_{sol}(Å^2)	0.31 , 56.8	EDS
L-test for twinning[2]	$< \|L\| > = 0.47$, $< L^2 > = 0.30$	Xtriage
Estimated twinning fraction	No twinning to report.	Xtriage
F_o,F_c correlation	0.92	EDS
Total number of atoms	32467	wwPDB-VP
Average B, all atoms (Å^2)	81.0	wwPDB-VP

Xtriage's analysis on translational NCS is as follows: *The largest off-origin peak in the Patterson function is 2.38% of the height of the origin peak. No significant pseudotranslation is detected.*

[1] Intensities estimated from amplitudes.

[2] Theoretical values of $< |L| >$, $< L^2 >$ for acentric reflections are 0.5, 0.333 respectively for untwinned datasets, and 0.375, 0.2 for perfectly twinned datasets.

For Manuscript Review

5 Model quality (i)

5.1 Standard geometry (i)

The Z score for a bond length (or angle) is the number of standard deviations the observed value is removed from the expected value. A bond length (or angle) with $|Z| > 5$ is considered an outlier worth inspection. RMSZ is the root-mean-square of all Z scores of the bond lengths (or angles).

Mol	Chain	Bond lengths		Bond angles	
		RMSZ	#|Z| >5	RMSZ	#|Z| >5
1	A	0.63	0/5634	0.84	4/7625 (0.1%)
1	B	0.54	0/5455	0.74	1/7375 (0.0%)
1	C	0.54	0/5297	0.75	1/7155 (0.0%)
1	D	0.51	0/5627	0.70	1/7615 (0.0%)
1	E	0.57	2/5627 (0.0%)	0.77	4/7615 (0.1%)
1	F	0.49	0/5540	0.75	3/7496 (0.0%)
All	All	0.55	2/33180 (0.0%)	0.76	14/44881 (0.0%)

All (2) bond length outliers are listed below:

Mol	Chain	Res	Type	Atoms	Z	Observed(Å)	Ideal(Å)
1	E	744	CYS	CB-SG	-6.91	1.70	1.82
1	E	698	GLU	CB-CG	5.77	1.63	1.52

All (14) bond angle outliers are listed below:

Mol	Chain	Res	Type	Atoms	Z	Observed(^{o})	Ideal(^{o})
1	A	177	LEU	CA-CB-CG	6.72	130.76	115.30
1	E	234	LEU	CA-CB-CG	6.52	130.29	115.30
1	F	249	LEU	CA-CB-CG	6.07	129.27	115.30
1	E	629	LEU	CA-CB-CG	-6.05	101.38	115.30
1	F	530	LEU	CA-CB-CG	5.90	128.87	115.30
1	E	650	LEU	CA-CB-CG	-5.83	101.89	115.30
1	E	212	LEU	CA-CB-CG	5.70	128.41	115.30
1	F	575	LEU	CA-CB-CG	5.70	128.40	115.30
1	A	570	ARG	NE-CZ-NH2	-5.51	117.55	120.30
1	D	270	LEU	CA-CB-CG	5.34	127.58	115.30
1	A	156	LEU	CB-CG-CD1	-5.17	102.21	111.00
1	A	649	LEU	CA-CB-CG	5.14	127.12	115.30
1	C	635	LYS	CD-CE-NZ	5.06	123.34	111.70
1	B	388	LEU	CB-CG-CD2	-5.03	102.44	111.00

There are no chirality outliers.

For Manuscript Review

There are no planarity outliers.

5.2 Too-close contacts (i)

In the following table, the Non-H and H(model) columns list the number of non-hydrogen atoms and hydrogen atoms in the chain respectively. The H(added) column lists the number of hydrogen atoms added and optimized by MolProbity. The Clashes column lists the number of clashes within the asymmetric unit, whereas Symm-Clashes lists symmetry-related clashes.

Mol	Chain	Non-H	H(model)	H(added)	Clashes	Symm-Clashes
1	A	5511	0	5420	228	0
1	B	5341	0	5268	196	0
1	C	5186	0	5140	199	0
1	D	5504	0	5413	199	0
1	E	5504	0	5413	181	0
1	F	5421	0	5323	224	0
All	All	32467	0	31977	1193	0

The all-atom clashscore is defined as the number of clashes found per 1000 atoms (including hydrogen atoms). The all-atom clashscore for this structure is 19.

All (1193) close contacts within the same asymmetric unit are listed below, sorted by their clash magnitude.

Atom-1	Atom-2	Interatomic distance (Å)	Clash overlap (Å)
1:E:608:ILE:HD11	1:E:621:LEU:HD22	1.34	1.08
1:A:156:LEU:HD11	1:A:205:LEU:HG	1.38	1.04
1:F:460:ILE:HG23	1:F:461:PRO:HD3	1.49	0.94
1:B:606:GLU:O	1:B:608:ILE:N	2.01	0.92
1:B:156:LEU:HD11	1:B:205:LEU:HG	1.52	0.91
1:F:270:LEU:HB2	1:F:360:ASN:HB2	1.51	0.91
1:A:690:ARG:HB3	1:A:706:ILE:HG21	1.53	0.91
1:E:212:LEU:HD12	1:E:214:LEU:HD23	1.53	0.90
1:F:608:ILE:HD11	1:F:622:ARG:HA	1.53	0.90
1:A:567:GLU:HB2	1:A:570:ARG:HG3	1.56	0.88
1:C:110:ALA:HB2	1:C:127:ILE:HD11	1.55	0.87
1:E:103:ALA:HB3	1:E:154:SER:HB2	1.55	0.87
1:F:140:TRP:HD1	1:F:155:THR:HA	1.38	0.86
1:B:261:TRP:NE1	1:B:287:GLN:HG2	1.91	0.85
1:D:628:TRP:HE1	1:D:646:THR:HG22	1.42	0.85
1:C:445:ARG:HE	1:C:468:MET:HE1	1.41	0.85
1:A:651:VAL:HG23	1:A:735:THR:HG22	1.58	0.84
1:B:261:TRP:HE1	1:B:287:GLN:HG2	1.42	0.83

Continued on next page...

For Manuscript Review

Continued from previous page...

Atom-1	Atom-2	Interatomic distance (Å)	Clash overlap (Å)
1:E:140:TRP:HD1	1:E:155:THR:HG23	1.44	0.83
1:B:494:VAL:HG22	1:B:502:ARG:HE	1.44	0.80
1:C:243:HIS:HB3	1:C:263:LYS:HG3	1.62	0.80
1:E:606:GLU:OE1	1:E:622:ARG:NH2	2.14	0.80
1:C:416:TYR:HE1	1:C:434:GLU:HG3	1.47	0.80
1:F:716:GLU:OE1	1:F:720:ARG:NH1	2.15	0.80
1:C:545:PHE:HB3	1:C:593:ARG:HH12	1.45	0.80
1:B:559:PHE:HE1	1:B:761:VAL:HG11	1.47	0.79
1:B:696:ASN:O	1:B:698:GLU:N	2.15	0.79
1:A:597:PRO:HG3	1:A:633:THR:HG22	1.62	0.79
1:F:488:TYR:HD2	1:F:512:LEU:HG	1.48	0.79
1:A:389:LYS:HB3	1:A:429:VAL:HG11	1.65	0.79
1:E:134:GLN:HG2	1:E:140:TRP:CZ2	2.18	0.79
1:F:183:MET:HG3	1:F:220:GLU:HG2	1.63	0.78
1:C:466:TYR:HE1	1:C:470:TYR:HB2	1.47	0.77
1:F:330:GLY:HA3	1:F:478:ARG:HH12	1.50	0.77
1:E:248:THR:HG21	1:E:400:LEU:HD22	1.65	0.77
1:F:700:THR:HA	1:F:703:VAL:HG12	1.67	0.77
1:A:104:TYR:OH	1:A:254:GLU:HG3	1.85	0.77
1:B:270:LEU:HB3	1:B:360:ASN:HB2	1.65	0.77
1:A:750:SER:HB2	1:A:753:THR:HG23	1.66	0.76
1:A:597:PRO:HD2	1:A:636:GLU:OE2	1.84	0.76
1:B:614:ASP:HB3	1:B:617:TYR:HB2	1.67	0.76
1:B:429:VAL:HG12	1:B:432:ARG:NH2	2.01	0.76
1:F:261:TRP:HE1	1:F:287:GLN:HG2	1.51	0.76
1:E:540:SER:HB3	1:E:610:LEU:HD22	1.67	0.76
1:D:559:PHE:HE1	1:D:761:VAL:HG11	1.49	0.75
1:B:203:PRO:HG3	1:B:230:ARG:HG3	1.69	0.75
1:E:270:LEU:HB2	1:E:360:ASN:HB2	1.68	0.75
1:B:429:VAL:HG12	1:B:432:ARG:HH21	1.50	0.75
1:B:687:LEU:HD21	1:B:744:CYS:HA	1.69	0.74
1:B:540:SER:HB3	1:B:610:LEU:HD22	1.68	0.74
1:C:169:THR:HA	1:C:172:CYS:HB2	1.69	0.74
1:C:270:LEU:HB2	1:C:360:ASN:HB2	1.67	0.74
1:F:628:TRP:HE1	1:F:646:THR:HG22	1.49	0.74
1:E:243:HIS:HB3	1:E:263:LYS:HG3	1.70	0.74
1:B:445:ARG:HH11	1:B:465:GLU:HG3	1.51	0.74
1:A:716:GLU:OE2	1:A:720:ARG:NH1	2.20	0.73
1:E:542:LEU:HD11	1:E:610:LEU:HD13	1.68	0.73
1:B:220:GLU:HB3	1:B:223:LEU:HB2	1.71	0.73
1:C:466:TYR:CE1	1:C:470:TYR:HB2	2.24	0.73

Continued on next page...

For Manuscript Review

Continued from previous page...

Atom-1	Atom-2	Interatomic distance (Å)	Clash overlap (Å)
1:E:648:ILE:HG12	1:E:677:GLU:HG2	1.69	0.73
1:C:250:LEU:HD21	1:C:267:LEU:HB3	1.69	0.73
1:A:529:GLU:HB3	1:A:575:LEU:HD21	1.69	0.73
1:D:140:TRP:CD1	1:D:155:THR:HA	2.23	0.73
1:E:317:TYR:HE2	1:E:503:MET:HE3	1.53	0.73
1:F:683:ILE:HD13	1:F:714:MET:HE2	1.70	0.73
1:E:129:TRP:O	1:E:133:ASN:ND2	2.19	0.72
1:D:196:VAL:HG22	1:D:497:GLY:C	2.08	0.72
1:F:396:LYS:HA	1:F:436:PHE:HE2	1.52	0.72
1:F:140:TRP:CD1	1:F:155:THR:HA	2.22	0.71
1:E:92:ARG:NH2	1:E:338:GLU:OE1	2.23	0.71
1:D:576:GLY:O	1:D:580:VAL:HG12	1.89	0.71
1:D:488:TYR:HD2	1:D:512:LEU:HG	1.54	0.71
1:C:135:LEU:HD21	1:C:139:SER:HB2	1.72	0.71
1:E:393:LYS:HB2	1:E:398:PHE:HE2	1.55	0.71
1:A:236:LYS:O	1:A:238:PRO:HD3	1.90	0.71
1:C:704:GLU:HA	1:C:707:ASP:HB2	1.72	0.71
1:D:103:ALA:HB3	1:D:154:SER:HB2	1.72	0.70
1:B:593:ARG:HB2	1:B:593:ARG:CZ	2.21	0.70
1:E:698:GLU:HG2	1:E:699:SER:H	1.56	0.70
1:D:628:TRP:NE1	1:D:646:THR:HG22	2.07	0.70
1:F:608:ILE:CD1	1:F:622:ARG:HA	2.22	0.69
1:C:540:SER:HB2	1:C:610:LEU:HD22	1.74	0.69
1:E:140:TRP:CD1	1:E:155:THR:HG23	2.28	0.69
1:D:565:ILE:HD11	1:D:570:ARG:HD3	1.75	0.69
1:F:389:LYS:HB3	1:F:429:VAL:HG11	1.75	0.69
1:D:194:MET:HG3	1:D:198:PHE:HD2	1.58	0.69
1:A:591:PHE:HE1	1:A:597:PRO:N	1.91	0.68
1:B:396:LYS:HA	1:B:436:PHE:CE2	2.28	0.68
1:C:108:LEU:HD23	1:C:282:ALA:HB3	1.76	0.68
1:D:140:TRP:HD1	1:D:155:THR:HG22	1.59	0.68
1:C:348:ARG:NH1	1:E:543:GLU:OE1	2.26	0.68
1:C:147:MET:O	1:C:151:ARG:HG2	1.94	0.68
1:C:664:THR:HG22	1:D:665:GLY:H	1.57	0.68
1:A:445:ARG:NH1	1:A:465:GLU:OE1	2.25	0.68
1:E:445:ARG:NH2	1:E:465:GLU:OE1	2.26	0.68
1:B:540:SER:HB2	1:B:542:LEU:HB2	1.75	0.68
1:D:231:GLU:HA	1:D:234:LEU:HG	1.76	0.68
1:D:683:ILE:HD13	1:D:714:MET:HE2	1.76	0.67
1:D:346:ILE:HG22	1:D:376:LEU:HD11	1.75	0.67
1:D:321:TRP:HB3	1:D:506:PHE:CZ	2.30	0.67

Continued on next page...

For Manuscript Review

Continued from previous page...

Atom-1	Atom-2	Interatomic distance (Å)	Clash overlap (Å)
1:B:389:LYS:HB3	1:B:429:VAL:HG11	1.77	0.67
1:C:636:GLU:HG2	1:C:637:SER:H	1.58	0.67
1:B:680:THR:HG21	1:B:736:PHE:HD1	1.60	0.67
1:D:104:TYR:CD2	1:D:153:MET:HG2	2.30	0.67
1:E:719:ARG:NH2	1:F:630:MET:SD	2.68	0.67
1:B:696:ASN:OD1	1:B:697:GLY:N	2.27	0.66
1:F:268:ARG:HB2	1:F:273:SER:O	1.96	0.66
1:A:456:ILE:HD11	1:A:494:VAL:HB	1.78	0.66
1:F:385:PRO:HB2	1:F:430:LEU:HD13	1.77	0.66
1:D:169:THR:HA	1:D:172:CYS:HB2	1.77	0.66
1:F:651:VAL:HG12	1:F:739:VAL:HG21	1.76	0.66
1:F:351:THR:OG1	1:F:354:GLY:O	2.12	0.66
1:F:160:VAL:O	1:F:164:SER:HB2	1.97	0.65
1:A:130:ILE:O	1:A:140:TRP:HZ3	1.80	0.65
1:D:690:ARG:NH2	1:D:707:ASP:OD1	2.29	0.65
1:F:176:LEU:O	1:F:180:GLN:HG3	1.96	0.65
1:F:757:HIS:O	1:F:761:VAL:HG12	1.96	0.65
1:A:728:ILE:O	1:A:733:ARG:NH1	2.30	0.65
1:B:530:LEU:HD13	1:B:534:ARG:HH22	1.62	0.65
1:E:203:PRO:HB2	1:E:230:ARG:HD2	1.78	0.65
1:D:257:VAL:HG22	1:D:258:ASP:H	1.61	0.65
1:A:107:SER:O	1:A:111:LEU:HD22	1.96	0.65
1:D:413:TYR:CZ	1:D:417:ARG:HD2	2.31	0.65
1:D:321:TRP:HB3	1:D:506:PHE:CE1	2.31	0.65
1:B:195:LEU:HD13	1:B:196:VAL:H	1.62	0.64
1:B:195:LEU:HD13	1:B:196:VAL:N	2.12	0.64
1:A:238:PRO:HG2	1:A:241:VAL:HB	1.78	0.64
1:C:549:ASP:O	1:C:551:GLN:N	2.31	0.64
1:E:281:THR:HG23	1:E:293:CYS:SG	2.38	0.64
1:F:540:SER:HB2	1:F:610:LEU:HD22	1.79	0.64
1:B:603:ASN:O	1:B:603:ASN:ND2	2.30	0.64
1:F:83:MET:HG2	1:F:510:LEU:HD11	1.78	0.64
1:F:676:LEU:O	1:F:680:THR:HG23	1.97	0.64
1:E:559:PHE:HE1	1:E:761:VAL:HG11	1.63	0.64
1:E:408:SER:HB2	1:E:411:PRO:HD2	1.79	0.64
1:E:494:VAL:HG22	1:E:502:ARG:NH2	2.13	0.64
1:B:243:HIS:CD2	1:B:260:ASP:HB3	2.33	0.64
1:F:427:ASP:OD1	1:F:429:VAL:HG13	1.98	0.64
1:D:270:LEU:HB2	1:D:360:ASN:HB2	1.80	0.63
1:A:103:ALA:HB3	1:A:154:SER:HB2	1.79	0.63
1:B:116:ASP:N	1:B:116:ASP:OD1	2.31	0.63

Continued on next page...

For Manuscript Review

Continued from previous page...

Atom-1	Atom-2	Interatomic distance (Å)	Clash overlap (Å)
1:C:139:SER:HA	1:C:155:THR:HG23	1.81	0.63
1:B:554:LEU:HA	1:B:582:LEU:HD21	1.80	0.63
1:B:98:GLU:HB3	1:B:504:THR:HB	1.81	0.63
1:E:608:ILE:HD13	1:E:622:ARG:HA	1.80	0.63
1:F:721:VAL:HA	1:F:733:ARG:HG2	1.80	0.63
1:C:333:ARG:NH2	1:C:518:ASP:OD2	2.32	0.63
1:D:183:MET:HG3	1:D:220:GLU:HG3	1.80	0.63
1:D:542:LEU:HD13	1:D:542:LEU:O	1.97	0.63
1:B:393:LYS:HD2	1:B:398:PHE:CD2	2.34	0.63
1:C:340:GLU:OE2	1:E:535:ARG:NH2	2.32	0.63
1:C:181:GLU:OE2	1:C:182:ASN:ND2	2.32	0.63
1:C:664:THR:HG21	1:D:663:LEU:HD12	1.79	0.63
1:B:259:LEU:HD13	1:B:264:LEU:HD22	1.80	0.62
1:A:265:LEU:HD11	1:A:288:THR:HG21	1.81	0.62
1:D:243:HIS:CD2	1:D:260:ASP:HB3	2.33	0.62
1:E:627:GLN:OE1	1:F:720:ARG:NH2	2.32	0.62
1:D:420:GLN:HE21	1:D:420:GLN:HA	1.63	0.62
1:F:87:ILE:HD12	1:F:511:TYR:CD1	2.35	0.62
1:E:472:TRP:O	1:E:741:LYS:NZ	2.28	0.62
1:F:683:ILE:HD12	1:F:717:LEU:HD12	1.81	0.62
1:A:755:ASP:HA	1:A:758:ILE:HD12	1.81	0.62
1:B:673:TYR:CD1	1:A:670:LEU:HD21	2.34	0.62
1:A:411:PRO:O	1:A:415:THR:HG23	1.99	0.62
1:F:449:ARG:HA	1:F:451:LYS:HE2	1.82	0.62
1:A:604:LEU:HD22	1:A:607:LEU:HD22	1.80	0.62
1:E:145:PHE:HD2	1:E:494:VAL:HG21	1.64	0.62
1:B:243:HIS:HD2	1:B:260:ASP:HB3	1.64	0.62
1:A:608:ILE:HD11	1:A:625:TRP:CD1	2.35	0.62
1:A:270:LEU:HB2	1:A:360:ASN:HB2	1.82	0.61
1:A:204:SER:HB2	1:A:255:GLY:HA3	1.82	0.61
1:A:613:PHE:CE1	1:A:617:TYR:HB3	2.35	0.61
1:C:138:GLY:O	1:C:139:SER:OG	2.18	0.61
1:E:176:LEU:O	1:E:180:GLN:HG3	2.00	0.61
1:B:103:ALA:HB3	1:B:154:SER:HB2	1.81	0.61
1:B:242:LEU:HD12	1:B:249:LEU:HD23	1.80	0.61
1:B:473:LYS:NZ	1:B:711:ASP:OD2	2.23	0.61
1:C:107:SER:O	1:C:110:ALA:N	2.32	0.61
1:C:335:PHE:HB3	1:C:338:GLU:HB2	1.81	0.61
1:A:149:GLY:N	1:A:189:GLU:OE2	2.25	0.61
1:E:137:ASP:OD2	1:E:151:ARG:NH1	2.34	0.61
1:F:730:ARG:HG2	1:F:733:ARG:HH22	1.64	0.61

Continued on next page...

For Manuscript Review

Continued from previous page...

Atom-1	Atom-2	Interatomic distance (Å)	Clash overlap (Å)
1:B:576:GLY:HA3	1:B:657:PHE:CE2	2.37	0.60
1:E:576:GLY:O	1:E:580:VAL:HG12	2.00	0.60
1:F:374:ARG:HG3	1:F:418:ALA:HA	1.83	0.60
1:B:204:SER:HB2	1:B:255:GLY:HA2	1.83	0.60
1:D:494:VAL:HG13	1:D:502:ARG:HG2	1.83	0.60
1:C:680:THR:HG21	1:C:736:PHE:HD1	1.65	0.60
1:F:611:VAL:HB	1:F:612:PRO:HD2	1.84	0.60
1:B:137:ASP:OD2	1:B:137:ASP:N	2.31	0.60
1:C:320:LEU:HD13	1:C:371:MET:HB3	1.83	0.60
1:C:458:LYS:HD3	1:C:489:GLY:HA2	1.83	0.60
1:C:535:ARG:HE	1:C:539[A]:ARG:NH1	1.99	0.60
1:E:698:GLU:CG	1:E:699:SER:H	2.14	0.60
1:F:628:TRP:NE1	1:F:646:THR:HG22	2.17	0.60
1:C:416:TYR:CE1	1:C:434:GLU:HG3	2.34	0.60
1:C:636:GLU:HG2	1:C:637:SER:N	2.16	0.60
1:F:728:ILE:HD11	1:F:733:ARG:HD3	1.84	0.60
1:A:140:TRP:CD1	1:A:155:THR:HA	2.37	0.60
1:E:320:LEU:HD21	1:E:346:ILE:HG12	1.82	0.60
1:C:445:ARG:NE	1:C:468:MET:HE1	2.15	0.59
1:C:535:ARG:HE	1:C:539[A]:ARG:HH12	1.49	0.59
1:F:217:PRO:O	1:F:219:ASP:N	2.35	0.59
1:F:396:LYS:HA	1:F:436:PHE:CE2	2.35	0.59
1:F:717:LEU:HD11	1:F:740:VAL:HG21	1.83	0.59
1:E:637:SER:HB3	1:F:712:LEU:HD11	1.83	0.59
1:D:628:TRP:HE1	1:D:646:THR:CG2	2.13	0.59
1:B:661:HIS:HD1	1:B:662:VAL:N	2.00	0.59
1:E:700:THR:O	1:E:703:VAL:N	2.36	0.59
1:E:537:TYR:CE2	1:E:543:GLU:HG2	2.37	0.59
1:F:374:ARG:HB2	1:F:418:ALA:HB2	1.85	0.59
1:A:663:LEU:O	1:A:664:THR:HG23	2.02	0.59
1:B:319:ARG:HD3	1:B:342:CYS:HB2	1.84	0.59
1:C:126:THR:O	1:C:130:ILE:HG13	2.02	0.59
1:E:160:VAL:O	1:E:164:SER:HB2	2.02	0.59
1:D:750:SER:O	1:D:753:THR:HG22	2.02	0.59
1:C:247:THR:H	1:C:250:LEU:HD13	1.68	0.59
1:F:223:LEU:O	1:F:227:TYR:HB2	2.02	0.59
1:A:83:MET:SD	1:A:513:LYS:HG2	2.43	0.59
1:C:321:TRP:HB3	1:C:506:PHE:CZ	2.37	0.59
1:F:121:PRO:N	1:F:165:TRP:HE1	2.00	0.59
1:B:608:ILE:HG13	1:B:609:SER:N	2.17	0.59
1:C:385:PRO:HB2	1:C:430:LEU:HD13	1.84	0.59

Continued on next page...

For Manuscript Review

Continued from previous page...

Atom-1	Atom-2	Interatomic distance (Å)	Clash overlap (Å)
1:E:552:THR:O	1:E:556:THR:HG23	2.02	0.59
1:D:524:LYS:O	1:D:528:VAL:HG23	2.02	0.59
1:F:87:ILE:HG23	1:F:511:TYR:CE1	2.38	0.59
1:B:126:THR:O	1:B:130:ILE:HG13	2.02	0.58
1:B:488:TYR:HD2	1:B:512:LEU:HG	1.67	0.58
1:A:745:TYR:OH	1:A:757:HIS:ND1	2.28	0.58
1:A:413:TYR:CZ	1:A:417:ARG:HD3	2.38	0.58
1:F:195:LEU:HD23	1:F:496:ILE:HG23	1.85	0.58
1:A:140:TRP:HD1	1:A:155:THR:HG23	1.66	0.58
1:A:687:LEU:HD21	1:A:744:CYS:HA	1.85	0.58
1:C:664:THR:OG1	1:C:665:GLY:N	2.32	0.58
1:E:728:ILE:O	1:E:733:ARG:NH1	2.37	0.58
1:A:156:LEU:C	1:A:156:LEU:HD12	2.24	0.58
1:E:533:LEU:HD22	1:E:554:LEU:CD1	2.33	0.58
1:D:565:ILE:HD11	1:D:570:ARG:CD	2.33	0.58
1:A:255:GLY:O	1:A:256:MET:HG2	2.04	0.58
1:A:591:PHE:HE1	1:A:596:GLY:C	2.07	0.58
1:C:664:THR:HB	1:D:663:LEU:HA	1.84	0.58
1:E:206:LEU:HD13	1:E:223:LEU:HD21	1.85	0.58
1:A:183:MET:HG2	1:A:217:PRO:HG2	1.85	0.58
1:E:151:ARG:O	1:E:155:THR:OG1	2.19	0.58
1:D:104:TYR:HB3	1:D:499:VAL:HG21	1.84	0.58
1:F:147:MET:O	1:F:151:ARG:HG2	2.03	0.58
1:F:441:LEU:HB2	1:F:468:MET:CE	2.33	0.58
1:F:441:LEU:O	1:F:445:ARG:HB2	2.03	0.58
1:A:130:ILE:O	1:A:140:TRP:CZ3	2.56	0.58
1:A:148:MET:HG3	1:A:186:LEU:HD22	1.85	0.58
1:B:156:LEU:HD11	1:B:205:LEU:CG	2.32	0.57
1:B:281:THR:HG23	1:B:293:CYS:SG	2.44	0.57
1:C:488:TYR:HD2	1:C:512:LEU:HG	1.68	0.57
1:E:140:TRP:CD1	1:E:155:THR:HA	2.39	0.57
1:E:508:ASN:OD1	1:E:510:LEU:N	2.37	0.57
1:D:766:VAL:O	1:D:767:ILE:HG13	2.03	0.57
1:B:447:SER:HB3	1:B:449:ARG:HG3	1.86	0.57
1:E:447:SER:OG	1:E:449:ARG:NH1	2.37	0.57
1:E:604:LEU:HA	1:E:607:LEU:HD13	1.85	0.57
1:D:216:ILE:HG13	1:D:218:TYR:CE1	2.40	0.57
1:A:140:TRP:CD1	1:A:155:THR:HG23	2.39	0.57
1:A:579:ARG:HD2	1:A:611:VAL:HG13	1.86	0.57
1:E:693:ALA:HA	1:E:696:ASN:HB2	1.85	0.57
1:A:112:LEU:HD11	1:A:282:ALA:HB1	1.85	0.57

Continued on next page...

For Manuscript Review

Continued from previous page...

Atom-1	Atom-2	Interatomic distance (Å)	Clash overlap (Å)
1:F:135:LEU:HD12	1:F:139:SER:H	1.69	0.57
1:F:253:LEU:HA	1:F:256:MET:HG2	1.85	0.57
1:E:138:GLY:O	1:E:175:GLY:HA2	2.05	0.57
1:C:722:LEU:O	1:C:724:GLY:N	2.37	0.57
1:E:397:PHE:HB2	1:E:436:PHE:CE2	2.40	0.57
1:D:259:LEU:H	1:D:259:LEU:HD23	1.68	0.57
1:F:155:THR:O	1:F:159:VAL:HG23	2.03	0.57
1:A:690:ARG:HB3	1:A:706:ILE:CG2	2.31	0.57
1:E:203:PRO:HB2	1:E:230:ARG:HH11	1.70	0.57
1:D:327:THR:HA	1:D:332:SER:HB3	1.87	0.57
1:F:429:VAL:HG12	1:F:432:ARG:HH21	1.69	0.57
1:C:175:GLY:O	1:C:179:ILE:HG13	2.05	0.57
1:E:447:SER:HB2	1:E:449:ARG:HG2	1.87	0.57
1:F:265:LEU:HD11	1:F:288:THR:HG21	1.86	0.57
1:D:134:GLN:HG3	1:D:140:TRP:CZ2	2.40	0.57
1:D:246:PRO:N	1:D:267:LEU:HD12	2.20	0.57
1:D:519:PHE:HZ	1:D:761:VAL:HG23	1.70	0.57
1:F:135:LEU:HD11	1:F:139:SER:HB2	1.84	0.57
1:A:169:THR:HA	1:A:172:CYS:HB2	1.87	0.57
1:E:187:ALA:HB2	1:E:222:ALA:HB2	1.87	0.56
1:E:385:PRO:HB2	1:E:430:LEU:HD13	1.87	0.56
1:A:220:GLU:HG3	1:A:222:ALA:H	1.70	0.56
1:C:545:PHE:HB3	1:C:593:ARG:NH1	2.17	0.56
1:C:273:SER:HB3	1:C:296:TYR:CZ	2.40	0.56
1:C:529:GLU:HB3	1:C:575:LEU:HD21	1.85	0.56
1:E:202:LEU:HD23	1:E:226:ILE:HD11	1.88	0.56
1:D:252:SER:HB3	1:D:498:LYS:HE2	1.87	0.56
1:F:413:TYR:CZ	1:F:417:ARG:HD2	2.39	0.56
1:A:321:TRP:HB3	1:A:506:PHE:CE2	2.41	0.56
1:C:376:LEU:HB3	1:C:383:VAL:HG21	1.85	0.56
1:C:155:THR:O	1:C:158:CYS:HB2	2.05	0.56
1:C:705:GLU:O	1:C:709:GLN:HG3	2.06	0.56
1:F:206:LEU:O	1:F:210:LYS:N	2.38	0.56
1:D:192:ASP:HB3	1:D:453:LYS:HG2	1.88	0.56
1:A:358:THR:HG23	1:A:360:ASN:H	1.70	0.56
1:A:570:ARG:HD2	1:A:573:GLU:OE1	2.06	0.56
1:B:269:CYS:HB3	1:B:360:ASN:O	2.06	0.56
1:B:565:ILE:O	1:B:574:ARG:HD3	2.06	0.56
1:B:597:PRO:HA	1:B:600:SER:HB3	1.87	0.56
1:A:268:ARG:NH2	1:A:290:ASP:OD2	2.39	0.56
1:B:373:PHE:HZ	1:B:377:ARG:HH21	1.54	0.56

Continued on next page...

For Manuscript Review

Continued from previous page...

Atom-1	Atom-2	Interatomic distance (Å)	Clash overlap (Å)
1:D:604:LEU:HA	1:D:607:LEU:HD13	1.88	0.56
1:F:730:ARG:HG2	1:F:733:ARG:NH2	2.21	0.56
1:A:613:PHE:CE1	1:A:617:TYR:CB	2.88	0.56
1:D:196:VAL:HG22	1:D:498:LYS:N	2.22	0.55
1:D:385:PRO:HB2	1:D:430:LEU:HD13	1.88	0.55
1:F:183:MET:HG2	1:F:217:PRO:HG2	1.87	0.55
1:B:214:LEU:HD12	1:B:214:LEU:O	2.05	0.55
1:F:127:ILE:HG21	1:F:167:ILE:HD11	1.89	0.55
1:D:392:GLU:HG3	1:D:397:PHE:CE1	2.41	0.55
1:C:259:LEU:HD12	1:C:261:TRP:CZ2	2.41	0.55
1:F:554:LEU:HA	1:F:582:LEU:HD21	1.88	0.55
1:F:614:ASP:HB3	1:F:617:TYR:CZ	2.42	0.55
1:A:424:PRO:HD3	1:A:715:GLN:HB3	1.89	0.55
1:A:690:ARG:NH2	1:A:748:TYR:HE1	2.04	0.55
1:C:321:TRP:HB3	1:C:506:PHE:CE1	2.41	0.55
1:D:182:ASN:OD1	1:D:185:ARG:NH1	2.31	0.55
1:E:203:PRO:HG2	1:E:230:ARG:HH11	1.72	0.55
1:D:590:HIS:O	1:D:594:ILE:HG12	2.07	0.55
1:F:160:VAL:HG12	1:F:212:LEU:HD13	1.89	0.55
1:B:652:ARG:O	1:B:656:ILE:HG13	2.07	0.55
1:C:275:HIS:CD2	1:C:363:VAL:HG11	2.42	0.55
1:D:597:PRO:HA	1:D:600:SER:HB3	1.88	0.54
1:F:107:SER:HB2	1:F:161:ALA:HB2	1.88	0.54
1:F:279:ALA:HB3	1:F:498:LYS:HZ2	1.73	0.54
1:F:652:ARG:O	1:F:656:ILE:HG13	2.07	0.54
1:A:621:LEU:HD12	1:A:656:ILE:HD12	1.88	0.54
1:C:530:LEU:HD21	1:C:555:MET:SD	2.47	0.54
1:B:346:ILE:HG22	1:B:376:LEU:HD11	1.90	0.54
1:E:687:LEU:O	1:E:691:VAL:HG23	2.07	0.54
1:F:181:GLU:O	1:F:184:TRP:HD1	1.91	0.54
1:C:142:ASP:HB3	1:C:500:LEU:HB2	1.90	0.54
1:F:83:MET:SD	1:F:513:LYS:HG2	2.47	0.54
1:B:251:HIS:CE1	1:B:277:SER:HB2	2.42	0.54
1:F:565:ILE:O	1:F:574:ARG:HD3	2.07	0.54
1:A:159:VAL:HG13	1:A:172:CYS:SG	2.47	0.54
1:A:285:PHE:CE1	1:A:294:PHE:HB2	2.42	0.54
1:A:613:PHE:CZ	1:A:617:TYR:HB3	2.43	0.54
1:B:103:ALA:O	1:B:106:THR:HG22	2.08	0.54
1:B:445:ARG:NH1	1:B:465:GLU:HG3	2.22	0.54
1:E:420:GLN:HE21	1:E:420:GLN:HA	1.72	0.54
1:D:680:THR:HG21	1:D:736:PHE:HD1	1.73	0.54

Continued on next page...

For Manuscript Review

Continued from previous page...

Atom-1	Atom-2	Interatomic distance (Å)	Clash overlap (Å)
1:F:135:LEU:HD13	1:F:137:ASP:HB3	1.89	0.54
1:A:565:ILE:O	1:A:574:ARG:HD3	2.08	0.54
1:A:705:GLU:O	1:A:709:GLN:HG2	2.08	0.54
1:B:104:TYR:OH	1:B:254:GLU:OE2	2.22	0.54
1:B:648:ILE:HD11	1:B:677:GLU:HB3	1.90	0.54
1:D:179:ILE:O	1:D:183:MET:HB2	2.08	0.54
1:F:588:SER:O	1:F:592:ARG:HD3	2.07	0.54
1:C:687:LEU:HD11	1:C:744:CYS:HA	1.90	0.54
1:E:134:GLN:HG2	1:E:140:TRP:CH2	2.43	0.54
1:F:169:THR:HA	1:F:172:CYS:HB2	1.89	0.54
1:A:135:LEU:HD23	1:A:140:TRP:HA	1.90	0.54
1:A:254:GLU:OE1	1:A:498:LYS:NZ	2.41	0.54
1:E:397:PHE:HB2	1:E:436:PHE:CD2	2.43	0.53
1:E:647:ALA:HB2	1:E:743:PHE:HE2	1.72	0.53
1:D:353:ASP:OD1	1:D:390:LYS:HE2	2.08	0.53
1:F:173:GLU:C	1:F:175:GLY:H	2.12	0.53
1:A:392:GLU:OE1	1:A:432:ARG:NH2	2.41	0.53
1:C:711:ASP:O	1:C:715:GLN:HG3	2.08	0.53
1:A:706:ILE:O	1:A:710:VAL:HG12	2.08	0.53
1:B:239:ARG:NH2	1:B:258:ASP:O	2.40	0.53
1:B:420:GLN:HA	1:B:420:GLN:HE21	1.73	0.53
1:D:265:LEU:O	1:D:268:ARG:NH1	2.32	0.53
1:D:723:GLN:OE1	1:D:725:CYS:HB2	2.08	0.53
1:B:605:GLU:HA	1:B:629:LEU:HD11	1.90	0.53
1:B:721:VAL:HG11	1:B:737:LEU:HB2	1.90	0.53
1:C:606:GLU:OE2	1:C:622:ARG:NH1	2.36	0.53
1:F:617:TYR:HB3	1:F:656:ILE:HG22	1.89	0.53
1:A:148:MET:HE3	1:A:152:ILE:HG12	1.90	0.53
1:B:106:THR:HA	1:B:308:VAL:HG12	1.90	0.53
1:C:111:LEU:O	1:C:112:LEU:HD23	2.09	0.53
1:C:683:ILE:HD13	1:C:714:MET:HE2	1.89	0.53
1:C:690:ARG:NH2	1:C:707:ASP:OD2	2.42	0.53
1:D:691:VAL:O	1:D:695:GLU:HG2	2.07	0.53
1:F:146:PHE:CE1	1:F:151:ARG:HG3	2.43	0.53
1:A:460:ILE:HG23	1:A:461:PRO:HD3	1.90	0.53
1:B:396:LYS:HA	1:B:436:PHE:HE2	1.70	0.53
1:C:328:ARG:NH1	1:C:484:TYR:CZ	2.77	0.53
1:D:147:MET:O	1:D:151:ARG:HG2	2.08	0.53
1:F:646:THR:O	1:F:649:LEU:HB3	2.09	0.53
1:B:134:GLN:HG3	1:B:140:TRP:CE2	2.44	0.53
1:B:204:SER:CB	1:B:255:GLY:HA2	2.39	0.53

Continued on next page...

For Manuscript Review

Continued from previous page...

Atom-1	Atom-2	Interatomic distance (Å)	Clash overlap (Å)
1:B:245:MET:O	1:B:247:THR:HG23	2.09	0.53
1:C:691:VAL:HG11	1:C:747:ALA:HA	1.90	0.53
1:D:335:PHE:HB3	1:D:338:GLU:HG2	1.91	0.53
1:D:657:PHE:C	1:D:659:GLY:H	2.12	0.53
1:F:249:LEU:HD12	1:F:250:LEU:HD12	1.90	0.53
1:F:277:SER:OG	1:F:280:SER:HB2	2.07	0.53
1:C:153:MET:HE3	1:C:198:PHE:HE1	1.73	0.53
1:C:664:THR:HG22	1:D:665:GLY:N	2.24	0.53
1:D:156:LEU:O	1:D:160:VAL:HG22	2.09	0.53
1:A:156:LEU:O	1:A:160:VAL:HG22	2.08	0.53
1:A:302:LYS:HG3	1:A:303:LYS:N	2.24	0.53
1:B:209:ALA:HA	1:B:214:LEU:HD21	1.90	0.53
1:F:386:CYS:O	1:F:389:LYS:HG2	2.08	0.53
1:F:146:PHE:HE1	1:F:151:ARG:HG3	1.74	0.53
1:F:261:TRP:NE1	1:F:287:GLN:HG2	2.21	0.53
1:F:730:ARG:HA	1:F:733:ARG:NH1	2.23	0.53
1:A:193:TRP:CZ3	1:A:456:ILE:HG22	2.43	0.53
1:A:690:ARG:O	1:A:694:GLN:N	2.42	0.53
1:C:589:SER:HA	1:C:592:ARG:HE	1.74	0.52
1:E:488:TYR:HD2	1:E:512:LEU:HG	1.73	0.52
1:E:717:LEU:O	1:E:721:VAL:HG23	2.09	0.52
1:D:673:TYR:CE1	1:D:677:GLU:HG3	2.44	0.52
1:A:134:GLN:HG3	1:A:140:TRP:CZ2	2.45	0.52
1:A:319:ARG:CD	1:A:345:TYR:HD2	2.21	0.52
1:C:745:TYR:OH	1:C:757:HIS:ND1	2.33	0.52
1:E:190:GLU:HG3	1:E:193:TRP:HE3	1.73	0.52
1:D:247:THR:OG1	1:D:248:THR:N	2.42	0.52
1:F:116:ASP:O	1:F:118:GLY:N	2.41	0.52
1:B:149:GLY:O	1:B:153:MET:HB3	2.09	0.52
1:B:651:VAL:HG12	1:B:739:VAL:HG21	1.92	0.52
1:D:650:LEU:O	1:D:654:ILE:HG13	2.09	0.52
1:F:271:ASP:OD2	1:F:313:PRO:HD3	2.09	0.52
1:A:530:LEU:HD11	1:A:555:MET:SD	2.50	0.52
1:A:587:VAL:O	1:A:591:PHE:HB2	2.08	0.52
1:A:672:GLU:OE2	1:A:726:SER:HB2	2.08	0.52
1:B:88:ARG:HG2	1:B:335:PHE:CD1	2.44	0.52
1:C:527:ARG:HH21	1:C:763:PHE:HA	1.73	0.52
1:F:84:ILE:HG23	1:F:335:PHE:HE1	1.75	0.52
1:F:265:LEU:O	1:F:268:ARG:NH1	2.43	0.52
1:B:268:ARG:HD2	1:B:272:GLY:O	2.09	0.52
1:E:608:ILE:HD12	1:E:625:TRP:CB	2.39	0.52

Continued on next page...

For Manuscript Review

Continued from previous page...

Atom-1	Atom-2	Interatomic distance (Å)	Clash overlap (Å)
1:E:726:SER:HB3	1:E:733:ARG:NH1	2.24	0.52
1:F:183:MET:CE	1:F:223:LEU:HD11	2.39	0.52
1:A:134:GLN:HG3	1:A:140:TRP:CE2	2.45	0.52
1:B:220:GLU:HG3	1:B:222:ALA:H	1.75	0.52
1:B:377:ARG:HG3	1:B:377:ARG:HH11	1.74	0.52
1:B:538:LEU:C	1:B:540:SER:H	2.13	0.52
1:E:277:SER:HB3	1:E:280:SER:HB2	1.91	0.52
1:F:529:GLU:OE1	1:F:574:ARG:NH2	2.36	0.52
1:D:757:HIS:O	1:D:761:VAL:HG12	2.10	0.52
1:F:141:GLY:N	1:F:154:SER:OG	2.42	0.52
1:F:358:THR:HG23	1:F:360:ASN:H	1.74	0.52
1:F:559:PHE:CE1	1:F:761:VAL:HG11	2.45	0.52
1:A:179:ILE:O	1:A:183:MET:HB2	2.08	0.52
1:D:636:GLU:C	1:D:638:SER:H	2.14	0.52
1:B:268:ARG:NH1	1:B:293:CYS:HB2	2.25	0.51
1:C:173:GLU:C	1:C:175:GLY:H	2.13	0.51
1:C:273:SER:HB3	1:C:296:TYR:CE1	2.43	0.51
1:C:409:VAL:HA	1:C:440:PHE:HE2	1.74	0.51
1:E:393:LYS:HB2	1:E:398:PHE:CE2	2.40	0.51
1:D:202:LEU:HD12	1:D:205:LEU:HB3	1.92	0.51
1:A:320:LEU:HG	1:A:346:ILE:CD1	2.40	0.51
1:A:597:PRO:CG	1:A:633:THR:HG22	2.37	0.51
1:C:412:MET:HG3	1:C:440:PHE:CD2	2.46	0.51
1:E:327:THR:HA	1:E:332:SER:HB3	1.92	0.51
1:D:134:GLN:HG3	1:D:140:TRP:CE2	2.46	0.51
1:D:690:ARG:HB2	1:D:706:ILE:HG21	1.91	0.51
1:A:160:VAL:O	1:A:164:SER:HB2	2.10	0.51
1:A:302:LYS:NZ	1:A:303:LYS:HB3	2.25	0.51
1:B:146:PHE:CD1	1:B:151:ARG:HG3	2.45	0.51
1:B:427:ASP:OD1	1:B:429:VAL:HG13	2.10	0.51
1:B:549:ASP:OD1	1:B:549:ASP:N	2.44	0.51
1:D:270:LEU:HB2	1:D:360:ASN:CB	2.40	0.51
1:A:380:GLY:HA2	1:A:722:LEU:O	2.10	0.51
1:C:669:ASP:OD1	1:C:727:ALA:HB3	2.11	0.51
1:E:420:GLN:OE1	1:E:471:PRO:HA	2.11	0.51
1:F:254:GLU:HA	1:F:283:THR:HG21	1.92	0.51
1:A:209:ALA:HB1	1:A:214:LEU:HD12	1.91	0.51
1:A:613:PHE:CD2	1:A:621:LEU:HD22	2.45	0.51
1:B:202:LEU:N	1:B:203:PRO:HD2	2.25	0.51
1:C:87:ILE:HD11	1:C:511:TYR:CD1	2.45	0.51
1:E:598:LYS:HG3	1:E:636:GLU:OE2	2.11	0.51

Continued on next page...

For Manuscript Review

Continued from previous page...

Atom-1	Atom-2	Interatomic distance (Å)	Clash overlap (Å)
1:D:324:ASP:OD2	1:D:328:ARG:NH2	2.28	0.51
1:D:598:LYS:HG2	1:D:636:GLU:OE2	2.10	0.51
1:F:135:LEU:CD1	1:F:139:SER:H	2.24	0.51
1:F:253:LEU:HD12	1:F:256:MET:HG3	1.92	0.51
1:F:380:GLY:HA2	1:F:722:LEU:O	2.11	0.51
1:C:682:SER:HB3	1:C:713:GLU:OE1	2.11	0.51
1:C:737:LEU:HG	1:C:741:LYS:HD2	1.93	0.51
1:D:173:GLU:C	1:D:175:GLY:H	2.14	0.51
1:D:414:ASN:OD1	1:D:417:ARG:NH1	2.44	0.51
1:E:445:ARG:HD2	1:E:465:GLU:OE2	2.10	0.51
1:F:134:GLN:HG3	1:F:140:TRP:CZ2	2.46	0.51
1:F:173:GLU:O	1:F:175:GLY:N	2.44	0.51
1:A:79:THR:HG23	1:A:82:THR:H	1.75	0.51
1:D:545:PHE:CD2	1:D:590:HIS:HB2	2.46	0.51
1:A:265:LEU:HD22	1:A:268:ARG:CZ	2.40	0.51
1:B:525:GLU:O	1:B:529:GLU:HB2	2.11	0.51
1:E:216:ILE:HG13	1:E:218:TYR:CE2	2.45	0.51
1:E:251:HIS:CE1	1:E:277:SER:HB2	2.45	0.51
1:D:347:PHE:CD1	1:D:381:TYR:HD2	2.28	0.51
1:A:147:MET:O	1:A:151:ARG:HG2	2.11	0.51
1:A:259:LEU:HD12	1:A:259:LEU:O	2.11	0.51
1:C:152:ILE:HG12	1:C:156:LEU:HD23	1.93	0.50
1:E:440:PHE:CD1	1:E:440:PHE:C	2.84	0.50
1:F:565:ILE:HD13	1:F:570:ARG:CZ	2.42	0.50
1:A:737:LEU:HG	1:A:741:LYS:HD2	1.93	0.50
1:B:112:LEU:HD11	1:B:282:ALA:HB1	1.92	0.50
1:B:176:LEU:HA	1:B:179:ILE:HD12	1.92	0.50
1:C:390:LYS:C	1:C:391:PHE:HD1	2.15	0.50
1:C:565:ILE:O	1:C:574:ARG:HD3	2.12	0.50
1:C:652:ARG:O	1:C:656:ILE:HG13	2.11	0.50
1:E:508:ASN:OD1	1:E:510:LEU:HB3	2.11	0.50
1:D:329:LEU:HD23	1:D:515:ALA:HB2	1.93	0.50
1:F:321:TRP:O	1:F:325:ARG:HG2	2.11	0.50
1:C:247:THR:OG1	1:C:248:THR:N	2.45	0.50
1:C:720:ARG:NH2	1:D:627:GLN:OE1	2.42	0.50
1:D:88:ARG:HD3	1:D:92:ARG:NH2	2.27	0.50
1:F:135:LEU:HG	1:F:139:SER:O	2.11	0.50
1:B:115:LEU:HD23	1:E:116:ASP:HB3	1.94	0.50
1:E:162:LEU:HD22	1:E:168:HIS:HB2	1.92	0.50
1:D:193:TRP:CZ2	1:D:451:LYS:HD2	2.47	0.50
1:F:559:PHE:HE1	1:F:761:VAL:HG11	1.77	0.50

Continued on next page...

For Manuscript Review

Continued from previous page...

Atom-1	Atom-2	Interatomic distance (Å)	Clash overlap (Å)
1:A:108:LEU:HA	1:A:111:LEU:HD23	1.92	0.50
1:A:333:ARG:HG3	1:A:334:HIS:HD2	1.76	0.50
1:A:613:PHE:CE2	1:A:621:LEU:HD22	2.47	0.50
1:B:214:LEU:O	1:B:216:ILE:HG12	2.12	0.50
1:F:579:ARG:NH1	1:F:612:PRO:HD3	2.27	0.50
1:F:724:GLY:HA2	1:F:733:ARG:NH2	2.26	0.50
1:A:268:ARG:HB2	1:A:273:SER:O	2.11	0.50
1:B:397:PHE:CE2	1:B:433:ALA:HA	2.47	0.50
1:A:668:PRO:HA	1:A:671:TRP:CE3	2.47	0.50
1:B:377:ARG:HD3	1:B:423:PHE:CZ	2.47	0.50
1:A:270:LEU:HB2	1:A:360:ASN:CB	2.41	0.50
1:B:91:LEU:HD21	1:B:326:LEU:HD11	1.94	0.50
1:B:535:ARG:NH2	1:D:340:GLU:OE1	2.44	0.50
1:B:605:GLU:HA	1:B:629:LEU:CD1	2.41	0.50
1:D:220:GLU:OE1	1:D:221:PRO:HD2	2.12	0.50
1:F:373:PHE:CZ	1:F:385:PRO:HB3	2.46	0.50
1:B:106:THR:OG1	1:B:307:GLY:HA2	2.12	0.49
1:B:427:ASP:OD1	1:B:428:GLY:N	2.45	0.49
1:E:377:ARG:HD3	1:E:421:LEU:HB3	1.94	0.49
1:E:533:LEU:HD22	1:E:554:LEU:HD13	1.94	0.49
1:D:270:LEU:HD22	1:D:360:ASN:HB3	1.93	0.49
1:D:687:LEU:HD21	1:D:744:CYS:HA	1.94	0.49
1:F:441:LEU:HB2	1:F:468:MET:HE1	1.93	0.49
1:B:377:ARG:HD2	1:B:421:LEU:HB3	1.94	0.49
1:B:682:SER:O	1:B:686:LYS:HD3	2.12	0.49
1:C:311:ILE:HD11	1:C:357:HIS:NE2	2.27	0.49
1:A:206:LEU:HG	1:A:218:TYR:HD2	1.77	0.49
1:C:651:VAL:HG23	1:C:735:THR:HG22	1.94	0.49
1:E:608:ILE:CD1	1:E:621:LEU:HD22	2.25	0.49
1:F:562:SER:HA	1:F:574:ARG:HD2	1.95	0.49
1:F:680:THR:HG21	1:F:736:PHE:HD1	1.76	0.49
1:C:320:LEU:HD21	1:C:372:GLY:HA2	1.93	0.49
1:E:729:ASN:OD1	1:E:730:ARG:N	2.44	0.49
1:B:131:VAL:HG13	1:B:132:GLN:HG3	1.93	0.49
1:B:482:ARG:HD3	1:B:519:PHE:CE1	2.48	0.49
1:E:320:LEU:CD2	1:E:346:ILE:HG12	2.43	0.49
1:E:536:TRP:CE2	1:E:579:ARG:HD3	2.48	0.49
1:D:530:LEU:HD21	1:D:558:TYR:CD1	2.47	0.49
1:B:569:ASN:C	1:B:571:ALA:H	2.16	0.49
1:C:246:PRO:HA	1:C:267:LEU:HD13	1.93	0.49
1:F:209:ALA:HB1	1:F:214:LEU:HD11	1.95	0.49

Continued on next page...

For Manuscript Review

Continued from previous page...

Atom-1	Atom-2	Interatomic distance (Å)	Clash overlap (Å)
1:F:672:GLU:OE2	1:F:726:SER:HB2	2.12	0.49
1:A:206:LEU:HD13	1:A:227:TYR:OH	2.12	0.49
1:C:80:THR:N	1:C:82:THR:HG1	2.10	0.49
1:D:374:ARG:NH2	1:D:375:LEU:HD21	2.27	0.49
1:D:397:PHE:CE2	1:D:433:ALA:HA	2.48	0.49
1:D:565:ILE:O	1:D:574:ARG:HD3	2.13	0.49
1:A:386:CYS:O	1:A:389:LYS:HG2	2.11	0.49
1:A:597:PRO:HD2	1:A:636:GLU:CD	2.33	0.49
1:E:268:ARG:HB3	1:E:274:PHE:CE1	2.48	0.49
1:A:478:ARG:HD3	1:A:566:PHE:CG	2.48	0.49
1:A:666:GLN:N	1:A:666:GLN:OE1	2.46	0.49
1:B:460:ILE:HG22	1:B:461:PRO:HD3	1.95	0.49
1:E:247:THR:H	1:E:250:LEU:HD13	1.77	0.49
1:E:614:ASP:OD1	1:E:616:ALA:N	2.41	0.49
1:F:108:LEU:HA	1:F:111:LEU:HD12	1.93	0.49
1:A:635:LYS:NZ	1:A:645:ASP:OD2	2.45	0.49
1:B:134:GLN:HG3	1:B:140:TRP:CZ2	2.48	0.49
1:C:675:GLN:HG2	1:C:720:ARG:NH1	2.28	0.49
1:E:135:LEU:HB3	1:E:136:PRO:HD2	1.95	0.49
1:E:203:PRO:HG2	1:E:230:ARG:NH1	2.27	0.49
1:F:260:ASP:O	1:F:264:LEU:HB2	2.13	0.49
1:A:252:SER:HB3	1:A:498:LYS:HE2	1.94	0.49
1:A:597:PRO:CD	1:A:636:GLU:OE2	2.57	0.49
1:B:140:TRP:CD1	1:B:155:THR:HG23	2.48	0.48
1:E:83:MET:SD	1:E:513:LYS:HG2	2.53	0.48
1:E:393:LYS:HE2	1:E:402:GLY:H	1.77	0.48
1:A:333:ARG:HA	1:A:336:THR:HG23	1.95	0.48
1:D:410:THR:OG1	1:D:411:PRO:HD3	2.13	0.48
1:C:496:ILE:HD13	1:C:496:ILE:HA	1.65	0.48
1:E:416:TYR:HE1	1:E:434:GLU:HG3	1.78	0.48
1:D:155:THR:O	1:D:159:VAL:HG23	2.12	0.48
1:D:196:VAL:HG22	1:D:497:GLY:O	2.12	0.48
1:D:323:VAL:HG22	1:D:339:ILE:HG23	1.94	0.48
1:C:598:LYS:HB2	1:C:636:GLU:OE2	2.13	0.48
1:E:203:PRO:CG	1:E:230:ARG:HH11	2.25	0.48
1:D:389:LYS:HB3	1:D:429:VAL:HG11	1.96	0.48
1:D:413:TYR:HA	1:D:441:LEU:HD11	1.96	0.48
1:A:248:THR:HG23	1:A:403:GLU:OE2	2.13	0.48
1:A:613:PHE:HE1	1:A:617:TYR:CD2	2.31	0.48
1:C:135:LEU:HD21	1:C:151:ARG:HH22	1.78	0.48
1:C:683:ILE:O	1:C:687:LEU:HB2	2.13	0.48

Continued on next page...

For Manuscript Review

Continued from previous page...

Atom-1	Atom-2	Interatomic distance (Å)	Clash overlap (Å)
1:B:419:SER:HA	1:B:430:LEU:HD11	1.96	0.48
1:B:538:LEU:O	1:B:540:SER:N	2.47	0.48
1:B:661:HIS:HD1	1:B:662:VAL:H	1.60	0.48
1:C:173:GLU:HG2	1:C:176:LEU:HD23	1.94	0.48
1:E:186:LEU:HA	1:E:186:LEU:HD23	1.63	0.48
1:E:564:ASN:HB3	1:E:738:HIS:ND1	2.29	0.48
1:B:233:LYS:O	1:B:237:ILE:HG23	2.12	0.48
1:B:628:TRP:HB2	1:B:649:LEU:HD13	1.94	0.48
1:C:107:SER:OG	1:C:157:ALA:HB1	2.13	0.48
1:C:704:GLU:O	1:C:708:GLN:NE2	2.43	0.48
1:F:417:ARG:HG2	1:F:417:ARG:HH11	1.77	0.48
1:A:163:LYS:NZ	1:A:169:THR:HB	2.29	0.48
1:B:259:LEU:HD11	1:B:261:TRP:CZ2	2.49	0.48
1:E:202:LEU:N	1:E:203:PRO:HD2	2.28	0.48
1:F:403:GLU:O	1:F:405:ASN:N	2.47	0.48
1:F:432:ARG:O	1:F:435:VAL:HG12	2.13	0.48
1:A:559:PHE:CE2	1:A:758:ILE:HA	2.49	0.48
1:C:112:LEU:HD21	1:C:286:GLN:HG3	1.96	0.48
1:C:265:LEU:HG	1:C:268:ARG:HD2	1.95	0.48
1:A:312:TYR:CG	1:A:313:PRO:HA	2.48	0.48
1:A:358:THR:HG23	1:A:360:ASN:N	2.29	0.48
1:C:559:PHE:CG	1:C:758:ILE:HG12	2.49	0.48
1:C:664:THR:HA	1:D:664:THR:OG1	2.14	0.48
1:F:217:PRO:C	1:F:219:ASP:H	2.17	0.48
1:F:370:ALA:HB1	1:F:415:THR:HA	1.95	0.48
1:F:390:LYS:C	1:F:391:PHE:HD1	2.18	0.48
1:A:210:LYS:HB2	1:A:210:LYS:HE3	1.64	0.48
1:C:153:MET:HA	1:C:156:LEU:HG	1.95	0.47
1:E:711:ASP:O	1:E:715:GLN:HG3	2.14	0.47
1:D:135:LEU:CD1	1:D:137:ASP:HB3	2.44	0.47
1:D:140:TRP:CD1	1:D:155:THR:HG22	2.45	0.47
1:D:374:ARG:HG3	1:D:418:ALA:HA	1.95	0.47
1:C:663:LEU:HD22	1:C:663:LEU:H	1.79	0.47
1:E:494:VAL:HG22	1:E:502:ARG:CZ	2.44	0.47
1:A:273:SER:HB3	1:A:296:TYR:CZ	2.49	0.47
1:B:270:LEU:HB3	1:B:360:ASN:CB	2.39	0.47
1:E:416:TYR:CE1	1:E:434:GLU:HG3	2.49	0.47
1:E:652:ARG:O	1:E:656:ILE:HG13	2.14	0.47
1:F:424:PRO:HG3	1:F:716:GLU:HB2	1.96	0.47
1:F:635:LYS:O	1:F:638:SER:OG	2.28	0.47
1:A:449:ARG:O	1:A:450:MET:HG2	2.15	0.47

Continued on next page...

For Manuscript Review

Continued from previous page...

Atom-1	Atom-2	Interatomic distance (Å)	Clash overlap (Å)
1:C:129:TRP:NE1	1:C:133:ASN:ND2	2.62	0.47
1:F:491:SER:OG	1:F:509:ASP:OD2	2.29	0.47
1:F:537:TYR:CD2	1:F:554:LEU:HD22	2.49	0.47
1:C:171:LYS:HA	1:C:174:ARG:HD3	1.95	0.47
1:E:139:SER:HB3	1:E:178:PHE:CD2	2.49	0.47
1:E:390:LYS:C	1:E:391:PHE:HD1	2.18	0.47
1:D:540:SER:OG	1:D:542:LEU:HB2	2.15	0.47
1:F:298:ASP:OD2	1:F:302:LYS:HD2	2.14	0.47
1:F:441:LEU:HB2	1:F:468:MET:HE3	1.97	0.47
1:B:111:LEU:HD21	1:B:161:ALA:HA	1.97	0.47
1:B:373:PHE:HZ	1:B:377:ARG:NH2	2.13	0.47
1:C:131:VAL:CG2	1:C:162:LEU:HD21	2.45	0.47
1:C:173:GLU:O	1:C:175:GLY:N	2.47	0.47
1:C:246:PRO:HD3	1:C:267:LEU:HD22	1.97	0.47
1:C:670:LEU:HD21	1:D:673:TYR:CD1	2.49	0.47
1:E:647:ALA:HB2	1:E:743:PHE:CE2	2.49	0.47
1:F:247:THR:HG23	1:F:249:LEU:HG	1.97	0.47
1:A:205:LEU:HD12	1:A:205:LEU:HA	1.52	0.47
1:A:328:ARG:NH1	1:A:484:TYR:CZ	2.83	0.47
1:A:591:PHE:CE1	1:A:597:PRO:HD3	2.50	0.47
1:B:458:LYS:HD3	1:B:489:GLY:HA2	1.97	0.47
1:C:256:MET:O	1:C:259:LEU:HD22	2.15	0.47
1:C:445:ARG:NH1	1:C:465:GLU:OE1	2.48	0.47
1:E:203:PRO:CB	1:E:230:ARG:HH11	2.27	0.47
1:D:148:MET:O	1:D:152:ILE:HG22	2.14	0.47
1:D:173:GLU:HA	1:D:176:LEU:HB3	1.96	0.47
1:A:427:ASP:OD1	1:A:428:GLY:N	2.47	0.47
1:B:750:SER:O	1:B:753:THR:HG22	2.14	0.47
1:C:265:LEU:HA	1:C:268:ARG:HD2	1.97	0.47
1:C:530:LEU:HD12	1:C:530:LEU:HA	1.60	0.47
1:E:344:ASP:HB3	1:E:348:ARG:HH12	1.79	0.47
1:E:455:ALA:HB2	1:E:495:TRP:CE3	2.50	0.47
1:E:545:PHE:HB3	1:E:593:ARG:NH2	2.29	0.47
1:D:483:LEU:O	1:D:487:GLN:HG2	2.15	0.47
1:D:538:LEU:HD12	1:F:348:ARG:NH1	2.30	0.47
1:F:755:ASP:HA	1:F:758:ILE:HD12	1.97	0.47
1:B:160:VAL:HG11	1:B:208:MET:HG2	1.97	0.46
1:B:300:ILE:HG21	1:B:308:VAL:HG21	1.96	0.46
1:B:661:HIS:ND1	1:B:662:VAL:N	2.64	0.46
1:C:478:ARG:NH1	1:C:566:PHE:CE2	2.83	0.46
1:F:537:TYR:HD2	1:F:554:LEU:HD22	1.80	0.46

Continued on next page...

For Manuscript Review

Continued from previous page...

Atom-1	Atom-2	Interatomic distance (Å)	Clash overlap (Å)
1:C:251:HIS:CD2	1:C:277:SER:HB3	2.50	0.46
1:C:271:ASP:O	1:C:296:TYR:HB2	2.14	0.46
1:C:722:LEU:HD12	1:C:722:LEU:N	2.31	0.46
1:D:209:ALA:O	1:D:218:TYR:OH	2.32	0.46
1:A:699:SER:CB	1:A:702:LYS:HB2	2.46	0.46
1:E:374:ARG:NH2	1:E:375:LEU:HD21	2.29	0.46
1:E:386:CYS:O	1:E:389:LYS:HG2	2.16	0.46
1:E:544:LYS:HG2	1:E:545:PHE:HD1	1.80	0.46
1:D:325:ARG:O	1:D:329:LEU:HD13	2.16	0.46
1:D:632:TRP:CD1	1:D:642:ILE:HD13	2.50	0.46
1:F:577:TRP:CD1	1:F:577:TRP:C	2.88	0.46
1:F:621:LEU:HD12	1:F:621:LEU:HA	1.74	0.46
1:F:642:ILE:O	1:F:646:THR:HG23	2.15	0.46
1:A:355:LEU:C	1:A:364:LYS:HD2	2.35	0.46
1:A:370:ALA:HB1	1:A:415:THR:HA	1.97	0.46
1:B:238:PRO:HG2	1:B:241:VAL:HB	1.97	0.46
1:C:152:ILE:HG23	1:C:153:MET:HE2	1.96	0.46
1:C:328:ARG:NH1	1:C:484:TYR:OH	2.49	0.46
1:F:328:ARG:HH21	1:F:417:ARG:NH1	2.13	0.46
1:A:363:VAL:O	1:A:363:VAL:HG13	2.14	0.46
1:A:483:LEU:HD23	1:A:483:LEU:HA	1.65	0.46
1:E:629:LEU:HD23	1:E:629:LEU:HA	1.72	0.46
1:D:107:SER:HB2	1:D:161:ALA:HB2	1.97	0.46
1:D:445:ARG:NH1	1:D:465:GLU:HG3	2.31	0.46
1:B:673:TYR:CE1	1:A:670:LEU:HD21	2.51	0.46
1:C:150:ASP:HB3	1:C:195:LEU:CD1	2.46	0.46
1:D:112:LEU:HD23	1:D:112:LEU:HA	1.67	0.46
1:D:356:ALA:N	1:D:364:LYS:HE3	2.30	0.46
1:F:87:ILE:HD12	1:F:511:TYR:CE1	2.51	0.46
1:F:687:LEU:HD21	1:F:744:CYS:HA	1.96	0.46
1:A:554:LEU:HA	1:A:582:LEU:HD21	1.97	0.46
1:F:323:VAL:HG22	1:F:339:ILE:HG23	1.98	0.46
1:F:403:GLU:OE1	1:F:405:ASN:ND2	2.49	0.46
1:B:214:LEU:O	1:B:214:LEU:CD1	2.64	0.46
1:B:656:ILE:HG12	1:A:667:ARG:CD	2.46	0.46
1:C:153:MET:HE2	1:C:153:MET:H	1.81	0.46
1:D:320:LEU:HD13	1:D:371:MET:HB3	1.98	0.46
1:F:85:ASP:HA	1:F:88:ARG:HB3	1.98	0.46
1:F:87:ILE:HD12	1:F:511:TYR:HD1	1.80	0.46
1:F:325:ARG:HA	1:F:325:ARG:HD3	1.80	0.46
1:A:642:ILE:O	1:A:645:ASP:N	2.48	0.46

Continued on next page...

For Manuscript Review

Continued from previous page...

Atom-1	Atom-2	Interatomic distance (Å)	Clash overlap (Å)
1:B:709:GLN:O	1:B:709:GLN:HG2	2.16	0.46
1:C:265:LEU:HA	1:C:268:ARG:CD	2.46	0.46
1:C:457:ALA:HB3	1:C:460:ILE:HG12	1.98	0.46
1:E:107:SER:HB2	1:E:161:ALA:HB2	1.98	0.46
1:D:484:TYR:HA	1:D:487:GLN:CG	2.46	0.46
1:F:576:GLY:O	1:F:580:VAL:HG12	2.16	0.46
1:B:139:SER:HB3	1:B:178:PHE:CG	2.51	0.46
1:C:270:LEU:HB2	1:C:360:ASN:CB	2.44	0.46
1:E:152:ILE:HD13	1:E:152:ILE:HA	1.73	0.46
1:D:638:SER:OG	1:D:639:GLN:N	2.48	0.46
1:F:135:LEU:HD21	1:F:151:ARG:NH2	2.31	0.46
1:F:275:HIS:CD2	1:F:363:VAL:HG11	2.51	0.46
1:F:358:THR:HG23	1:F:360:ASN:N	2.31	0.46
1:F:455:ALA:HB2	1:F:495:TRP:CZ3	2.51	0.46
1:A:288:THR:HG22	1:A:290:ASP:HB2	1.98	0.46
1:A:320:LEU:HD23	1:A:320:LEU:HA	1.78	0.46
1:A:419:SER:HA	1:A:430:LEU:HD11	1.97	0.46
1:A:440:PHE:CD1	1:A:440:PHE:C	2.89	0.46
1:B:155:THR:O	1:B:159:VAL:HG23	2.16	0.45
1:E:721:VAL:HG11	1:E:737:LEU:HB2	1.97	0.45
1:D:83:MET:SD	1:D:513:LYS:HG2	2.56	0.45
1:D:686:LYS:HD2	1:D:689:ARG:NH2	2.30	0.45
1:F:127:ILE:O	1:F:131:VAL:HG13	2.16	0.45
1:F:214:LEU:HD12	1:F:218:TYR:OH	2.16	0.45
1:F:414:ASN:HA	1:F:417:ARG:HD3	1.98	0.45
1:B:146:PHE:HD1	1:B:151:ARG:HG3	1.82	0.45
1:B:214:LEU:HD13	1:B:216:ILE:HG12	1.97	0.45
1:B:410:THR:CG2	1:B:460:ILE:HD11	2.45	0.45
1:C:348:ARG:NH2	1:E:538:LEU:HD12	2.31	0.45
1:D:343:LEU:HD23	1:D:343:LEU:HA	1.52	0.45
1:F:565:ILE:HD11	1:F:570:ARG:HB3	1.98	0.45
1:F:608:ILE:HD13	1:F:625:TRP:HB3	1.97	0.45
1:F:629:LEU:O	1:F:632:TRP:HB2	2.16	0.45
1:A:663:LEU:HD22	1:A:663:LEU:H	1.81	0.45
1:B:292:LYS:HD2	1:B:292:LYS:HA	1.70	0.45
1:C:494:VAL:HG13	1:C:502:ARG:HG2	1.98	0.45
1:C:680:THR:HG22	1:C:740:VAL:HG21	1.98	0.45
1:F:151:ARG:O	1:F:155:THR:OG1	2.28	0.45
1:F:488:TYR:CD2	1:F:512:LEU:HG	2.39	0.45
1:A:319:ARG:HD2	1:A:345:TYR:HD2	1.81	0.45
1:E:139:SER:HB3	1:E:178:PHE:CG	2.51	0.45

Continued on next page...

For Manuscript Review

Continued from previous page...

Atom-1	Atom-2	Interatomic distance (Å)	Clash overlap (Å)
1:E:662:VAL:HG22	1:F:670:LEU:HD22	1.98	0.45
1:D:140:TRP:HD1	1:D:155:THR:HA	1.75	0.45
1:D:745:TYR:O	1:D:749:CYS:HB2	2.16	0.45
1:A:445:ARG:HD2	1:A:465:GLU:OE1	2.17	0.45
1:B:163:LYS:HG3	1:B:212:LEU:HD23	1.99	0.45
1:C:523:GLN:O	1:C:527:ARG:HG3	2.16	0.45
1:C:236:LYS:HB2	1:C:236:LYS:HE3	1.58	0.45
1:C:388:LEU:HD22	1:C:430:LEU:HD12	1.99	0.45
1:E:321:TRP:O	1:E:325:ARG:HG2	2.16	0.45
1:D:519:PHE:CZ	1:D:761:VAL:HG23	2.51	0.45
1:D:631:ALA:HB1	1:D:645:ASP:OD2	2.16	0.45
1:F:247:THR:HG23	1:F:250:LEU:HD13	1.97	0.45
1:F:650:LEU:HD23	1:F:650:LEU:HA	1.76	0.45
1:B:402:GLY:O	1:B:404:SER:N	2.50	0.45
1:C:721:VAL:HG11	1:C:737:LEU:HD22	1.98	0.45
1:D:195:LEU:HD13	1:D:498:LYS:O	2.17	0.45
1:D:325:ARG:HA	1:D:325:ARG:HD3	1.72	0.45
1:A:296:TYR:CD2	1:A:297:LEU:HD23	2.51	0.45
1:E:190:GLU:HB2	1:E:193:TRP:HB2	1.99	0.45
1:E:391:PHE:CD1	1:E:391:PHE:N	2.83	0.45
1:E:478:ARG:HG3	1:E:522:PHE:CZ	2.52	0.45
1:E:575:LEU:HD23	1:E:575:LEU:HA	1.83	0.45
1:D:614:ASP:OD1	1:D:615:ASP:N	2.50	0.45
1:F:128:ASP:O	1:F:131:VAL:HG22	2.16	0.45
1:F:489:GLY:HA3	1:F:493:ASP:OD1	2.17	0.45
1:B:519:PHE:HZ	1:B:761:VAL:HG23	1.82	0.45
1:C:159:VAL:HG13	1:C:172:CYS:SG	2.57	0.45
1:C:377:ARG:NH2	1:C:426:ASP:OD2	2.50	0.45
1:E:195:LEU:HD13	1:E:498:LYS:O	2.17	0.45
1:E:312:TYR:CG	1:E:313:PRO:HA	2.51	0.45
1:E:413:TYR:CD1	1:E:464:VAL:HG22	2.51	0.45
1:D:570:ARG:HH21	1:D:573:GLU:CD	2.21	0.45
1:F:326:LEU:HD22	1:F:335:PHE:HE2	1.82	0.45
1:A:731:LEU:HD13	1:A:731:LEU:HA	1.70	0.45
1:B:140:TRP:HD1	1:B:155:THR:HG23	1.81	0.45
1:C:533:LEU:HD23	1:C:554:LEU:HD11	1.99	0.45
1:C:545:PHE:CB	1:C:593:ARG:HH22	2.30	0.45
1:C:664:THR:CG2	1:D:663:LEU:HD12	2.47	0.45
1:F:104:TYR:O	1:F:108:LEU:HD12	2.17	0.45
1:F:554:LEU:HD13	1:F:582:LEU:HD11	1.99	0.45
1:A:692:LEU:HD23	1:A:692:LEU:HA	1.80	0.45

Continued on next page...

For Manuscript Review

Continued from previous page...

Atom-1	Atom-2	Interatomic distance (Å)	Clash overlap (Å)
1:C:169:THR:HA	1:C:172:CYS:CB	2.44	0.44
1:E:94:ILE:HD12	1:E:94:ILE:HA	1.69	0.44
1:E:131:VAL:HG11	1:E:168:HIS:CE1	2.51	0.44
1:E:606:GLU:CD	1:E:622:ARG:HH22	2.19	0.44
1:D:294:PHE:O	1:D:297:LEU:N	2.50	0.44
1:A:540:SER:HB2	1:A:610:LEU:HD22	1.99	0.44
1:C:391:PHE:CD1	1:C:391:PHE:N	2.85	0.44
1:E:427:ASP:OD1	1:E:428:GLY:N	2.50	0.44
1:D:104:TYR:OH	1:D:201:ALA:HB1	2.17	0.44
1:D:494:VAL:HG22	1:D:502:ARG:NE	2.32	0.44
1:A:87:ILE:O	1:A:91:LEU:HD23	2.18	0.44
1:C:482:ARG:HD3	1:C:519:PHE:CE1	2.52	0.44
1:C:499:VAL:HG12	1:C:500:LEU:N	2.31	0.44
1:E:529:GLU:HG2	1:E:575:LEU:HD11	1.98	0.44
1:E:537:TYR:CD2	1:E:543:GLU:HG2	2.52	0.44
1:E:608:ILE:HD12	1:E:625:TRP:HB2	1.98	0.44
1:F:420:GLN:HB3	1:F:472:TRP:HB2	1.99	0.44
1:F:533:LEU:HD13	1:F:554:LEU:HD11	1.99	0.44
1:F:651:VAL:HG12	1:F:739:VAL:CG2	2.46	0.44
1:A:114:ARG:HH12	1:A:120:GLY:H	1.66	0.44
1:A:193:TRP:CE3	1:A:456:ILE:HG22	2.52	0.44
1:B:575:LEU:HD23	1:B:575:LEU:HA	1.85	0.44
1:C:83:MET:HG2	1:C:510:LEU:HD11	2.00	0.44
1:D:629:LEU:O	1:D:632:TRP:HB2	2.17	0.44
1:F:319:ARG:HD2	1:F:345:TYR:HD2	1.82	0.44
1:F:355:LEU:C	1:F:364:LYS:HG3	2.38	0.44
1:A:203:PRO:HB2	1:A:230:ARG:NH2	2.33	0.44
1:A:494:VAL:HG22	1:A:502:ARG:CZ	2.47	0.44
1:C:156:LEU:HD11	1:C:205:LEU:CG	2.46	0.44
1:C:427:ASP:OD1	1:C:428:GLY:N	2.50	0.44
1:E:105:ASP:O	1:E:109:VAL:HG23	2.18	0.44
1:E:690:ARG:HD2	1:E:707:ASP:OD1	2.17	0.44
1:D:264:LEU:HA	1:D:267:LEU:HD23	2.00	0.44
1:D:328:ARG:O	1:D:481:THR:HG23	2.18	0.44
1:D:712:LEU:HD23	1:D:712:LEU:HA	1.73	0.44
1:F:400:LEU:HB2	1:F:403:GLU:HB3	2.00	0.44
1:A:80:THR:HA	1:A:83:MET:HE2	1.99	0.44
1:B:177:LEU:O	1:B:181:GLU:HG3	2.17	0.44
1:B:478:ARG:HD3	1:B:566:PHE:CG	2.53	0.44
1:E:388:LEU:HA	1:E:388:LEU:HD23	1.64	0.44
1:E:624:ALA:HB2	1:F:671:TRP:CH2	2.52	0.44

Continued on next page...

For Manuscript Review

Continued from previous page...

Atom-1	Atom-2	Interatomic distance (Å)	Clash overlap (Å)
1:D:526:CYS:O	1:D:530:LEU:HD23	2.18	0.44
1:F:206:LEU:HA	1:F:209:ALA:HB3	1.99	0.44
1:F:391:PHE:N	1:F:391:PHE:CD1	2.86	0.44
1:F:632:TRP:CD1	1:F:642:ILE:HD13	2.53	0.44
1:A:148:MET:CE	1:A:152:ILE:HG12	2.47	0.44
1:A:238:PRO:HG2	1:A:241:VAL:CB	2.45	0.44
1:B:216:ILE:O	1:B:218:TYR:N	2.51	0.44
1:E:317:TYR:CD1	1:E:321:TRP:CD1	3.06	0.44
1:F:199:GLU:CD	1:F:199:GLU:H	2.21	0.44
1:B:731:LEU:HA	1:B:731:LEU:HD23	1.66	0.44
1:C:466:TYR:HE2	1:C:480:GLU:CB	2.31	0.44
1:D:233:LYS:O	1:D:237:ILE:HG23	2.18	0.44
1:D:549:ASP:OD2	1:D:552:THR:OG1	2.35	0.44
1:F:608:ILE:HD13	1:F:625:TRP:CB	2.47	0.44
1:B:176:LEU:O	1:B:180:GLN:HG3	2.18	0.44
1:C:350:TRP:HA	1:C:355:LEU:HD21	1.98	0.44
1:E:366:ILE:HG22	1:E:411:PRO:HB3	1.99	0.44
1:A:106:THR:OG1	1:A:308:VAL:N	2.51	0.44
1:A:370:ALA:HB2	1:A:415:THR:HG22	2.00	0.44
1:A:456:ILE:HG12	1:A:494:VAL:O	2.17	0.44
1:C:146:PHE:HA	1:C:151:ARG:HD3	1.99	0.43
1:E:253:LEU:HA	1:E:253:LEU:HD23	1.85	0.43
1:E:410:THR:HB	1:E:411:PRO:HD3	2.00	0.43
1:F:590:HIS:O	1:F:594:ILE:HG12	2.17	0.43
1:A:397:PHE:CE2	1:A:433:ALA:HA	2.53	0.43
1:A:591:PHE:CE1	1:A:597:PRO:N	2.80	0.43
1:B:512:LEU:HD23	1:B:512:LEU:HA	1.46	0.43
1:E:148:MET:HE1	1:E:182:ASN:HB3	2.00	0.43
1:E:256:MET:HB3	1:E:259:LEU:CD2	2.48	0.43
1:E:313:PRO:O	1:E:314:LEU:HD23	2.17	0.43
1:E:559:PHE:CE1	1:E:761:VAL:HG11	2.50	0.43
1:F:145:PHE:HZ	1:F:193:TRP:CH2	2.36	0.43
1:F:209:ALA:HB1	1:F:214:LEU:HD21	2.00	0.43
1:A:227:TYR:O	1:A:230:ARG:HB3	2.18	0.43
1:A:341:ASP:O	1:A:344:ASP:HB2	2.19	0.43
1:B:130:ILE:O	1:B:140:TRP:HZ3	2.02	0.43
1:C:376:LEU:CB	1:C:383:VAL:HG21	2.47	0.43
1:C:690:ARG:HD2	1:C:748:TYR:HE1	1.84	0.43
1:D:643:GLU:HG3	1:D:688:SER:HB2	2.00	0.43
1:A:376:LEU:HA	1:A:376:LEU:HD23	1.76	0.43
1:A:652:ARG:O	1:A:656:ILE:HG13	2.18	0.43

Continued on next page...

For Manuscript Review

Continued from previous page...

Atom-1	Atom-2	Interatomic distance (Å)	Clash overlap (Å)
1:B:534:ARG:HH21	1:B:555:MET:HE3	1.83	0.43
1:B:559:PHE:HE1	1:B:761:VAL:CG1	2.25	0.43
1:B:661:HIS:CD2	1:A:667:ARG:HG3	2.53	0.43
1:C:106:THR:HA	1:C:308:VAL:CG1	2.49	0.43
1:C:346:ILE:HG22	1:C:376:LEU:HD11	1.99	0.43
1:E:750:SER:HB2	1:E:753:THR:H	1.83	0.43
1:D:669:ASP:N	1:D:669:ASP:OD1	2.51	0.43
1:F:413:TYR:O	1:F:417:ARG:HD3	2.18	0.43
1:A:608:ILE:C	1:A:610:LEU:N	2.72	0.43
1:B:343:LEU:HD23	1:B:343:LEU:HA	1.74	0.43
1:C:538:LEU:HD12	1:A:348:ARG:CZ	2.48	0.43
1:D:377:ARG:NH2	1:D:426:ASP:OD2	2.52	0.43
1:D:447:SER:C	1:D:449:ARG:H	2.21	0.43
1:D:516:LYS:HG2	1:D:766:VAL:HG11	2.00	0.43
1:D:731:LEU:HD23	1:D:731:LEU:HA	1.82	0.43
1:A:377:ARG:NH2	1:A:426:ASP:OD2	2.51	0.43
1:B:88:ARG:HG2	1:B:335:PHE:HD1	1.84	0.43
1:C:478:ARG:NH1	1:C:566:PHE:CD2	2.87	0.43
1:C:687:LEU:HD23	1:C:687:LEU:HA	1.88	0.43
1:C:723:GLN:OE1	1:C:726:SER:HB2	2.18	0.43
1:E:97:GLY:HA2	1:E:318:GLU:OE1	2.19	0.43
1:F:315:ASP:OD1	1:F:359:LYS:N	2.44	0.43
1:F:628:TRP:HE1	1:F:646:THR:CG2	2.24	0.43
1:F:632:TRP:CE2	1:F:642:ILE:HD13	2.54	0.43
1:A:199:GLU:OE1	1:A:233:LYS:NZ	2.38	0.43
1:A:327:THR:HG1	1:A:379:TYR:HH	1.63	0.43
1:B:87:ILE:O	1:B:91:LEU:HD13	2.18	0.43
1:B:209:ALA:CA	1:B:214:LEU:HD21	2.48	0.43
1:C:285:PHE:HA	1:C:288:THR:O	2.19	0.43
1:E:545:PHE:HB3	1:E:593:ARG:NH1	2.34	0.43
1:D:223:LEU:O	1:D:227:TYR:HB2	2.19	0.43
1:F:270:LEU:HB2	1:F:360:ASN:CB	2.36	0.43
1:F:498:LYS:HB2	1:F:498:LYS:HE3	1.64	0.43
1:A:121:PRO:HB3	1:A:165:TRP:CZ2	2.54	0.43
1:A:128:ASP:O	1:A:132:GLN:HG3	2.19	0.43
1:A:181:GLU:O	1:A:184:TRP:HD1	2.01	0.43
1:B:144:SER:HB2	1:B:502:ARG:HH12	1.84	0.43
1:B:300:ILE:CG2	1:B:308:VAL:CG2	2.97	0.43
1:B:508:ASN:OD1	1:B:510:LEU:N	2.45	0.43
1:C:106:THR:HG21	1:C:129:TRP:CE3	2.54	0.43
1:E:352:PRO:O	1:E:387:VAL:HG23	2.19	0.43

Continued on next page...

For Manuscript Review

Continued from previous page...

Atom-1	Atom-2	Interatomic distance (Å)	Clash overlap (Å)
1:D:271:ASP:OD2	1:D:313:PRO:HD3	2.19	0.43
1:D:329:LEU:HD12	1:D:484:TYR:HE2	1.83	0.43
1:D:373:PHE:CZ	1:D:385:PRO:HB3	2.53	0.43
1:D:657:PHE:O	1:D:659:GLY:N	2.50	0.43
1:D:676:LEU:HD13	1:D:736:PHE:CD2	2.54	0.43
1:F:477:PRO:HB2	1:F:566:PHE:HE2	1.84	0.43
1:A:297:LEU:O	1:A:301:VAL:HG23	2.19	0.43
1:A:558:TYR:CD1	1:A:558:TYR:C	2.92	0.43
1:A:692:LEU:O	1:A:696:ASN:HB2	2.18	0.43
1:B:638:SER:HB3	1:A:712:LEU:CD2	2.49	0.43
1:E:365:ASP:C	1:E:391:PHE:HE2	2.22	0.43
1:D:104:TYR:HB3	1:D:499:VAL:CG2	2.48	0.43
1:D:125:SER:HA	1:D:128:ASP:HB2	1.99	0.43
1:F:478:ARG:HA	1:F:478:ARG:HD2	1.43	0.43
1:F:577:TRP:HD1	1:F:577:TRP:O	2.02	0.43
1:A:226:ILE:HG21	1:A:226:ILE:HD13	1.76	0.43
1:A:242:LEU:HD22	1:A:243:HIS:CE1	2.54	0.43
1:A:375:LEU:HD23	1:A:375:LEU:HA	1.79	0.43
1:A:548:THR:O	1:A:550:PRO:HD3	2.18	0.43
1:A:613:PHE:HD1	1:A:614:ASP:N	2.17	0.43
1:B:325:ARG:HA	1:B:325:ARG:HD3	1.88	0.43
1:C:288:THR:HG22	1:C:290:ASP:HB2	2.01	0.43
1:C:452:ASP:HB3	1:C:455:ALA:O	2.19	0.43
1:C:589:SER:OG	1:C:592:ARG:NH2	2.45	0.43
1:E:576:GLY:HA3	1:E:657:PHE:CE2	2.54	0.43
1:E:605:GLU:O	1:E:608:ILE:HG22	2.19	0.43
1:D:325:ARG:HG3	1:D:506:PHE:CE1	2.54	0.43
1:F:264:LEU:HA	1:F:264:LEU:HD12	1.71	0.43
1:F:272:GLY:HA3	1:F:292:LYS:HG2	2.00	0.43
1:F:472:TRP:O	1:F:741:LYS:HE3	2.19	0.43
1:F:702:LYS:HA	1:F:702:LYS:HD3	1.82	0.43
1:A:265:LEU:HA	1:A:265:LEU:HD23	1.82	0.43
1:A:538:LEU:O	1:A:540:SER:N	2.51	0.43
1:B:529:GLU:HG2	1:B:558:TYR:OH	2.19	0.42
1:B:538:LEU:HD12	1:D:348:ARG:CZ	2.49	0.42
1:B:690:ARG:HD2	1:B:707:ASP:OD1	2.19	0.42
1:D:152:ILE:HG23	1:D:153:MET:N	2.33	0.42
1:D:403:GLU:HG2	1:D:405:ASN:ND2	2.34	0.42
1:D:660:ARG:HD2	1:D:729:ASN:ND2	2.34	0.42
1:F:319:ARG:CD	1:F:345:TYR:HD2	2.32	0.42
1:F:338:GLU:HA	1:F:341:ASP:HB2	2.01	0.42

Continued on next page...

For Manuscript Review

Continued from previous page...

Atom-1	Atom-2	Interatomic distance (Å)	Clash overlap (Å)
1:F:561:ALA:O	1:F:565:ILE:HG23	2.19	0.42
1:F:687:LEU:HD13	1:F:710:VAL:HG21	2.00	0.42
1:A:157:ALA:HA	1:A:160:VAL:CG2	2.48	0.42
1:A:676:LEU:HD23	1:A:676:LEU:HA	1.65	0.42
1:A:699:SER:HB2	1:A:702:LYS:HB2	2.00	0.42
1:B:92:ARG:HH22	1:E:92:ARG:HH11	1.66	0.42
1:B:202:LEU:HD12	1:B:202:LEU:HA	1.80	0.42
1:B:300:ILE:HG23	1:B:308:VAL:HG23	2.00	0.42
1:C:131:VAL:HG23	1:C:162:LEU:HD21	2.01	0.42
1:C:291:GLN:O	1:C:295:GLU:HG3	2.19	0.42
1:C:670:LEU:HD23	1:C:670:LEU:C	2.40	0.42
1:E:676:LEU:HD23	1:E:676:LEU:HA	1.76	0.42
1:D:470:TYR:HE1	1:D:749:CYS:SG	2.41	0.42
1:D:728:ILE:O	1:D:729:ASN:C	2.57	0.42
1:F:84:ILE:HG23	1:F:335:PHE:CE1	2.53	0.42
1:F:202:LEU:O	1:F:206:LEU:HG	2.19	0.42
1:A:603:ASN:O	1:A:604:LEU:HB2	2.19	0.42
1:B:91:LEU:HD21	1:B:326:LEU:CD1	2.49	0.42
1:C:142:ASP:N	1:C:142:ASP:OD1	2.52	0.42
1:C:533:LEU:HD12	1:C:533:LEU:HA	1.62	0.42
1:C:691:VAL:O	1:C:695:GLU:HG2	2.19	0.42
1:F:126:THR:O	1:F:130:ILE:HG12	2.19	0.42
1:B:632:TRP:O	1:B:636:GLU:HB2	2.19	0.42
1:B:711:ASP:O	1:B:715:GLN:HG3	2.19	0.42
1:C:110:ALA:C	1:C:112:LEU:H	2.23	0.42
1:C:156:LEU:HD11	1:C:205:LEU:HG	2.00	0.42
1:C:173:GLU:HA	1:C:176:LEU:HB3	2.00	0.42
1:C:538:LEU:HD12	1:A:348:ARG:NH1	2.34	0.42
1:C:664:THR:CB	1:D:664:THR:H	2.32	0.42
1:E:408:SER:HB2	1:E:411:PRO:CD	2.49	0.42
1:F:276:CYS:O	1:F:311:ILE:HA	2.19	0.42
1:A:99:ILE:HG12	1:A:100:SER:N	2.34	0.42
1:A:153:MET:HE3	1:A:153:MET:HB2	1.66	0.42
1:A:419:SER:HB2	1:A:434:GLU:HB2	2.01	0.42
1:B:341:ASP:O	1:B:344:ASP:HB2	2.19	0.42
1:B:678:GLN:HG2	1:A:678:GLN:HG2	2.01	0.42
1:C:152:ILE:HG23	1:C:153:MET:H	1.85	0.42
1:C:233:LYS:HE3	1:C:233:LYS:HB3	1.84	0.42
1:E:549:ASP:HA	1:E:550:PRO:HD3	1.82	0.42
1:D:216:ILE:HA	1:D:217:PRO:HD3	1.85	0.42
1:D:420:GLN:HA	1:D:420:GLN:NE2	2.34	0.42

Continued on next page...

For Manuscript Review

Continued from previous page...

Atom-1	Atom-2	Interatomic distance (Å)	Clash overlap (Å)
1:F:130:ILE:HD12	1:F:158:CYS:SG	2.60	0.42
1:A:94:ILE:HG23	1:A:319:ARG:NH2	2.34	0.42
1:B:102:SER:OG	1:B:105:ASP:HB2	2.20	0.42
1:B:444:ARG:HE	1:B:444:ARG:HB2	1.54	0.42
1:C:312:TYR:CG	1:C:313:PRO:HA	2.54	0.42
1:D:670:LEU:HD12	1:D:670:LEU:HA	1.85	0.42
1:F:721:VAL:HG11	1:F:737:LEU:HB2	2.02	0.42
1:F:745:TYR:CE1	1:F:749:CYS:SG	3.13	0.42
1:A:140:TRP:HD1	1:A:155:THR:HA	1.81	0.42
1:A:220:GLU:HB3	1:A:223:LEU:HD13	2.01	0.42
1:A:320:LEU:HD21	1:A:372:GLY:HA2	2.00	0.42
1:B:264:LEU:HD12	1:B:264:LEU:HA	1.73	0.42
1:C:163:LYS:HB2	1:C:163:LYS:HE2	1.73	0.42
1:C:300:ILE:HG21	1:C:308:VAL:HG23	2.01	0.42
1:C:543:GLU:C	1:C:545:PHE:H	2.21	0.42
1:C:632:TRP:CE2	1:C:642:ILE:HD13	2.54	0.42
1:E:412:MET:HG3	1:E:440:PHE:CD2	2.55	0.42
1:A:271:ASP:OD2	1:A:313:PRO:HD3	2.19	0.42
1:A:452:ASP:HB3	1:A:455:ALA:O	2.18	0.42
1:B:444:ARG:HG3	1:B:449:ARG:HB2	2.01	0.42
1:C:718:THR:O	1:C:722:LEU:HD13	2.20	0.42
1:D:96:GLU:HB2	1:D:303:LYS:HE3	2.00	0.42
1:D:427:ASP:OD1	1:D:428:GLY:N	2.53	0.42
1:D:444:ARG:HD2	1:D:449:ARG:HB2	2.01	0.42
1:F:135:LEU:CD1	1:F:137:ASP:HB3	2.48	0.42
1:A:83:MET:HE3	1:A:514:ALA:HA	2.02	0.42
1:A:546:GLY:HA3	1:A:550:PRO:HG3	2.01	0.42
1:B:130:ILE:O	1:B:140:TRP:CZ3	2.73	0.42
1:B:479:ILE:HD11	1:B:559:PHE:HZ	1.84	0.42
1:E:377:ARG:HD3	1:E:421:LEU:CB	2.50	0.42
1:D:320:LEU:HD21	1:D:372:GLY:HA2	2.01	0.42
1:D:341:ASP:O	1:D:344:ASP:HB2	2.19	0.42
1:F:321:TRP:CZ3	1:F:325:ARG:NH2	2.88	0.42
1:F:366:ILE:HA	1:F:391:PHE:HE2	1.85	0.42
1:F:473:LYS:O	1:F:741:LYS:HG2	2.20	0.42
1:F:482:ARG:NH2	1:F:766:VAL:HG13	2.34	0.42
1:A:96:GLU:HB3	1:A:312:TYR:CE2	2.55	0.42
1:A:461:PRO:O	1:A:465:GLU:N	2.45	0.42
1:A:466:TYR:CE1	1:A:470:TYR:HB2	2.55	0.42
1:A:662:VAL:O	1:A:662:VAL:HG23	2.19	0.42
1:A:687:LEU:O	1:A:691:VAL:HG23	2.20	0.42

Continued on next page...

For Manuscript Review

Continued from previous page...

Atom-1	Atom-2	Interatomic distance (Å)	Clash overlap (Å)
1:B:162:LEU:HD22	1:B:168:HIS:HB2	2.02	0.42
1:B:300:ILE:HG21	1:B:308:VAL:CG2	2.50	0.42
1:C:304:PHE:CE2	1:C:309:PRO:HD3	2.55	0.42
1:E:142:ASP:OD1	1:E:502:ARG:NH1	2.52	0.42
1:E:193:TRP:CH2	1:E:451:LYS:HD3	2.53	0.42
1:E:545:PHE:HB3	1:E:593:ARG:CZ	2.49	0.42
1:D:91:LEU:HD23	1:D:91:LEU:HA	1.58	0.42
1:B:300:ILE:CG2	1:B:308:VAL:HG23	2.50	0.41
1:B:412:MET:HA	1:B:415:THR:OG1	2.20	0.41
1:B:559:PHE:CG	1:B:758:ILE:HG12	2.55	0.41
1:C:664:THR:HG1	1:C:665:GLY:H	1.62	0.41
1:C:675:GLN:HG2	1:C:720:ARG:CZ	2.50	0.41
1:D:556:THR:HG22	1:D:758:ILE:HD11	2.02	0.41
1:D:755:ASP:HB3	1:D:758:ILE:HD12	2.02	0.41
1:F:135:LEU:HB2	1:F:136:PRO:HD2	2.01	0.41
1:C:513:LYS:HD3	1:C:513:LYS:HA	1.90	0.41
1:E:671:TRP:HH2	1:F:656:ILE:HD11	1.85	0.41
1:D:104:TYR:HD2	1:D:153:MET:HG2	1.81	0.41
1:D:458:LYS:HE2	1:D:486:ASP:O	2.20	0.41
1:F:376:LEU:HD23	1:F:376:LEU:HA	1.85	0.41
1:F:529:GLU:HG2	1:F:558:TYR:OH	2.20	0.41
1:A:153:MET:HE2	1:A:202:LEU:HD13	2.03	0.41
1:A:690:ARG:HG3	1:A:691:VAL:N	2.35	0.41
1:B:690:ARG:HB2	1:B:706:ILE:HG21	2.02	0.41
1:B:766:VAL:O	1:B:767:ILE:HG22	2.20	0.41
1:C:348:ARG:HG2	1:C:348:ARG:HH11	1.85	0.41
1:C:482:ARG:HG2	1:C:761:VAL:HA	2.01	0.41
1:C:692:LEU:HA	1:C:695:GLU:HG3	2.01	0.41
1:E:444:ARG:HE	1:E:444:ARG:HB2	1.35	0.41
1:D:135:LEU:HD11	1:D:139:SER:HB2	2.02	0.41
1:D:224:LYS:HB3	1:D:224:LYS:HE3	1.87	0.41
1:D:291:GLN:O	1:D:295:GLU:HG3	2.21	0.41
1:A:281:THR:HB	1:A:297:LEU:HD21	2.01	0.41
1:A:754:ILE:O	1:A:758:ILE:HG13	2.20	0.41
1:C:320:LEU:CD2	1:C:372:GLY:HA2	2.49	0.41
1:D:377:ARG:HD3	1:D:421:LEU:HB3	2.02	0.41
1:F:470:TYR:N	1:F:470:TYR:CD1	2.89	0.41
1:F:731:LEU:HD23	1:F:731:LEU:HA	1.71	0.41
1:A:133:ASN:OD1	1:A:133:ASN:N	2.52	0.41
1:A:478:ARG:HD2	1:A:478:ARG:HA	1.52	0.41
1:D:173:GLU:O	1:D:175:GLY:N	2.54	0.41

Continued on next page...

For Manuscript Review

Continued from previous page...

Atom-1	Atom-2	Interatomic distance (Å)	Clash overlap (Å)
1:D:194:MET:HG3	1:D:198:PHE:CD2	2.47	0.41
1:D:403:GLU:HG2	1:D:405:ASN:HD21	1.84	0.41
1:F:80:THR:HG23	1:F:517:ALA:HB1	2.02	0.41
1:F:392:GLU:HB2	1:F:397:PHE:CE1	2.56	0.41
1:F:456:ILE:HD12	1:F:456:ILE:HA	1.93	0.41
1:A:135:LEU:HD11	1:A:143:ALA:HB2	2.01	0.41
1:A:621:LEU:HD12	1:A:621:LEU:HA	1.83	0.41
1:E:375:LEU:HD23	1:E:375:LEU:HA	1.83	0.41
1:E:385:PRO:CB	1:E:430:LEU:HD13	2.50	0.41
1:F:263:LYS:HE2	1:F:263:LYS:HB3	1.64	0.41
1:F:514:ALA:O	1:F:517:ALA:HB3	2.20	0.41
1:B:268:ARG:HH11	1:B:293:CYS:HB2	1.85	0.41
1:B:285:PHE:HA	1:B:288:THR:O	2.21	0.41
1:B:478:ARG:HD2	1:B:478:ARG:HA	1.72	0.41
1:C:331:ILE:HG21	1:C:331:ILE:HD13	1.83	0.41
1:E:328:ARG:HB3	1:E:484:TYR:CD2	2.55	0.41
1:E:614:ASP:HB3	1:E:617:TYR:HB2	2.01	0.41
1:D:325:ARG:CG	1:D:506:PHE:CE1	3.03	0.41
1:D:377:ARG:NH1	1:D:383:VAL:HG23	2.35	0.41
1:D:445:ARG:HH11	1:D:465:GLU:HG3	1.85	0.41
1:D:472:TRP:O	1:D:741:LYS:HE3	2.20	0.41
1:C:196:VAL:HG22	1:C:497:GLY:O	2.20	0.41
1:C:730:ARG:O	1:C:734:GLU:HG3	2.20	0.41
1:D:321:TRP:HZ3	1:D:325:ARG:NH2	2.18	0.41
1:D:664:THR:HG23	1:D:670:LEU:HD13	2.02	0.41
1:A:169:THR:HA	1:A:172:CYS:CB	2.49	0.41
1:C:223:LEU:O	1:C:225:ALA:N	2.53	0.41
1:C:352:PRO:O	1:C:387:VAL:HG23	2.20	0.41
1:C:377:ARG:HD3	1:C:421:LEU:HB3	2.02	0.41
1:C:423:PHE:CZ	1:C:719:ARG:HA	2.56	0.41
1:E:329:LEU:HA	1:E:329:LEU:HD23	1.87	0.41
1:E:419:SER:HA	1:E:430:LEU:HG	2.02	0.41
1:E:478:ARG:HD2	1:E:478:ARG:HA	1.58	0.41
1:E:533:LEU:HD22	1:E:554:LEU:HD11	2.02	0.41
1:D:292:LYS:HA	1:D:292:LYS:HD2	1.83	0.41
1:D:297:LEU:HD23	1:D:297:LEU:HA	1.78	0.41
1:D:535:ARG:HG2	1:D:539:ARG:HD3	2.02	0.41
1:F:262:GLU:HG2	1:F:263:LYS:N	2.36	0.41
1:F:313:PRO:HG2	1:F:358:THR:OG1	2.21	0.41
1:F:754:ILE:O	1:F:758:ILE:HG13	2.21	0.41
1:A:206:LEU:HD23	1:A:210:LYS:HE2	2.02	0.41

Continued on next page...

For Manuscript Review

Continued from previous page...

Atom-1	Atom-2	Interatomic distance (Å)	Clash overlap (Å)
1:A:429:VAL:HG12	1:A:432:ARG:HH12	1.85	0.41
1:A:628:TRP:HE1	1:A:646:THR:HG23	1.85	0.41
1:B:320:LEU:HD23	1:B:320:LEU:HA	1.89	0.41
1:B:489:GLY:HA3	1:B:493:ASP:OD1	2.21	0.41
1:B:542:LEU:HD12	1:B:542:LEU:HA	1.84	0.41
1:C:714:MET:HE2	1:C:714:MET:HB2	1.96	0.41
1:E:320:LEU:HA	1:E:320:LEU:HD23	1.85	0.41
1:E:344:ASP:HB3	1:E:348:ARG:HH22	1.85	0.41
1:D:335:PHE:HB3	1:D:338:GLU:CG	2.51	0.41
1:A:83:MET:HG2	1:A:510:LEU:HD11	2.03	0.41
1:A:168:HIS:O	1:A:172:CYS:HB2	2.21	0.41
1:A:292:LYS:H	1:A:292:LYS:HG2	1.56	0.41
1:A:494:VAL:HG22	1:A:502:ARG:NH2	2.36	0.41
1:B:535:ARG:NH1	1:D:340:GLU:OE1	2.51	0.40
1:E:540:SER:O	1:E:542:LEU:HD12	2.21	0.40
1:D:187:ALA:HB2	1:D:222:ALA:HB2	2.02	0.40
1:D:676:LEU:HA	1:D:676:LEU:HD23	1.69	0.40
1:F:343:LEU:HD23	1:F:346:ILE:HG13	2.03	0.40
1:F:676:LEU:HA	1:F:676:LEU:HD23	1.75	0.40
1:A:302:LYS:HG3	1:A:303:LYS:H	1.85	0.40
1:A:346:ILE:HG22	1:A:376:LEU:HD11	2.02	0.40
1:A:374:ARG:HB2	1:A:418:ALA:HB2	2.04	0.40
1:B:88:ARG:HG2	1:B:335:PHE:CE1	2.56	0.40
1:B:140:TRP:CD1	1:B:155:THR:HA	2.57	0.40
1:B:720:ARG:HH12	1:A:627:GLN:CD	2.24	0.40
1:C:422:LYS:H	1:C:422:LYS:HG2	1.64	0.40
1:C:678:GLN:HG2	1:D:678:GLN:HG2	2.04	0.40
1:E:131:VAL:HG23	1:E:132:GLN:HG3	2.02	0.40
1:E:297:LEU:HD23	1:E:297:LEU:HA	1.75	0.40
1:F:343:LEU:HD23	1:F:343:LEU:HA	1.61	0.40
1:A:165:TRP:O	1:A:167:ILE:HG23	2.21	0.40
1:A:206:LEU:HD11	1:A:218:TYR:HB3	2.02	0.40
1:A:312:TYR:CD2	1:A:313:PRO:HA	2.56	0.40
1:A:613:PHE:CE1	1:A:617:TYR:CD2	3.09	0.40
1:B:173:GLU:HA	1:B:176:LEU:HB3	2.03	0.40
1:B:195:LEU:HD11	1:B:498:LYS:O	2.20	0.40
1:B:495:TRP:CD1	1:B:503:MET:HG2	2.57	0.40
1:B:566:PHE:CD1	1:B:566:PHE:N	2.87	0.40
1:C:156:LEU:HD11	1:C:205:LEU:HD21	2.03	0.40
1:C:325:ARG:HA	1:C:325:ARG:HD3	1.70	0.40
1:E:412:MET:HB2	1:E:441:LEU:HD21	2.04	0.40

Continued on next page...

For Manuscript Review

Continued from previous page...

Atom-1	Atom-2	Interatomic distance (Å)	Clash overlap (Å)
1:E:687:LEU:HD23	1:E:687:LEU:HA	1.92	0.40
1:D:97:GLY:HA2	1:D:318:GLU:OE1	2.21	0.40
1:D:728:ILE:HG21	1:D:728:ILE:HD13	1.85	0.40
1:F:755:ASP:HA	1:F:758:ILE:HB	2.03	0.40
1:A:105:ASP:OD2	1:A:278:PRO:HD2	2.22	0.40
1:B:376:LEU:HD23	1:B:376:LEU:HA	1.71	0.40
1:B:423:PHE:CE1	1:B:719:ARG:HA	2.56	0.40
1:B:650:LEU:HA	1:B:650:LEU:HD23	1.87	0.40
1:C:482:ARG:HB2	1:C:519:PHE:CZ	2.56	0.40
1:E:190:GLU:HG3	1:E:193:TRP:CE3	2.54	0.40
1:E:650:LEU:HD23	1:E:650:LEU:HA	1.59	0.40
1:D:312:TYR:CG	1:D:313:PRO:HA	2.56	0.40
1:D:664:THR:CG2	1:D:670:LEU:HD13	2.51	0.40
1:F:422:LYS:HB3	1:F:422:LYS:HE2	1.76	0.40
1:F:683:ILE:O	1:F:687:LEU:HB2	2.20	0.40
1:A:162:LEU:HA	1:A:162:LEU:HD23	1.81	0.40
1:A:445:ARG:HA	1:A:450:MET:CE	2.52	0.40
1:A:574:ARG:O	1:A:577:TRP:HB3	2.22	0.40
1:A:642:ILE:H	1:A:642:ILE:HG13	1.81	0.40
1:B:206:LEU:HD11	1:B:223:LEU:HD21	2.02	0.40
1:B:321:TRP:O	1:B:325:ARG:HG2	2.22	0.40
1:B:389:LYS:HB3	1:B:429:VAL:CG1	2.49	0.40
1:B:698:GLU:O	1:B:699:SER:HB2	2.22	0.40
1:C:239:ARG:HA	1:C:239:ARG:HD3	1.78	0.40
1:C:508:ASN:OD1	1:C:509:ASP:N	2.55	0.40
1:D:321:TRP:CZ3	1:D:325:ARG:NH2	2.90	0.40
1:D:484:TYR:HA	1:D:487:GLN:HG3	2.04	0.40

There are no symmetry-related clashes.

5.3 Torsion angles (i)

5.3.1 Protein backbone (i)

In the following table, the Percentiles column shows the percent Ramachandran outliers of the chain as a percentile score with respect to all X-ray entries followed by that with respect to entries of similar resolution.

The Analysed column shows the number of residues for which the backbone conformation was analysed, and the total number of residues.

For Manuscript Review

Mol	Chain	Analysed	Favoured	Allowed	Outliers	Percentiles	
1	A	687/775 (89%)	621 (90%)	56 (8%)	10 (2%)	10	45
1	B	658/775 (85%)	611 (93%)	37 (6%)	10 (2%)	10	45
1	C	635/775 (82%)	571 (90%)	52 (8%)	12 (2%)	8	40
1	D	686/775 (88%)	637 (93%)	41 (6%)	8 (1%)	13	50
1	E	686/775 (88%)	630 (92%)	49 (7%)	7 (1%)	15	54
1	F	671/775 (87%)	605 (90%)	57 (8%)	9 (1%)	12	48
All	All	4023/4650 (86%)	3675 (91%)	292 (7%)	56 (1%)	11	46

All (56) Ramachandran outliers are listed below:

Mol	Chain	Res	Type
1	B	403	GLU
1	B	607	LEU
1	B	697	GLY
1	C	183	MET
1	C	664	THR
1	C	697	GLY
1	F	218	TYR
1	F	258	ASP
1	A	99	ILE
1	A	694	GLN
1	A	698	GLU
1	B	606	GLU
1	B	723	GLN
1	C	174	ARG
1	C	224	LYS
1	C	723	GLN
1	E	548	THR
1	E	697	GLY
1	D	174	ARG
1	D	658	GLY
1	D	699	SER
1	F	117	GLY
1	F	174	ARG
1	F	404	SER
1	F	663	LEU
1	F	724	GLY
1	A	394	ASP
1	A	696	ASN
1	B	539	ARG
1	B	594	ILE

Continued on next page...

For Manuscript Review

Continued from previous page...

Mol	Chain	Res	Type
1	B	699	SER
1	A	191	GLU
1	A	213	ASP
1	A	238	PRO
1	B	458	LYS
1	C	725	CYS
1	E	230	ARG
1	E	699	SER
1	D	548	THR
1	A	450	MET
1	B	217	PRO
1	C	699	SER
1	E	696	ASN
1	D	111	LEU
1	F	402	GLY
1	C	125	SER
1	C	550	PRO
1	E	111	LEU
1	D	175	GLY
1	D	663	LEU
1	A	699	SER
1	E	118	GLY
1	D	406	PRO
1	C	175	GLY
1	F	175	GLY
1	C	138	GLY

5.3.2 Protein sidechains (i)

In the following table, the Percentiles column shows the percent sidechain outliers of the chain as a percentile score with respect to all X-ray entries followed by that with respect to entries of similar resolution.

The Analysed column shows the number of residues for which the sidechain conformation was analysed, and the total number of residues.

Mol	Chain	Analysed	Rotameric	Outliers	Percentiles	
1	A	598/672 (89%)	571 (96%)	27 (4%)	27	61
1	B	582/672 (87%)	563 (97%)	19 (3%)	38	68
1	C	565/672 (84%)	547 (97%)	18 (3%)	39	69
1	D	597/672 (89%)	580 (97%)	17 (3%)	43	72

Continued on next page...

For Manuscript Review

Continued from previous page...

Mol	Chain	Analysed	Rotameric	Outliers	Percentiles	
1	E	597/672 (89%)	568 (95%)	29 (5%)	25	59
1	F	589/672 (88%)	569 (97%)	20 (3%)	37	68
All	All	3528/4032 (88%)	3398 (96%)	130 (4%)	34	65

All (130) residues with a non-rotameric sidechain are listed below:

Mol	Chain	Res	Type
1	B	102	SER
1	B	164	SER
1	B	165	TRP
1	B	213	ASP
1	B	214	LEU
1	B	227	TYR
1	B	263	LYS
1	B	314	LEU
1	B	315	ASP
1	B	333	ARG
1	B	420	GLN
1	B	444	ARG
1	B	478	ARG
1	B	529	GLU
1	B	537	TYR
1	B	604	LEU
1	B	618	SER
1	B	711	ASP
1	B	726	SER
1	C	146	PHE
1	C	193	TRP
1	C	204	SER
1	C	232	ARG
1	C	259	LEU
1	C	271	ASP
1	C	277	SER
1	C	360	ASN
1	C	478	ARG
1	C	506	PHE
1	C	512	LEU
1	C	537	TYR
1	C	545	PHE
1	C	591	PHE
1	C	599	ASN

Continued on next page...

For Manuscript Review

Continued from previous page...

Mol	Chain	Res	Type
1	C	641	SER
1	C	690	ARG
1	C	711	ASP
1	E	102	SER
1	E	114	ARG
1	E	119	ASP
1	E	145	PHE
1	E	170	ASP
1	E	210	LYS
1	E	219	ASP
1	E	220	GLU
1	E	227	TYR
1	E	230	ARG
1	E	232	ARG
1	E	239	ARG
1	E	261	TRP
1	E	315	ASP
1	E	333	ARG
1	E	360	ASN
1	E	440	PHE
1	E	444	ARG
1	E	453	LYS
1	E	478	ARG
1	E	512	LEU
1	E	541	ASN
1	E	545	PHE
1	E	549	ASP
1	E	592	ARG
1	E	599	ASN
1	E	615	ASP
1	E	641	SER
1	E	643	GLU
1	D	102	SER
1	D	116	ASP
1	D	144	SER
1	D	190	GLU
1	D	227	TYR
1	D	239	ARG
1	D	259	LEU
1	D	315	ASP
1	D	478	ARG
1	D	487	GLN

Continued on next page...

For Manuscript Review

Continued from previous page...

Mol	Chain	Res	Type
1	D	506	PHE
1	D	512	LEU
1	D	537	TYR
1	D	545	PHE
1	D	604	LEU
1	D	690	ARG
1	D	730	ARG
1	F	165	TRP
1	F	166	ASN
1	F	220	GLU
1	F	227	TYR
1	F	256	MET
1	F	271	ASP
1	F	277	SER
1	F	287	GLN
1	F	317	TYR
1	F	360	ASN
1	F	394	ASP
1	F	417	ARG
1	F	445	ARG
1	F	468	MET
1	F	613	PHE
1	F	618	SER
1	F	622	ARG
1	F	666	GLN
1	F	726	SER
1	F	730	ARG
1	A	133	ASN
1	A	192	ASP
1	A	193	TRP
1	A	227	TYR
1	A	239	ARG
1	A	245	MET
1	A	259	LEU
1	A	280	SER
1	A	291	GLN
1	A	293	CYS
1	A	305	ASN
1	A	337	SER
1	A	444	ARG
1	A	447	SER
1	A	478	ARG

Continued on next page...

For Manuscript Review

Continued from previous page...

Mol	Chain	Res	Type
1	A	539	ARG
1	A	545	PHE
1	A	549	ASP
1	A	569	ASN
1	A	591	PHE
1	A	599	ASN
1	A	609	SER
1	A	613	PHE
1	A	622	ARG
1	A	632	TRP
1	A	690	ARG
1	A	723	GLN

Sometimes sidechains can be flipped to improve hydrogen bonding and reduce clashes. All (5) such sidechains are listed below:

Mol	Chain	Res	Type
1	B	420	GLN
1	C	251	HIS
1	E	420	GLN
1	D	420	GLN
1	F	420	GLN

5.3.3 RNA (i)

There are no RNA molecules in this entry.

5.4 Non-standard residues in protein, DNA, RNA chains (i)

There are no non-standard protein/DNA/RNA residues in this entry.

5.5 Carbohydrates (i)

There are no monosaccharides in this entry.

5.6 Ligand geometry (i)

There are no ligands in this entry.

For Manuscript Review

5.7 Other polymers (i)

There are no such residues in this entry.

5.8 Polymer linkage issues (i)

There are no chain breaks in this entry.

For Manuscript Review

6 Fit of model and data (i)

6.1 Protein, DNA and RNA chains (i)

In the following table, the column labelled '#RSRZ> 2' contains the number (and percentage) of RSRZ outliers, followed by percent RSRZ outliers for the chain as percentile scores relative to all X-ray entries and entries of similar resolution. The OWAB column contains the minimum, median, 95th percentile and maximum values of the occupancy-weighted average B-factor per residue. The column labelled 'Q< 0.9' lists the number of (and percentage) of residues with an average occupancy less than 0.9.

Mol	Chain	Analysed	<RSRZ>	#RSRZ>2			OWAB(Å^2)	Q<0.9
1	A	689/775 (88%)	0.33	3 (0%)	92	90	32, 56, 100, 127	0
1	B	668/775 (86%)	0.56	39 (5%)	23	20	34, 76, 118, 132	0
1	C	648/775 (83%)	0.72	69 (10%)	6	7	39, 85, 150, 177	0
1	D	688/775 (88%)	0.67	65 (9%)	8	9	41, 84, 138, 160	0
1	E	688/775 (88%)	0.31	3 (0%)	92	90	41, 67, 111, 131	0
1	F	677/775 (87%)	0.75	68 (10%)	7	8	50, 95, 145, 169	0
All	All	4058/4650 (87%)	0.55	247 (6%)	21	19	32, 76, 135, 177	0

All (247) RSRZ outliers are listed below:

Mol	Chain	Res	Type	RSRZ
1	C	496	ILE	5.6
1	D	454	TRP	5.2
1	F	192	ASP	5.1
1	C	200	ILE	5.1
1	C	104	TYR	4.6
1	C	179	ILE	4.6
1	C	497	GLY	4.6
1	C	105	ASP	4.5
1	C	180	GLN	4.2
1	D	160	VAL	4.2
1	F	454	TRP	4.0
1	C	159	VAL	3.9
1	C	357	HIS	3.9
1	F	193	TRP	3.8
1	F	176	LEU	3.8
1	C	106	THR	3.8
1	F	500	LEU	3.7
1	D	127	ILE	3.7
1	C	112	LEU	3.6

Continued on next page...

For Manuscript Review

Continued from previous page...

Mol	Chain	Res	Type	RSRZ
1	C	243	HIS	3.6
1	F	179	ILE	3.6
1	D	221	PRO	3.6
1	C	155	THR	3.6
1	B	498	LYS	3.6
1	C	300	ILE	3.6
1	C	152	ILE	3.5
1	C	500	LEU	3.4
1	C	454	TRP	3.4
1	D	253	LEU	3.4
1	F	617	TYR	3.3
1	B	256	MET	3.3
1	C	253	LEU	3.3
1	B	216	ILE	3.3
1	C	256	MET	3.3
1	C	296	TYR	3.2
1	D	104	TYR	3.2
1	F	172	CYS	3.2
1	C	111	LEU	3.2
1	D	261	TRP	3.2
1	D	107	SER	3.2
1	C	279	ALA	3.2
1	D	108	LEU	3.2
1	F	138	GLY	3.2
1	D	126	THR	3.1
1	C	278	PRO	3.1
1	D	208	MET	3.1
1	D	252	SER	3.1
1	C	314	LEU	3.1
1	C	313	PRO	3.0
1	D	222	ALA	3.0
1	F	307	GLY	3.0
1	D	180	GLN	3.0
1	B	198	PHE	3.0
1	C	252	SER	3.0
1	D	307	GLY	3.0
1	D	109	VAL	3.0
1	D	159	VAL	3.0
1	D	141	GLY	3.0
1	F	157	ALA	3.0
1	C	251	HIS	3.0
1	C	131	VAL	3.0

Continued on next page...

For Manuscript Review

Continued from previous page...

Mol	Chain	Res	Type	RSRZ
1	B	104	TYR	2.9
1	D	297	LEU	2.9
1	F	450	MET	2.9
1	C	165	TRP	2.9
1	C	153	MET	2.9
1	C	99	ILE	2.9
1	F	314	LEU	2.9
1	F	761	VAL	2.8
1	D	495	TRP	2.8
1	C	504	THR	2.8
1	C	156	LEU	2.8
1	D	200	ILE	2.8
1	D	182	ASN	2.8
1	C	108	LEU	2.8
1	F	103	ALA	2.8
1	D	400	LEU	2.8
1	B	251	HIS	2.8
1	C	148	MET	2.7
1	C	311	ILE	2.7
1	C	485	LEU	2.7
1	D	455	ALA	2.7
1	B	286	GLN	2.7
1	C	371	MET	2.7
1	F	153	MET	2.7
1	C	181	GLU	2.7
1	F	563	ALA	2.7
1	F	91	LEU	2.7
1	B	148	MET	2.7
1	C	309	PRO	2.7
1	D	453	LYS	2.7
1	B	215	ASP	2.7
1	B	156	LEU	2.7
1	D	157	ALA	2.7
1	D	215	ASP	2.6
1	D	238	PRO	2.6
1	F	616	ALA	2.6
1	D	165	TRP	2.6
1	F	308	VAL	2.6
1	F	99	ILE	2.6
1	A	239	ARG	2.6
1	C	283	THR	2.6
1	B	500	LEU	2.6

Continued on next page...

For Manuscript Review

Continued from previous page...

Mol	Chain	Res	Type	RSRZ
1	C	103	ALA	2.6
1	D	496	ILE	2.6
1	C	205	LEU	2.6
1	F	420	GLN	2.6
1	F	506	PHE	2.6
1	F	178	PHE	2.6
1	F	398	PHE	2.6
1	B	311	ILE	2.6
1	F	300	ILE	2.6
1	D	460	ILE	2.6
1	F	123	PHE	2.5
1	B	217	PRO	2.5
1	C	127	ILE	2.5
1	D	174	ARG	2.5
1	C	400	LEU	2.5
1	C	310	CYS	2.5
1	F	183	MET	2.5
1	B	280	SER	2.5
1	D	179	ILE	2.5
1	C	174	ARG	2.5
1	C	162	LEU	2.5
1	D	278	PRO	2.5
1	D	309	PRO	2.5
1	C	505	LEU	2.5
1	C	498	LYS	2.5
1	F	503	MET	2.5
1	D	146	PHE	2.5
1	F	317	TYR	2.5
1	F	498	LYS	2.5
1	D	131	VAL	2.4
1	F	108	LEU	2.4
1	B	178	PHE	2.4
1	B	318	GLU	2.4
1	D	300	ILE	2.4
1	B	205	LEU	2.4
1	F	400	LEU	2.4
1	D	254	GLU	2.4
1	C	208	MET	2.4
1	C	318	GLU	2.4
1	F	309	PRO	2.4
1	D	321	TRP	2.4
1	D	398	PHE	2.4

Continued on next page...

For Manuscript Review

Continued from previous page...

Mol	Chain	Res	Type	RSRZ
1	C	149	GLY	2.4
1	B	397	PHE	2.4
1	D	103	ALA	2.4
1	F	184	TRP	2.4
1	B	180	GLN	2.4
1	B	485	LEU	2.4
1	C	317	TYR	2.4
1	F	502	ARG	2.4
1	F	222	ALA	2.4
1	F	762	ILE	2.4
1	C	399	CYS	2.4
1	B	516	LYS	2.4
1	B	208	MET	2.4
1	D	274	PHE	2.4
1	F	186	LEU	2.4
1	D	225	ALA	2.3
1	D	161	ALA	2.3
1	F	412	MET	2.3
1	F	764	GLN	2.3
1	C	274	PHE	2.3
1	F	111	LEU	2.3
1	A	274	PHE	2.3
1	B	261	TRP	2.3
1	F	401	HIS	2.3
1	B	300	ILE	2.3
1	C	150	ASP	2.3
1	B	297	LEU	2.3
1	F	451	LYS	2.3
1	B	252	SER	2.3
1	F	214	LEU	2.3
1	D	357	HIS	2.3
1	F	104	TYR	2.3
1	D	149	GLY	2.3
1	B	279	ALA	2.3
1	C	282	ALA	2.3
1	B	153	MET	2.2
1	F	215	ASP	2.2
1	D	99	ILE	2.2
1	E	283	THR	2.2
1	B	488	TYR	2.2
1	C	280	SER	2.2
1	C	506	PHE	2.2

Continued on next page...

For Manuscript Review

Continued from previous page...

Mol	Chain	Res	Type	RSRZ
1	D	762	ILE	2.2
1	F	143	ALA	2.2
1	C	161	ALA	2.2
1	D	153	MET	2.2
1	C	178	PHE	2.2
1	F	281	THR	2.2
1	C	242	LEU	2.2
1	C	197	GLY	2.2
1	B	309	PRO	2.2
1	F	109	VAL	2.2
1	B	199	GLU	2.2
1	B	141	GLY	2.2
1	F	593	ARG	2.2
1	D	317	TYR	2.1
1	D	156	LEU	2.1
1	D	178	PHE	2.1
1	F	113	LYS	2.1
1	C	271	ASP	2.1
1	F	320	LEU	2.1
1	B	242	LEU	2.1
1	C	261	TRP	2.1
1	C	281	THR	2.1
1	D	293	CYS	2.1
1	A	99	ILE	2.1
1	F	142	ASP	2.1
1	E	698	GLU	2.1
1	F	246	PRO	2.1
1	B	254	GLU	2.1
1	F	365	ASP	2.1
1	B	314	LEU	2.1
1	D	500	LEU	2.1
1	F	149	GLY	2.1
1	C	262	GLU	2.1
1	D	331	ILE	2.1
1	F	329	LEU	2.1
1	F	397	PHE	2.1
1	D	173	GLU	2.1
1	D	498	LYS	2.1
1	F	311	ILE	2.1
1	F	106	THR	2.1
1	D	313	PRO	2.1
1	F	316	VAL	2.1

Continued on next page...

For Manuscript Review

Continued from previous page...

Mol	Chain	Res	Type	RSRZ
1	D	142	ASP	2.1
1	B	157	ALA	2.0
1	D	452	ASP	2.0
1	D	111	LEU	2.0
1	B	450	MET	2.0
1	F	452	ASP	2.0
1	C	397	PHE	2.0
1	D	281	THR	2.0
1	E	213	ASP	2.0
1	F	518	ASP	2.0
1	F	180	GLN	2.0
1	F	199	GLU	2.0
1	F	318	GLU	2.0
1	D	408	SER	2.0
1	B	103	ALA	2.0
1	B	179	ILE	2.0
1	F	463	GLU	2.0
1	D	195	LEU	2.0

6.2 Non-standard residues in protein, DNA, RNA chains (i)

There are no non-standard protein/DNA/RNA residues in this entry.

6.3 Carbohydrates (i)

There are no monosaccharides in this entry.

6.4 Ligands (i)

There are no ligands in this entry.

6.5 Other polymers (i)

There are no such residues in this entry.

For Manuscript Review
