# Supplementary material for: Structural and functional investigations of syn-copalyl diphosphate synthase from Oryza sativa
Source: Commun Chem. 2023 Nov 6;6:240. doi: 10.1038/s42004-023-01042-w (PMC10628199; doi:10.1038/s42004-023-01042-w)
Supplement: Supplementary file 7 — Supplementary Data 4 [file 42004_2023_1042_MOESM7_ESM.pdf]

# Full wwPDB EM Validation Report (i)

Feb 9, 2023 – 05:57 PM JST

| | | |
|---|---|---|
| PDB ID | : | 8I6P |
| EMDB ID | : | EMD-35202 |
| Title | : | The cryo-EM structure of OsCyc1 tetramer state |
| Deposited on | : | 2023-01-29 |
| Resolution | : | 3.50 Å(reported) |

**This wwPDB validation report is for manuscript review**

This is a Full wwPDB EM Validation Report.

This report is produced by the wwPDB biocuration pipeline after annotation of the structure.

We welcome your comments at *validation@mail.wwpdb.org*
A user guide is available at
https://www.wwpdb.org/validation/2017/EMValidationReportHelp
with specific help available everywhere you see the (i) symbol.

The types of validation reports are described at
http://www.wwpdb.org/validation/2017/FAQs#types.

For Manuscript Review

---

The following versions of software and data (see references (i)) were used in the production of this report:

| | | |
|---|---|---|
| EMDB validation analysis | : | 0.0.1.dev43 |
| MolProbity | : | 4.02b-467 |
| Percentile statistics | : | 20191225.v01 (using entries in the PDB archive December 25th 2019) |
| MapQ | : | 1.9.9 |
| Ideal geometry (proteins) | : | Engh & Huber (2001) |
| Ideal geometry (DNA, RNA) | : | Parkinson et al. (1996) |
| Validation Pipeline (wwPDB-VP) | : | 2.32.1 |

# 1 Overall quality at a glance (i)

The following experimental techniques were used to determine the structure:
*ELECTRON MICROSCOPY*

The reported resolution of this entry is 3.50 Å.

Percentile scores (ranging between 0-100) for global validation metrics of the entry are shown in the following graphic. The table shows the number of entries on which the scores are based.

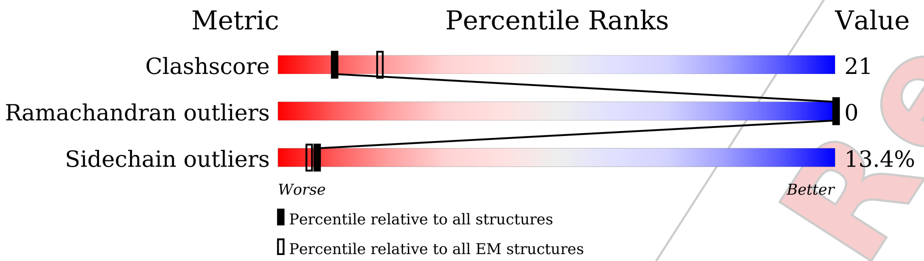


| Metric | Whole archive (#Entries) | EM structures (#Entries) |
|---|---|---|
| Clashscore | 158937 | 4297 |
| Ramachandran outliers | 154571 | 4023 |
| Sidechain outliers | 154315 | 3826 |

The table below summarises the geometric issues observed across the polymeric chains and their fit to the map. The red, orange, yellow and green segments of the bar indicate the fraction of residues that contain outliers for >=3, 2, 1 and 0 types of geometric quality criteria respectively. A grey segment represents the fraction of residues that are not modelled. The numeric value for each fraction is indicated below the corresponding segment, with a dot representing fractions <=5% The upper red bar (where present) indicates the fraction of residues that have poor fit to the EM map (all-atom inclusion < 40%). The numeric value is given above the bar.

| Mol | Chain | Length | Quality of chain |
|---|---|---|---|
| 1 | A | 775 | • 44% 40% 5% 11% |
| 1 | B | 775 | • 45% 39% 5% 11% |
| 1 | C | 775 | • 46% 38% • 11% |
| 1 | D | 775 | • 46% 38% • 11% |

For Manuscript Review

## 2 Entry composition (i)

There is only 1 type of molecule in this entry. The entry contains 22016 atoms, of which 0 are hydrogens and 0 are deuteriums.

In the tables below, the AltConf column contains the number of residues with at least one atom in alternate conformation and the Trace column contains the number of residues modelled with at most 2 atoms.

- Molecule 1 is a protein called Syn-copalyl diphosphate synthase, chloroplastic.

| Mol | Chain | Residues | Atoms | | | | | AltConf | Trace |
|---|---|---|---|---|---|---|---|---|---|
| | | | Total | C | N | O | S | | |
| 1 | B | 688 | 5504 | 3492 | 944 | 1033 | 35 | 0 | 0 |
| 1 | C | 688 | 5504 | 3492 | 944 | 1033 | 35 | 0 | 0 |
| 1 | D | 688 | 5504 | 3492 | 944 | 1033 | 35 | 0 | 0 |
| 1 | A | 688 | 5504 | 3492 | 944 | 1033 | 35 | 0 | 0 |

There are 32 discrepancies between the modelled and reference sequences:

| Chain | Residue | Modelled | Actual | Comment | Reference |
|---|---|---|---|---|---|
| B | 768 | GLU | - | expression tag | UNP Q0JF02 |
| B | 769 | PHE | - | expression tag | UNP Q0JF02 |
| B | 770 | HIS | - | expression tag | UNP Q0JF02 |
| B | 771 | HIS | - | expression tag | UNP Q0JF02 |
| B | 772 | HIS | - | expression tag | UNP Q0JF02 |
| B | 773 | HIS | - | expression tag | UNP Q0JF02 |
| B | 774 | HIS | - | expression tag | UNP Q0JF02 |
| B | 775 | HIS | - | expression tag | UNP Q0JF02 |
| C | 768 | GLU | - | expression tag | UNP Q0JF02 |
| C | 769 | PHE | - | expression tag | UNP Q0JF02 |
| C | 770 | HIS | - | expression tag | UNP Q0JF02 |
| C | 771 | HIS | - | expression tag | UNP Q0JF02 |
| C | 772 | HIS | - | expression tag | UNP Q0JF02 |
| C | 773 | HIS | - | expression tag | UNP Q0JF02 |
| C | 774 | HIS | - | expression tag | UNP Q0JF02 |
| C | 775 | HIS | - | expression tag | UNP Q0JF02 |
| D | 768 | GLU | - | expression tag | UNP Q0JF02 |
| D | 769 | PHE | - | expression tag | UNP Q0JF02 |
| D | 770 | HIS | - | expression tag | UNP Q0JF02 |
| D | 771 | HIS | - | expression tag | UNP Q0JF02 |
| D | 772 | HIS | - | expression tag | UNP Q0JF02 |
| D | 773 | HIS | - | expression tag | UNP Q0JF02 |

*Continued on next page...*

For Manuscript Review

*Continued from previous page...*

| Chain | Residue | Modelled | Actual | Comment | Reference |
|---|---|---|---|---|---|
| D | 774 | HIS | - | expression tag | UNP Q0JF02 |
| D | 775 | HIS | - | expression tag | UNP Q0JF02 |
| A | 768 | GLU | - | expression tag | UNP Q0JF02 |
| A | 769 | PHE | - | expression tag | UNP Q0JF02 |
| A | 770 | HIS | - | expression tag | UNP Q0JF02 |
| A | 771 | HIS | - | expression tag | UNP Q0JF02 |
| A | 772 | HIS | - | expression tag | UNP Q0JF02 |
| A | 773 | HIS | - | expression tag | UNP Q0JF02 |
| A | 774 | HIS | - | expression tag | UNP Q0JF02 |
| A | 775 | HIS | - | expression tag | UNP Q0JF02 |

For Manuscript Review

# 3 Residue-property plots (i)

These plots are drawn for all protein, RNA, DNA and oligosaccharide chains in the entry. The first graphic for a chain summarises the proportions of the various outlier classes displayed in the second graphic. The second graphic shows the sequence view annotated by issues in geometry and atom inclusion in map density. Residues are color-coded according to the number of geometric quality criteria for which they contain at least one outlier: green = 0, yellow = 1, orange = 2 and red = 3 or more. A red diamond above a residue indicates a poor fit to the EM map for this residue (all-atom inclusion < 40%). Stretches of 2 or more consecutive residues without any outlier are shown as a green connector. Residues present in the sample, but not in the model, are shown in grey.

- Molecule 1: Syn-copalyl diphosphate synthase, chloroplastic

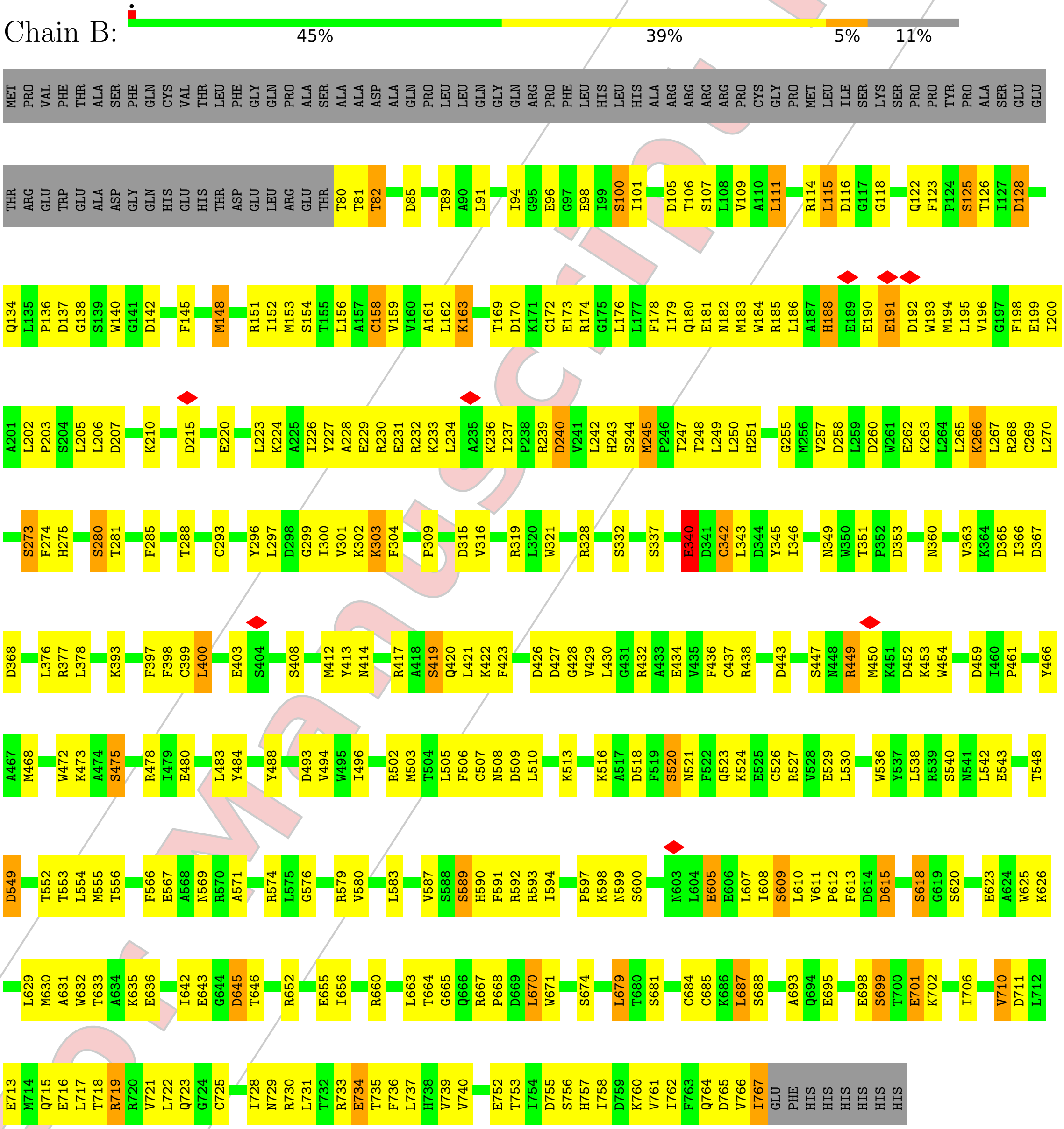


- Molecule 1: Syn-copalyl diphosphate synthase, chloroplastic

For Manuscript Review

Chain C:

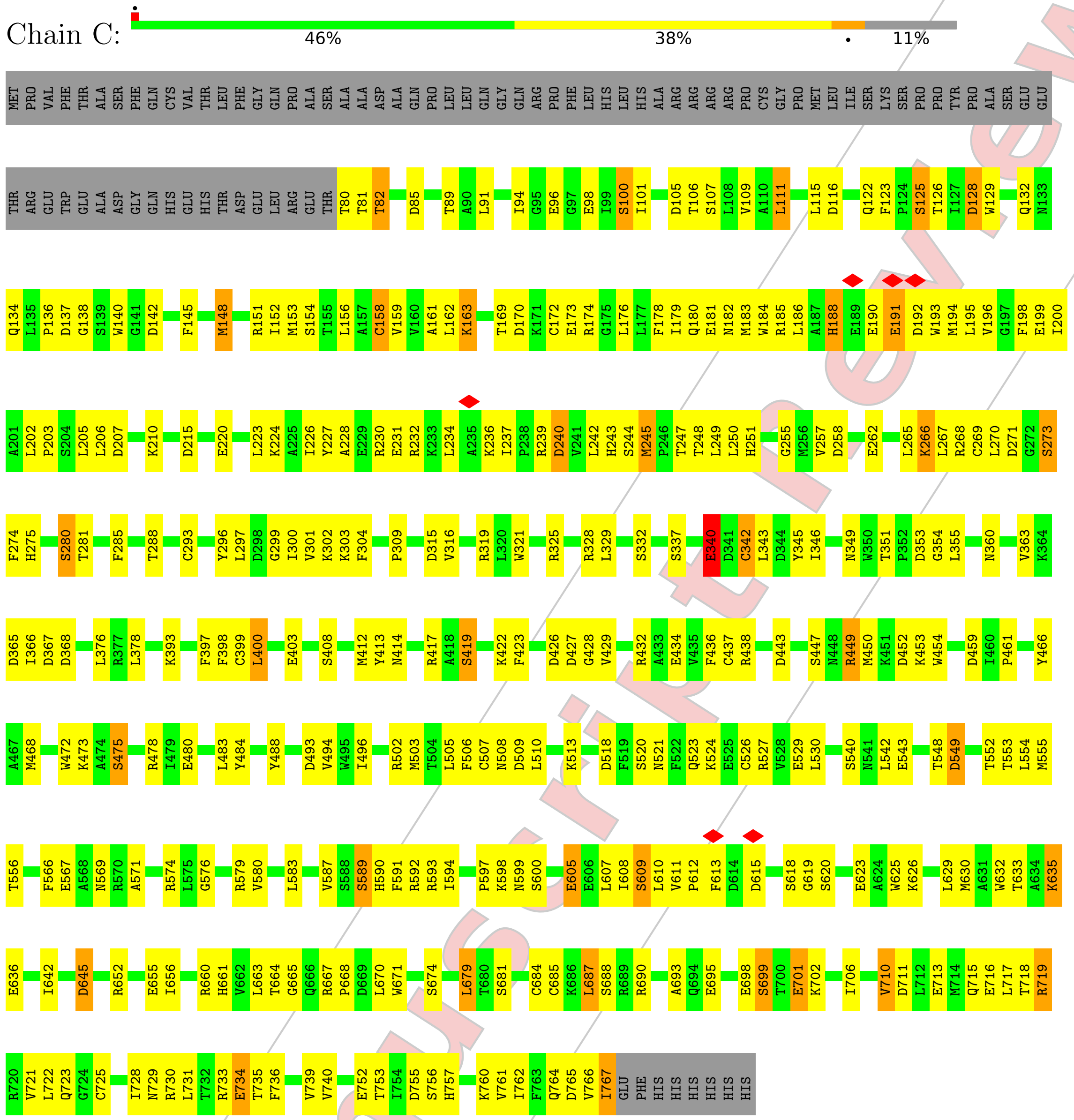


• Molecule 1: Syn-copalyl diphosphate synthase, chloroplastic

Chain D:

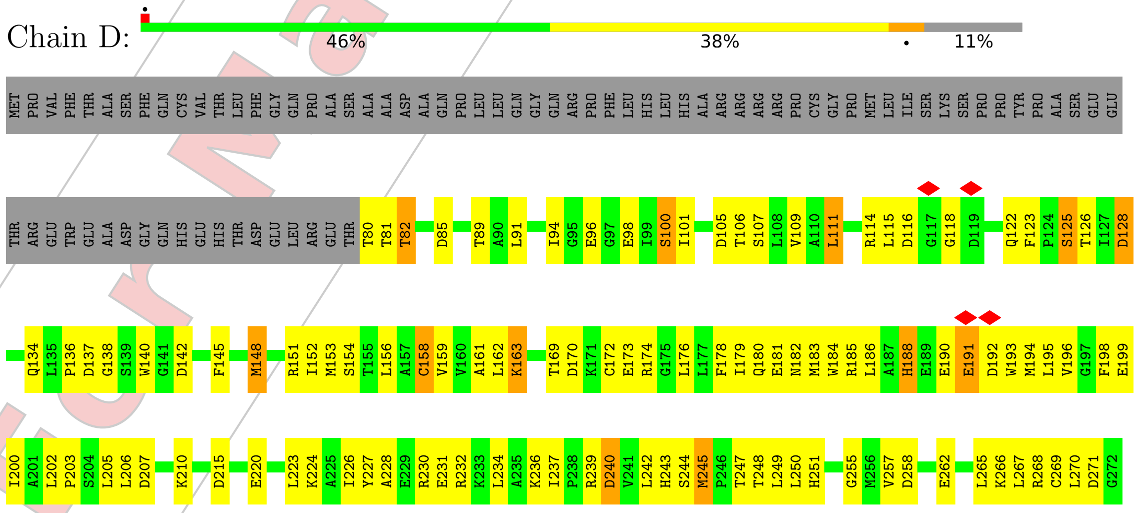


For Manuscript Review

S273 F274 H275 S280 T281 F285 T288 C293 Y296 L297 D298 G299 I300 V301 K302 K303 F304 P309 D315 V316 R319 L320 W321 R328 S332 S337 E340 D341 C342 L343 D344 Y345 I346 N349 W350 T351 P352 D353 N360 V363 K364 D365 I366 D367 D368

L376 R377 L378 K393 F397 F398 C399 L400 E403 S404 S408 M412 Y413 N414 R417 A418 S419 Q420 L421 K422 F423 D426 D427 G428 V429 L430 G431 R432 A433 E434 V435 F436 C437 R438 L441 Q442 D443 S447 N448 R449 M450 K451 D452 K453 W454 D459 I460 P461

Y466 A467 M468 W472 K473 A474 S475 R478 I479 E480 L483 Y484 Y488 D493 V494 W495 I496 R502 M503 T504 L505 F506 C507 N508 D509 L510 K513 K516 A517 D518 F519 S520 N521 F522 Q523 K524 E525 C526 R527 V528 E529 L530 W536 S540 N541 L542 E543 T548 D549

T552 T553 L554 M555 T556 F566 E567 A568 N569 R570 A571 R574 L575 G576 R579 V580 L583 V587 S588 S589 H590 F591 R592 R593 I594 P597 K598 N599 S600 E605 E606 L607 I608 S609 L610 V611 P612 F613 D614 D615 S618 G619 S620 E623 A624 W625 K626 L629 M630

A631 W632 T633 A634 K635 E636 D645 R652 E655 I656 R660 L663 T664 G665 Q666 R667 L670 W671 S674 L679 T680 S681 C684 C685 K686 L687 S688 A693 Q694 E695 E698 S699 T700 E701 K702 I706 V710 D711 L712 E713 M714 Q715 E716 L717 T718 R719 R720

V721 L722 Q723 G724 C725 I728 N729 R730 L731 T732 R733 E734 T735 F736 L737 H738 V739 V740 E752 D755 S756 H757 I758 D759 K760 V761 I762 F763 Q764 D765 V766 I767 GLU PHE HIS HIS HIS HIS HIS HIS

• Molecule 1: Syn-copalyl diphosphate synthase, chloroplastic

Chain A: 44% 40% 5% 11%

MET PRO VAL PHE THR ALA SER PHE GLN CYS VAL THR LEU PHE GLY GLN PRO ALA SER ALA ALA ASP ALA GLN PRO LEU LEU GLN GLY GLN ARG PRO PHE LEU HIS LEU HIS ALA ARG ARG ARG ARG PRO CYS GLY PRO MET LEU ILE SER LYS SER PRO PRO TYR PRO ALA SER GLU GLU

THR ARG GLU TRP GLU ALA ASP GLY GLN HIS GLU HIS THR ASP GLU LEU ARG GLU THR T80 T81 T82 D85 T89 A90 L91 I94 G95 E96 G97 E98 I99 S100 I101 D105 T106 S107 L108 V109 A110 L111 R114 L115 G118 Q122 F123 P124 S125 T126 I127 D128 W129

Q132 N133 Q134 L135 P136 D137 G138 S139 W140 G141 D142 F145 M148 R151 I152 M153 S154 T155 L156 A157 C158 V159 V160 A161 L162 K163 T169 D170 K171 C172 E173 R174 G175 L176 L177 F178 I179 Q180 E181 N182 M183 W184 R185 L186 A187 H188 E189 E190 E191 D192 W193 M194 L195 V196 G197 F198

E199 I200 A201 L202 P203 S204 L205 L206 D207 K210 D215 E220 L223 K224 A225 I226 Y227 A228 E229 R230 E231 R232 K233 L234 A235 K236 I237 P238 R239 D240 V241 L242 H243 S244 M245 P246 T247 T248 L249 L250 H251 E254 G255 M256 V257 D258 L259 D260 W261 E262 K263 L264 L265 K266 L267

R268 C269 L270 D271 G272 S273 F274 H275 S280 T281 F285 T288 C293 Y296 L297 D298 G299 I300 V301 K302 K303 F304 N305 P309 D315 V316 R319 L320 W321 R325 R328 L329 S332 S337 E338 I339 E340 D341 C342 L343 D344 Y345 I346 N349 W350 T351 P352

D353 G354 L355 N360 V363 K364 D365 I366 D367 D368 M371 L376 R377 L378 K393 K396 F397 F398 C399 L400 E403 S404 S407 S408 P411 M412 Y413 N414 T415 Y416 R417 A418 S419 Q420 L421 K422 F423 D426 D427 G428 V429 R432 A433 E434 V435 F436 C437 R438

D443 S447 N448 R449 M450 K451 D452 K453 W454 D459 I460 P461 Y466 A467 M468 W472 K473 A474 S475 R478 I479 E480 L483 Y484 L485 Y488 D493 V494 W495 I496 R502 M503 T504 L505 F506 C507 N508 D509 L510 K513 K516 A517 D518 F519 S520 N521 F522 Q523 K524

E525 C526 R527 V528 E529 L530 S540 N541 L542 E543 T548 D549 T552 T553 L554 M555 T556 S557 Y558 F566 E567 A568 N569 R570 A571 R574 L575 G576 R579 V580 L583 V587 S588 S589 H590 F591 R592 R593 I594 P597 K598 N599 E605 E606 L607 I608 S609 L610 V611 P612

For Manuscript Review

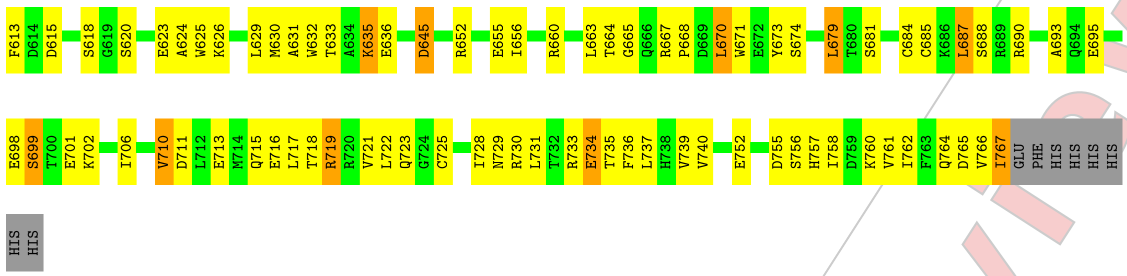


For Manuscript Review

# 4 Experimental information (i)

| Property | Value | Source |
|---|---|---|
| EM reconstruction method | SINGLE PARTICLE | Depositor |
| Imposed symmetry | POINT, Not provided | |
| Number of particles used | 149837 | Depositor |
| Resolution determination method | FSC 0.143 CUT-OFF | Depositor |
| CTF correction method | PHASE FLIPPING AND AMPLITUDE CORRECTION | Depositor |
| Microscope | FEI TITAN KRIOS | Depositor |
| Voltage (kV) | 300 | Depositor |
| Electron dose ($e^-/\text{Å}^2$) | 60 | Depositor |
| Minimum defocus (nm) | 1500 | Depositor |
| Maximum defocus (nm) | 2300 | Depositor |
| Magnification | Not provided | |
| Image detector | GATAN K3 (6k x 4k) | Depositor |
| Maximum map value | 0.065 | Depositor |
| Minimum map value | -0.036 | Depositor |
| Average map value | 0.000 | Depositor |
| Map value standard deviation | 0.004 | Depositor |
| Recommended contour level | 0.0137 | Depositor |
| Map size (Å) | 299.6, 299.6, 299.6 | wwPDB |
| Map dimensions | 280, 280, 280 | wwPDB |
| Map angles (°) | 90.0, 90.0, 90.0 | wwPDB |
| Pixel spacing (Å) | 1.07, 1.07, 1.07 | Depositor |

For Manuscript Review

# 5 Model quality (i)

## 5.1 Standard geometry (i)

The Z score for a bond length (or angle) is the number of standard deviations the observed value is removed from the expected value. A bond length (or angle) with $|Z| > 5$ is considered an outlier worth inspection. RMSZ is the root-mean-square of all Z scores of the bond lengths (or angles).

| Mol | Chain | Bond lengths | | Bond angles | |
|---|---|---|---|---|---|
| | | RMSZ | $\#|Z| > 5$ | RMSZ | $\#|Z| > 5$ |
| 1 | A | 0.34 | 0/5627 | 0.47 | 1/7615 (0.0%) |
| 1 | B | 0.34 | 0/5627 | 0.47 | 1/7615 (0.0%) |
| 1 | C | 0.34 | 0/5627 | 0.47 | 1/7615 (0.0%) |
| 1 | D | 0.34 | 0/5627 | 0.47 | 1/7615 (0.0%) |
| All | All | 0.34 | 0/22508 | 0.47 | 4/30460 (0.0%) |

There are no bond length outliers.

All (4) bond angle outliers are listed below:

| Mol | Chain | Res | Type | Atoms | Z | Observed($^o$) | Ideal($^o$) |
|---|---|---|---|---|---|---|---|
| 1 | B | 340 | GLU | CA-CB-CG | 5.32 | 125.10 | 113.40 |
| 1 | C | 340 | GLU | CA-CB-CG | 5.31 | 125.08 | 113.40 |
| 1 | A | 340 | GLU | CA-CB-CG | 5.31 | 125.08 | 113.40 |
| 1 | D | 340 | GLU | CA-CB-CG | 5.30 | 125.06 | 113.40 |

There are no chirality outliers.

There are no planarity outliers.

## 5.2 Too-close contacts (i)

In the following table, the Non-H and H(model) columns list the number of non-hydrogen atoms and hydrogen atoms in the chain respectively. The H(added) column lists the number of hydrogen atoms added and optimized by MolProbity. The Clashes column lists the number of clashes within the asymmetric unit, whereas Symm-Clashes lists symmetry-related clashes.

| Mol | Chain | Non-H | H(model) | H(added) | Clashes | Symm-Clashes |
|---|---|---|---|---|---|---|
| 1 | A | 5504 | 0 | 5413 | 237 | 0 |
| 1 | B | 5504 | 0 | 5413 | 249 | 0 |
| 1 | C | 5504 | 0 | 5413 | 229 | 0 |
| 1 | D | 5504 | 0 | 5413 | 236 | 0 |
| All | All | 22016 | 0 | 21652 | 938 | 0 |

For Manuscript Review

The all-atom clashscore is defined as the number of clashes found per 1000 atoms (including hydrogen atoms). The all-atom clashscore for this structure is 21.

All (938) close contacts within the same asymmetric unit are listed below, sorted by their clash magnitude.

| Atom-1 | Atom-2 | Interatomic distance (Å) | Clash overlap (Å) |
|---|---|---|---|
| 1:D:297:LEU:O | 1:D:301:VAL:CG1 | 1.94 | 1.16 |
| 1:D:297:LEU:O | 1:D:301:VAL:HG12 | 1.44 | 1.11 |
| 1:D:631:ALA:C | 1:D:635:LYS:HE2 | 1.77 | 1.05 |
| 1:A:631:ALA:C | 1:A:635:LYS:HE2 | 1.82 | 0.98 |
| 1:B:631:ALA:O | 1:B:635:LYS:HE3 | 1.67 | 0.93 |
| 1:D:631:ALA:O | 1:D:635:LYS:HE2 | 1.68 | 0.93 |
| 1:B:297:LEU:O | 1:B:301:VAL:HG13 | 1.70 | 0.91 |
| 1:B:632:TRP:HA | 1:B:635:LYS:CE | 2.01 | 0.90 |
| 1:A:297:LEU:O | 1:A:301:VAL:HG13 | 1.70 | 0.90 |
| 1:C:661:HIS:NE2 | 1:D:667:ARG:NH2 | 2.21 | 0.89 |
| 1:D:304:PHE:HE2 | 1:D:309:PRO:HD3 | 1.36 | 0.89 |
| 1:A:631:ALA:O | 1:A:635:LYS:HE2 | 1.72 | 0.88 |
| 1:C:297:LEU:O | 1:C:301:VAL:HG13 | 1.74 | 0.87 |
| 1:D:365:ASP:OD1 | 1:D:366:ILE:N | 2.09 | 0.86 |
| 1:B:365:ASP:OD1 | 1:B:366:ILE:N | 2.09 | 0.85 |
| 1:C:365:ASP:OD1 | 1:C:366:ILE:N | 2.09 | 0.85 |
| 1:A:663:LEU:HD12 | 1:A:665:GLY:H | 1.42 | 0.85 |
| 1:B:663:LEU:HD12 | 1:B:665:GLY:H | 1.42 | 0.84 |
| 1:A:365:ASP:OD1 | 1:A:366:ILE:N | 2.09 | 0.84 |
| 1:C:663:LEU:HD12 | 1:C:665:GLY:H | 1.42 | 0.83 |
| 1:D:297:LEU:O | 1:D:301:VAL:HG13 | 1.76 | 0.83 |
| 1:D:663:LEU:HD12 | 1:D:665:GLY:H | 1.42 | 0.83 |
| 1:A:645:ASP:N | 1:A:645:ASP:OD1 | 2.14 | 0.80 |
| 1:D:632:TRP:HA | 1:D:635:LYS:CE | 2.10 | 0.80 |
| 1:D:298:ASP:HA | 1:D:301:VAL:HG13 | 1.63 | 0.80 |
| 1:A:632:TRP:HA | 1:A:635:LYS:CE | 2.12 | 0.79 |
| 1:C:297:LEU:O | 1:C:301:VAL:CG1 | 2.30 | 0.79 |
| 1:C:645:ASP:N | 1:C:645:ASP:OD1 | 2.14 | 0.79 |
| 1:D:645:ASP:OD1 | 1:D:645:ASP:N | 2.14 | 0.78 |
| 1:D:632:TRP:N | 1:D:635:LYS:HE2 | 1.98 | 0.78 |
| 1:B:297:LEU:O | 1:B:301:VAL:CG1 | 2.32 | 0.78 |
| 1:C:635:LYS:NZ | 1:C:642:ILE:HG22 | 1.98 | 0.78 |
| 1:A:297:LEU:O | 1:A:301:VAL:CG1 | 2.32 | 0.77 |
| 1:B:645:ASP:OD1 | 1:B:645:ASP:N | 2.14 | 0.76 |
| 1:B:191:GLU:OE1 | 1:B:191:GLU:N | 2.18 | 0.76 |
| 1:D:298:ASP:CA | 1:D:301:VAL:HG13 | 2.14 | 0.76 |
| 1:A:632:TRP:N | 1:A:635:LYS:HE2 | 2.01 | 0.75 |

Continued on next page...

For Manuscript Review

*Continued from previous page...*

| Atom-1 | Atom-2 | Interatomic distance (Å) | Clash overlap (Å) |
|---|---|---|---|
| 1:A:207:ASP:HA | 1:A:210:LYS:HE3 | 1.69 | 0.75 |
| 1:C:635:LYS:HZ2 | 1:C:642:ILE:HG22 | 1.51 | 0.74 |
| 1:D:304:PHE:CE2 | 1:D:309:PRO:HD3 | 2.21 | 0.74 |
| 1:C:207:ASP:HA | 1:C:210:LYS:HE3 | 1.69 | 0.74 |
| 1:B:207:ASP:HA | 1:B:210:LYS:HE3 | 1.69 | 0.74 |
| 1:A:191:GLU:OE1 | 1:A:191:GLU:N | 2.18 | 0.73 |
| 1:D:207:ASP:HA | 1:D:210:LYS:HE3 | 1.69 | 0.73 |
| 1:D:191:GLU:OE1 | 1:D:191:GLU:N | 2.18 | 0.71 |
| 1:D:631:ALA:O | 1:D:635:LYS:CE | 2.38 | 0.71 |
| 1:B:631:ALA:O | 1:B:635:LYS:CE | 2.37 | 0.71 |
| 1:C:191:GLU:OE1 | 1:C:191:GLU:N | 2.18 | 0.71 |
| 1:B:615:ASP:OD2 | 1:D:667:ARG:NH1 | 2.25 | 0.69 |
| 1:D:203:PRO:HB3 | 1:D:227:TYR:HE1 | 1.58 | 0.69 |
| 1:C:203:PRO:HB3 | 1:C:227:TYR:HE1 | 1.58 | 0.68 |
| 1:B:239:ARG:O | 1:B:243:HIS:ND1 | 2.26 | 0.68 |
| 1:A:239:ARG:O | 1:A:243:HIS:ND1 | 2.26 | 0.68 |
| 1:C:239:ARG:O | 1:C:243:HIS:ND1 | 2.26 | 0.68 |
| 1:D:192:ASP:HB2 | 1:D:453:LYS:HG3 | 1.76 | 0.68 |
| 1:C:192:ASP:HB2 | 1:C:453:LYS:HG3 | 1.76 | 0.68 |
| 1:A:632:TRP:CA | 1:A:635:LYS:HE2 | 2.23 | 0.68 |
| 1:D:632:TRP:CA | 1:D:635:LYS:HE2 | 2.23 | 0.68 |
| 1:A:716:GLU:OE2 | 1:A:719:ARG:NH1 | 2.27 | 0.67 |
| 1:A:203:PRO:HB3 | 1:A:227:TYR:HE1 | 1.58 | 0.67 |
| 1:C:667:ARG:NH2 | 1:D:660:ARG:O | 2.28 | 0.67 |
| 1:C:716:GLU:OE2 | 1:C:719:ARG:NH1 | 2.27 | 0.67 |
| 1:B:192:ASP:HB2 | 1:B:453:LYS:HG3 | 1.76 | 0.67 |
| 1:B:203:PRO:HB3 | 1:B:227:TYR:HE1 | 1.58 | 0.67 |
| 1:B:716:GLU:OE2 | 1:B:719:ARG:NH1 | 2.27 | 0.67 |
| 1:D:239:ARG:O | 1:D:243:HIS:ND1 | 2.26 | 0.67 |
| 1:A:631:ALA:O | 1:A:635:LYS:CE | 2.43 | 0.67 |
| 1:A:632:TRP:HA | 1:A:635:LYS:HE2 | 1.76 | 0.67 |
| 1:D:716:GLU:OE2 | 1:D:719:ARG:NH1 | 2.27 | 0.66 |
| 1:A:98:GLU:OE1 | 1:A:505:LEU:N | 2.29 | 0.66 |
| 1:D:345:TYR:O | 1:D:349:ASN:ND2 | 2.28 | 0.66 |
| 1:C:98:GLU:OE1 | 1:C:505:LEU:N | 2.28 | 0.66 |
| 1:C:106:THR:CG2 | 1:C:126:THR:HB | 2.26 | 0.66 |
| 1:D:106:THR:CG2 | 1:D:126:THR:HB | 2.26 | 0.66 |
| 1:D:713:GLU:N | 1:D:713:GLU:OE2 | 2.29 | 0.66 |
| 1:B:631:ALA:C | 1:B:635:LYS:CE | 2.64 | 0.66 |
| 1:A:192:ASP:HB2 | 1:A:453:LYS:HG3 | 1.76 | 0.66 |
| 1:B:106:THR:CG2 | 1:B:126:THR:HB | 2.26 | 0.66 |

*Continued on next page...*

For Manuscript Review

*Continued from previous page...*

| Atom-1 | Atom-2 | Interatomic distance (Å) | Clash overlap (Å) |
|---|---|---|---|
| 1:D:98:GLU:OE1 | 1:D:505:LEU:N | 2.29 | 0.65 |
| 1:A:713:GLU:OE2 | 1:A:713:GLU:N | 2.29 | 0.65 |
| 1:B:98:GLU:OE1 | 1:B:505:LEU:N | 2.29 | 0.65 |
| 1:A:140:TRP:NE1 | 1:A:158:CYS:SG | 2.69 | 0.65 |
| 1:C:443:ASP:OD1 | 1:C:443:ASP:N | 2.29 | 0.65 |
| 1:D:729:ASN:OD1 | 1:D:730:ARG:N | 2.30 | 0.65 |
| 1:C:140:TRP:NE1 | 1:C:158:CYS:SG | 2.70 | 0.65 |
| 1:C:713:GLU:OE2 | 1:C:713:GLU:N | 2.29 | 0.65 |
| 1:B:140:TRP:NE1 | 1:B:158:CYS:SG | 2.69 | 0.65 |
| 1:C:304:PHE:HE2 | 1:C:309:PRO:HD3 | 1.60 | 0.65 |
| 1:B:632:TRP:CA | 1:B:635:LYS:CE | 2.74 | 0.65 |
| 1:D:140:TRP:NE1 | 1:D:158:CYS:SG | 2.69 | 0.65 |
| 1:A:106:THR:CG2 | 1:A:126:THR:HB | 2.26 | 0.65 |
| 1:A:345:TYR:O | 1:A:349:ASN:ND2 | 2.28 | 0.65 |
| 1:B:729:ASN:OD1 | 1:B:730:ARG:N | 2.30 | 0.64 |
| 1:A:761:VAL:HG13 | 1:A:762:ILE:HG13 | 1.79 | 0.64 |
| 1:C:761:VAL:HG13 | 1:C:762:ILE:HG13 | 1.79 | 0.64 |
| 1:B:761:VAL:HG13 | 1:B:762:ILE:HG13 | 1.79 | 0.64 |
| 1:A:594:ILE:O | 1:A:599:ASN:ND2 | 2.27 | 0.64 |
| 1:A:443:ASP:N | 1:A:443:ASP:OD1 | 2.29 | 0.64 |
| 1:B:443:ASP:OD1 | 1:B:443:ASP:N | 2.29 | 0.64 |
| 1:B:632:TRP:HA | 1:B:635:LYS:HE3 | 1.77 | 0.64 |
| 1:D:443:ASP:N | 1:D:443:ASP:OD1 | 2.29 | 0.64 |
| 1:A:729:ASN:OD1 | 1:A:730:ARG:N | 2.30 | 0.64 |
| 1:B:713:GLU:N | 1:B:713:GLU:OE2 | 2.29 | 0.64 |
| 1:B:736:PHE:O | 1:B:740:VAL:HG12 | 1.98 | 0.64 |
| 1:C:729:ASN:OD1 | 1:C:730:ARG:N | 2.30 | 0.63 |
| 1:A:736:PHE:O | 1:A:740:VAL:HG12 | 1.98 | 0.63 |
| 1:B:345:TYR:O | 1:B:349:ASN:ND2 | 2.28 | 0.63 |
| 1:D:632:TRP:HA | 1:D:635:LYS:HE2 | 1.79 | 0.63 |
| 1:D:297:LEU:HA | 1:D:300:ILE:HG12 | 1.80 | 0.63 |
| 1:B:304:PHE:HE2 | 1:B:309:PRO:HD3 | 1.63 | 0.63 |
| 1:C:345:TYR:O | 1:C:349:ASN:ND2 | 2.28 | 0.63 |
| 1:D:761:VAL:HG13 | 1:D:762:ILE:HG13 | 1.79 | 0.63 |
| 1:D:220:GLU:O | 1:D:224:LYS:NZ | 2.30 | 0.63 |
| 1:A:304:PHE:HE2 | 1:A:309:PRO:HD3 | 1.63 | 0.63 |
| 1:D:736:PHE:O | 1:D:740:VAL:HG12 | 1.98 | 0.63 |
| 1:C:736:PHE:O | 1:C:740:VAL:HG12 | 1.98 | 0.62 |
| 1:C:661:HIS:CD2 | 1:D:667:ARG:HH21 | 2.17 | 0.62 |
| 1:B:594:ILE:O | 1:B:599:ASN:ND2 | 2.27 | 0.62 |
| 1:D:608:ILE:HD12 | 1:D:625:TRP:HB2 | 1.82 | 0.62 |

*Continued on next page...*

For Manuscript Review

Continued from previous page...

| Atom-1 | Atom-2 | Interatomic distance (Å) | Clash overlap (Å) |
|---|---|---|---|
| 1:B:608:ILE:HD12 | 1:B:625:TRP:HB2 | 1.81 | 0.62 |
| 1:C:667:ARG:HB3 | 1:C:671:TRP:CZ2 | 2.35 | 0.62 |
| 1:C:181:GLU:O | 1:C:184:TRP:NE1 | 2.33 | 0.62 |
| 1:D:181:GLU:O | 1:D:184:TRP:NE1 | 2.33 | 0.62 |
| 1:A:181:GLU:O | 1:A:184:TRP:NE1 | 2.33 | 0.62 |
| 1:B:667:ARG:CZ | 1:A:656:ILE:HG13 | 2.30 | 0.61 |
| 1:A:667:ARG:HB3 | 1:A:671:TRP:CZ2 | 2.35 | 0.61 |
| 1:B:181:GLU:O | 1:B:184:TRP:NE1 | 2.33 | 0.61 |
| 1:A:523:GLN:NE2 | 1:A:761:VAL:O | 2.34 | 0.61 |
| 1:B:632:TRP:HA | 1:B:635:LYS:NZ | 2.14 | 0.61 |
| 1:B:667:ARG:HB3 | 1:B:671:TRP:CZ2 | 2.35 | 0.61 |
| 1:C:367:ASP:OD1 | 1:C:368:ASP:N | 2.33 | 0.61 |
| 1:C:608:ILE:HD12 | 1:C:625:TRP:HB2 | 1.81 | 0.61 |
| 1:D:413:TYR:CZ | 1:D:417:ARG:HD2 | 2.36 | 0.61 |
| 1:B:413:TYR:CZ | 1:B:417:ARG:HD2 | 2.36 | 0.60 |
| 1:A:265:LEU:O | 1:A:268:ARG:NH1 | 2.30 | 0.60 |
| 1:A:608:ILE:HD12 | 1:A:625:TRP:HB2 | 1.81 | 0.60 |
| 1:C:413:TYR:CZ | 1:C:417:ARG:HD2 | 2.36 | 0.60 |
| 1:B:523:GLN:NE2 | 1:B:761:VAL:O | 2.34 | 0.60 |
| 1:C:523:GLN:NE2 | 1:C:761:VAL:O | 2.34 | 0.60 |
| 1:B:569:ASN:N | 1:B:569:ASN:OD1 | 2.34 | 0.60 |
| 1:A:413:TYR:CZ | 1:A:417:ARG:HD2 | 2.36 | 0.60 |
| 1:B:367:ASP:OD1 | 1:B:368:ASP:N | 2.33 | 0.60 |
| 1:D:265:LEU:O | 1:D:268:ARG:NH1 | 2.30 | 0.60 |
| 1:B:408:SER:HB2 | 1:B:453:LYS:HE2 | 1.84 | 0.60 |
| 1:B:632:TRP:N | 1:B:635:LYS:HE2 | 2.17 | 0.60 |
| 1:C:543:GLU:OE1 | 1:C:543:GLU:N | 2.30 | 0.60 |
| 1:D:170:ASP:OD1 | 1:D:170:ASP:N | 2.35 | 0.60 |
| 1:D:523:GLN:NE2 | 1:D:761:VAL:O | 2.34 | 0.60 |
| 1:C:594:ILE:O | 1:C:599:ASN:ND2 | 2.27 | 0.59 |
| 1:D:717:LEU:O | 1:D:721:VAL:HG23 | 2.02 | 0.59 |
| 1:B:170:ASP:N | 1:B:170:ASP:OD1 | 2.35 | 0.59 |
| 1:A:367:ASP:OD1 | 1:A:368:ASP:N | 2.33 | 0.59 |
| 1:A:408:SER:HB2 | 1:A:453:LYS:HE2 | 1.84 | 0.59 |
| 1:C:408:SER:HB2 | 1:C:453:LYS:HE2 | 1.84 | 0.59 |
| 1:A:170:ASP:OD1 | 1:A:170:ASP:N | 2.35 | 0.59 |
| 1:A:569:ASN:OD1 | 1:A:569:ASN:N | 2.34 | 0.59 |
| 1:A:717:LEU:O | 1:A:721:VAL:HG23 | 2.02 | 0.59 |
| 1:D:367:ASP:OD1 | 1:D:368:ASP:N | 2.33 | 0.59 |
| 1:C:170:ASP:OD1 | 1:C:170:ASP:N | 2.35 | 0.59 |
| 1:C:717:LEU:O | 1:C:721:VAL:HG23 | 2.02 | 0.59 |

Continued on next page...

For Manuscript Review

*Continued from previous page...*

| Atom-1 | Atom-2 | Interatomic distance (Å) | Clash overlap (Å) |
|---|---|---|---|
| 1:A:220:GLU:O | 1:A:224:LYS:NZ | 2.30 | 0.58 |
| 1:B:543:GLU:OE1 | 1:B:543:GLU:N | 2.29 | 0.58 |
| 1:D:543:GLU:OE1 | 1:D:543:GLU:N | 2.30 | 0.58 |
| 1:D:569:ASN:N | 1:D:569:ASN:OD1 | 2.34 | 0.58 |
| 1:D:408:SER:HB2 | 1:D:453:LYS:HE2 | 1.84 | 0.58 |
| 1:D:105:ASP:O | 1:D:109:VAL:HG23 | 2.04 | 0.58 |
| 1:D:667:ARG:HB3 | 1:D:671:TRP:CZ2 | 2.39 | 0.58 |
| 1:A:181:GLU:OE2 | 1:A:182:ASN:ND2 | 2.37 | 0.58 |
| 1:C:181:GLU:OE2 | 1:C:182:ASN:ND2 | 2.37 | 0.58 |
| 1:A:105:ASP:O | 1:A:109:VAL:HG23 | 2.04 | 0.57 |
| 1:B:717:LEU:O | 1:B:721:VAL:HG23 | 2.02 | 0.57 |
| 1:A:509:ASP:O | 1:A:513:LYS:NZ | 2.37 | 0.57 |
| 1:B:632:TRP:CA | 1:B:635:LYS:HE3 | 2.34 | 0.57 |
| 1:C:158:CYS:SG | 1:C:159:VAL:N | 2.78 | 0.57 |
| 1:C:661:HIS:CD2 | 1:D:667:ARG:NH2 | 2.72 | 0.57 |
| 1:B:105:ASP:O | 1:B:109:VAL:HG23 | 2.04 | 0.57 |
| 1:B:269:CYS:N | 1:B:273:SER:O | 2.32 | 0.57 |
| 1:B:419:SER:O | 1:B:419:SER:OG | 2.23 | 0.57 |
| 1:D:297:LEU:C | 1:D:301:VAL:CG1 | 2.71 | 0.57 |
| 1:D:423:PHE:CG | 1:D:719:ARG:HG3 | 2.40 | 0.57 |
| 1:D:181:GLU:OE2 | 1:D:182:ASN:ND2 | 2.37 | 0.57 |
| 1:D:419:SER:O | 1:D:419:SER:OG | 2.23 | 0.57 |
| 1:B:181:GLU:OE2 | 1:B:182:ASN:ND2 | 2.37 | 0.57 |
| 1:B:509:ASP:O | 1:B:513:LYS:NZ | 2.38 | 0.57 |
| 1:D:158:CYS:SG | 1:D:159:VAL:N | 2.78 | 0.57 |
| 1:B:412:MET:HB3 | 1:B:437:CYS:SG | 2.45 | 0.56 |
| 1:C:105:ASP:O | 1:C:109:VAL:HG23 | 2.04 | 0.56 |
| 1:A:360:ASN:OD1 | 1:A:360:ASN:N | 2.38 | 0.56 |
| 1:A:423:PHE:CG | 1:A:719:ARG:HG3 | 2.40 | 0.56 |
| 1:C:265:LEU:O | 1:C:268:ARG:NH1 | 2.30 | 0.56 |
| 1:A:158:CYS:SG | 1:A:159:VAL:N | 2.78 | 0.56 |
| 1:B:360:ASN:OD1 | 1:B:360:ASN:N | 2.38 | 0.56 |
| 1:B:670:LEU:HD13 | 1:A:673:TYR:CE2 | 2.41 | 0.56 |
| 1:C:220:GLU:O | 1:C:224:LYS:NZ | 2.30 | 0.56 |
| 1:C:227:TYR:HD1 | 1:C:230:ARG:HD3 | 1.70 | 0.56 |
| 1:D:227:TYR:HD1 | 1:D:230:ARG:HD3 | 1.70 | 0.56 |
| 1:A:227:TYR:HD1 | 1:A:230:ARG:HD3 | 1.70 | 0.56 |
| 1:A:412:MET:HB3 | 1:A:437:CYS:SG | 2.45 | 0.56 |
| 1:C:412:MET:HB3 | 1:C:437:CYS:SG | 2.45 | 0.56 |
| 1:D:509:ASP:O | 1:D:513:LYS:NZ | 2.37 | 0.56 |
| 1:D:706:ILE:O | 1:D:710:VAL:HG12 | 2.06 | 0.56 |

*Continued on next page...*

For Manuscript Review

*Continued from previous page...*

| Atom-1 | Atom-2 | Interatomic distance (Å) | Clash overlap (Å) |
|---|---|---|---|
| 1:D:360:ASN:OD1 | 1:D:360:ASN:N | 2.38 | 0.56 |
| 1:B:265:LEU:O | 1:B:268:ARG:NH1 | 2.30 | 0.56 |
| 1:C:423:PHE:CG | 1:C:719:ARG:HG3 | 2.40 | 0.56 |
| 1:C:450:MET:HG3 | 1:C:461:PRO:HG3 | 1.88 | 0.56 |
| 1:D:594:ILE:O | 1:D:599:ASN:ND2 | 2.27 | 0.56 |
| 1:A:706:ILE:O | 1:A:710:VAL:HG12 | 2.06 | 0.56 |
| 1:C:509:ASP:O | 1:C:513:LYS:NZ | 2.38 | 0.56 |
| 1:C:569:ASN:OD1 | 1:C:569:ASN:N | 2.34 | 0.56 |
| 1:A:450:MET:HG3 | 1:A:461:PRO:HG3 | 1.88 | 0.56 |
| 1:B:423:PHE:CG | 1:B:719:ARG:HG3 | 2.40 | 0.56 |
| 1:B:631:ALA:C | 1:B:635:LYS:HE2 | 2.25 | 0.56 |
| 1:C:706:ILE:O | 1:C:710:VAL:HG12 | 2.06 | 0.56 |
| 1:B:227:TYR:HD1 | 1:B:230:ARG:HD3 | 1.70 | 0.56 |
| 1:D:752:GLU:OE1 | 1:D:752:GLU:N | 2.37 | 0.56 |
| 1:A:576:GLY:O | 1:A:580:VAL:HG13 | 2.06 | 0.56 |
| 1:D:450:MET:HG3 | 1:D:461:PRO:HG3 | 1.88 | 0.55 |
| 1:B:597:PRO:HD2 | 1:B:636:GLU:HG3 | 1.89 | 0.55 |
| 1:D:412:MET:HB3 | 1:D:437:CYS:SG | 2.45 | 0.55 |
| 1:B:576:GLY:O | 1:B:580:VAL:HG13 | 2.06 | 0.55 |
| 1:B:760:LYS:HA | 1:B:764:GLN:HB3 | 1.89 | 0.55 |
| 1:B:158:CYS:SG | 1:B:159:VAL:N | 2.78 | 0.55 |
| 1:B:706:ILE:O | 1:B:710:VAL:HG12 | 2.06 | 0.55 |
| 1:D:693:ALA:HB1 | 1:D:698:GLU:HG3 | 1.88 | 0.55 |
| 1:A:597:PRO:HD2 | 1:A:636:GLU:HG3 | 1.89 | 0.55 |
| 1:C:136:PRO:O | 1:C:174:ARG:NH2 | 2.34 | 0.55 |
| 1:C:242:LEU:HD21 | 1:C:249:LEU:HD12 | 1.89 | 0.55 |
| 1:C:635:LYS:HZ2 | 1:C:642:ILE:HA | 1.72 | 0.55 |
| 1:D:242:LEU:HD21 | 1:D:249:LEU:HD12 | 1.89 | 0.55 |
| 1:D:298:ASP:HA | 1:D:301:VAL:CG1 | 2.33 | 0.55 |
| 1:D:597:PRO:HD2 | 1:D:636:GLU:HG3 | 1.89 | 0.55 |
| 1:B:368:ASP:N | 1:B:368:ASP:OD1 | 2.40 | 0.55 |
| 1:C:576:GLY:O | 1:C:580:VAL:HG13 | 2.06 | 0.55 |
| 1:C:693:ALA:HB1 | 1:C:698:GLU:HG3 | 1.88 | 0.55 |
| 1:A:148:MET:HE2 | 1:A:148:MET:HA | 1.88 | 0.55 |
| 1:A:419:SER:O | 1:A:419:SER:OG | 2.23 | 0.55 |
| 1:A:760:LYS:HA | 1:A:764:GLN:HB3 | 1.89 | 0.55 |
| 1:B:195:LEU:HB2 | 1:B:198:PHE:HB2 | 1.89 | 0.54 |
| 1:C:368:ASP:N | 1:C:368:ASP:OD1 | 2.39 | 0.54 |
| 1:C:597:PRO:HD2 | 1:C:636:GLU:HG3 | 1.89 | 0.54 |
| 1:D:368:ASP:N | 1:D:368:ASP:OD1 | 2.39 | 0.54 |
| 1:A:100:SER:OG | 1:A:502:ARG:O | 2.25 | 0.54 |

*Continued on next page...*

For Manuscript Review

Continued from previous page...

| Atom-1 | Atom-2 | Interatomic distance (Å) | Clash overlap (Å) |
|---|---|---|---|
| 1:A:145:PHE:O | 1:A:151:ARG:NH2 | 2.29 | 0.54 |
| 1:A:543:GLU:OE1 | 1:A:543:GLU:N | 2.29 | 0.54 |
| 1:B:631:ALA:C | 1:B:635:LYS:HE3 | 2.24 | 0.54 |
| 1:D:576:GLY:O | 1:D:580:VAL:HG13 | 2.06 | 0.54 |
| 1:A:452:ASP:OD1 | 1:A:454:TRP:N | 2.36 | 0.54 |
| 1:A:693:ALA:HB1 | 1:A:698:GLU:HG3 | 1.88 | 0.54 |
| 1:B:100:SER:OG | 1:B:502:ARG:O | 2.25 | 0.54 |
| 1:B:220:GLU:O | 1:B:224:LYS:NZ | 2.30 | 0.54 |
| 1:C:269:CYS:N | 1:C:273:SER:O | 2.32 | 0.54 |
| 1:A:242:LEU:HD21 | 1:A:249:LEU:HD12 | 1.89 | 0.54 |
| 1:B:242:LEU:HD21 | 1:B:249:LEU:HD12 | 1.89 | 0.54 |
| 1:B:693:ALA:HB1 | 1:B:698:GLU:HG3 | 1.88 | 0.54 |
| 1:B:450:MET:HG3 | 1:B:461:PRO:HG3 | 1.88 | 0.54 |
| 1:D:195:LEU:HB2 | 1:D:198:PHE:HB2 | 1.89 | 0.54 |
| 1:A:136:PRO:O | 1:A:174:ARG:NH2 | 2.34 | 0.54 |
| 1:A:368:ASP:N | 1:A:368:ASP:OD1 | 2.39 | 0.54 |
| 1:C:195:LEU:HB2 | 1:C:198:PHE:HB2 | 1.89 | 0.54 |
| 1:A:752:GLU:OE1 | 1:A:752:GLU:N | 2.37 | 0.54 |
| 1:B:632:TRP:HA | 1:B:635:LYS:HZ1 | 1.72 | 0.54 |
| 1:C:609:SER:HA | 1:C:613:PHE:CE2 | 2.43 | 0.54 |
| 1:C:760:LYS:HA | 1:C:764:GLN:HB3 | 1.89 | 0.54 |
| 1:D:100:SER:OG | 1:D:502:ARG:O | 2.25 | 0.54 |
| 1:D:760:LYS:HA | 1:D:764:GLN:HB3 | 1.89 | 0.54 |
| 1:A:220:GLU:H | 1:A:224:LYS:HZ1 | 1.55 | 0.54 |
| 1:A:548:THR:OG1 | 1:A:549:ASP:N | 2.41 | 0.54 |
| 1:B:766:VAL:HG23 | 1:B:767:ILE:HG23 | 1.90 | 0.53 |
| 1:B:145:PHE:O | 1:B:151:ARG:NH2 | 2.29 | 0.53 |
| 1:C:548:THR:OG1 | 1:C:549:ASP:N | 2.41 | 0.53 |
| 1:A:527:ARG:NH1 | 1:A:762:ILE:O | 2.42 | 0.53 |
| 1:C:598:LYS:NZ | 1:C:636:GLU:OE2 | 2.42 | 0.53 |
| 1:D:269:CYS:N | 1:D:273:SER:O | 2.32 | 0.53 |
| 1:A:609:SER:HA | 1:A:613:PHE:CE2 | 2.43 | 0.53 |
| 1:D:598:LYS:NZ | 1:D:636:GLU:OE2 | 2.42 | 0.53 |
| 1:B:148:MET:HA | 1:B:148:MET:HE2 | 1.91 | 0.53 |
| 1:B:527:ARG:NH1 | 1:B:762:ILE:O | 2.42 | 0.53 |
| 1:B:609:SER:HA | 1:B:613:PHE:CE2 | 2.43 | 0.53 |
| 1:C:527:ARG:NH1 | 1:C:762:ILE:O | 2.42 | 0.53 |
| 1:C:752:GLU:OE1 | 1:C:752:GLU:N | 2.37 | 0.53 |
| 1:A:195:LEU:HB2 | 1:A:198:PHE:HB2 | 1.89 | 0.53 |
| 1:A:605:GLU:CD | 1:A:605:GLU:H | 2.12 | 0.53 |
| 1:B:630:MET:O | 1:B:633:THR:OG1 | 2.21 | 0.53 |

Continued on next page...

For Manuscript Review

Continued from previous page...

| Atom-1 | Atom-2 | Interatomic distance (Å) | Clash overlap (Å) |
|---|---|---|---|
| 1:D:136:PRO:O | 1:D:174:ARG:NH2 | 2.34 | 0.53 |
| 1:D:609:SER:HA | 1:D:613:PHE:CE2 | 2.43 | 0.53 |
| 1:A:598:LYS:NZ | 1:A:636:GLU:OE2 | 2.42 | 0.53 |
| 1:A:766:VAL:HG23 | 1:A:767:ILE:HG23 | 1.90 | 0.53 |
| 1:C:145:PHE:O | 1:C:151:ARG:NH2 | 2.30 | 0.53 |
| 1:C:765:ASP:OD1 | 1:C:766:VAL:N | 2.42 | 0.53 |
| 1:D:548:THR:OG1 | 1:D:549:ASP:N | 2.41 | 0.53 |
| 1:A:630:MET:O | 1:A:633:THR:OG1 | 2.21 | 0.53 |
| 1:C:148:MET:HE2 | 1:C:148:MET:HA | 1.91 | 0.53 |
| 1:D:527:ARG:NH1 | 1:D:762:ILE:O | 2.42 | 0.53 |
| 1:A:605:GLU:HA | 1:A:625:TRP:CD1 | 2.44 | 0.53 |
| 1:A:728:ILE:O | 1:A:733:ARG:NH1 | 2.42 | 0.53 |
| 1:B:605:GLU:H | 1:B:605:GLU:CD | 2.12 | 0.52 |
| 1:A:148:MET:O | 1:A:152:ILE:HG12 | 2.10 | 0.52 |
| 1:B:296:TYR:O | 1:B:300:ILE:HG23 | 2.09 | 0.52 |
| 1:C:605:GLU:CD | 1:C:605:GLU:H | 2.12 | 0.52 |
| 1:C:605:GLU:HA | 1:C:625:TRP:CD1 | 2.45 | 0.52 |
| 1:D:605:GLU:HA | 1:D:625:TRP:CD1 | 2.44 | 0.52 |
| 1:D:148:MET:O | 1:D:152:ILE:HG12 | 2.09 | 0.52 |
| 1:B:419:SER:O | 1:B:422:LYS:NZ | 2.36 | 0.52 |
| 1:C:148:MET:O | 1:C:152:ILE:HG12 | 2.09 | 0.52 |
| 1:C:766:VAL:HG23 | 1:C:767:ILE:HG23 | 1.90 | 0.52 |
| 1:B:548:THR:OG1 | 1:B:549:ASP:N | 2.41 | 0.52 |
| 1:D:134:GLN:HG3 | 1:D:138:GLY:HA2 | 1.92 | 0.52 |
| 1:D:766:VAL:HG23 | 1:D:767:ILE:HG23 | 1.90 | 0.52 |
| 1:A:106:THR:HG23 | 1:A:126:THR:HB | 1.91 | 0.52 |
| 1:A:765:ASP:OD1 | 1:A:766:VAL:N | 2.42 | 0.52 |
| 1:B:518:ASP:HA | 1:B:521:ASN:HD21 | 1.75 | 0.52 |
| 1:B:598:LYS:NZ | 1:B:636:GLU:OE2 | 2.42 | 0.52 |
| 1:D:518:ASP:HA | 1:D:521:ASN:HD21 | 1.75 | 0.52 |
| 1:C:106:THR:HG23 | 1:C:126:THR:HB | 1.91 | 0.52 |
| 1:B:106:THR:HG23 | 1:B:126:THR:HB | 1.91 | 0.52 |
| 1:B:148:MET:O | 1:B:152:ILE:HG12 | 2.09 | 0.52 |
| 1:B:605:GLU:HA | 1:B:625:TRP:CD1 | 2.45 | 0.52 |
| 1:C:134:GLN:HG3 | 1:C:138:GLY:HA2 | 1.92 | 0.52 |
| 1:C:447:SER:OG | 1:C:449:ARG:NH1 | 2.43 | 0.52 |
| 1:D:106:THR:HG23 | 1:D:126:THR:HB | 1.91 | 0.52 |
| 1:A:296:TYR:O | 1:A:300:ILE:HG23 | 2.09 | 0.52 |
| 1:B:134:GLN:HG3 | 1:B:138:GLY:HA2 | 1.92 | 0.52 |
| 1:B:452:ASP:OD1 | 1:B:454:TRP:N | 2.36 | 0.52 |
| 1:C:296:TYR:O | 1:C:300:ILE:HG23 | 2.09 | 0.52 |

Continued on next page...

For Manuscript Review

Continued from previous page...

| Atom-1 | Atom-2 | Interatomic distance (Å) | Clash overlap (Å) |
|---|---|---|---|
| 1:D:605:GLU:H | 1:D:605:GLU:CD | 2.12 | 0.52 |
| 1:A:190:GLU:HB2 | 1:A:193:TRP:HB2 | 1.92 | 0.52 |
| 1:B:190:GLU:HB2 | 1:B:193:TRP:HB2 | 1.92 | 0.51 |
| 1:C:400:LEU:HD13 | 1:C:403:GLU:HB3 | 1.92 | 0.51 |
| 1:C:419:SER:O | 1:C:419:SER:OG | 2.23 | 0.51 |
| 1:C:518:ASP:HA | 1:C:521:ASN:HD21 | 1.75 | 0.51 |
| 1:D:393:LYS:HZ2 | 1:D:398:PHE:HB2 | 1.75 | 0.51 |
| 1:A:447:SER:OG | 1:A:449:ARG:NH1 | 2.43 | 0.51 |
| 1:C:240:ASP:O | 1:C:244:SER:N | 2.43 | 0.51 |
| 1:C:190:GLU:HB2 | 1:C:193:TRP:HB2 | 1.92 | 0.51 |
| 1:D:765:ASP:OD1 | 1:D:766:VAL:N | 2.42 | 0.51 |
| 1:A:316:VAL:HG13 | 1:A:346:ILE:HD12 | 1.93 | 0.51 |
| 1:B:447:SER:OG | 1:B:449:ARG:NH1 | 2.43 | 0.51 |
| 1:A:269:CYS:N | 1:A:273:SER:O | 2.32 | 0.51 |
| 1:A:518:ASP:HA | 1:A:521:ASN:HD21 | 1.75 | 0.51 |
| 1:A:240:ASP:O | 1:A:244:SER:N | 2.43 | 0.51 |
| 1:B:240:ASP:O | 1:B:244:SER:N | 2.43 | 0.51 |
| 1:D:116:ASP:OD1 | 1:D:116:ASP:N | 2.44 | 0.51 |
| 1:A:508:ASN:OD1 | 1:A:509:ASP:N | 2.44 | 0.51 |
| 1:D:316:VAL:HG13 | 1:D:346:ILE:HD12 | 1.93 | 0.51 |
| 1:A:478:ARG:HD3 | 1:A:566:PHE:CD2 | 2.46 | 0.51 |
| 1:B:478:ARG:HD3 | 1:B:566:PHE:CD2 | 2.46 | 0.51 |
| 1:C:360:ASN:N | 1:C:360:ASN:OD1 | 2.38 | 0.51 |
| 1:D:630:MET:O | 1:D:633:THR:OG1 | 2.21 | 0.51 |
| 1:B:400:LEU:HD13 | 1:B:403:GLU:HB3 | 1.92 | 0.51 |
| 1:B:765:ASP:OD1 | 1:B:766:VAL:N | 2.42 | 0.51 |
| 1:C:478:ARG:HD3 | 1:C:566:PHE:CD2 | 2.46 | 0.51 |
| 1:D:190:GLU:HB2 | 1:D:193:TRP:HB2 | 1.92 | 0.51 |
| 1:D:400:LEU:HD13 | 1:D:403:GLU:HB3 | 1.92 | 0.51 |
| 1:A:134:GLN:HG3 | 1:A:138:GLY:HA2 | 1.92 | 0.51 |
| 1:A:611:VAL:HB | 1:A:613:PHE:CE1 | 2.46 | 0.51 |
| 1:B:274:PHE:H | 1:B:281:THR:HG22 | 1.76 | 0.50 |
| 1:D:240:ASP:O | 1:D:244:SER:N | 2.43 | 0.50 |
| 1:B:611:VAL:HB | 1:B:613:PHE:CE1 | 2.46 | 0.50 |
| 1:C:191:GLU:H | 1:C:191:GLU:CD | 2.11 | 0.50 |
| 1:C:274:PHE:H | 1:C:281:THR:HG22 | 1.76 | 0.50 |
| 1:D:478:ARG:HD3 | 1:D:566:PHE:CD2 | 2.46 | 0.50 |
| 1:B:508:ASN:OD1 | 1:B:509:ASP:N | 2.44 | 0.50 |
| 1:C:508:ASN:OD1 | 1:C:509:ASP:N | 2.44 | 0.50 |
| 1:D:611:VAL:HB | 1:D:613:PHE:CE1 | 2.46 | 0.50 |
| 1:A:274:PHE:H | 1:A:281:THR:HG22 | 1.76 | 0.50 |

Continued on next page...

For Manuscript Review

*Continued from previous page...*

| Atom-1 | Atom-2 | Interatomic distance (Å) | Clash overlap (Å) |
|---|---|---|---|
| 1:D:508:ASN:OD1 | 1:D:509:ASP:N | 2.44 | 0.50 |
| 1:A:400:LEU:HD13 | 1:A:403:GLU:HB3 | 1.92 | 0.50 |
| 1:B:304:PHE:CE2 | 1:B:309:PRO:HD3 | 2.44 | 0.50 |
| 1:B:316:VAL:HG13 | 1:B:346:ILE:HD12 | 1.93 | 0.50 |
| 1:D:296:TYR:O | 1:D:300:ILE:HG23 | 2.11 | 0.50 |
| 1:A:315:ASP:OD1 | 1:A:315:ASP:N | 2.43 | 0.50 |
| 1:C:316:VAL:HG13 | 1:C:346:ILE:HD12 | 1.93 | 0.50 |
| 1:C:635:LYS:NZ | 1:C:645:ASP:OD2 | 2.28 | 0.50 |
| 1:D:728:ILE:O | 1:D:733:ARG:NH1 | 2.42 | 0.50 |
| 1:C:728:ILE:O | 1:C:733:ARG:NH1 | 2.42 | 0.50 |
| 1:A:419:SER:O | 1:A:422:LYS:NZ | 2.36 | 0.50 |
| 1:D:274:PHE:H | 1:D:281:THR:HG22 | 1.76 | 0.49 |
| 1:C:304:PHE:CE2 | 1:C:309:PRO:HD3 | 2.43 | 0.49 |
| 1:B:199:GLU:HB2 | 1:B:226:ILE:HB | 1.95 | 0.49 |
| 1:B:635:LYS:NZ | 1:B:642:ILE:HG22 | 2.27 | 0.49 |
| 1:C:611:VAL:HB | 1:C:613:PHE:CE1 | 2.46 | 0.49 |
| 1:D:452:ASP:OD1 | 1:D:454:TRP:N | 2.36 | 0.49 |
| 1:B:728:ILE:O | 1:B:733:ARG:NH1 | 2.42 | 0.49 |
| 1:C:393:LYS:HZ2 | 1:C:398:PHE:HB2 | 1.77 | 0.49 |
| 1:C:199:GLU:HB2 | 1:C:226:ILE:HB | 1.95 | 0.49 |
| 1:C:100:SER:OG | 1:C:502:ARG:O | 2.25 | 0.49 |
| 1:C:635:LYS:NZ | 1:C:642:ILE:CG2 | 2.73 | 0.49 |
| 1:D:223:LEU:HB3 | 1:D:224:LYS:HZ2 | 1.78 | 0.49 |
| 1:D:315:ASP:N | 1:D:315:ASP:OD1 | 2.43 | 0.49 |
| 1:A:484:TYR:CE2 | 1:A:488:TYR:HB2 | 2.48 | 0.49 |
| 1:C:484:TYR:CE2 | 1:C:488:TYR:HB2 | 2.48 | 0.49 |
| 1:D:589:SER:HA | 1:D:592:ARG:HE | 1.77 | 0.49 |
| 1:C:315:ASP:OD1 | 1:C:315:ASP:N | 2.43 | 0.49 |
| 1:B:220:GLU:H | 1:B:224:LYS:HZ1 | 1.61 | 0.49 |
| 1:B:251:HIS:HB2 | 1:B:275:HIS:CG | 2.48 | 0.49 |
| 1:B:315:ASP:N | 1:B:315:ASP:OD1 | 2.43 | 0.49 |
| 1:B:670:LEU:HD13 | 1:A:673:TYR:HE2 | 1.77 | 0.49 |
| 1:C:438:ARG:NH2 | 1:C:468:MET:O | 2.46 | 0.49 |
| 1:D:298:ASP:C | 1:D:301:VAL:HG13 | 2.33 | 0.49 |
| 1:D:484:TYR:CE2 | 1:D:488:TYR:HB2 | 2.48 | 0.49 |
| 1:A:438:ARG:NH2 | 1:A:468:MET:O | 2.46 | 0.49 |
| 1:A:251:HIS:HB2 | 1:A:275:HIS:CG | 2.48 | 0.48 |
| 1:B:484:TYR:CE2 | 1:B:488:TYR:HB2 | 2.48 | 0.48 |
| 1:C:176:LEU:HD21 | 1:C:215:ASP:OD1 | 2.13 | 0.48 |
| 1:C:427:ASP:OD1 | 1:C:428:GLY:N | 2.46 | 0.48 |
| 1:D:251:HIS:HB2 | 1:D:275:HIS:CG | 2.48 | 0.48 |

*Continued on next page...*

For Manuscript Review

*Continued from previous page...*

| Atom-1 | Atom-2 | Interatomic distance (Å) | Clash overlap (Å) |
|---|---|---|---|
| 1:D:438:ARG:NH2 | 1:D:468:MET:O | 2.46 | 0.48 |
| 1:A:199:GLU:HB2 | 1:A:226:ILE:HB | 1.95 | 0.48 |
| 1:B:203:PRO:O | 1:B:206:LEU:HG | 2.13 | 0.48 |
| 1:C:116:ASP:OD1 | 1:C:116:ASP:N | 2.44 | 0.48 |
| 1:C:731:LEU:HA | 1:C:734:GLU:CD | 2.34 | 0.48 |
| 1:D:145:PHE:O | 1:D:151:ARG:NH2 | 2.30 | 0.48 |
| 1:B:140:TRP:HB2 | 1:B:154:SER:HB2 | 1.96 | 0.48 |
| 1:D:199:GLU:HB2 | 1:D:226:ILE:HB | 1.95 | 0.48 |
| 1:D:731:LEU:HA | 1:D:734:GLU:CD | 2.34 | 0.48 |
| 1:B:579:ARG:NH1 | 1:B:612:PRO:HD2 | 2.29 | 0.48 |
| 1:C:589:SER:HA | 1:C:592:ARG:HE | 1.78 | 0.48 |
| 1:A:203:PRO:O | 1:A:206:LEU:HG | 2.13 | 0.48 |
| 1:B:136:PRO:O | 1:B:174:ARG:NH2 | 2.34 | 0.48 |
| 1:B:438:ARG:NH2 | 1:B:468:MET:O | 2.46 | 0.48 |
| 1:A:176:LEU:HD21 | 1:A:215:ASP:OD1 | 2.13 | 0.48 |
| 1:B:176:LEU:HA | 1:B:179:ILE:HD12 | 1.96 | 0.48 |
| 1:B:589:SER:HA | 1:B:592:ARG:HE | 1.78 | 0.48 |
| 1:B:731:LEU:HA | 1:B:734:GLU:CD | 2.34 | 0.48 |
| 1:D:427:ASP:OD1 | 1:D:428:GLY:N | 2.46 | 0.48 |
| 1:D:447:SER:OG | 1:D:449:ARG:NH1 | 2.43 | 0.48 |
| 1:A:140:TRP:HB2 | 1:A:154:SER:HB2 | 1.96 | 0.48 |
| 1:A:304:PHE:CE2 | 1:A:309:PRO:HD3 | 2.45 | 0.48 |
| 1:A:579:ARG:NH1 | 1:A:612:PRO:HD2 | 2.29 | 0.48 |
| 1:D:148:MET:HA | 1:D:148:MET:CE | 2.44 | 0.48 |
| 1:D:169:THR:HG22 | 1:D:173:GLU:OE2 | 2.14 | 0.48 |
| 1:D:203:PRO:O | 1:D:206:LEU:HG | 2.13 | 0.48 |
| 1:D:579:ARG:NH1 | 1:D:612:PRO:HD2 | 2.29 | 0.48 |
| 1:B:169:THR:HG22 | 1:B:173:GLU:OE2 | 2.14 | 0.48 |
| 1:B:176:LEU:HD21 | 1:B:215:ASP:OD1 | 2.13 | 0.48 |
| 1:B:542:LEU:HD12 | 1:B:542:LEU:HA | 1.70 | 0.48 |
| 1:C:236:LYS:HB3 | 1:C:236:LYS:HE3 | 1.56 | 0.48 |
| 1:C:630:MET:O | 1:C:633:THR:OG1 | 2.21 | 0.48 |
| 1:A:285:PHE:HA | 1:A:288:THR:O | 2.14 | 0.48 |
| 1:B:427:ASP:OD1 | 1:B:428:GLY:N | 2.47 | 0.48 |
| 1:D:236:LYS:HB3 | 1:D:236:LYS:HE3 | 1.56 | 0.48 |
| 1:A:427:ASP:OD1 | 1:A:428:GLY:N | 2.46 | 0.48 |
| 1:A:715:GLN:O | 1:A:718:THR:OG1 | 2.29 | 0.48 |
| 1:A:731:LEU:HA | 1:A:734:GLU:CD | 2.34 | 0.48 |
| 1:B:632:TRP:N | 1:B:635:LYS:CE | 2.77 | 0.47 |
| 1:C:176:LEU:HA | 1:C:179:ILE:HD12 | 1.96 | 0.47 |
| 1:C:297:LEU:O | 1:C:301:VAL:HG12 | 2.12 | 0.47 |

*Continued on next page...*

For Manuscript Review

*Continued from previous page...*

| Atom-1 | Atom-2 | Interatomic distance (Å) | Clash overlap (Å) |
|---|---|---|---|
| 1:C:715:GLN:O | 1:C:718:THR:OG1 | 2.29 | 0.47 |
| 1:A:122:GLN:HG3 | 1:A:123:PHE:HD2 | 1.79 | 0.47 |
| 1:C:251:HIS:HB2 | 1:C:275:HIS:CG | 2.48 | 0.47 |
| 1:D:176:LEU:HA | 1:D:179:ILE:HD12 | 1.96 | 0.47 |
| 1:C:148:MET:HA | 1:C:148:MET:CE | 2.44 | 0.47 |
| 1:C:169:THR:HG22 | 1:C:173:GLU:OE2 | 2.14 | 0.47 |
| 1:C:203:PRO:O | 1:C:206:LEU:HG | 2.13 | 0.47 |
| 1:C:452:ASP:OD1 | 1:C:454:TRP:N | 2.36 | 0.47 |
| 1:D:122:GLN:HG3 | 1:D:123:PHE:HD2 | 1.79 | 0.47 |
| 1:D:530:LEU:HD21 | 1:D:555:MET:HE1 | 1.96 | 0.47 |
| 1:A:169:THR:HG22 | 1:A:173:GLU:OE2 | 2.14 | 0.47 |
| 1:A:567:GLU:OE2 | 1:A:567:GLU:N | 2.45 | 0.47 |
| 1:B:148:MET:HA | 1:B:148:MET:CE | 2.44 | 0.47 |
| 1:B:494:VAL:HG12 | 1:B:502:ARG:HD2 | 1.97 | 0.47 |
| 1:C:285:PHE:HA | 1:C:288:THR:O | 2.14 | 0.47 |
| 1:C:579:ARG:NH1 | 1:C:612:PRO:HD2 | 2.29 | 0.47 |
| 1:C:122:GLN:HG3 | 1:C:123:PHE:HD2 | 1.80 | 0.47 |
| 1:D:635:LYS:HE3 | 1:D:635:LYS:HB2 | 1.40 | 0.47 |
| 1:D:735:THR:O | 1:D:739:VAL:HG23 | 2.15 | 0.47 |
| 1:A:176:LEU:HA | 1:A:179:ILE:HD12 | 1.96 | 0.47 |
| 1:B:605:GLU:HA | 1:B:625:TRP:NE1 | 2.30 | 0.47 |
| 1:D:176:LEU:HD21 | 1:D:215:ASP:OD1 | 2.14 | 0.47 |
| 1:D:605:GLU:HA | 1:D:625:TRP:NE1 | 2.30 | 0.47 |
| 1:B:285:PHE:HA | 1:B:288:THR:O | 2.14 | 0.47 |
| 1:C:494:VAL:HG12 | 1:C:502:ARG:HD2 | 1.97 | 0.47 |
| 1:D:185:ARG:HA | 1:D:188:HIS:CE1 | 2.50 | 0.47 |
| 1:A:148:MET:HA | 1:A:148:MET:CE | 2.44 | 0.47 |
| 1:A:185:ARG:HA | 1:A:188:HIS:CE1 | 2.50 | 0.47 |
| 1:A:230:ARG:NH1 | 1:A:231:GLU:OE2 | 2.48 | 0.47 |
| 1:A:494:VAL:HG12 | 1:A:502:ARG:HD2 | 1.97 | 0.47 |
| 1:A:737:LEU:HD23 | 1:A:737:LEU:HA | 1.76 | 0.47 |
| 1:B:185:ARG:HA | 1:B:188:HIS:CE1 | 2.50 | 0.47 |
| 1:D:140:TRP:HB2 | 1:D:154:SER:HB2 | 1.96 | 0.47 |
| 1:D:230:ARG:NH1 | 1:D:231:GLU:OE2 | 2.48 | 0.47 |
| 1:A:589:SER:HA | 1:A:592:ARG:HE | 1.78 | 0.47 |
| 1:C:140:TRP:HB2 | 1:C:154:SER:HB2 | 1.96 | 0.47 |
| 1:C:620:SER:HA | 1:C:623:GLU:OE2 | 2.15 | 0.47 |
| 1:D:152:ILE:HG13 | 1:D:153:MET:N | 2.30 | 0.47 |
| 1:A:236:LYS:HE3 | 1:A:236:LYS:HB3 | 1.56 | 0.47 |
| 1:B:228:ALA:O | 1:B:232:ARG:HG3 | 2.15 | 0.47 |
| 1:C:230:ARG:NH1 | 1:C:231:GLU:OE2 | 2.48 | 0.47 |

*Continued on next page...*

For Manuscript Review

Continued from previous page...

| Atom-1 | Atom-2 | Interatomic distance (Å) | Clash overlap (Å) |
|---|---|---|---|
| 1:C:605:GLU:HA | 1:C:625:TRP:NE1 | 2.30 | 0.47 |
| 1:D:494:VAL:HG12 | 1:D:502:ARG:HD2 | 1.97 | 0.47 |
| 1:C:735:THR:O | 1:C:739:VAL:HG23 | 2.15 | 0.46 |
| 1:D:163:LYS:HD3 | 1:D:169:THR:HG23 | 1.97 | 0.46 |
| 1:D:251:HIS:O | 1:D:280:SER:OG | 2.33 | 0.46 |
| 1:D:285:PHE:HA | 1:D:288:THR:O | 2.14 | 0.46 |
| 1:B:122:GLN:HG3 | 1:B:123:PHE:HD2 | 1.79 | 0.46 |
| 1:B:230:ARG:NH1 | 1:B:231:GLU:OE2 | 2.48 | 0.46 |
| 1:B:251:HIS:O | 1:B:280:SER:OG | 2.33 | 0.46 |
| 1:B:620:SER:HA | 1:B:623:GLU:OE2 | 2.15 | 0.46 |
| 1:B:752:GLU:OE1 | 1:B:752:GLU:N | 2.37 | 0.46 |
| 1:C:210:LYS:HB3 | 1:C:210:LYS:HE2 | 1.61 | 0.46 |
| 1:C:299:GLY:O | 1:C:302:LYS:HG2 | 2.16 | 0.46 |
| 1:D:240:ASP:HA | 1:D:243:HIS:HB2 | 1.98 | 0.46 |
| 1:A:605:GLU:HA | 1:A:625:TRP:NE1 | 2.30 | 0.46 |
| 1:B:116:ASP:N | 1:B:116:ASP:OD1 | 2.44 | 0.46 |
| 1:B:735:THR:O | 1:B:739:VAL:HG23 | 2.15 | 0.46 |
| 1:D:191:GLU:H | 1:D:191:GLU:CD | 2.11 | 0.46 |
| 1:A:152:ILE:HG13 | 1:A:153:MET:N | 2.30 | 0.46 |
| 1:A:163:LYS:HD3 | 1:A:169:THR:HG23 | 1.97 | 0.46 |
| 1:A:452:ASP:OD1 | 1:A:453:LYS:N | 2.49 | 0.46 |
| 1:A:529:GLU:OE2 | 1:A:558:TYR:OH | 2.26 | 0.46 |
| 1:C:163:LYS:HD3 | 1:C:169:THR:HG23 | 1.97 | 0.46 |
| 1:C:542:LEU:HD12 | 1:C:542:LEU:HA | 1.70 | 0.46 |
| 1:D:419:SER:OG | 1:D:434:GLU:HB2 | 2.16 | 0.46 |
| 1:A:223:LEU:HD23 | 1:A:224:LYS:HZ2 | 1.80 | 0.46 |
| 1:A:620:SER:HA | 1:A:623:GLU:OE2 | 2.15 | 0.46 |
| 1:B:397:PHE:HB2 | 1:B:436:PHE:CE2 | 2.51 | 0.46 |
| 1:C:223:LEU:HB3 | 1:C:224:LYS:HZ2 | 1.80 | 0.46 |
| 1:C:452:ASP:OD1 | 1:C:453:LYS:N | 2.49 | 0.46 |
| 1:D:737:LEU:HD23 | 1:D:737:LEU:HA | 1.76 | 0.46 |
| 1:A:735:THR:O | 1:A:739:VAL:HG23 | 2.15 | 0.46 |
| 1:B:503:MET:SD | 1:B:503:MET:N | 2.89 | 0.46 |
| 1:C:719:ARG:O | 1:C:723:GLN:HG2 | 2.16 | 0.46 |
| 1:D:620:SER:HA | 1:D:623:GLU:OE2 | 2.15 | 0.46 |
| 1:D:719:ARG:O | 1:D:723:GLN:HG2 | 2.16 | 0.46 |
| 1:A:419:SER:OG | 1:A:434:GLU:HB2 | 2.16 | 0.46 |
| 1:B:419:SER:OG | 1:B:434:GLU:HB2 | 2.16 | 0.46 |
| 1:B:719:ARG:O | 1:B:723:GLN:HG2 | 2.16 | 0.46 |
| 1:D:397:PHE:HB2 | 1:D:436:PHE:CE2 | 2.51 | 0.46 |
| 1:A:240:ASP:HA | 1:A:243:HIS:HB2 | 1.98 | 0.46 |

Continued on next page...

For Manuscript Review

Continued from previous page...

| Atom-1 | Atom-2 | Interatomic distance (Å) | Clash overlap (Å) |
|---|---|---|---|
| 1:B:180:GLN:HA | 1:B:183:MET:HG2 | 1.98 | 0.46 |
| 1:B:240:ASP:HA | 1:B:243:HIS:HB2 | 1.98 | 0.46 |
| 1:B:393:LYS:HZ2 | 1:B:398:PHE:HB2 | 1.80 | 0.46 |
| 1:B:567:GLU:H | 1:B:567:GLU:CD | 2.19 | 0.46 |
| 1:D:542:LEU:HA | 1:D:542:LEU:HD12 | 1.70 | 0.46 |
| 1:D:632:TRP:HA | 1:D:635:LYS:NZ | 2.31 | 0.46 |
| 1:A:251:HIS:O | 1:A:280:SER:OG | 2.33 | 0.46 |
| 1:A:530:LEU:HD21 | 1:A:555:MET:HE1 | 1.98 | 0.46 |
| 1:B:163:LYS:HD3 | 1:B:169:THR:HG23 | 1.97 | 0.46 |
| 1:C:180:GLN:HA | 1:C:183:MET:HG2 | 1.98 | 0.46 |
| 1:C:240:ASP:HA | 1:C:243:HIS:HB2 | 1.98 | 0.46 |
| 1:C:567:GLU:H | 1:C:567:GLU:CD | 2.19 | 0.46 |
| 1:D:202:LEU:HA | 1:D:205:LEU:HB2 | 1.98 | 0.46 |
| 1:D:228:ALA:O | 1:D:232:ARG:HG3 | 2.16 | 0.46 |
| 1:D:667:ARG:HB3 | 1:D:671:TRP:CH2 | 2.51 | 0.46 |
| 1:A:503:MET:SD | 1:A:503:MET:N | 2.89 | 0.46 |
| 1:B:152:ILE:HG13 | 1:B:153:MET:N | 2.30 | 0.45 |
| 1:B:202:LEU:HA | 1:B:205:LEU:HB2 | 1.98 | 0.45 |
| 1:B:210:LYS:HB3 | 1:B:210:LYS:HE2 | 1.61 | 0.45 |
| 1:B:667:ARG:HH21 | 1:A:655:GLU:HG3 | 1.80 | 0.45 |
| 1:C:503:MET:SD | 1:C:503:MET:N | 2.89 | 0.45 |
| 1:A:567:GLU:H | 1:A:567:GLU:CD | 2.19 | 0.45 |
| 1:C:185:ARG:HA | 1:C:188:HIS:CE1 | 2.50 | 0.45 |
| 1:C:228:ALA:O | 1:C:232:ARG:HG3 | 2.16 | 0.45 |
| 1:C:419:SER:OG | 1:C:434:GLU:HB2 | 2.16 | 0.45 |
| 1:D:503:MET:SD | 1:D:503:MET:N | 2.89 | 0.45 |
| 1:A:202:LEU:HA | 1:A:205:LEU:HB2 | 1.98 | 0.45 |
| 1:A:299:GLY:O | 1:A:302:LYS:HG2 | 2.16 | 0.45 |
| 1:A:397:PHE:HB2 | 1:A:436:PHE:CE2 | 2.51 | 0.45 |
| 1:B:299:GLY:O | 1:B:302:LYS:HG2 | 2.15 | 0.45 |
| 1:B:684:CYS:O | 1:B:687:LEU:HD23 | 2.16 | 0.45 |
| 1:D:452:ASP:OD1 | 1:D:453:LYS:N | 2.49 | 0.45 |
| 1:D:684:CYS:O | 1:D:687:LEU:HD23 | 2.16 | 0.45 |
| 1:A:228:ALA:O | 1:A:232:ARG:HG3 | 2.16 | 0.45 |
| 1:A:393:LYS:NZ | 1:A:398:PHE:HB2 | 2.32 | 0.45 |
| 1:A:719:ARG:O | 1:A:723:GLN:HG2 | 2.16 | 0.45 |
| 1:B:679:LEU:HD13 | 1:B:679:LEU:HA | 1.73 | 0.45 |
| 1:C:376:LEU:HD12 | 1:C:376:LEU:HA | 1.77 | 0.45 |
| 1:C:684:CYS:O | 1:C:687:LEU:HD23 | 2.16 | 0.45 |
| 1:D:210:LYS:HE2 | 1:D:210:LYS:HB3 | 1.61 | 0.45 |
| 1:D:262:GLU:OE1 | 1:D:262:GLU:N | 2.50 | 0.45 |

Continued on next page...

For Manuscript Review

*Continued from previous page...*

| Atom-1 | Atom-2 | Interatomic distance (Å) | Clash overlap (Å) |
|---|---|---|---|
| 1:D:567:GLU:OE2 | 1:D:567:GLU:N | 2.45 | 0.45 |
| 1:A:180:GLN:HA | 1:A:183:MET:HG2 | 1.98 | 0.45 |
| 1:B:452:ASP:OD1 | 1:B:453:LYS:N | 2.49 | 0.45 |
| 1:C:342:CYS:O | 1:C:346:ILE:HG12 | 2.17 | 0.45 |
| 1:D:152:ILE:HG21 | 1:D:186:LEU:HD21 | 1.99 | 0.45 |
| 1:A:722:LEU:HD23 | 1:A:722:LEU:HA | 1.73 | 0.45 |
| 1:B:111:LEU:HD21 | 1:B:161:ALA:HA | 1.99 | 0.45 |
| 1:C:152:ILE:HG13 | 1:C:153:MET:N | 2.30 | 0.45 |
| 1:C:397:PHE:HB2 | 1:C:436:PHE:CE2 | 2.51 | 0.45 |
| 1:C:629:LEU:O | 1:C:633:THR:HG23 | 2.17 | 0.45 |
| 1:D:299:GLY:HA2 | 1:D:302:LYS:HG2 | 1.99 | 0.45 |
| 1:D:629:LEU:O | 1:D:633:THR:HG23 | 2.17 | 0.45 |
| 1:B:303:LYS:HB2 | 1:B:303:LYS:HZ3 | 1.82 | 0.45 |
| 1:D:111:LEU:HD21 | 1:D:161:ALA:HA | 1.99 | 0.45 |
| 1:A:393:LYS:HZ2 | 1:A:398:PHE:HB2 | 1.81 | 0.45 |
| 1:A:629:LEU:O | 1:A:633:THR:HG23 | 2.17 | 0.45 |
| 1:B:152:ILE:HG21 | 1:B:186:LEU:HD21 | 1.99 | 0.45 |
| 1:D:148:MET:HA | 1:D:148:MET:HE2 | 1.98 | 0.45 |
| 1:D:180:GLN:HA | 1:D:183:MET:HG2 | 1.98 | 0.45 |
| 1:D:419:SER:O | 1:D:422:LYS:NZ | 2.36 | 0.45 |
| 1:D:715:GLN:O | 1:D:718:THR:OG1 | 2.29 | 0.45 |
| 1:A:342:CYS:O | 1:A:346:ILE:HG12 | 2.17 | 0.45 |
| 1:C:262:GLU:N | 1:C:262:GLU:OE1 | 2.50 | 0.45 |
| 1:C:472:TRP:O | 1:C:475:SER:OG | 2.35 | 0.45 |
| 1:A:162:LEU:HD11 | 1:A:172:CYS:HB2 | 1.98 | 0.45 |
| 1:B:342:CYS:O | 1:B:346:ILE:HG12 | 2.17 | 0.44 |
| 1:B:393:LYS:NZ | 1:B:398:PHE:HB2 | 2.32 | 0.44 |
| 1:B:655:GLU:OE2 | 1:B:660:ARG:HB2 | 2.17 | 0.44 |
| 1:B:667:ARG:N | 1:B:668:PRO:HD2 | 2.33 | 0.44 |
| 1:A:684:CYS:O | 1:A:687:LEU:HD23 | 2.16 | 0.44 |
| 1:B:223:LEU:HB3 | 1:B:224:LYS:HZ2 | 1.82 | 0.44 |
| 1:C:251:HIS:O | 1:C:280:SER:OG | 2.33 | 0.44 |
| 1:D:571:ALA:HA | 1:D:574:ARG:HB2 | 1.99 | 0.44 |
| 1:A:111:LEU:HD21 | 1:A:161:ALA:HA | 1.99 | 0.44 |
| 1:A:262:GLU:N | 1:A:262:GLU:OE1 | 2.50 | 0.44 |
| 1:A:472:TRP:O | 1:A:475:SER:OG | 2.35 | 0.44 |
| 1:B:262:GLU:OE1 | 1:B:262:GLU:N | 2.50 | 0.44 |
| 1:C:162:LEU:HD11 | 1:C:172:CYS:HB2 | 1.99 | 0.44 |
| 1:C:667:ARG:N | 1:C:668:PRO:HD2 | 2.32 | 0.44 |
| 1:A:667:ARG:N | 1:A:668:PRO:HD2 | 2.33 | 0.44 |
| 1:B:266:LYS:HB3 | 1:B:266:LYS:HE3 | 1.84 | 0.44 |

*Continued on next page...*

For Manuscript Review

*Continued from previous page...*

| Atom-1 | Atom-2 | Interatomic distance (Å) | Clash overlap (Å) |
|---|---|---|---|
| 1:B:571:ALA:HA | 1:B:574:ARG:HB2 | 1.99 | 0.44 |
| 1:D:626:LYS:HE2 | 1:D:626:LYS:HB3 | 1.77 | 0.44 |
| 1:A:571:ALA:HA | 1:A:574:ARG:HB2 | 1.99 | 0.44 |
| 1:A:755:ASP:OD1 | 1:A:756:SER:N | 2.51 | 0.44 |
| 1:C:111:LEU:HD21 | 1:C:161:ALA:HA | 1.99 | 0.44 |
| 1:C:521:ASN:O | 1:C:524:LYS:HG2 | 2.18 | 0.44 |
| 1:A:521:ASN:O | 1:A:524:LYS:HG2 | 2.18 | 0.44 |
| 1:B:552:THR:O | 1:B:556:THR:HG23 | 2.18 | 0.44 |
| 1:C:428:GLY:O | 1:C:432:ARG:HG3 | 2.18 | 0.44 |
| 1:C:635:LYS:HE3 | 1:C:635:LYS:HB2 | 1.36 | 0.44 |
| 1:D:552:THR:O | 1:D:556:THR:HG23 | 2.18 | 0.44 |
| 1:A:428:GLY:O | 1:A:432:ARG:HG3 | 2.18 | 0.44 |
| 1:B:321:TRP:HB3 | 1:B:506:PHE:CE2 | 2.53 | 0.44 |
| 1:B:530:LEU:HD21 | 1:B:555:MET:HE1 | 2.00 | 0.44 |
| 1:B:629:LEU:O | 1:B:633:THR:HG23 | 2.17 | 0.44 |
| 1:C:552:THR:O | 1:C:556:THR:HG23 | 2.18 | 0.44 |
| 1:C:755:ASP:OD1 | 1:C:756:SER:N | 2.51 | 0.44 |
| 1:D:342:CYS:O | 1:D:346:ILE:HG12 | 2.17 | 0.44 |
| 1:A:321:TRP:CE2 | 1:A:371:MET:HE1 | 2.53 | 0.44 |
| 1:B:162:LEU:HD11 | 1:B:172:CYS:HB2 | 1.99 | 0.44 |
| 1:B:567:GLU:OE2 | 1:B:567:GLU:N | 2.45 | 0.44 |
| 1:B:618:SER:CB | 1:C:619:GLY:HA3 | 2.47 | 0.44 |
| 1:C:266:LYS:HE3 | 1:C:266:LYS:HB3 | 1.84 | 0.44 |
| 1:C:393:LYS:NZ | 1:C:398:PHE:HB2 | 2.32 | 0.44 |
| 1:D:162:LEU:HD11 | 1:D:172:CYS:HB2 | 1.99 | 0.44 |
| 1:D:567:GLU:H | 1:D:567:GLU:CD | 2.19 | 0.44 |
| 1:D:655:GLU:OE2 | 1:D:660:ARG:HB2 | 2.17 | 0.44 |
| 1:A:152:ILE:HG21 | 1:A:186:LEU:HD21 | 1.99 | 0.44 |
| 1:B:268:ARG:H | 1:B:268:ARG:HG2 | 1.68 | 0.43 |
| 1:B:376:LEU:HD12 | 1:B:376:LEU:HA | 1.77 | 0.43 |
| 1:B:428:GLY:O | 1:B:432:ARG:HG3 | 2.18 | 0.43 |
| 1:B:605:GLU:CD | 1:B:605:GLU:N | 2.71 | 0.43 |
| 1:C:571:ALA:HA | 1:C:574:ARG:HB2 | 1.99 | 0.43 |
| 1:C:605:GLU:CD | 1:C:605:GLU:N | 2.71 | 0.43 |
| 1:C:722:LEU:HA | 1:C:722:LEU:HD23 | 1.73 | 0.43 |
| 1:A:250:LEU:HD11 | 1:A:267:LEU:HD23 | 2.00 | 0.43 |
| 1:A:303:LYS:HZ3 | 1:A:303:LYS:HB2 | 1.82 | 0.43 |
| 1:B:340:GLU:HA | 1:B:343:LEU:HB2 | 2.00 | 0.43 |
| 1:C:152:ILE:HG21 | 1:C:186:LEU:HD21 | 1.99 | 0.43 |
| 1:C:202:LEU:HA | 1:C:205:LEU:HB2 | 1.99 | 0.43 |
| 1:C:699:SER:HB2 | 1:C:702:LYS:HE2 | 2.00 | 0.43 |

*Continued on next page...*

For Manuscript Review

*Continued from previous page...*

| Atom-1 | Atom-2 | Interatomic distance (Å) | Clash overlap (Å) |
|---|---|---|---|
| 1:D:521:ASN:O | 1:D:524:LYS:HG2 | 2.18 | 0.43 |
| 1:D:605:GLU:CD | 1:D:605:GLU:N | 2.71 | 0.43 |
| 1:C:220:GLU:H | 1:C:224:LYS:NZ | 2.16 | 0.43 |
| 1:C:473:LYS:HE2 | 1:C:711:ASP:OD2 | 2.19 | 0.43 |
| 1:D:376:LEU:HD12 | 1:D:376:LEU:HA | 1.77 | 0.43 |
| 1:D:428:GLY:O | 1:D:432:ARG:HG3 | 2.18 | 0.43 |
| 1:D:472:TRP:O | 1:D:475:SER:OG | 2.35 | 0.43 |
| 1:D:755:ASP:OD1 | 1:D:756:SER:N | 2.51 | 0.43 |
| 1:A:220:GLU:H | 1:A:224:LYS:NZ | 2.16 | 0.43 |
| 1:A:655:GLU:OE2 | 1:A:660:ARG:HB2 | 2.17 | 0.43 |
| 1:B:200:ILE:HG22 | 1:B:255:GLY:HA3 | 2.01 | 0.43 |
| 1:B:472:TRP:O | 1:B:475:SER:OG | 2.35 | 0.43 |
| 1:C:223:LEU:HD11 | 1:C:227:TYR:CZ | 2.53 | 0.43 |
| 1:C:655:GLU:OE2 | 1:C:660:ARG:HB2 | 2.17 | 0.43 |
| 1:D:234:LEU:HD23 | 1:D:237:ILE:HD11 | 2.01 | 0.43 |
| 1:A:137:ASP:HB2 | 1:A:178:PHE:CE1 | 2.54 | 0.43 |
| 1:C:503:MET:O | 1:C:507:CYS:HB2 | 2.19 | 0.43 |
| 1:C:530:LEU:HD21 | 1:C:555:MET:HE1 | 1.99 | 0.43 |
| 1:D:200:ILE:HG22 | 1:D:255:GLY:HA3 | 2.01 | 0.43 |
| 1:D:220:GLU:H | 1:D:224:LYS:NZ | 2.16 | 0.43 |
| 1:D:503:MET:O | 1:D:507:CYS:HB2 | 2.19 | 0.43 |
| 1:A:200:ILE:HG22 | 1:A:255:GLY:HA3 | 2.01 | 0.43 |
| 1:A:321:TRP:HB3 | 1:A:506:PHE:CE2 | 2.53 | 0.43 |
| 1:A:752:GLU:H | 1:A:752:GLU:CD | 2.22 | 0.43 |
| 1:B:227:TYR:HD1 | 1:B:227:TYR:HA | 1.72 | 0.43 |
| 1:B:343:LEU:HD23 | 1:B:343:LEU:HA | 1.85 | 0.43 |
| 1:C:589:SER:HB2 | 1:C:592:ARG:HH21 | 1.84 | 0.43 |
| 1:C:605:GLU:HB3 | 1:C:629:LEU:HD12 | 2.00 | 0.43 |
| 1:D:321:TRP:HB3 | 1:D:506:PHE:CE2 | 2.53 | 0.43 |
| 1:D:393:LYS:NZ | 1:D:398:PHE:HB2 | 2.32 | 0.43 |
| 1:D:623:GLU:HA | 1:D:626:LYS:HE3 | 2.01 | 0.43 |
| 1:D:699:SER:HB2 | 1:D:702:LYS:HE2 | 2.00 | 0.43 |
| 1:A:210:LYS:HB3 | 1:A:210:LYS:HE2 | 1.61 | 0.43 |
| 1:A:503:MET:O | 1:A:507:CYS:HB2 | 2.19 | 0.43 |
| 1:A:552:THR:O | 1:A:556:THR:HG23 | 2.18 | 0.43 |
| 1:C:125:SER:HA | 1:C:128:ASP:OD2 | 2.19 | 0.43 |
| 1:A:223:LEU:HD11 | 1:A:227:TYR:CZ | 2.54 | 0.43 |
| 1:A:340:GLU:HA | 1:A:343:LEU:HB2 | 2.00 | 0.43 |
| 1:A:473:LYS:HE2 | 1:A:711:ASP:OD2 | 2.19 | 0.43 |
| 1:A:605:GLU:HB3 | 1:A:629:LEU:HD12 | 2.00 | 0.43 |
| 1:B:236:LYS:HB3 | 1:B:236:LYS:HE3 | 1.56 | 0.43 |

*Continued on next page...*

For Manuscript Review

*Continued from previous page...*

| Atom-1 | Atom-2 | Interatomic distance (Å) | Clash overlap (Å) |
|---|---|---|---|
| 1:B:503:MET:O | 1:B:507:CYS:HB2 | 2.19 | 0.43 |
| 1:B:643:GLU:O | 1:B:646:THR:OG1 | 2.23 | 0.43 |
| 1:C:178:PHE:O | 1:C:182:ASN:ND2 | 2.39 | 0.43 |
| 1:C:321:TRP:HB3 | 1:C:506:PHE:CE2 | 2.53 | 0.43 |
| 1:C:567:GLU:OE2 | 1:C:567:GLU:N | 2.45 | 0.43 |
| 1:C:701:GLU:H | 1:C:701:GLU:HG3 | 1.61 | 0.43 |
| 1:A:266:LYS:HE3 | 1:A:266:LYS:HB3 | 1.84 | 0.43 |
| 1:B:223:LEU:HD11 | 1:B:227:TYR:CZ | 2.54 | 0.43 |
| 1:B:719:ARG:HD2 | 1:B:723:GLN:HE21 | 1.84 | 0.43 |
| 1:C:220:GLU:H | 1:C:224:LYS:HZ1 | 1.66 | 0.43 |
| 1:C:325:ARG:O | 1:C:329:LEU:N | 2.47 | 0.43 |
| 1:D:701:GLU:H | 1:D:701:GLU:HG3 | 1.61 | 0.43 |
| 1:A:223:LEU:HB3 | 1:A:224:LYS:HZ2 | 1.84 | 0.43 |
| 1:B:671:TRP:CD1 | 1:A:652:ARG:NE | 2.87 | 0.43 |
| 1:B:715:GLN:O | 1:B:718:THR:OG1 | 2.29 | 0.43 |
| 1:B:755:ASP:OD1 | 1:B:756:SER:N | 2.51 | 0.43 |
| 1:C:200:ILE:HG22 | 1:C:255:GLY:HA3 | 2.01 | 0.43 |
| 1:D:340:GLU:HA | 1:D:343:LEU:HB2 | 2.00 | 0.43 |
| 1:D:731:LEU:HA | 1:D:734:GLU:OE1 | 2.19 | 0.43 |
| 1:A:699:SER:HB2 | 1:A:702:LYS:HE2 | 2.00 | 0.43 |
| 1:B:137:ASP:HB2 | 1:B:178:PHE:CE1 | 2.54 | 0.42 |
| 1:B:299:GLY:HA2 | 1:B:302:LYS:HG2 | 2.01 | 0.42 |
| 1:B:328:ARG:HD2 | 1:B:484:TYR:CD1 | 2.54 | 0.42 |
| 1:B:393:LYS:NZ | 1:B:400:LEU:O | 2.49 | 0.42 |
| 1:D:137:ASP:HB2 | 1:D:178:PHE:CE1 | 2.54 | 0.42 |
| 1:D:250:LEU:HD11 | 1:D:267:LEU:HD23 | 2.00 | 0.42 |
| 1:A:178:PHE:O | 1:A:182:ASN:ND2 | 2.39 | 0.42 |
| 1:B:245:MET:O | 1:B:247:THR:HG23 | 2.19 | 0.42 |
| 1:B:632:TRP:CA | 1:B:635:LYS:HE2 | 2.48 | 0.42 |
| 1:B:699:SER:HB2 | 1:B:702:LYS:HE2 | 2.00 | 0.42 |
| 1:C:635:LYS:HZ2 | 1:C:642:ILE:CG2 | 2.27 | 0.42 |
| 1:D:125:SER:HA | 1:D:128:ASP:OD2 | 2.19 | 0.42 |
| 1:D:223:LEU:HD11 | 1:D:227:TYR:CZ | 2.54 | 0.42 |
| 1:D:245:MET:O | 1:D:247:THR:HG23 | 2.19 | 0.42 |
| 1:D:473:LYS:HE2 | 1:D:711:ASP:OD2 | 2.19 | 0.42 |
| 1:B:125:SER:HA | 1:B:128:ASP:OD2 | 2.19 | 0.42 |
| 1:B:178:PHE:O | 1:B:182:ASN:ND2 | 2.39 | 0.42 |
| 1:B:521:ASN:O | 1:B:524:LYS:HG2 | 2.18 | 0.42 |
| 1:C:137:ASP:HB2 | 1:C:178:PHE:CE1 | 2.54 | 0.42 |
| 1:C:250:LEU:HD11 | 1:C:267:LEU:HD23 | 2.00 | 0.42 |
| 1:C:340:GLU:HA | 1:C:343:LEU:HB2 | 2.00 | 0.42 |

*Continued on next page...*

For Manuscript Review

Continued from previous page...

| Atom-1 | Atom-2 | Interatomic distance (Å) | Clash overlap (Å) |
|---|---|---|---|
| 1:C:510:LEU:HA | 1:C:513:LYS:HG2 | 2.01 | 0.42 |
| 1:C:623:GLU:HA | 1:C:626:LYS:HE3 | 2.01 | 0.42 |
| 1:C:752:GLU:H | 1:C:752:GLU:CD | 2.22 | 0.42 |
| 1:B:516:LYS:O | 1:B:520:SER:OG | 2.34 | 0.42 |
| 1:C:719:ARG:HD2 | 1:C:723:GLN:HE21 | 1.84 | 0.42 |
| 1:C:753:THR:O | 1:C:756:SER:OG | 2.25 | 0.42 |
| 1:D:605:GLU:HB3 | 1:D:629:LEU:HD12 | 2.00 | 0.42 |
| 1:A:485:LEU:HD23 | 1:A:485:LEU:HA | 1.86 | 0.42 |
| 1:A:719:ARG:HD2 | 1:A:723:GLN:HE21 | 1.84 | 0.42 |
| 1:A:731:LEU:HA | 1:A:734:GLU:OE1 | 2.19 | 0.42 |
| 1:B:220:GLU:H | 1:B:224:LYS:NZ | 2.16 | 0.42 |
| 1:B:623:GLU:HA | 1:B:626:LYS:HE3 | 2.01 | 0.42 |
| 1:B:753:THR:O | 1:B:756:SER:OG | 2.25 | 0.42 |
| 1:C:472:TRP:HE1 | 1:C:718:THR:HG21 | 1.85 | 0.42 |
| 1:D:526:CYS:HA | 1:D:529:GLU:HG2 | 2.02 | 0.42 |
| 1:A:343:LEU:HD23 | 1:A:343:LEU:HA | 1.85 | 0.42 |
| 1:A:626:LYS:HB3 | 1:A:626:LYS:HE2 | 1.77 | 0.42 |
| 1:B:260:ASP:OD2 | 1:B:263:LYS:N | 2.50 | 0.42 |
| 1:B:510:LEU:HA | 1:B:513:LYS:HG2 | 2.01 | 0.42 |
| 1:B:526:CYS:HA | 1:B:529:GLU:HG2 | 2.02 | 0.42 |
| 1:B:671:TRP:CH2 | 1:A:624:ALA:HB2 | 2.54 | 0.42 |
| 1:C:234:LEU:HD23 | 1:C:237:ILE:HD11 | 2.01 | 0.42 |
| 1:D:472:TRP:HE1 | 1:D:718:THR:HG21 | 1.85 | 0.42 |
| 1:A:234:LEU:HD23 | 1:A:237:ILE:HD11 | 2.01 | 0.42 |
| 1:A:328:ARG:HD2 | 1:A:484:TYR:CD1 | 2.54 | 0.42 |
| 1:A:623:GLU:HA | 1:A:626:LYS:HE3 | 2.01 | 0.42 |
| 1:B:250:LEU:HD11 | 1:B:267:LEU:HD23 | 2.00 | 0.42 |
| 1:B:423:PHE:HB2 | 1:B:426:ASP:OD2 | 2.20 | 0.42 |
| 1:B:589:SER:HB2 | 1:B:592:ARG:HH21 | 1.84 | 0.42 |
| 1:B:605:GLU:HB3 | 1:B:629:LEU:HD12 | 2.00 | 0.42 |
| 1:D:510:LEU:HA | 1:D:513:LYS:HG2 | 2.01 | 0.42 |
| 1:D:757:HIS:O | 1:D:761:VAL:HG12 | 2.20 | 0.42 |
| 1:A:125:SER:HA | 1:A:128:ASP:OD2 | 2.19 | 0.42 |
| 1:A:260:ASP:OD2 | 1:A:263:LYS:N | 2.50 | 0.42 |
| 1:A:325:ARG:O | 1:A:329:LEU:N | 2.48 | 0.42 |
| 1:A:510:LEU:HA | 1:A:513:LYS:HG2 | 2.01 | 0.42 |
| 1:B:473:LYS:HE2 | 1:B:711:ASP:OD2 | 2.19 | 0.42 |
| 1:C:328:ARG:HD2 | 1:C:484:TYR:CD1 | 2.54 | 0.42 |
| 1:C:494:VAL:HA | 1:C:502:ARG:HG2 | 2.02 | 0.42 |
| 1:C:635:LYS:NZ | 1:C:642:ILE:HA | 2.34 | 0.42 |
| 1:D:178:PHE:O | 1:D:182:ASN:ND2 | 2.39 | 0.42 |

Continued on next page...

For Manuscript Review

*Continued from previous page...*

| Atom-1 | Atom-2 | Interatomic distance (Å) | Clash overlap (Å) |
|---|---|---|---|
| 1:D:589:SER:HB2 | 1:D:592:ARG:HH21 | 1.84 | 0.42 |
| 1:B:626:LYS:HB3 | 1:B:626:LYS:HE2 | 1.77 | 0.42 |
| 1:C:281:THR:HB | 1:C:293:CYS:SG | 2.60 | 0.42 |
| 1:C:423:PHE:HB2 | 1:C:426:ASP:OD2 | 2.20 | 0.42 |
| 1:C:526:CYS:HA | 1:C:529:GLU:HG2 | 2.02 | 0.42 |
| 1:D:271:ASP:N | 1:D:271:ASP:OD1 | 2.53 | 0.42 |
| 1:D:281:THR:HB | 1:D:293:CYS:SG | 2.60 | 0.42 |
| 1:A:80:THR:OG1 | 1:A:82:THR:HG23 | 2.20 | 0.42 |
| 1:A:542:LEU:HD12 | 1:A:542:LEU:HA | 1.70 | 0.42 |
| 1:A:589:SER:HB2 | 1:A:592:ARG:HH21 | 1.84 | 0.42 |
| 1:A:635:LYS:HB2 | 1:A:635:LYS:HE3 | 1.40 | 0.42 |
| 1:B:91:LEU:O | 1:B:94:ILE:HG12 | 2.20 | 0.42 |
| 1:B:731:LEU:HA | 1:B:734:GLU:OE1 | 2.19 | 0.42 |
| 1:C:85:ASP:O | 1:C:89:THR:HG23 | 2.20 | 0.42 |
| 1:D:220:GLU:C | 1:D:224:LYS:HZ3 | 2.21 | 0.42 |
| 1:A:245:MET:O | 1:A:247:THR:HG23 | 2.19 | 0.42 |
| 1:B:234:LEU:HD23 | 1:B:237:ILE:HD11 | 2.01 | 0.41 |
| 1:B:516:LYS:HE3 | 1:B:767:ILE:HG22 | 2.02 | 0.41 |
| 1:C:589:SER:HA | 1:C:592:ARG:NE | 2.35 | 0.41 |
| 1:C:731:LEU:HA | 1:C:734:GLU:OE1 | 2.19 | 0.41 |
| 1:D:85:ASP:O | 1:D:89:THR:HG23 | 2.20 | 0.41 |
| 1:A:454:TRP:HA | 1:A:496:ILE:HB | 2.03 | 0.41 |
| 1:A:472:TRP:HE1 | 1:A:718:THR:HG21 | 1.85 | 0.41 |
| 1:B:590:HIS:CE1 | 1:B:594:ILE:HD13 | 2.55 | 0.41 |
| 1:B:652:ARG:O | 1:B:656:ILE:HD12 | 2.21 | 0.41 |
| 1:C:245:MET:O | 1:C:247:THR:HG23 | 2.19 | 0.41 |
| 1:C:271:ASP:OD1 | 1:C:271:ASP:N | 2.53 | 0.41 |
| 1:C:757:HIS:O | 1:C:761:VAL:HG12 | 2.20 | 0.41 |
| 1:D:423:PHE:HB2 | 1:D:426:ASP:OD2 | 2.20 | 0.41 |
| 1:D:752:GLU:H | 1:D:752:GLU:CD | 2.22 | 0.41 |
| 1:A:91:LEU:O | 1:A:94:ILE:HG12 | 2.20 | 0.41 |
| 1:A:393:LYS:NZ | 1:A:400:LEU:O | 2.49 | 0.41 |
| 1:C:91:LEU:O | 1:C:94:ILE:HG12 | 2.20 | 0.41 |
| 1:C:319:ARG:HD3 | 1:C:346:ILE:HD11 | 2.02 | 0.41 |
| 1:C:454:TRP:HA | 1:C:496:ILE:HB | 2.03 | 0.41 |
| 1:C:518:ASP:HA | 1:C:521:ASN:ND2 | 2.35 | 0.41 |
| 1:D:454:TRP:HA | 1:D:496:ILE:HB | 2.03 | 0.41 |
| 1:D:719:ARG:HD2 | 1:D:723:GLN:HE21 | 1.84 | 0.41 |
| 1:A:281:THR:HB | 1:A:293:CYS:SG | 2.60 | 0.41 |
| 1:B:115:LEU:H | 1:B:115:LEU:HG | 1.45 | 0.41 |
| 1:B:281:THR:HB | 1:B:293:CYS:SG | 2.60 | 0.41 |

*Continued on next page...*

For Manuscript Review

Continued from previous page...

| Atom-1 | Atom-2 | Interatomic distance (Å) | Clash overlap (Å) |
|---|---|---|---|
| 1:B:737:LEU:HD23 | 1:B:737:LEU:HA | 1.76 | 0.41 |
| 1:B:752:GLU:H | 1:B:752:GLU:CD | 2.22 | 0.41 |
| 1:C:393:LYS:NZ | 1:C:400:LEU:O | 2.49 | 0.41 |
| 1:C:632:TRP:HA | 1:C:635:LYS:HE2 | 2.02 | 0.41 |
| 1:D:494:VAL:HA | 1:D:502:ARG:HG2 | 2.02 | 0.41 |
| 1:D:590:HIS:CE1 | 1:D:594:ILE:HD13 | 2.55 | 0.41 |
| 1:A:590:HIS:CE1 | 1:A:594:ILE:HD13 | 2.55 | 0.41 |
| 1:C:605:GLU:HA | 1:C:625:TRP:HE1 | 1.85 | 0.41 |
| 1:C:626:LYS:HB3 | 1:C:626:LYS:HE2 | 1.77 | 0.41 |
| 1:C:652:ARG:O | 1:C:656:ILE:HD12 | 2.21 | 0.41 |
| 1:D:328:ARG:HD2 | 1:D:484:TYR:CD1 | 2.54 | 0.41 |
| 1:A:299:GLY:HA2 | 1:A:302:LYS:HG2 | 2.01 | 0.41 |
| 1:A:757:HIS:O | 1:A:761:VAL:HG12 | 2.20 | 0.41 |
| 1:B:518:ASP:HA | 1:B:521:ASN:ND2 | 2.35 | 0.41 |
| 1:C:635:LYS:HZ1 | 1:C:642:ILE:HG22 | 1.82 | 0.41 |
| 1:D:298:ASP:O | 1:D:302:LYS:N | 2.53 | 0.41 |
| 1:D:652:ARG:O | 1:D:656:ILE:HD12 | 2.21 | 0.41 |
| 1:A:85:ASP:O | 1:A:89:THR:HG23 | 2.20 | 0.41 |
| 1:A:338:GLU:H | 1:A:338:GLU:HG3 | 1.47 | 0.41 |
| 1:A:376:LEU:HA | 1:A:376:LEU:HD12 | 1.77 | 0.41 |
| 1:A:589:SER:HA | 1:A:592:ARG:NE | 2.35 | 0.41 |
| 1:B:227:TYR:O | 1:B:231:GLU:HG2 | 2.21 | 0.41 |
| 1:B:589:SER:HA | 1:B:592:ARG:NE | 2.35 | 0.41 |
| 1:B:605:GLU:HA | 1:B:625:TRP:HE1 | 1.85 | 0.41 |
| 1:D:114:ARG:HB3 | 1:D:118:GLY:HA3 | 2.03 | 0.41 |
| 1:D:319:ARG:HD3 | 1:D:346:ILE:HD11 | 2.02 | 0.41 |
| 1:D:417:ARG:O | 1:D:420:GLN:HG2 | 2.21 | 0.41 |
| 1:D:589:SER:HA | 1:D:592:ARG:NE | 2.35 | 0.41 |
| 1:A:114:ARG:HB3 | 1:A:118:GLY:HA3 | 2.03 | 0.41 |
| 1:A:423:PHE:HB2 | 1:A:426:ASP:OD2 | 2.20 | 0.41 |
| 1:A:516:LYS:HE3 | 1:A:767:ILE:HG22 | 2.02 | 0.41 |
| 1:B:80:THR:OG1 | 1:B:82:THR:HG23 | 2.20 | 0.41 |
| 1:B:85:ASP:O | 1:B:89:THR:HG23 | 2.20 | 0.41 |
| 1:B:454:TRP:HA | 1:B:496:ILE:HB | 2.03 | 0.41 |
| 1:B:538:LEU:HD21 | 1:D:344:ASP:HB2 | 2.03 | 0.41 |
| 1:B:722:LEU:HD23 | 1:B:722:LEU:HA | 1.73 | 0.41 |
| 1:D:80:THR:OG1 | 1:D:82:THR:HG23 | 2.20 | 0.41 |
| 1:D:466:TYR:CE2 | 1:D:480:GLU:HG3 | 2.56 | 0.41 |
| 1:D:518:ASP:HA | 1:D:521:ASN:ND2 | 2.35 | 0.41 |
| 1:A:411:PRO:O | 1:A:415:THR:OG1 | 2.32 | 0.41 |
| 1:A:526:CYS:HA | 1:A:529:GLU:HG2 | 2.02 | 0.41 |

Continued on next page...

For Manuscript Review

Continued from previous page...

| Atom-1 | Atom-2 | Interatomic distance (Å) | Clash overlap (Å) |
|---|---|---|---|
| 1:B:114:ARG:HB3 | 1:B:118:GLY:HA3 | 2.03 | 0.41 |
| 1:B:242:LEU:HA | 1:B:242:LEU:HD23 | 1.90 | 0.41 |
| 1:B:494:VAL:HA | 1:B:502:ARG:HG2 | 2.02 | 0.41 |
| 1:B:549:ASP:O | 1:B:553:THR:HG22 | 2.21 | 0.41 |
| 1:C:80:THR:OG1 | 1:C:82:THR:HG23 | 2.20 | 0.41 |
| 1:C:549:ASP:O | 1:C:553:THR:HG22 | 2.21 | 0.41 |
| 1:D:227:TYR:O | 1:D:231:GLU:HG2 | 2.21 | 0.41 |
| 1:D:441:LEU:HD23 | 1:D:441:LEU:HA | 1.91 | 0.41 |
| 1:D:516:LYS:HE3 | 1:D:767:ILE:HG22 | 2.02 | 0.41 |
| 1:D:597:PRO:O | 1:D:600:SER:HB3 | 2.21 | 0.41 |
| 1:A:140:TRP:HE1 | 1:A:158:CYS:HG | 1.63 | 0.41 |
| 1:A:227:TYR:O | 1:A:231:GLU:HG2 | 2.21 | 0.41 |
| 1:A:271:ASP:OD1 | 1:A:271:ASP:N | 2.53 | 0.41 |
| 1:A:319:ARG:HD3 | 1:A:346:ILE:HD11 | 2.02 | 0.41 |
| 1:A:417:ARG:O | 1:A:420:GLN:HG2 | 2.21 | 0.41 |
| 1:A:549:ASP:O | 1:A:553:THR:HG22 | 2.21 | 0.41 |
| 1:A:605:GLU:CD | 1:A:605:GLU:N | 2.71 | 0.41 |
| 1:A:679:LEU:HD13 | 1:A:679:LEU:HA | 1.73 | 0.41 |
| 1:B:466:TYR:CE2 | 1:B:480:GLU:HG3 | 2.56 | 0.41 |
| 1:C:679:LEU:HD13 | 1:C:679:LEU:HA | 1.73 | 0.41 |
| 1:D:419:SER:HA | 1:D:430:LEU:HD22 | 2.03 | 0.41 |
| 1:D:536:TRP:HE1 | 1:D:612:PRO:HD3 | 1.86 | 0.41 |
| 1:D:549:ASP:O | 1:D:553:THR:HG22 | 2.21 | 0.41 |
| 1:D:589:SER:HA | 1:D:592:ARG:HH21 | 1.86 | 0.41 |
| 1:D:721:VAL:HG13 | 1:D:733:ARG:HB3 | 2.03 | 0.41 |
| 1:A:518:ASP:HA | 1:A:521:ASN:ND2 | 2.35 | 0.41 |
| 1:B:417:ARG:O | 1:B:420:GLN:HG2 | 2.21 | 0.40 |
| 1:B:757:HIS:O | 1:B:761:VAL:HG12 | 2.20 | 0.40 |
| 1:C:245:MET:HA | 1:C:245:MET:CE | 2.52 | 0.40 |
| 1:C:590:HIS:CE1 | 1:C:594:ILE:HD13 | 2.55 | 0.40 |
| 1:C:597:PRO:O | 1:C:600:SER:HB3 | 2.21 | 0.40 |
| 1:C:690:ARG:HB2 | 1:C:706:ILE:HG21 | 2.03 | 0.40 |
| 1:D:91:LEU:O | 1:D:94:ILE:HG12 | 2.20 | 0.40 |
| 1:D:122:GLN:HG3 | 1:D:123:PHE:CD2 | 2.56 | 0.40 |
| 1:A:245:MET:CE | 1:A:245:MET:HA | 2.51 | 0.40 |
| 1:A:605:GLU:HA | 1:A:625:TRP:HE1 | 1.85 | 0.40 |
| 1:B:319:ARG:HD3 | 1:B:346:ILE:HD11 | 2.03 | 0.40 |
| 1:C:122:GLN:HG3 | 1:C:123:PHE:CD2 | 2.56 | 0.40 |
| 1:C:203:PRO:HG3 | 1:C:230:ARG:NE | 2.36 | 0.40 |
| 1:C:227:TYR:O | 1:C:231:GLU:HG2 | 2.21 | 0.40 |
| 1:C:299:GLY:HA2 | 1:C:302:LYS:CD | 2.51 | 0.40 |

Continued on next page...

For Manuscript Review

*Continued from previous page...*

| Atom-1 | Atom-2 | Interatomic distance (Å) | Clash overlap (Å) |
|---|---|---|---|
| 1:C:721:VAL:HG13 | 1:C:733:ARG:HB3 | 2.03 | 0.40 |
| 1:D:328:ARG:HD2 | 1:D:484:TYR:CE1 | 2.57 | 0.40 |
| 1:A:407:SER:C | 1:A:453:LYS:HZ1 | 2.24 | 0.40 |
| 1:B:229:GLU:HB3 | 1:B:233:LYS:NZ | 2.37 | 0.40 |
| 1:B:377:ARG:NH1 | 1:B:421:LEU:O | 2.48 | 0.40 |
| 1:B:472:TRP:HE1 | 1:B:718:THR:HG21 | 1.85 | 0.40 |
| 1:B:721:VAL:HG13 | 1:B:733:ARG:HB3 | 2.03 | 0.40 |
| 1:C:129:TRP:HA | 1:C:132:GLN:OE1 | 2.22 | 0.40 |
| 1:C:419:SER:O | 1:C:422:LYS:NZ | 2.36 | 0.40 |
| 1:C:466:TYR:CE2 | 1:C:480:GLU:HG3 | 2.56 | 0.40 |
| 1:D:758:ILE:HG23 | 1:D:762:ILE:HD12 | 2.03 | 0.40 |
| 1:A:129:TRP:HA | 1:A:132:GLN:OE1 | 2.22 | 0.40 |
| 1:A:494:VAL:HA | 1:A:502:ARG:HG2 | 2.02 | 0.40 |
| 1:A:632:TRP:HA | 1:A:635:LYS:NZ | 2.37 | 0.40 |
| 1:A:652:ARG:O | 1:A:656:ILE:HD12 | 2.21 | 0.40 |
| 1:A:690:ARG:HB2 | 1:A:706:ILE:HG21 | 2.04 | 0.40 |
| 1:B:223:LEU:HD23 | 1:B:224:LYS:HZ2 | 1.86 | 0.40 |
| 1:B:245:MET:HA | 1:B:245:MET:CE | 2.51 | 0.40 |
| 1:B:297:LEU:O | 1:B:301:VAL:HG12 | 2.20 | 0.40 |
| 1:B:297:LEU:O | 1:B:300:ILE:HG12 | 2.21 | 0.40 |
| 1:B:328:ARG:HD2 | 1:B:484:TYR:CE1 | 2.57 | 0.40 |
| 1:B:597:PRO:O | 1:B:600:SER:HB3 | 2.21 | 0.40 |
| 1:B:758:ILE:HG23 | 1:B:762:ILE:HD12 | 2.03 | 0.40 |
| 1:C:510:LEU:HA | 1:C:510:LEU:HD13 | 1.93 | 0.40 |
| 1:C:589:SER:HA | 1:C:592:ARG:HH21 | 1.86 | 0.40 |
| 1:D:245:MET:CE | 1:D:245:MET:HA | 2.51 | 0.40 |
| 1:A:393:LYS:O | 1:A:396:LYS:N | 2.54 | 0.40 |
| 1:A:670:LEU:H | 1:A:670:LEU:HG | 1.66 | 0.40 |
| 1:A:758:ILE:HG23 | 1:A:762:ILE:HD12 | 2.03 | 0.40 |
| 1:B:419:SER:HA | 1:B:430:LEU:HD22 | 2.03 | 0.40 |
| 1:B:536:TRP:HE1 | 1:B:612:PRO:HD3 | 1.86 | 0.40 |
| 1:B:701:GLU:H | 1:B:701:GLU:HG3 | 1.61 | 0.40 |
| 1:C:354:GLY:O | 1:C:355:LEU:HD23 | 2.22 | 0.40 |
| 1:D:207:ASP:O | 1:D:210:LYS:HG2 | 2.22 | 0.40 |
| 1:D:242:LEU:HD23 | 1:D:242:LEU:HA | 1.89 | 0.40 |
| 1:D:605:GLU:HA | 1:D:625:TRP:HE1 | 1.85 | 0.40 |
| 1:D:722:LEU:HA | 1:D:722:LEU:HD23 | 1.73 | 0.40 |
| 1:A:354:GLY:O | 1:A:355:LEU:HD23 | 2.22 | 0.40 |
| 1:A:466:TYR:CE2 | 1:A:480:GLU:HG3 | 2.56 | 0.40 |
| 1:A:589:SER:HA | 1:A:592:ARG:HH21 | 1.87 | 0.40 |

There are no symmetry-related clashes.

For Manuscript Review

## 5.3 Torsion angles ⓘ

### 5.3.1 Protein backbone ⓘ

In the following table, the Percentiles column shows the percent Ramachandran outliers of the chain as a percentile score with respect to all PDB entries followed by that with respect to all EM entries.

The Analysed column shows the number of residues for which the backbone conformation was analysed, and the total number of residues.

| Mol | Chain | Analysed | Favoured | Allowed | Outliers | Percentiles | |
|---|---|---|---|---|---|---|---|
| 1 | A | 686/775 (88%) | 647 (94%) | 39 (6%) | 0 | 100 | 100 |
| 1 | B | 686/775 (88%) | 647 (94%) | 39 (6%) | 0 | 100 | 100 |
| 1 | C | 686/775 (88%) | 647 (94%) | 39 (6%) | 0 | 100 | 100 |
| 1 | D | 686/775 (88%) | 646 (94%) | 40 (6%) | 0 | 100 | 100 |
| All | All | 2744/3100 (88%) | 2587 (94%) | 157 (6%) | 0 | 100 | 100 |

There are no Ramachandran outliers to report.

### 5.3.2 Protein sidechains ⓘ

In the following table, the Percentiles column shows the percent sidechain outliers of the chain as a percentile score with respect to all PDB entries followed by that with respect to all EM entries.

The Analysed column shows the number of residues for which the sidechain conformation was analysed, and the total number of residues.

| Mol | Chain | Analysed | Rotameric | Outliers | Percentiles | |
|---|---|---|---|---|---|---|
| 1 | A | 597/672 (89%) | 517 (87%) | 80 (13%) | 4 | 21 |
| 1 | B | 597/672 (89%) | 518 (87%) | 79 (13%) | 4 | 21 |
| 1 | C | 597/672 (89%) | 517 (87%) | 80 (13%) | 4 | 21 |
| 1 | D | 597/672 (89%) | 516 (86%) | 81 (14%) | 3 | 20 |
| All | All | 2388/2688 (89%) | 2068 (87%) | 320 (13%) | 7 | 21 |

All (320) residues with a non-rotameric sidechain are listed below:

| Mol | Chain | Res | Type |
|---|---|---|---|
| 1 | B | 81 | THR |
| 1 | B | 82 | THR |

*Continued on next page...*

For Manuscript Review

*Continued from previous page...*

| Mol | Chain | Res | Type |
|---|---|---|---|
| 1 | B | 96 | GLU |
| 1 | B | 100 | SER |
| 1 | B | 101 | ILE |
| 1 | B | 107 | SER |
| 1 | B | 111 | LEU |
| 1 | B | 115 | LEU |
| 1 | B | 125 | SER |
| 1 | B | 128 | ASP |
| 1 | B | 142 | ASP |
| 1 | B | 148 | MET |
| 1 | B | 156 | LEU |
| 1 | B | 158 | CYS |
| 1 | B | 163 | LYS |
| 1 | B | 188 | HIS |
| 1 | B | 191 | GLU |
| 1 | B | 194 | MET |
| 1 | B | 196 | VAL |
| 1 | B | 240 | ASP |
| 1 | B | 245 | MET |
| 1 | B | 248 | THR |
| 1 | B | 257 | VAL |
| 1 | B | 258 | ASP |
| 1 | B | 266 | LYS |
| 1 | B | 270 | LEU |
| 1 | B | 273 | SER |
| 1 | B | 280 | SER |
| 1 | B | 303 | LYS |
| 1 | B | 332 | SER |
| 1 | B | 337 | SER |
| 1 | B | 340 | GLU |
| 1 | B | 342 | CYS |
| 1 | B | 351 | THR |
| 1 | B | 353 | ASP |
| 1 | B | 363 | VAL |
| 1 | B | 378 | LEU |
| 1 | B | 399 | CYS |
| 1 | B | 400 | LEU |
| 1 | B | 414 | ASN |
| 1 | B | 419 | SER |
| 1 | B | 429 | VAL |
| 1 | B | 449 | ARG |
| 1 | B | 459 | ASP |

*Continued on next page...*

For Manuscript Review

*Continued from previous page...*

| Mol | Chain | Res | Type |
|---|---|---|---|
| 1 | B | 475 | SER |
| 1 | B | 483 | LEU |
| 1 | B | 493 | ASP |
| 1 | B | 520 | SER |
| 1 | B | 540 | SER |
| 1 | B | 549 | ASP |
| 1 | B | 554 | LEU |
| 1 | B | 583 | LEU |
| 1 | B | 587 | VAL |
| 1 | B | 589 | SER |
| 1 | B | 591 | PHE |
| 1 | B | 593 | ARG |
| 1 | B | 605 | GLU |
| 1 | B | 607 | LEU |
| 1 | B | 609 | SER |
| 1 | B | 610 | LEU |
| 1 | B | 615 | ASP |
| 1 | B | 618 | SER |
| 1 | B | 645 | ASP |
| 1 | B | 664 | THR |
| 1 | B | 670 | LEU |
| 1 | B | 674 | SER |
| 1 | B | 679 | LEU |
| 1 | B | 681 | SER |
| 1 | B | 685 | CYS |
| 1 | B | 687 | LEU |
| 1 | B | 688 | SER |
| 1 | B | 695 | GLU |
| 1 | B | 699 | SER |
| 1 | B | 701 | GLU |
| 1 | B | 710 | VAL |
| 1 | B | 719 | ARG |
| 1 | B | 725 | CYS |
| 1 | B | 734 | GLU |
| 1 | B | 767 | ILE |
| 1 | C | 81 | THR |
| 1 | C | 82 | THR |
| 1 | C | 96 | GLU |
| 1 | C | 100 | SER |
| 1 | C | 101 | ILE |
| 1 | C | 107 | SER |
| 1 | C | 111 | LEU |

*Continued on next page...*

For Manuscript Review

*Continued from previous page...*

| Mol | Chain | Res | Type |
|---|---|---|---|
| 1 | C | 115 | LEU |
| 1 | C | 125 | SER |
| 1 | C | 128 | ASP |
| 1 | C | 142 | ASP |
| 1 | C | 148 | MET |
| 1 | C | 156 | LEU |
| 1 | C | 158 | CYS |
| 1 | C | 163 | LYS |
| 1 | C | 188 | HIS |
| 1 | C | 191 | GLU |
| 1 | C | 194 | MET |
| 1 | C | 196 | VAL |
| 1 | C | 240 | ASP |
| 1 | C | 245 | MET |
| 1 | C | 248 | THR |
| 1 | C | 257 | VAL |
| 1 | C | 258 | ASP |
| 1 | C | 266 | LYS |
| 1 | C | 270 | LEU |
| 1 | C | 273 | SER |
| 1 | C | 280 | SER |
| 1 | C | 303 | LYS |
| 1 | C | 332 | SER |
| 1 | C | 337 | SER |
| 1 | C | 340 | GLU |
| 1 | C | 342 | CYS |
| 1 | C | 351 | THR |
| 1 | C | 353 | ASP |
| 1 | C | 363 | VAL |
| 1 | C | 378 | LEU |
| 1 | C | 399 | CYS |
| 1 | C | 400 | LEU |
| 1 | C | 414 | ASN |
| 1 | C | 419 | SER |
| 1 | C | 429 | VAL |
| 1 | C | 449 | ARG |
| 1 | C | 459 | ASP |
| 1 | C | 475 | SER |
| 1 | C | 483 | LEU |
| 1 | C | 493 | ASP |
| 1 | C | 520 | SER |
| 1 | C | 540 | SER |

*Continued on next page...*

For Manuscript Review

*Continued from previous page...*

| Mol | Chain | Res | Type |
|---|---|---|---|
| 1 | C | 549 | ASP |
| 1 | C | 554 | LEU |
| 1 | C | 583 | LEU |
| 1 | C | 587 | VAL |
| 1 | C | 589 | SER |
| 1 | C | 591 | PHE |
| 1 | C | 593 | ARG |
| 1 | C | 605 | GLU |
| 1 | C | 607 | LEU |
| 1 | C | 609 | SER |
| 1 | C | 610 | LEU |
| 1 | C | 615 | ASP |
| 1 | C | 618 | SER |
| 1 | C | 635 | LYS |
| 1 | C | 645 | ASP |
| 1 | C | 664 | THR |
| 1 | C | 670 | LEU |
| 1 | C | 674 | SER |
| 1 | C | 679 | LEU |
| 1 | C | 681 | SER |
| 1 | C | 685 | CYS |
| 1 | C | 687 | LEU |
| 1 | C | 688 | SER |
| 1 | C | 695 | GLU |
| 1 | C | 699 | SER |
| 1 | C | 701 | GLU |
| 1 | C | 710 | VAL |
| 1 | C | 719 | ARG |
| 1 | C | 725 | CYS |
| 1 | C | 734 | GLU |
| 1 | C | 767 | ILE |
| 1 | D | 81 | THR |
| 1 | D | 82 | THR |
| 1 | D | 96 | GLU |
| 1 | D | 100 | SER |
| 1 | D | 101 | ILE |
| 1 | D | 107 | SER |
| 1 | D | 111 | LEU |
| 1 | D | 115 | LEU |
| 1 | D | 125 | SER |
| 1 | D | 128 | ASP |
| 1 | D | 142 | ASP |

*Continued on next page...*

For Manuscript Review

*Continued from previous page...*

| Mol | Chain | Res | Type |
|---|---|---|---|
| 1 | D | 148 | MET |
| 1 | D | 156 | LEU |
| 1 | D | 158 | CYS |
| 1 | D | 163 | LYS |
| 1 | D | 188 | HIS |
| 1 | D | 191 | GLU |
| 1 | D | 194 | MET |
| 1 | D | 196 | VAL |
| 1 | D | 240 | ASP |
| 1 | D | 245 | MET |
| 1 | D | 248 | THR |
| 1 | D | 257 | VAL |
| 1 | D | 258 | ASP |
| 1 | D | 266 | LYS |
| 1 | D | 270 | LEU |
| 1 | D | 273 | SER |
| 1 | D | 280 | SER |
| 1 | D | 301 | VAL |
| 1 | D | 303 | LYS |
| 1 | D | 332 | SER |
| 1 | D | 337 | SER |
| 1 | D | 340 | GLU |
| 1 | D | 342 | CYS |
| 1 | D | 351 | THR |
| 1 | D | 353 | ASP |
| 1 | D | 363 | VAL |
| 1 | D | 378 | LEU |
| 1 | D | 399 | CYS |
| 1 | D | 400 | LEU |
| 1 | D | 414 | ASN |
| 1 | D | 419 | SER |
| 1 | D | 429 | VAL |
| 1 | D | 449 | ARG |
| 1 | D | 459 | ASP |
| 1 | D | 475 | SER |
| 1 | D | 483 | LEU |
| 1 | D | 493 | ASP |
| 1 | D | 520 | SER |
| 1 | D | 540 | SER |
| 1 | D | 549 | ASP |
| 1 | D | 554 | LEU |
| 1 | D | 583 | LEU |

*Continued on next page...*

For Manuscript Review

*Continued from previous page...*

| Mol | Chain | Res | Type |
|---|---|---|---|
| 1 | D | 587 | VAL |
| 1 | D | 589 | SER |
| 1 | D | 591 | PHE |
| 1 | D | 593 | ARG |
| 1 | D | 605 | GLU |
| 1 | D | 607 | LEU |
| 1 | D | 609 | SER |
| 1 | D | 610 | LEU |
| 1 | D | 615 | ASP |
| 1 | D | 618 | SER |
| 1 | D | 635 | LYS |
| 1 | D | 645 | ASP |
| 1 | D | 664 | THR |
| 1 | D | 670 | LEU |
| 1 | D | 674 | SER |
| 1 | D | 679 | LEU |
| 1 | D | 681 | SER |
| 1 | D | 685 | CYS |
| 1 | D | 687 | LEU |
| 1 | D | 688 | SER |
| 1 | D | 695 | GLU |
| 1 | D | 699 | SER |
| 1 | D | 701 | GLU |
| 1 | D | 710 | VAL |
| 1 | D | 719 | ARG |
| 1 | D | 725 | CYS |
| 1 | D | 734 | GLU |
| 1 | D | 767 | ILE |
| 1 | A | 81 | THR |
| 1 | A | 82 | THR |
| 1 | A | 96 | GLU |
| 1 | A | 100 | SER |
| 1 | A | 101 | ILE |
| 1 | A | 107 | SER |
| 1 | A | 111 | LEU |
| 1 | A | 115 | LEU |
| 1 | A | 125 | SER |
| 1 | A | 128 | ASP |
| 1 | A | 142 | ASP |
| 1 | A | 148 | MET |
| 1 | A | 156 | LEU |
| 1 | A | 158 | CYS |

*Continued on next page...*

For Manuscript Review

*Continued from previous page...*

| Mol | Chain | Res | Type |
|---|---|---|---|
| 1 | A | 163 | LYS |
| 1 | A | 188 | HIS |
| 1 | A | 191 | GLU |
| 1 | A | 194 | MET |
| 1 | A | 196 | VAL |
| 1 | A | 240 | ASP |
| 1 | A | 245 | MET |
| 1 | A | 248 | THR |
| 1 | A | 257 | VAL |
| 1 | A | 258 | ASP |
| 1 | A | 266 | LYS |
| 1 | A | 270 | LEU |
| 1 | A | 273 | SER |
| 1 | A | 280 | SER |
| 1 | A | 303 | LYS |
| 1 | A | 332 | SER |
| 1 | A | 337 | SER |
| 1 | A | 340 | GLU |
| 1 | A | 342 | CYS |
| 1 | A | 351 | THR |
| 1 | A | 353 | ASP |
| 1 | A | 363 | VAL |
| 1 | A | 378 | LEU |
| 1 | A | 399 | CYS |
| 1 | A | 400 | LEU |
| 1 | A | 414 | ASN |
| 1 | A | 419 | SER |
| 1 | A | 429 | VAL |
| 1 | A | 449 | ARG |
| 1 | A | 459 | ASP |
| 1 | A | 475 | SER |
| 1 | A | 483 | LEU |
| 1 | A | 493 | ASP |
| 1 | A | 520 | SER |
| 1 | A | 540 | SER |
| 1 | A | 549 | ASP |
| 1 | A | 554 | LEU |
| 1 | A | 583 | LEU |
| 1 | A | 587 | VAL |
| 1 | A | 589 | SER |
| 1 | A | 591 | PHE |
| 1 | A | 593 | ARG |

*Continued on next page...*

For Manuscript Review

Continued from previous page...

| Mol | Chain | Res | Type |
|---|---|---|---|
| 1 | A | 605 | GLU |
| 1 | A | 607 | LEU |
| 1 | A | 609 | SER |
| 1 | A | 610 | LEU |
| 1 | A | 615 | ASP |
| 1 | A | 618 | SER |
| 1 | A | 635 | LYS |
| 1 | A | 645 | ASP |
| 1 | A | 664 | THR |
| 1 | A | 670 | LEU |
| 1 | A | 674 | SER |
| 1 | A | 679 | LEU |
| 1 | A | 681 | SER |
| 1 | A | 685 | CYS |
| 1 | A | 687 | LEU |
| 1 | A | 688 | SER |
| 1 | A | 695 | GLU |
| 1 | A | 699 | SER |
| 1 | A | 701 | GLU |
| 1 | A | 710 | VAL |
| 1 | A | 719 | ARG |
| 1 | A | 725 | CYS |
| 1 | A | 734 | GLU |
| 1 | A | 767 | ILE |

Sometimes sidechains can be flipped to improve hydrogen bonding and reduce clashes. All (42) such sidechains are listed below:

| Mol | Chain | Res | Type |
|---|---|---|---|
| 1 | B | 168 | HIS |
| 1 | B | 291 | GLN |
| 1 | B | 334 | HIS |
| 1 | B | 357 | HIS |
| 1 | B | 531 | ASN |
| 1 | B | 551 | GLN |
| 1 | B | 678 | GLN |
| 1 | B | 696 | ASN |
| 1 | B | 709 | GLN |
| 1 | B | 723 | GLN |
| 1 | B | 764 | GLN |
| 1 | C | 291 | GLN |
| 1 | C | 334 | HIS |
| 1 | C | 357 | HIS |

Continued on next page...

For Manuscript Review

*Continued from previous page...*

| Mol | Chain | Res | Type |
|---|---|---|---|
| 1 | C | 531 | ASN |
| 1 | C | 551 | GLN |
| 1 | C | 678 | GLN |
| 1 | C | 696 | ASN |
| 1 | C | 709 | GLN |
| 1 | C | 723 | GLN |
| 1 | C | 764 | GLN |
| 1 | D | 291 | GLN |
| 1 | D | 334 | HIS |
| 1 | D | 357 | HIS |
| 1 | D | 531 | ASN |
| 1 | D | 551 | GLN |
| 1 | D | 678 | GLN |
| 1 | D | 696 | ASN |
| 1 | D | 709 | GLN |
| 1 | D | 723 | GLN |
| 1 | D | 764 | GLN |
| 1 | A | 168 | HIS |
| 1 | A | 291 | GLN |
| 1 | A | 334 | HIS |
| 1 | A | 357 | HIS |
| 1 | A | 531 | ASN |
| 1 | A | 551 | GLN |
| 1 | A | 678 | GLN |
| 1 | A | 696 | ASN |
| 1 | A | 709 | GLN |
| 1 | A | 723 | GLN |
| 1 | A | 764 | GLN |

### 5.3.3 RNA (i)

There are no RNA molecules in this entry.

## 5.4 Non-standard residues in protein, DNA, RNA chains (i)

There are no non-standard protein/DNA/RNA residues in this entry.

## 5.5 Carbohydrates (i)

There are no monosaccharides in this entry.

For Manuscript Review

## 5.6 Ligand geometry (i)

There are no ligands in this entry.

## 5.7 Other polymers (i)

There are no such residues in this entry.

## 5.8 Polymer linkage issues (i)

There are no chain breaks in this entry.

For Manuscript Review

# 6 Map visualisation (i)

This section contains visualisations of the EMDB entry EMD-35202. These allow visual inspection of the internal detail of the map and identification of artifacts.

No raw map or half-maps were deposited for this entry and therefore no images, graphs, etc. pertaining to the raw map can be shown.

## 6.1 Orthogonal projections (i)

### 6.1.1 Primary map

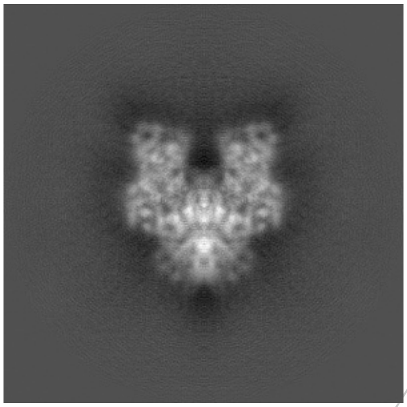
X

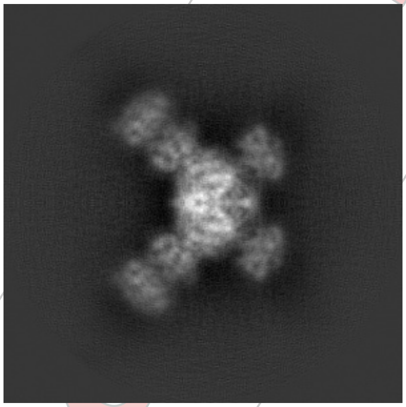
Y

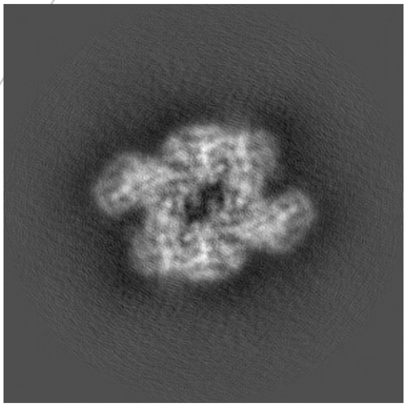
Z

The images above show the map projected in three orthogonal directions.

## 6.2 Central slices (i)

### 6.2.1 Primary map

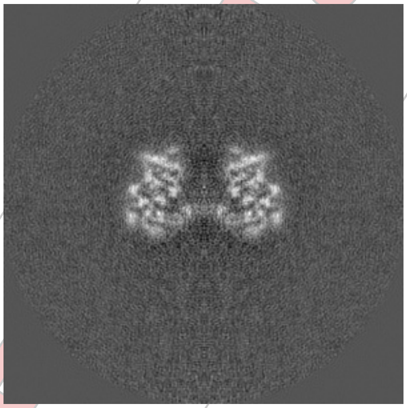
X Index: 140

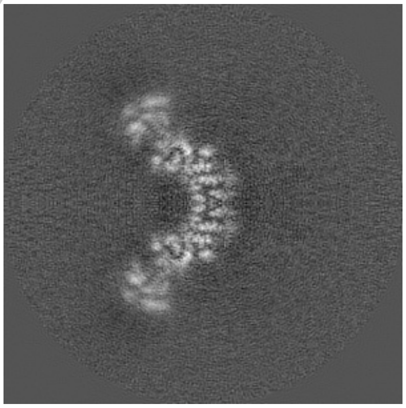
Y Index: 140

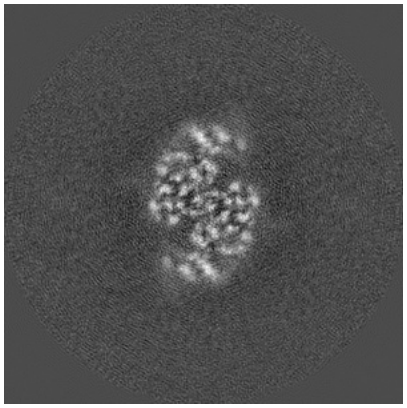
Z Index: 140

For Manuscript Review

The images above show central slices of the map in three orthogonal directions.

## 6.3 Largest variance slices (i)

### 6.3.1 Primary map

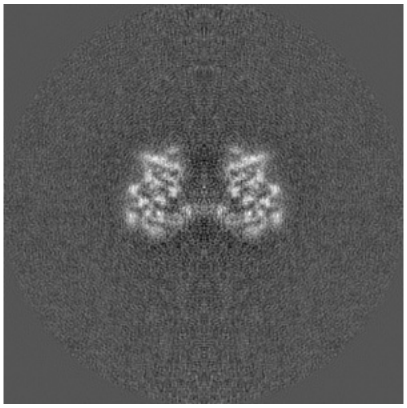
X Index: 140

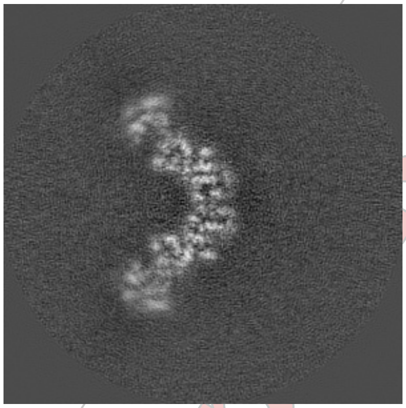
Y Index: 141

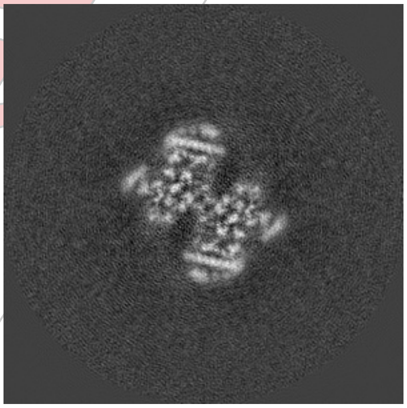
Z Index: 133

The images above show the largest variance slices of the map in three orthogonal directions.

## 6.4 Orthogonal surface views (i)

### 6.4.1 Primary map

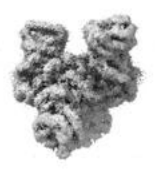
X

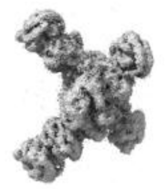
Y

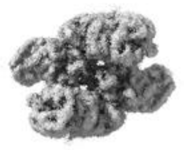
Z

The images above show the 3D surface view of the map at the recommended contour level 0.0137. These images, in conjunction with the slice images, may facilitate assessment of whether an appropriate contour level has been provided.

For Manuscript Review

## 6.5 Mask visualisation (i)

This section was not generated. No masks/segmentation were deposited.

For Manuscript Review

# 7 Map analysis (i)

This section contains the results of statistical analysis of the map.

## 7.1 Map-value distribution (i)

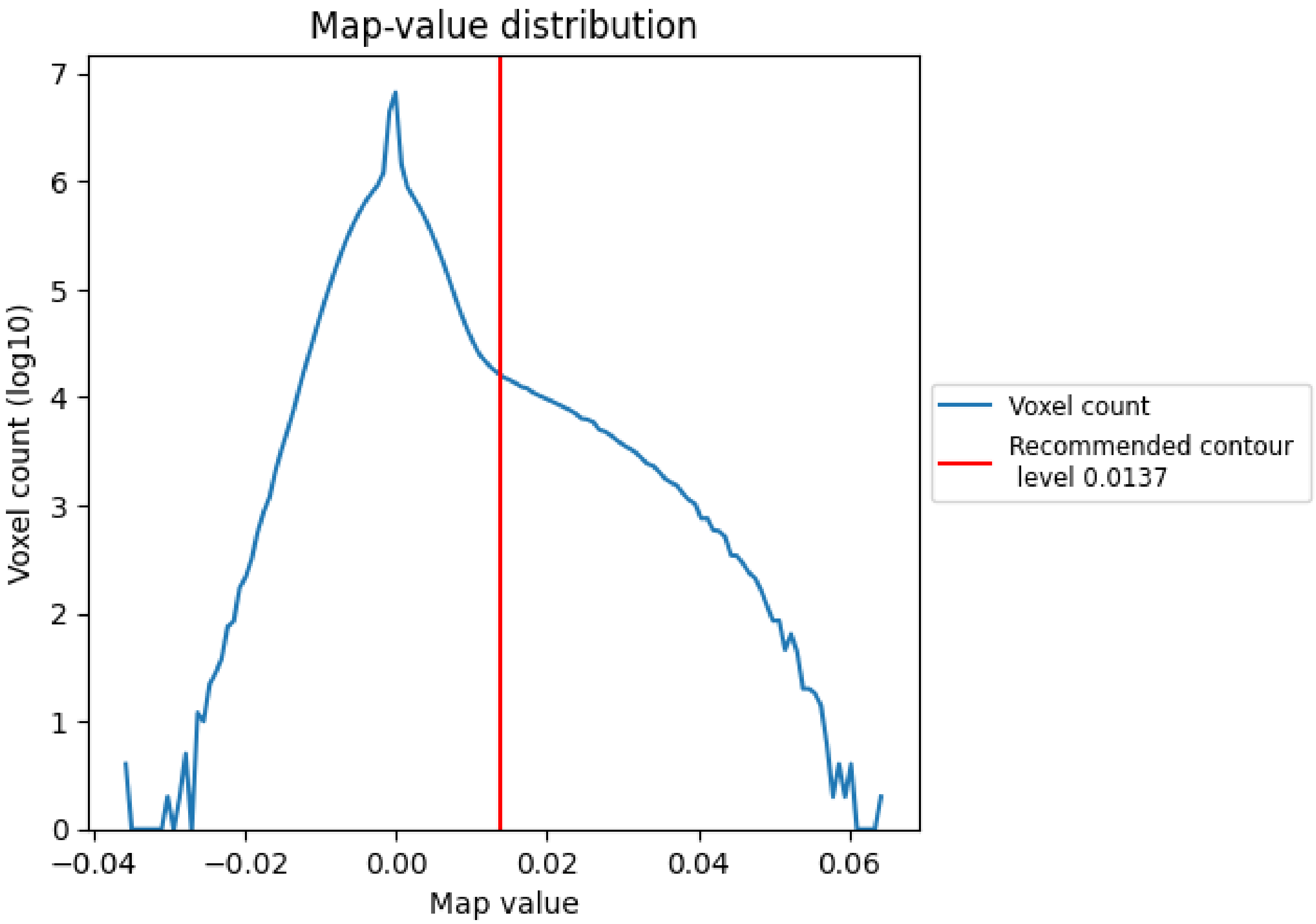


The map-value distribution is plotted in 128 intervals along the x-axis. The y-axis is logarithmic. A spike in this graph at zero usually indicates that the volume has been masked.

For Manuscript Review

## 7.2 Volume estimate (i)

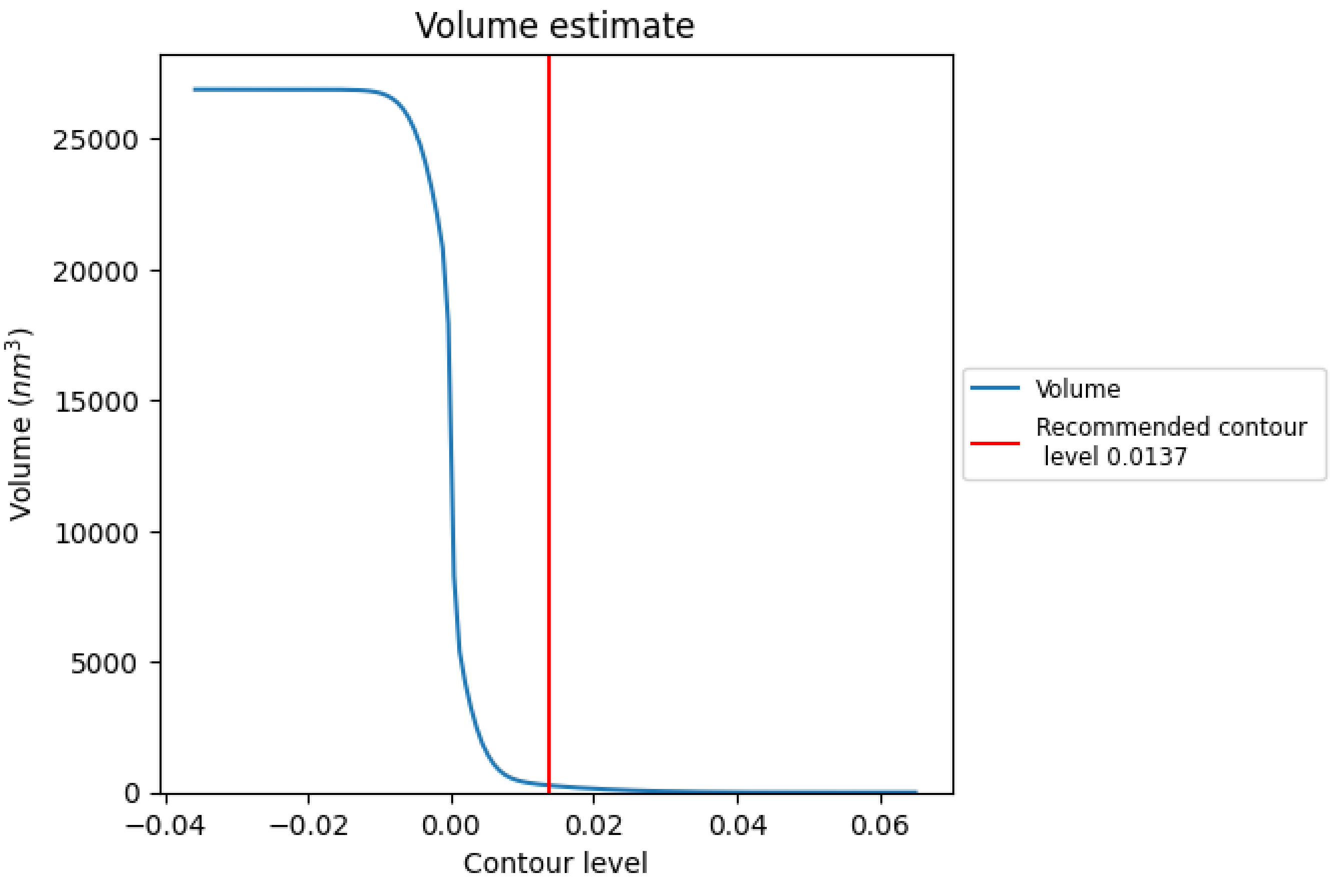


The volume at the recommended contour level is 270 $nm^3$; this corresponds to an approximate mass of 244 kDa.

The volume estimate graph shows how the enclosed volume varies with the contour level. The recommended contour level is shown as a vertical line and the intersection between the line and the curve gives the volume of the enclosed surface at the given level.

For Manuscript Review

## 7.3 Rotationally averaged power spectrum (i)

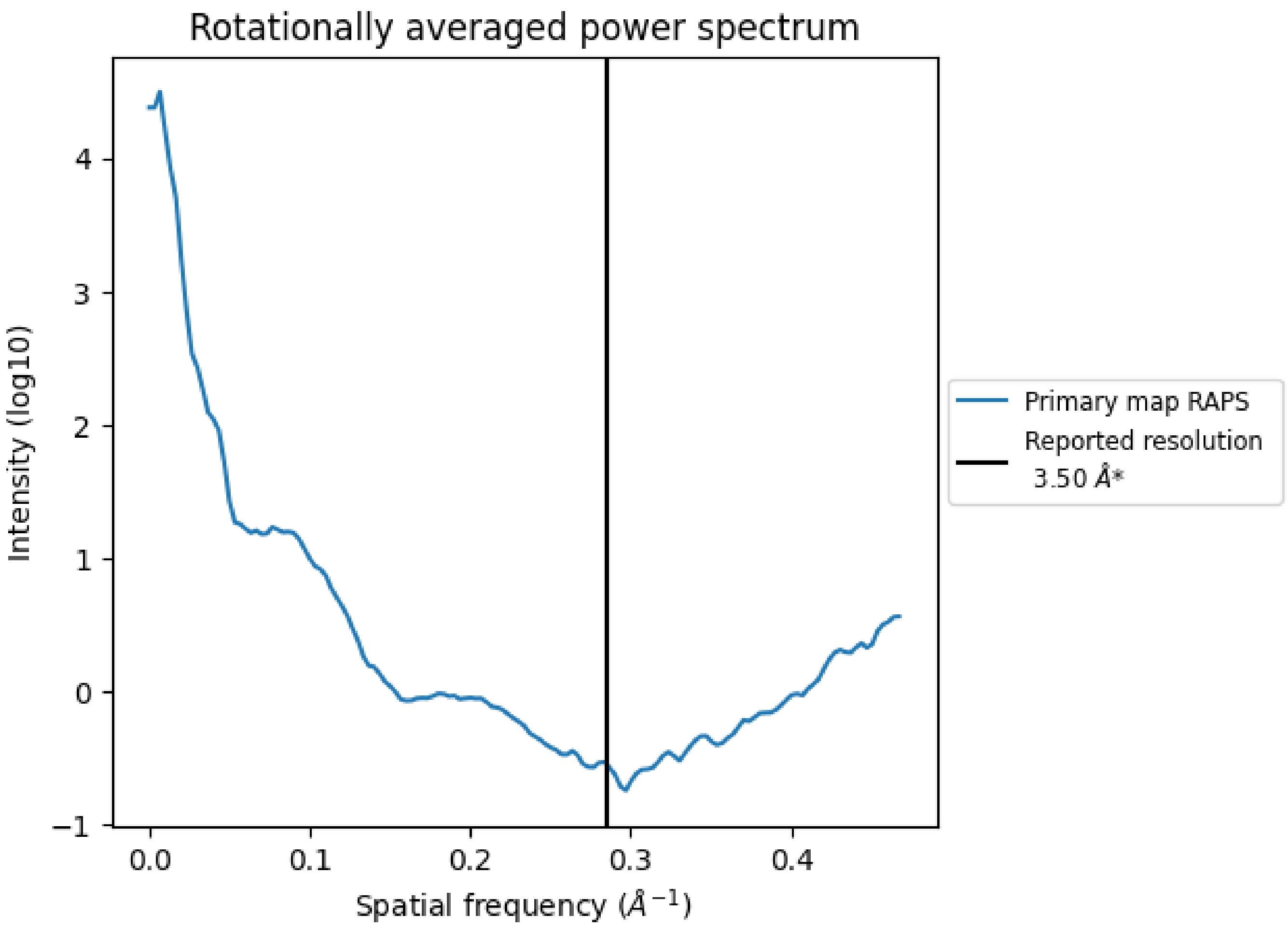


*Reported resolution corresponds to spatial frequency of 0.286 $Å^{-1}$

For Manuscript Review

# 8 Fourier-Shell correlation (i)

This section was not generated. No FSC curve or half-maps provided.

For Manuscript Review

# 9 Map-model fit (i)

This section contains information regarding the fit between EMDB map EMD-35202 and PDB model 8I6P. Per-residue inclusion information can be found in section 3 on page 5.

## 9.1 Map-model overlay (i)

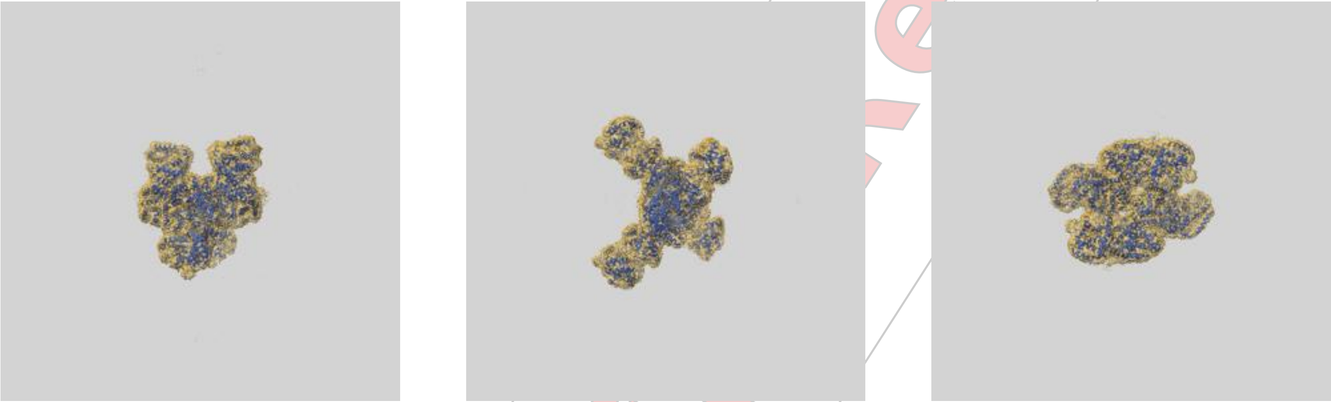

X Y Z

The images above show the 3D surface view of the map at the recommended contour level 0.0137 at 50% transparency in yellow overlaid with a ribbon representation of the model coloured in blue. These images allow for the visual assessment of the quality of fit between the atomic model and the map.

For Manuscript Review

## 9.2 Q-score mapped to coordinate model (i)

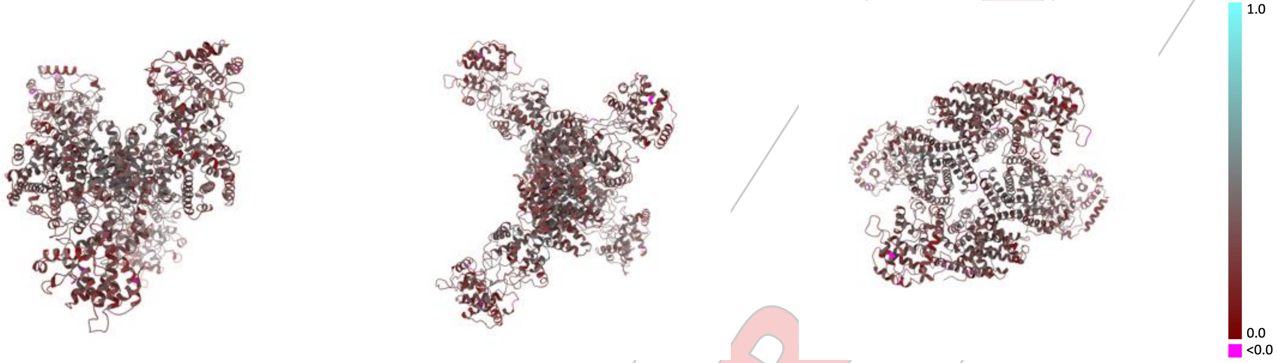


X Y Z

The images above show the model with each residue coloured according its Q-score. This shows their resolvability in the map with higher Q-score values reflecting better resolvability. Please note: Q-score is calculating the resolvability of atoms, and thus high values are only expected at resolutions at which atoms can be resolved. Low Q-score values may therefore be expected for many entries.

## 9.3 Atom inclusion mapped to coordinate model (i)

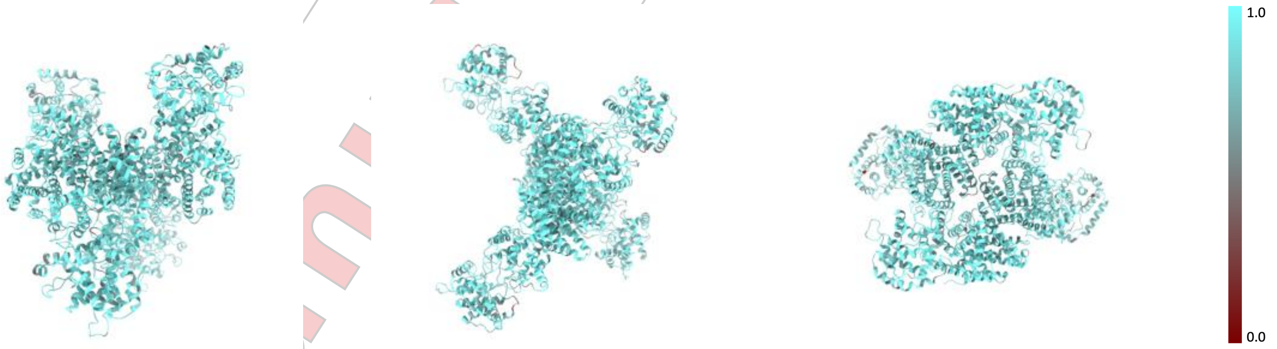


X Y Z

The images above show the model with each residue coloured according to its atom inclusion. This shows to what extent they are inside the map at the recommended contour level (0.0137).

For Manuscript Review

## 9.4 Atom inclusion (i)

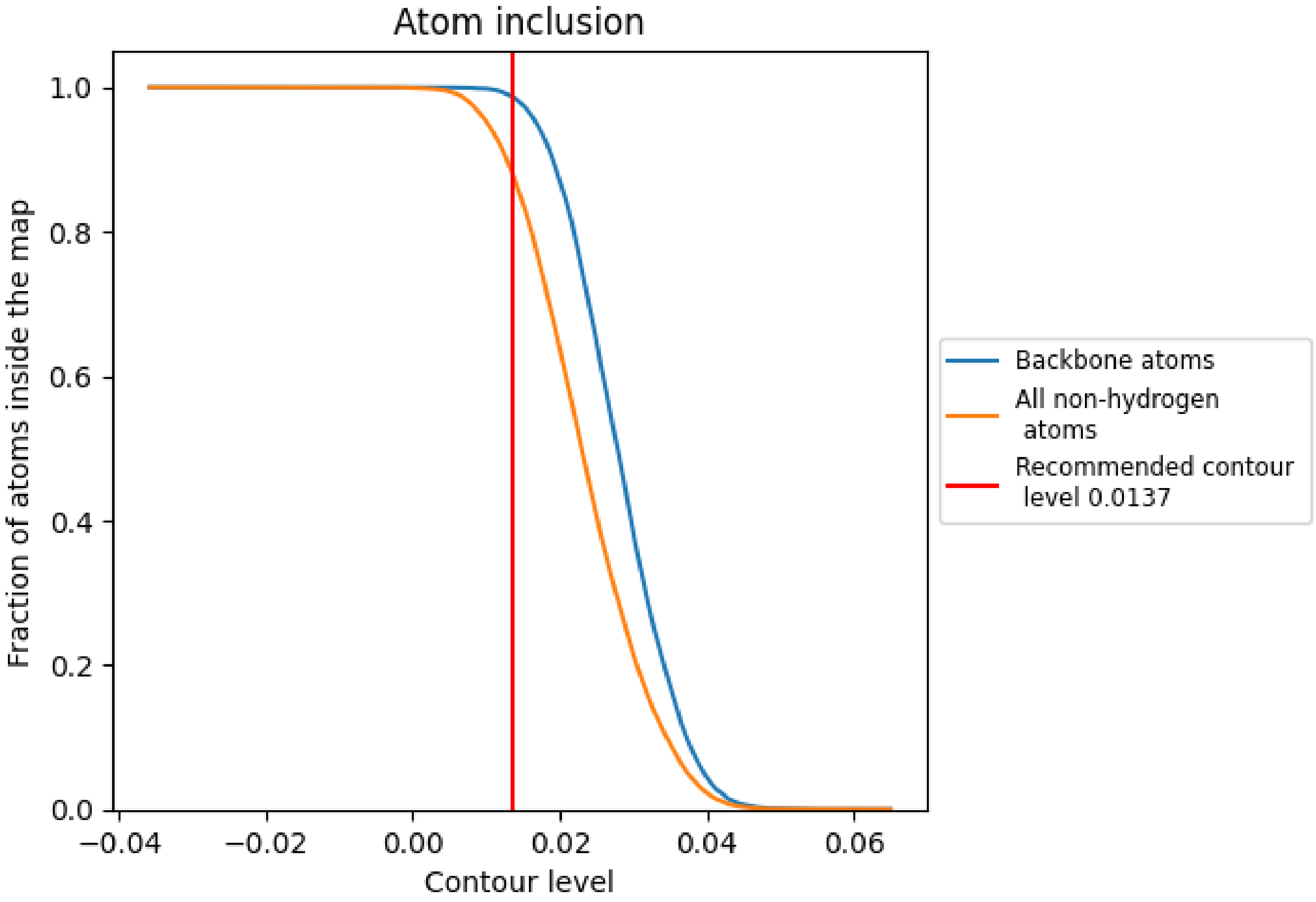


At the recommended contour level, 99% of all backbone atoms, 88% of all non-hydrogen atoms, are inside the map.

For Manuscript Review

## 9.5 Map-model fit summary (i)

The table lists the average atom inclusion at the recommended contour level (0.0137) and Q-score for the entire model and for each chain.

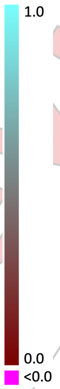


| Chain | Atom inclusion | Q-score |
|---|---|---|
| All | 0.8773 | 0.3190 |
| A | 0.8843 | 0.3140 |
| B | 0.8696 | 0.3210 |
| C | 0.8696 | 0.3270 |
| D | 0.8860 | 0.3130 |

For Manuscript Review
