# Supplementary material for: Structural and functional investigations of syn-copalyl diphosphate synthase from Oryza sativa
Source: Commun Chem. 2023 Nov 6;6:240. doi: 10.1038/s42004-023-01042-w (PMC10628199; doi:10.1038/s42004-023-01042-w)
Supplement: Supplementary file 8 — Supplementary Data 5 [file 42004_2023_1042_MOESM8_ESM.pdf]

Full wwPDB EM Validation Report (i)

Feb 9, 2023 – 08:33 PM JST

PDB ID	:	8I6T
EMDB ID	:	EMD-35206
Title	:	The cryo-EM structure of OsCyc1 hexamer state
Deposited on	:	2023-01-29
Resolution	:	3.70 Å(reported)

This wwPDB validation report is for manuscript review

For Manuscript Review

This is a Full wwPDB EM Validation Report.

This report is produced by the wwPDB biocuration pipeline after annotation of the structure.

We welcome your comments at *validation@mail.wwpdb.org*
A user guide is available at
https://www.wwpdb.org/validation/2017/EMValidationReportHelp
with specific help available everywhere you see the (i) symbol.

The types of validation reports are described at
http://www.wwpdb.org/validation/2017/FAQs#types.

The following versions of software and data (see references (i)) were used in the production of this report:

EMDB validation analysis	:	0.0.1.dev43
MolProbity	:	4.02b-467
Percentile statistics	:	20191225.v01 (using entries in the PDB archive December 25th 2019)
MapQ	:	1.9.9
Ideal geometry (proteins)	:	Engh & Huber (2001)
Ideal geometry (DNA, RNA)	:	Parkinson et al. (1996)
Validation Pipeline (wwPDB-VP)	:	2.32.1

1 Overall quality at a glance (i)

The following experimental techniques were used to determine the structure:
ELECTRON MICROSCOPY

The reported resolution of this entry is 3.70 Å.

Percentile scores (ranging between 0-100) for global validation metrics of the entry are shown in the following graphic. The table shows the number of entries on which the scores are based.

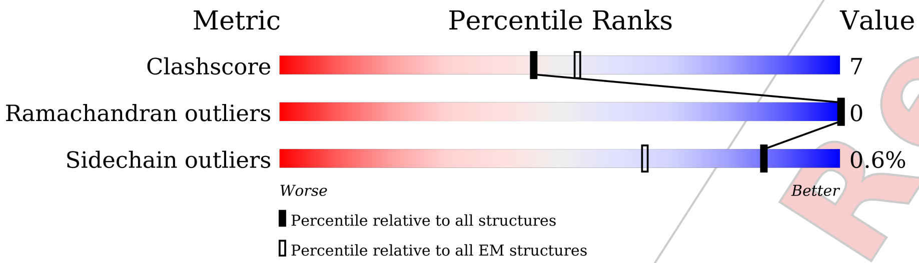

Metric	Whole archive (#Entries)	EM structures (#Entries)
Clashscore	158937	4297
Ramachandran outliers	154571	4023
Sidechain outliers	154315	3826

The table below summarises the geometric issues observed across the polymeric chains and their fit to the map. The red, orange, yellow and green segments of the bar indicate the fraction of residues that contain outliers for >=3, 2, 1 and 0 types of geometric quality criteria respectively. A grey segment represents the fraction of residues that are not modelled. The numeric value for each fraction is indicated below the corresponding segment, with a dot representing fractions <=5% The upper red bar (where present) indicates the fraction of residues that have poor fit to the EM map (all-atom inclusion < 40%). The numeric value is given above the bar.

Mol	Chain	Length	Quality of chain
1	A	775	9% / 74% 14% • 11%
1	B	775	5% / 69% 19% 11%
1	C	775	10% / 75% 14% 11%
1	D	775	• / 74% 14% 11%
1	E	775	14% / 71% 18% 11%
1	F	775	9% / 73% 15% 12%

For Manuscript Review

2 Entry composition (i)

There is only 1 type of molecule in this entry. The entry contains 32983 atoms, of which 0 are hydrogens and 0 are deuteriums.

In the tables below, the AltConf column contains the number of residues with at least one atom in alternate conformation and the Trace column contains the number of residues modelled with at most 2 atoms.

- Molecule 1 is a protein called Syn-copalyl diphosphate synthase, chloroplastic.

Mol	Chain	Residues	Atoms					AltConf	Trace
			Total	C	N	O	S		
1	A	688	5504	3492	944	1033	35	0	0
1	B	688	5504	3492	944	1033	35	0	0
1	C	688	5504	3492	944	1033	35	0	0
1	D	688	5504	3492	944	1033	35	0	0
1	E	688	5504	3492	944	1033	35	0	0
1	F	682	5463	3466	938	1024	35	0	0

There are 48 discrepancies between the modelled and reference sequences:

Chain	Residue	Modelled	Actual	Comment	Reference
A	768	GLU	-	expression tag	UNP Q0JF02
A	769	PHE	-	expression tag	UNP Q0JF02
A	770	HIS	-	expression tag	UNP Q0JF02
A	771	HIS	-	expression tag	UNP Q0JF02
A	772	HIS	-	expression tag	UNP Q0JF02
A	773	HIS	-	expression tag	UNP Q0JF02
A	774	HIS	-	expression tag	UNP Q0JF02
A	775	HIS	-	expression tag	UNP Q0JF02
B	768	GLU	-	expression tag	UNP Q0JF02
B	769	PHE	-	expression tag	UNP Q0JF02
B	770	HIS	-	expression tag	UNP Q0JF02
B	771	HIS	-	expression tag	UNP Q0JF02
B	772	HIS	-	expression tag	UNP Q0JF02
B	773	HIS	-	expression tag	UNP Q0JF02
B	774	HIS	-	expression tag	UNP Q0JF02
B	775	HIS	-	expression tag	UNP Q0JF02
C	768	GLU	-	expression tag	UNP Q0JF02
C	769	PHE	-	expression tag	UNP Q0JF02

Continued on next page...

For Manuscript Review

Continued from previous page...

Chain	Residue	Modelled	Actual	Comment	Reference
C	770	HIS	-	expression tag	UNP Q0JF02
C	771	HIS	-	expression tag	UNP Q0JF02
C	772	HIS	-	expression tag	UNP Q0JF02
C	773	HIS	-	expression tag	UNP Q0JF02
C	774	HIS	-	expression tag	UNP Q0JF02
C	775	HIS	-	expression tag	UNP Q0JF02
D	768	GLU	-	expression tag	UNP Q0JF02
D	769	PHE	-	expression tag	UNP Q0JF02
D	770	HIS	-	expression tag	UNP Q0JF02
D	771	HIS	-	expression tag	UNP Q0JF02
D	772	HIS	-	expression tag	UNP Q0JF02
D	773	HIS	-	expression tag	UNP Q0JF02
D	774	HIS	-	expression tag	UNP Q0JF02
D	775	HIS	-	expression tag	UNP Q0JF02
E	768	GLU	-	expression tag	UNP Q0JF02
E	769	PHE	-	expression tag	UNP Q0JF02
E	770	HIS	-	expression tag	UNP Q0JF02
E	771	HIS	-	expression tag	UNP Q0JF02
E	772	HIS	-	expression tag	UNP Q0JF02
E	773	HIS	-	expression tag	UNP Q0JF02
E	774	HIS	-	expression tag	UNP Q0JF02
E	775	HIS	-	expression tag	UNP Q0JF02
F	768	GLU	-	expression tag	UNP Q0JF02
F	769	PHE	-	expression tag	UNP Q0JF02
F	770	HIS	-	expression tag	UNP Q0JF02
F	771	HIS	-	expression tag	UNP Q0JF02
F	772	HIS	-	expression tag	UNP Q0JF02
F	773	HIS	-	expression tag	UNP Q0JF02
F	774	HIS	-	expression tag	UNP Q0JF02
F	775	HIS	-	expression tag	UNP Q0JF02

For Manuscript Review

3 Residue-property plots

These plots are drawn for all protein, RNA, DNA and oligosaccharide chains in the entry. The first graphic for a chain summarises the proportions of the various outlier classes displayed in the second graphic. The second graphic shows the sequence view annotated by issues in geometry and atom inclusion in map density. Residues are color-coded according to the number of geometric quality criteria for which they contain at least one outlier: green = 0, yellow = 1, orange = 2 and red = 3 or more. A red diamond above a residue indicates a poor fit to the EM map for this residue (all-atom inclusion < 40%). Stretches of 2 or more consecutive residues without any outlier are shown as a green connector. Residues present in the sample, but not in the model, are shown in grey.

- Molecule 1: Syn-copalyl diphosphate synthase, chloroplastic

Chain A:

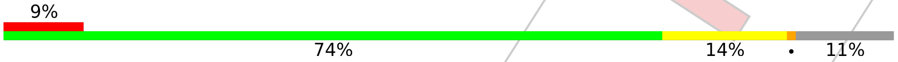

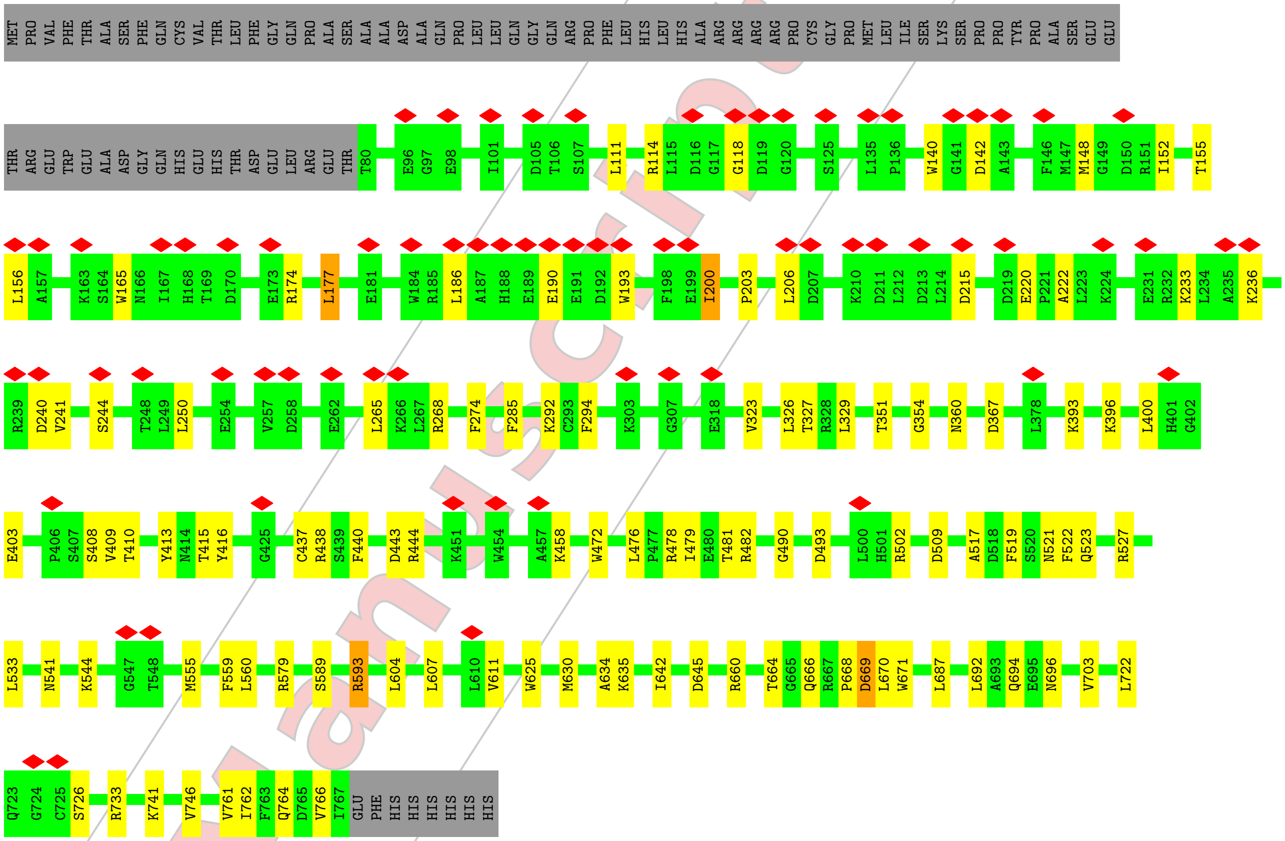

- Molecule 1: Syn-copalyl diphosphate synthase, chloroplastic

Chain B:

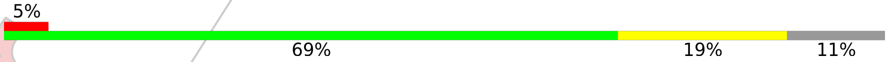

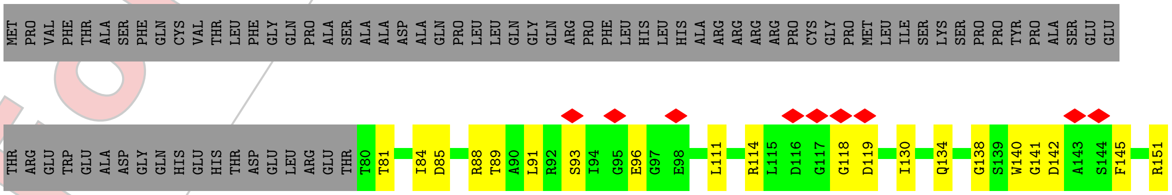

For Manuscript Review

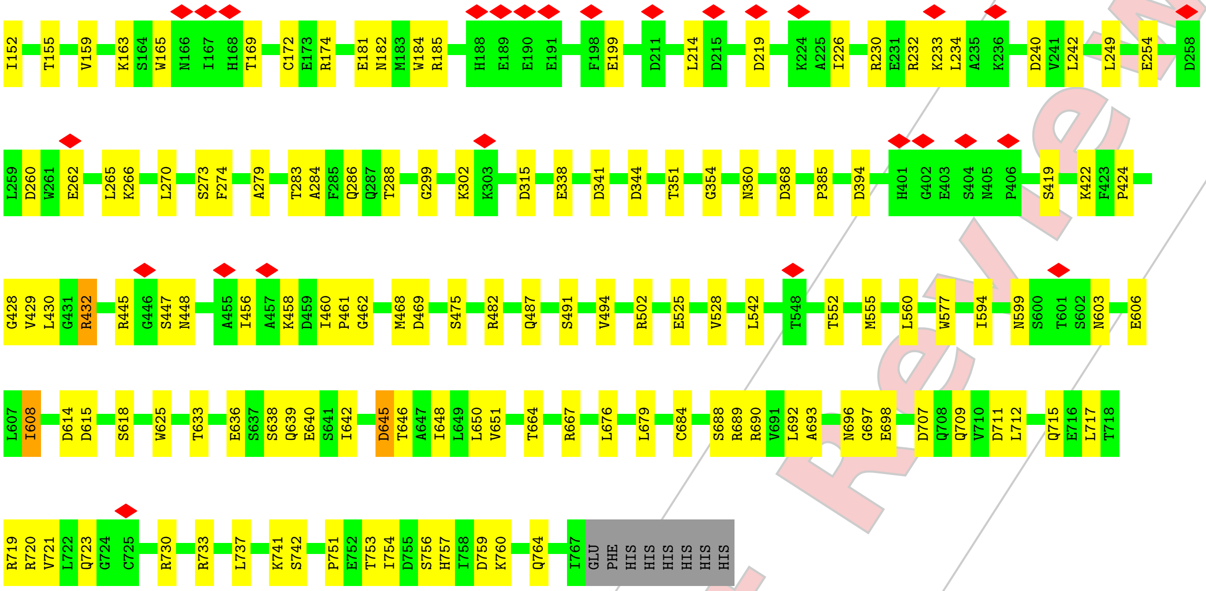

• Molecule 1: Syn-copalyl diphosphate synthase, chloroplastic

Chain C:

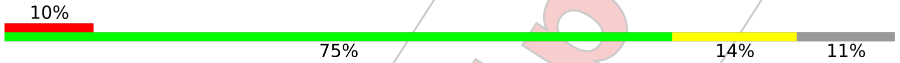

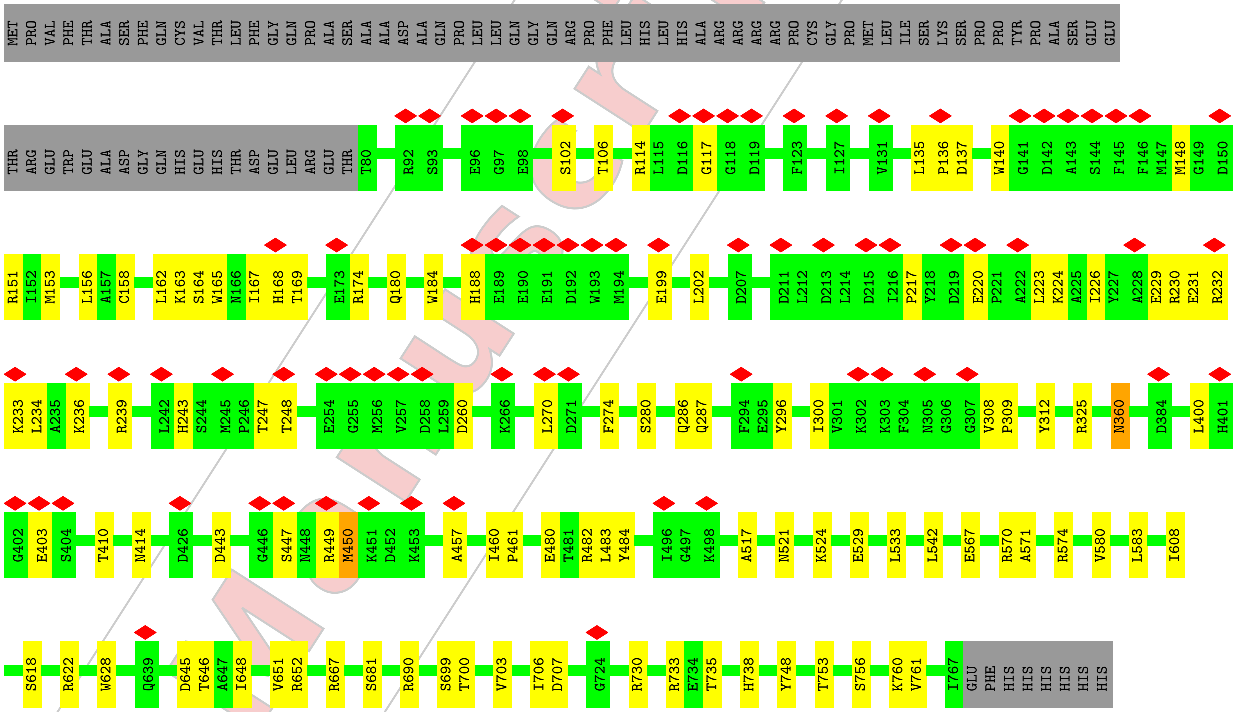

• Molecule 1: Syn-copalyl diphosphate synthase, chloroplastic

Chain D:

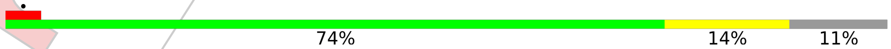

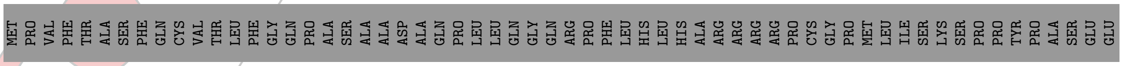

For Manuscript Review

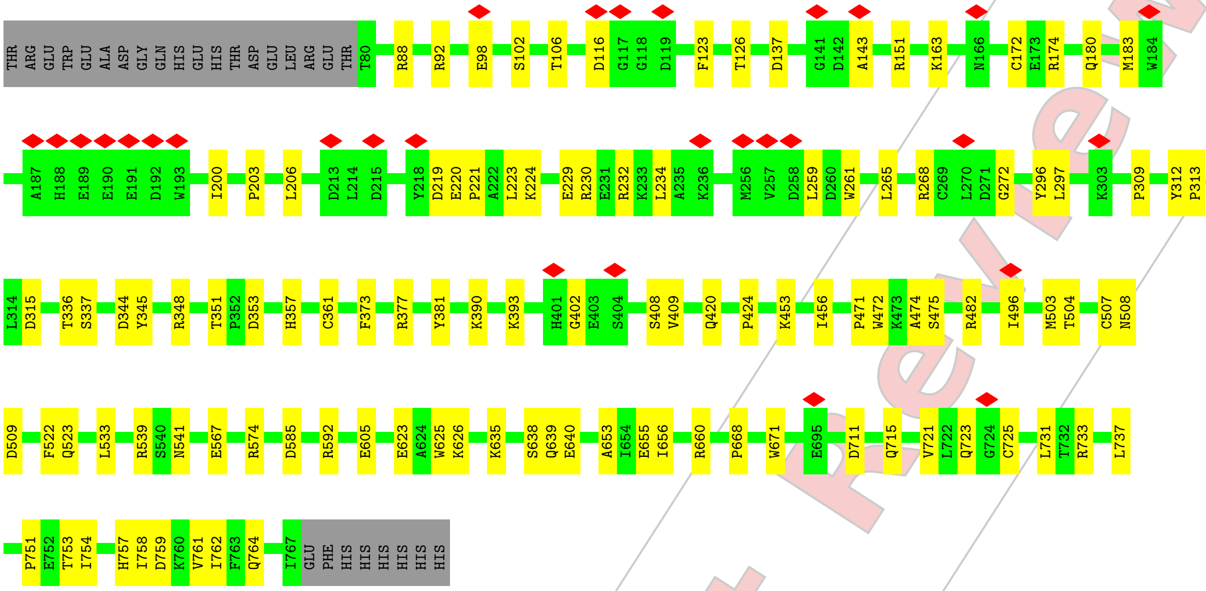

• Molecule 1: Syn-copalyl diphosphate synthase, chloroplastic

Chain E:

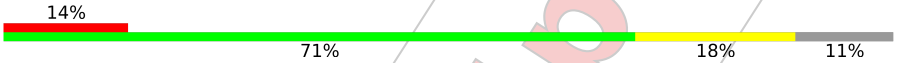

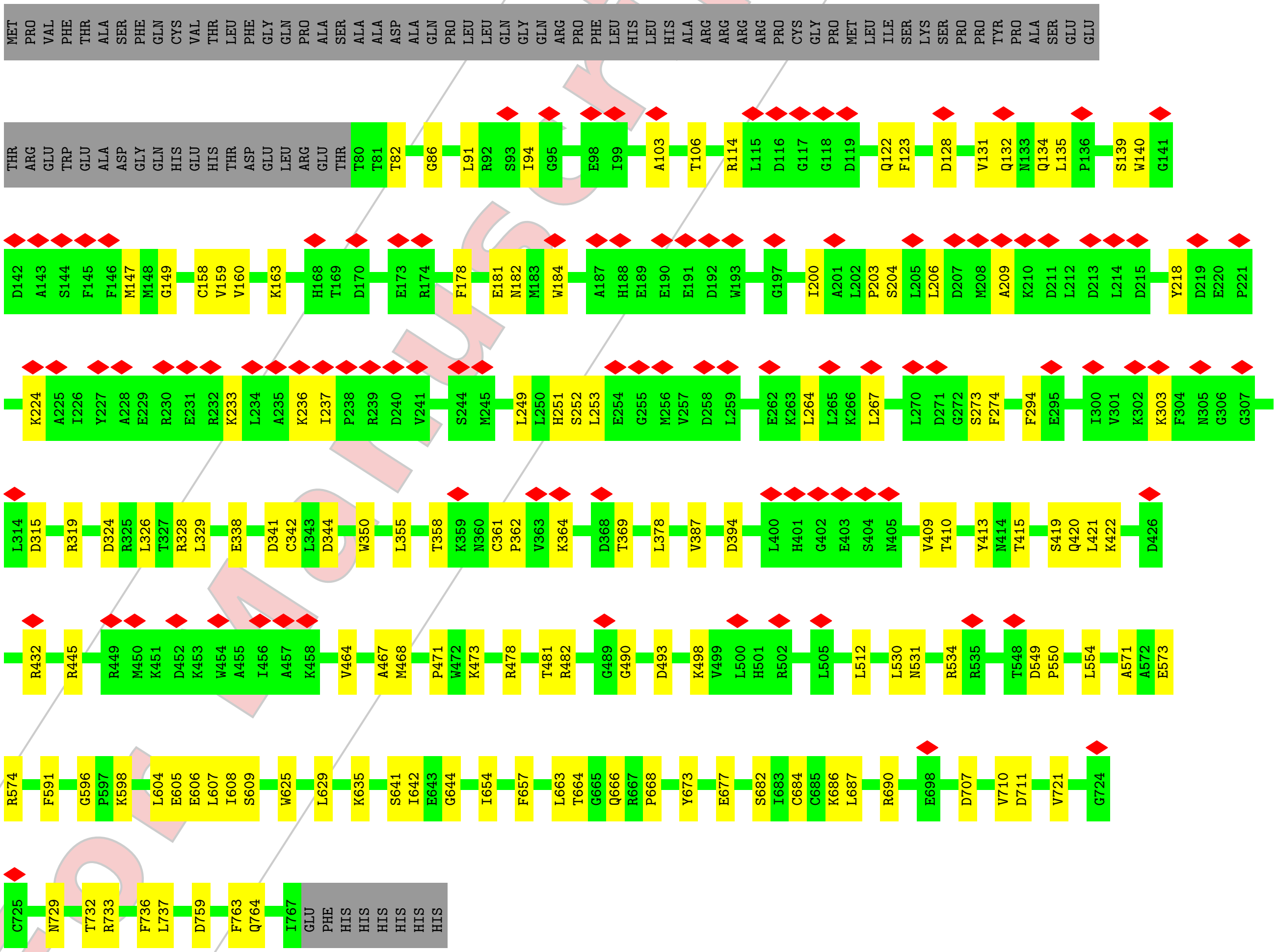

• Molecule 1: Syn-copalyl diphosphate synthase, chloroplastic

For Manuscript Review

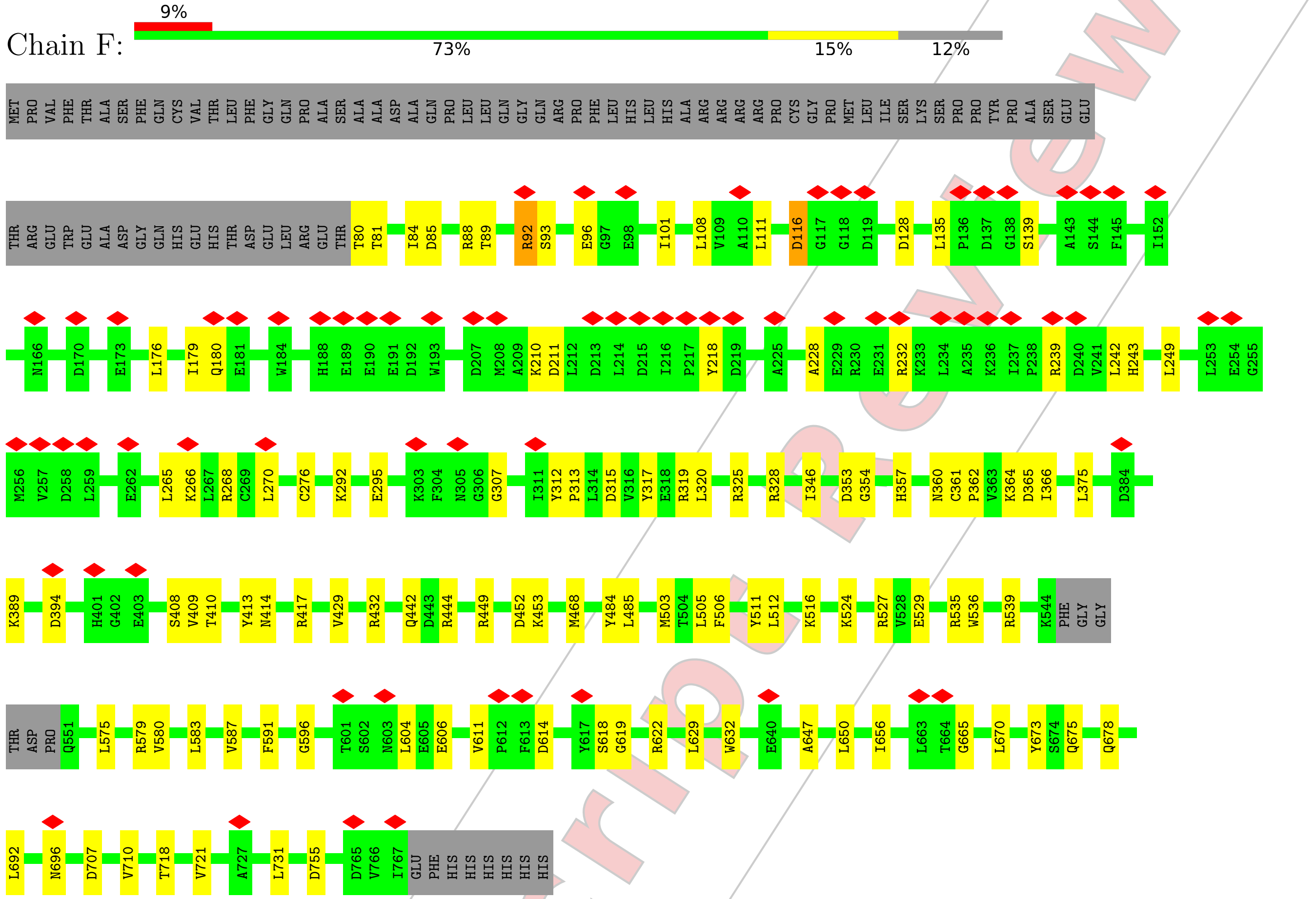

For Manuscript Review

4 Experimental information (i)

Property	Value	Source
EM reconstruction method	SINGLE PARTICLE	Depositor
Imposed symmetry	POINT, Not provided	
Number of particles used	97919	Depositor
Resolution determination method	FSC 0.143 CUT-OFF	Depositor
CTF correction method	NONE	Depositor
Microscope	FEI TITAN KRIOS	Depositor
Voltage (kV)	300	Depositor
Electron dose $(e^-/\text{Å}^2)$	60	Depositor
Minimum defocus (nm)	1500	Depositor
Maximum defocus (nm)	2300	Depositor
Magnification	Not provided	
Image detector	GATAN K3 (6k x 4k)	Depositor
Maximum map value	0.072	Depositor
Minimum map value	-0.041	Depositor
Average map value	0.000	Depositor
Map value standard deviation	0.005	Depositor
Recommended contour level	0.0176	Depositor
Map size (Å)	299.6, 299.6, 299.6	wwPDB
Map dimensions	280, 280, 280	wwPDB
Map angles (°)	90.0, 90.0, 90.0	wwPDB
Pixel spacing (Å)	1.07, 1.07, 1.07	Depositor

For Manuscript Review

5 Model quality ⓘ

5.1 Standard geometry ⓘ

The Z score for a bond length (or angle) is the number of standard deviations the observed value is removed from the expected value. A bond length (or angle) with $|Z| > 5$ is considered an outlier worth inspection. RMSZ is the root-mean-square of all Z scores of the bond lengths (or angles).

Mol	Chain	Bond lengths		Bond angles	
		RMSZ	#|Z| >5	RMSZ	#|Z| >5
1	A	0.31	0/5627	0.55	3/7615 (0.0%)
1	B	0.31	0/5627	0.54	2/7615 (0.0%)
1	C	0.31	0/5627	0.54	1/7615 (0.0%)
1	D	0.31	0/5627	0.53	0/7615
1	E	0.31	0/5627	0.54	3/7615 (0.0%)
1	F	0.30	0/5583	0.55	1/7553 (0.0%)
All	All	0.31	0/33718	0.54	10/45628 (0.0%)

There are no bond length outliers.

All (10) bond angle outliers are listed below:

Mol	Chain	Res	Type	Atoms	Z	Observed(°)	Ideal(°)
1	F	394	ASP	CB-CG-OD1	6.10	123.79	118.30
1	E	253	LEU	CA-CB-CG	5.85	128.75	115.30
1	A	722	LEU	CA-CB-CG	5.76	128.55	115.30
1	C	542	LEU	CA-CB-CG	5.60	128.19	115.30
1	B	394	ASP	CB-CG-OD1	5.26	123.03	118.30
1	E	394	ASP	CB-CG-OD1	5.23	123.01	118.30
1	B	608	ILE	CG1-CB-CG2	-5.20	99.96	111.40
1	A	156	LEU	CA-CB-CG	5.13	127.10	115.30
1	E	378	LEU	CA-CB-CG	5.10	127.04	115.30
1	A	177	LEU	CA-CB-CG	5.01	126.83	115.30

There are no chirality outliers.

There are no planarity outliers.

5.2 Too-close contacts ⓘ

In the following table, the Non-H and H(model) columns list the number of non-hydrogen atoms and hydrogen atoms in the chain respectively. The H(added) column lists the number of hydrogen atoms added and optimized by MolProbity. The Clashes column lists the number of clashes within the asymmetric unit, whereas Symm-Clashes lists symmetry-related clashes.

For Manuscript Review

Mol	Chain	Non-H	H(model)	H(added)	Clashes	Symm-Clashes
1	A	5504	0	5413	68	0
1	B	5504	0	5413	93	0
1	C	5504	0	5413	65	0
1	D	5504	0	5413	69	0
1	E	5504	0	5413	79	0
1	F	5463	0	5379	70	0
All	All	32983	0	32444	436	0

The all-atom clashscore is defined as the number of clashes found per 1000 atoms (including hydrogen atoms). The all-atom clashscore for this structure is 7.

All (436) close contacts within the same asymmetric unit are listed below, sorted by their clash magnitude.

Atom-1	Atom-2	Interatomic distance (Å)	Clash overlap (Å)
1:A:400:LEU:HD23	1:A:403:GLU:HB2	1.75	0.68
1:D:163:LYS:NZ	1:D:172:CYS:SG	2.68	0.66
1:C:628:TRP:HE1	1:C:646:THR:HG22	1.61	0.66
1:D:503:MET:HB2	1:D:507:CYS:HB2	1.79	0.65
1:C:220:GLU:HB3	1:C:223:LEU:HB2	1.79	0.65
1:F:228:ALA:O	1:F:232:ARG:N	2.29	0.64
1:A:114:ARG:HD3	1:A:118:GLY:HA3	1.78	0.64
1:C:325:ARG:NH1	1:C:484:TYR:OH	2.30	0.64
1:A:472:TRP:O	1:A:741:LYS:NZ	2.32	0.63
1:C:223:LEU:HG	1:C:226:ILE:HD11	1.80	0.63
1:F:579:ARG:HD2	1:F:611:VAL:HG23	1.82	0.62
1:E:140:TRP:NE1	1:E:158:CYS:SG	2.73	0.61
1:E:355:LEU:H	1:E:369:THR:HG22	1.65	0.61
1:E:654:ILE:HA	1:E:657:PHE:HB2	1.82	0.61
1:F:529:GLU:HG2	1:F:575:LEU:HD21	1.82	0.61
1:C:199:GLU:HG2	1:C:226:ILE:HG22	1.81	0.61
1:C:648:ILE:HD11	1:C:681:SER:HB3	1.83	0.61
1:C:135:LEU:HD12	1:C:137:ASP:H	1.64	0.61
1:C:608:ILE:HG21	1:C:622:ARG:HG3	1.83	0.60
1:E:410:THR:HA	1:E:413:TYR:HB3	1.82	0.60
1:E:134:GLN:NE2	1:E:135:LEU:O	2.33	0.60
1:B:299:GLY:HA2	1:B:302:LYS:HD2	1.82	0.59
1:C:243:HIS:HE1	1:C:260:ASP:H	1.48	0.59
1:A:410:THR:HA	1:A:413:TYR:HB3	1.84	0.59
1:F:116:ASP:OD1	1:F:116:ASP:N	2.35	0.59
1:B:85:ASP:HA	1:B:88:ARG:HB3	1.85	0.59
1:E:682:SER:O	1:E:686:LYS:NZ	2.34	0.59
1:B:645:ASP:OD1	1:B:645:ASP:N	2.32	0.59

Continued on next page...

For Manuscript Review

Continued from previous page...

Atom-1	Atom-2	Interatomic distance (Å)	Clash overlap (Å)
1:D:137:ASP:O	1:D:174:ARG:NH1	2.35	0.59
1:F:292:LYS:HA	1:F:295:GLU:HB2	1.85	0.59
1:E:160:VAL:HA	1:E:163:LYS:HG2	1.85	0.58
1:E:324:ASP:O	1:E:328:ARG:NH2	2.37	0.58
1:E:571:ALA:HA	1:E:574:ARG:HH21	1.68	0.58
1:D:102:SER:H	1:D:309:PRO:HA	1.68	0.58
1:D:259:LEU:HD12	1:D:261:TRP:HE1	1.69	0.58
1:A:490:GLY:HA3	1:A:509:ASP:HB3	1.86	0.58
1:B:429:VAL:HA	1:B:432:ARG:HH21	1.69	0.58
1:F:629:LEU:HA	1:F:632:TRP:HD1	1.68	0.58
1:B:603:ASN:ND2	1:B:606:GLU:OE2	2.37	0.57
1:B:633:THR:HA	1:B:636:GLU:HB2	1.86	0.57
1:D:203:PRO:HA	1:D:206:LEU:HB2	1.86	0.57
1:B:152:ILE:HA	1:B:155:THR:HG22	1.86	0.57
1:C:286:GLN:NE2	1:C:287:GLN:OE1	2.38	0.57
1:C:517:ALA:O	1:C:521:ASN:ND2	2.37	0.57
1:F:96:GLU:HB3	1:F:313:PRO:HB3	1.85	0.57
1:A:493:ASP:O	1:A:502:ARG:NH1	2.38	0.57
1:C:410:THR:O	1:C:414:ASN:ND2	2.37	0.57
1:A:579:ARG:HD2	1:A:611:VAL:HG23	1.87	0.57
1:A:692:LEU:O	1:A:696:ASN:ND2	2.38	0.57
1:B:491:SER:O	1:B:502:ARG:NH1	2.36	0.57
1:D:351:THR:HG22	1:D:353:ASP:H	1.69	0.57
1:D:721:VAL:HG21	1:D:737:LEU:HD11	1.86	0.56
1:E:482:ARG:NH2	1:E:764:GLN:O	2.37	0.56
1:F:442:GLN:NE2	1:F:468:MET:SD	2.78	0.56
1:B:469:ASP:OD1	1:B:469:ASP:N	2.38	0.56
1:A:660:ARG:O	1:B:667:ARG:NH2	2.39	0.56
1:B:182:ASN:OD1	1:B:185:ARG:NH2	2.36	0.56
1:D:408:SER:HB2	1:D:453:LYS:H	1.71	0.56
1:F:270:LEU:HB2	1:F:360:ASN:HD22	1.71	0.56
1:D:393:LYS:HE2	1:D:402:GLY:H	1.71	0.56
1:A:285:PHE:HZ	1:A:294:PHE:HB2	1.70	0.55
1:D:344:ASP:OD1	1:D:344:ASP:N	2.39	0.55
1:E:135:LEU:HD22	1:E:139:SER:HB2	1.88	0.55
1:C:102:SER:H	1:C:309:PRO:HA	1.70	0.55
1:B:140:TRP:HE1	1:B:159:VAL:HG13	1.71	0.55
1:B:638:SER:OG	1:B:639:GLN:N	2.40	0.55
1:B:642:ILE:O	1:B:646:THR:OG1	2.24	0.55
1:B:759:ASP:N	1:B:759:ASP:OD1	2.39	0.55
1:C:140:TRP:HE1	1:C:158:CYS:HG	1.53	0.55

Continued on next page...

For Manuscript Review

Continued from previous page...

Atom-1	Atom-2	Interatomic distance (Å)	Clash overlap (Å)
1:D:221:PRO:HA	1:D:224:LYS:HG2	1.88	0.55
1:D:635:LYS:HD2	1:D:640:GLU:HB2	1.88	0.55
1:A:527:ARG:NH2	1:A:762:ILE:O	2.36	0.55
1:E:147:MET:HG3	1:E:149:GLY:H	1.72	0.55
1:E:677:GLU:HB3	1:E:736:PHE:HE2	1.72	0.55
1:A:694:GLN:HE22	1:A:703:VAL:HG22	1.71	0.55
1:E:315:ASP:OD1	1:E:315:ASP:N	2.39	0.55
1:F:389:LYS:HG2	1:F:429:VAL:HG21	1.89	0.55
1:A:240:ASP:N	1:A:240:ASP:OD1	2.39	0.55
1:B:560:LEU:HG	1:B:742:SER:HB2	1.89	0.55
1:F:270:LEU:HD13	1:F:360:ASN:HB2	1.89	0.55
1:A:233:LYS:HA	1:A:236:LYS:HG2	1.89	0.54
1:A:443:ASP:N	1:A:443:ASP:OD1	2.37	0.54
1:D:123:PHE:HB2	1:D:126:THR:HG23	1.90	0.54
1:D:711:ASP:O	1:D:715:GLN:NE2	2.41	0.54
1:E:128:ASP:O	1:E:132:GLN:NE2	2.40	0.54
1:E:82:THR:O	1:E:86:GLY:N	2.41	0.54
1:F:755:ASP:N	1:F:755:ASP:OD1	2.40	0.54
1:D:420:GLN:HB3	1:D:472:TRP:HB2	1.88	0.54
1:F:429:VAL:HG23	1:F:432:ARG:HH21	1.73	0.54
1:C:230:ARG:NH1	1:C:231:GLU:OE2	2.38	0.54
1:E:666:GLN:HE21	1:E:668:PRO:HD3	1.73	0.54
1:A:482:ARG:NH2	1:A:764:GLN:O	2.34	0.54
1:A:541:ASN:O	1:A:544:LYS:NZ	2.38	0.54
1:C:148:MET:HA	1:C:151:ARG:HB3	1.90	0.54
1:C:231:GLU:HA	1:C:234:LEU:HG	1.90	0.54
1:B:89:THR:O	1:B:93:SER:OG	2.25	0.53
1:B:460:ILE:HG13	1:B:461:PRO:HD3	1.88	0.53
1:E:530:LEU:HB3	1:E:534:ARG:HH21	1.72	0.53
1:E:707:ASP:HA	1:E:710:VAL:HG22	1.90	0.53
1:A:726:SER:OG	1:A:733:ARG:NH1	2.40	0.53
1:D:265:LEU:O	1:D:268:ARG:NH1	2.41	0.53
1:F:315:ASP:OD1	1:F:315:ASP:N	2.40	0.53
1:C:648:ILE:HA	1:C:651:VAL:HG12	1.90	0.53
1:D:567:GLU:O	1:D:574:ARG:NH2	2.41	0.53
1:A:220:GLU:HG2	1:A:222:ALA:H	1.72	0.53
1:E:606:GLU:O	1:E:609:SER:OG	2.26	0.53
1:D:751:PRO:HA	1:D:754:ILE:HG12	1.91	0.53
1:E:209:ALA:O	1:E:218:TYR:OH	2.24	0.53
1:C:248:THR:HG21	1:C:400:LEU:HD21	1.90	0.53
1:F:675:GLN:NE2	1:F:678:GLN:OE1	2.42	0.53

Continued on next page...

For Manuscript Review

Continued from previous page...

Atom-1	Atom-2	Interatomic distance (Å)	Clash overlap (Å)
1:A:393:LYS:HB2	1:A:396:LYS:HB2	1.91	0.53
1:F:452:ASP:OD1	1:F:452:ASP:N	2.37	0.53
1:F:101:ILE:HD13	1:F:307:GLY:HA3	1.91	0.52
1:B:163:LYS:HE3	1:B:214:LEU:HD11	1.91	0.52
1:B:525:GLU:HA	1:B:528:VAL:HG22	1.89	0.52
1:D:353:ASP:HA	1:D:390:LYS:HE3	1.91	0.52
1:B:482:ARG:NH2	1:B:764:GLN:O	2.43	0.52
1:C:521:ASN:HA	1:C:524:LYS:HB3	1.90	0.52
1:B:756:SER:O	1:B:760:LYS:NZ	2.36	0.52
1:D:471:PRO:O	1:D:475:SER:OG	2.27	0.52
1:F:409:VAL:HG12	1:F:452:ASP:HB3	1.92	0.52
1:E:549:ASP:N	1:E:549:ASP:OD1	2.43	0.52
1:B:711:ASP:OD1	1:B:711:ASP:N	2.42	0.52
1:D:408:SER:OG	1:D:409:VAL:N	2.43	0.52
1:F:135:LEU:HB3	1:F:139:SER:H	1.72	0.52
1:D:723:GLN:OE1	1:D:725:CYS:N	2.43	0.51
1:E:729:ASN:HB3	1:E:732:THR:HG22	1.92	0.51
1:A:415:THR:HG1	1:A:437:CYS:HG	1.58	0.51
1:B:385:PRO:HB2	1:B:430:LEU:HD11	1.91	0.51
1:C:480:GLU:O	1:C:484:TYR:N	2.43	0.51
1:D:315:ASP:OD1	1:D:315:ASP:N	2.38	0.51
1:F:85:ASP:OD1	1:F:88:ARG:NH2	2.44	0.51
1:C:567:GLU:OE2	1:C:570:ARG:NH1	2.43	0.51
1:D:336:THR:OG1	1:D:337:SER:N	2.44	0.51
1:F:81:THR:HA	1:F:84:ILE:HG12	1.93	0.51
1:F:670:LEU:HA	1:F:673:TYR:HB3	1.91	0.51
1:E:445:ARG:NH1	1:E:468:MET:SD	2.83	0.51
1:B:447:SER:OG	1:B:448:ASN:N	2.44	0.51
1:B:696:ASN:HB3	1:B:698:GLU:HG2	1.93	0.51
1:C:457:ALA:HB3	1:C:460:ILE:HD11	1.93	0.51
1:E:114:ARG:HD3	1:E:122:GLN:HG3	1.92	0.50
1:E:200:ILE:O	1:E:204:SER:OG	2.27	0.50
1:E:178:PHE:O	1:E:182:ASN:ND2	2.44	0.50
1:F:80:THR:OG1	1:F:81:THR:N	2.44	0.50
1:E:664:THR:HG21	1:F:665:GLY:HA3	1.93	0.50
1:C:167:ILE:HG22	1:C:168:HIS:HD2	1.77	0.50
1:F:535:ARG:O	1:F:539:ARG:N	2.43	0.50
1:A:517:ALA:O	1:A:521:ASN:ND2	2.44	0.50
1:D:424:PRO:HB3	1:D:715:GLN:HB3	1.94	0.50
1:E:131:VAL:HG23	1:E:132:GLN:HG3	1.92	0.50
1:F:647:ALA:HA	1:F:650:LEU:HD12	1.93	0.50

Continued on next page...

For Manuscript Review

Continued from previous page...

Atom-1	Atom-2	Interatomic distance (Å)	Clash overlap (Å)
1:B:419:SER:O	1:B:422:LYS:NZ	2.44	0.50
1:B:552:THR:HA	1:B:555:MET:HB2	1.92	0.50
1:A:440:PHE:O	1:A:444:ARG:NE	2.44	0.50
1:B:270:LEU:HB2	1:B:360:ASN:HB2	1.93	0.50
1:B:615:ASP:OD1	1:B:615:ASP:N	2.45	0.50
1:E:471:PRO:HG2	1:E:473:LYS:HG2	1.93	0.50
1:E:273:SER:OG	1:E:274:PHE:N	2.45	0.50
1:A:416:TYR:OH	1:A:438:ARG:NH2	2.43	0.50
1:A:265:LEU:O	1:A:268:ARG:NH1	2.42	0.49
1:B:240:ASP:OD1	1:B:240:ASP:N	2.42	0.49
1:A:148:MET:HE3	1:A:186:LEU:HG	1.94	0.49
1:F:239:ARG:O	1:F:243:HIS:ND1	2.45	0.49
1:E:604:LEU:HA	1:E:607:LEU:HD13	1.95	0.49
1:A:669:ASP:OD1	1:A:669:ASP:N	2.45	0.49
1:C:153:MET:HG3	1:C:202:LEU:HD12	1.95	0.49
1:D:668:PRO:HA	1:D:671:TRP:HB2	1.95	0.49
1:F:409:VAL:HG22	1:F:413:TYR:HB2	1.95	0.49
1:B:199:GLU:HA	1:B:226:ILE:HD12	1.95	0.49
1:D:523:GLN:NE2	1:D:761:VAL:O	2.45	0.49
1:A:200:ILE:HG12	1:A:233:LYS:HD3	1.95	0.49
1:F:211:ASP:OD1	1:F:211:ASP:N	2.46	0.49
1:F:606:GLU:OE1	1:F:622:ARG:NH2	2.44	0.49
1:F:618:SER:OG	1:F:619:GLY:N	2.43	0.49
1:B:273:SER:OG	1:B:274:PHE:N	2.46	0.48
1:B:462:GLY:O	1:B:487:GLN:NE2	2.44	0.48
1:B:693:ALA:O	1:B:697:GLY:N	2.40	0.48
1:D:623:GLU:HA	1:D:626:LYS:HG2	1.94	0.48
1:E:478:ARG:HA	1:E:481:THR:HG22	1.93	0.48
1:A:203:PRO:HA	1:A:206:LEU:HD23	1.95	0.48
1:A:241:VAL:O	1:A:244:SER:OG	2.30	0.48
1:F:128:ASP:OD1	1:F:128:ASP:N	2.47	0.48
1:A:761:VAL:HG23	1:A:762:ILE:HG13	1.94	0.48
1:B:114:ARG:NH1	1:B:119:ASP:OD1	2.46	0.48
1:D:102:SER:O	1:D:106:THR:OG1	2.31	0.48
1:E:233:LYS:HA	1:E:236:LYS:HG2	1.94	0.48
1:E:721:VAL:HG11	1:E:737:LEU:HD22	1.95	0.48
1:B:648:ILE:HA	1:B:651:VAL:HG22	1.95	0.48
1:D:230:ARG:O	1:D:234:LEU:N	2.41	0.48
1:F:408:SER:HB2	1:F:453:LYS:H	1.78	0.48
1:B:140:TRP:HD1	1:B:155:THR:HA	1.78	0.48
1:B:577:TRP:HE1	1:B:650:LEU:HD13	1.78	0.48

Continued on next page...

For Manuscript Review

Continued from previous page...

Atom-1	Atom-2	Interatomic distance (Å)	Clash overlap (Å)
1:B:594:ILE:HG13	1:B:599:ASN:HB2	1.96	0.48
1:E:158:CYS:SG	1:E:159:VAL:N	2.87	0.48
1:E:203:PRO:HA	1:E:206:LEU:HB2	1.95	0.48
1:E:550:PRO:O	1:E:554:LEU:N	2.46	0.48
1:B:456:ILE:HD11	1:B:494:VAL:HB	1.96	0.48
1:E:419:SER:O	1:E:422:LYS:NZ	2.40	0.48
1:A:630:MET:SD	1:B:719:ARG:NH2	2.83	0.48
1:F:89:THR:O	1:F:93:SER:OG	2.28	0.48
1:B:242:LEU:HD12	1:B:249:LEU:HD13	1.96	0.48
1:F:320:LEU:HD12	1:F:375:LEU:HD12	1.96	0.48
1:F:365:ASP:OD1	1:F:366:ILE:N	2.46	0.48
1:A:174:ARG:HH11	1:A:177:LEU:HD21	1.79	0.47
1:D:357:HIS:NE2	1:D:361:CYS:SG	2.80	0.47
1:A:351:THR:OG1	1:A:354:GLY:O	2.32	0.47
1:B:81:THR:HA	1:B:84:ILE:HD12	1.96	0.47
1:A:215:ASP:OD1	1:A:215:ASP:N	2.47	0.47
1:A:408:SER:OG	1:A:409:VAL:N	2.47	0.47
1:A:589:SER:O	1:A:593:ARG:NH1	2.47	0.47
1:E:608:ILE:HD12	1:E:625:TRP:HB2	1.96	0.47
1:B:351:THR:OG1	1:B:354:GLY:O	2.31	0.47
1:D:200:ILE:HG23	1:D:230:ARG:HE	1.80	0.47
1:F:409:VAL:O	1:F:413:TYR:N	2.47	0.47
1:C:618:SER:O	1:C:618:SER:OG	2.33	0.47
1:C:234:LEU:O	1:C:239:ARG:NH2	2.47	0.47
1:D:456:ILE:HB	1:D:496:ILE:HD11	1.97	0.47
1:D:482:ARG:NH2	1:D:764:GLN:O	2.40	0.47
1:E:759:ASP:HA	1:E:763:PHE:HB2	1.97	0.47
1:E:635:LYS:NZ	1:E:641:SER:O	2.41	0.47
1:B:141:GLY:HA3	1:B:151:ARG:HG3	1.97	0.47
1:C:652:ARG:HD2	1:D:671:TRP:CD1	2.49	0.47
1:A:140:TRP:HD1	1:A:155:THR:HG22	1.81	0.46
1:A:250:LEU:HD22	1:A:274:PHE:HD2	1.80	0.46
1:C:735:THR:HA	1:C:738:HIS:HB2	1.97	0.46
1:C:760:LYS:HA	1:C:760:LYS:HD3	1.81	0.46
1:D:219:ASP:OD1	1:D:219:ASP:N	2.45	0.46
1:E:759:ASP:N	1:E:759:ASP:OD1	2.46	0.46
1:B:219:ASP:N	1:B:219:ASP:OD1	2.44	0.46
1:E:251:HIS:HE1	1:E:498:LYS:HD3	1.81	0.46
1:A:148:MET:HG2	1:A:152:ILE:HG23	1.98	0.46
1:B:169:THR:HA	1:B:172:CYS:HB3	1.97	0.46
1:C:645:ASP:N	1:C:645:ASP:OD1	2.46	0.46

Continued on next page...

For Manuscript Review

Continued from previous page...

Atom-1	Atom-2	Interatomic distance (Å)	Clash overlap (Å)
1:F:536:TRP:HA	1:F:539:ARG:HB2	1.96	0.46
1:B:96:GLU:HG3	1:B:315:ASP:HB3	1.97	0.46
1:C:730:ARG:HA	1:C:733:ARG:HE	1.80	0.46
1:B:753:THR:O	1:B:757:HIS:ND1	2.45	0.46
1:C:274:PHE:O	1:C:280:SER:OG	2.26	0.46
1:D:229:GLU:HA	1:D:232:ARG:HE	1.81	0.46
1:D:758:ILE:HG23	1:D:762:ILE:HD12	1.97	0.46
1:B:475:SER:O	1:B:741:LYS:NZ	2.40	0.46
1:B:707:ASP:OD1	1:B:707:ASP:N	2.46	0.46
1:A:476:LEU:HB2	1:A:479:ILE:HG12	1.98	0.46
1:D:88:ARG:O	1:D:92:ARG:N	2.49	0.46
1:D:585:ASP:N	1:D:585:ASP:OD1	2.48	0.46
1:B:91:LEU:HG	1:B:338:GLU:HB3	1.99	0.45
1:A:111:LEU:HA	1:A:165:TRP:HE1	1.81	0.45
1:A:645:ASP:OD1	1:A:645:ASP:N	2.39	0.45
1:B:721:VAL:HG11	1:B:737:LEU:HD13	1.96	0.45
1:C:443:ASP:OD1	1:C:443:ASP:N	2.49	0.45
1:E:237:ILE:HD13	1:E:249:LEU:HD13	1.96	0.45
1:F:276:CYS:HB3	1:F:312:TYR:H	1.81	0.45
1:B:283:THR:O	1:B:286:GLN:NE2	2.50	0.45
1:C:753:THR:O	1:C:756:SER:OG	2.32	0.45
1:B:265:LEU:HD12	1:B:266:LYS:HG3	1.99	0.45
1:B:445:ARG:NH1	1:B:468:MET:SD	2.89	0.45
1:C:529:GLU:O	1:C:533:LEU:N	2.49	0.45
1:F:319:ARG:HB3	1:F:346:ILE:HD11	1.99	0.45
1:A:360:ASN:OD1	1:A:360:ASN:N	2.49	0.45
1:D:753:THR:O	1:D:757:HIS:ND1	2.47	0.45
1:B:341:ASP:HA	1:B:344:ASP:HB2	1.99	0.45
1:F:210:LYS:NZ	1:F:218:TYR:OH	2.44	0.45
1:C:480:GLU:HA	1:C:483:LEU:HB2	1.99	0.45
1:E:409:VAL:O	1:E:413:TYR:N	2.48	0.45
1:E:415:THR:O	1:E:419:SER:N	2.49	0.45
1:E:420:GLN:HG3	1:E:421:LEU:HD12	1.97	0.45
1:F:242:LEU:HD21	1:F:249:LEU:HD11	1.99	0.45
1:B:119:ASP:OD1	1:B:119:ASP:N	2.45	0.45
1:A:634:ALA:HA	1:B:424:PRO:HB2	1.99	0.45
1:D:98:GLU:HB3	1:D:504:THR:HB	1.99	0.45
1:E:338:GLU:HA	1:E:341:ASP:HB2	1.99	0.45
1:E:200:ILE:HD11	1:E:252:SER:HB3	1.99	0.44
1:F:325:ARG:NH1	1:F:484:TYR:OH	2.50	0.44
1:B:608:ILE:HD11	1:B:625:TRP:CD1	2.53	0.44

Continued on next page...

For Manuscript Review

Continued from previous page...

Atom-1	Atom-2	Interatomic distance (Å)	Clash overlap (Å)
1:E:684:CYS:HA	1:E:687:LEU:HG	2.00	0.44
1:F:176:LEU:HG	1:F:180:GLN:HE22	1.82	0.44
1:F:265:LEU:HD12	1:F:268:ARG:HH12	1.82	0.44
1:A:478:ARG:HA	1:A:481:THR:HG22	1.99	0.44
1:A:604:LEU:HA	1:A:607:LEU:HB2	1.99	0.44
1:A:635:LYS:HE3	1:A:642:ILE:HD13	1.98	0.44
1:E:319:ARG:HD3	1:E:342:CYS:HB2	2.00	0.44
1:E:326:LEU:HA	1:E:329:LEU:HB2	1.99	0.44
1:F:580:VAL:HA	1:F:583:LEU:HB3	2.00	0.44
1:A:523:GLN:HE22	1:A:766:VAL:H	1.65	0.44
1:F:328:ARG:HH22	1:F:417:ARG:HH12	1.64	0.44
1:D:180:GLN:HA	1:D:183:MET:HG3	2.00	0.44
1:A:142:ASP:OD1	1:A:502:ARG:NH2	2.46	0.44
1:A:555:MET:O	1:A:559:PHE:N	2.45	0.44
1:B:614:ASP:O	1:B:618:SER:OG	2.28	0.44
1:B:689:ARG:HA	1:B:692:LEU:HG	2.00	0.44
1:F:176:LEU:HA	1:F:179:ILE:HD12	2.00	0.44
1:C:580:VAL:HA	1:C:583:LEU:HB2	1.98	0.44
1:B:260:ASP:OD1	1:B:260:ASP:N	2.50	0.44
1:C:162:LEU:HB3	1:C:163:LYS:HZ2	1.83	0.44
1:E:605:GLU:OE1	1:E:605:GLU:N	2.51	0.44
1:E:721:VAL:HG23	1:E:733:ARG:HB3	1.98	0.44
1:B:368:ASP:OD1	1:B:368:ASP:N	2.51	0.44
1:C:690:ARG:HB2	1:C:706:ILE:HG21	1.99	0.44
1:C:707:ASP:OD1	1:C:748:TYR:OH	2.36	0.44
1:D:759:ASP:O	1:D:764:GLN:NE2	2.51	0.44
1:E:344:ASP:N	1:E:344:ASP:OD1	2.48	0.44
1:F:317:TYR:N	1:F:357:HIS:O	2.51	0.44
1:A:458:LYS:HB3	1:A:458:LYS:HE2	1.80	0.43
1:A:604:LEU:O	1:A:625:TRP:NE1	2.48	0.43
1:B:145:PHE:O	1:B:151:ARG:NH2	2.51	0.43
1:C:232:ARG:O	1:C:236:LYS:NZ	2.34	0.43
1:E:490:GLY:N	1:E:493:ASP:OD2	2.50	0.43
1:F:591:PHE:O	1:F:596:GLY:N	2.46	0.43
1:C:482:ARG:HB2	1:C:761:VAL:HG23	1.98	0.43
1:E:264:LEU:HA	1:E:267:LEU:HB2	2.00	0.43
1:B:428:GLY:O	1:B:432:ARG:NE	2.50	0.43
1:E:673:TYR:HE1	1:F:670:LEU:HD13	1.82	0.43
1:F:410:THR:OG1	1:F:414:ASN:ND2	2.51	0.43
1:B:134:GLN:HE21	1:B:138:GLY:HA2	1.83	0.43
1:B:679:LEU:HD13	1:B:717:LEU:HA	2.00	0.43

Continued on next page...

For Manuscript Review

Continued from previous page...

Atom-1	Atom-2	Interatomic distance (Å)	Clash overlap (Å)
1:E:512:LEU:HD23	1:E:512:LEU:HA	1.90	0.43
1:F:108:LEU:HA	1:F:111:LEU:HD12	2.00	0.43
1:B:542:LEU:HD23	1:B:542:LEU:HA	1.87	0.43
1:E:103:ALA:HA	1:E:106:THR:HG22	2.01	0.43
1:E:361:CYS:O	1:E:364:LYS:NZ	2.36	0.43
1:A:666:GLN:HB3	1:A:668:PRO:HD2	2.01	0.43
1:D:508:ASN:OD1	1:D:509:ASP:N	2.51	0.43
1:A:152:ILE:HG21	1:A:186:LEU:HD11	2.00	0.43
1:B:751:PRO:HA	1:B:754:ILE:HG22	1.99	0.43
1:D:143:ALA:HA	1:D:151:ARG:HH21	1.83	0.43
1:E:361:CYS:HA	1:E:362:PRO:HD3	1.88	0.43
1:C:164:SER:OG	1:C:165:TRP:N	2.51	0.43
1:D:116:ASP:OD1	1:D:116:ASP:N	2.41	0.43
1:D:638:SER:OG	1:D:639:GLN:N	2.51	0.43
1:E:91:LEU:HD13	1:E:94:ILE:HD13	2.00	0.43
1:B:260:ASP:HB2	1:B:262:GLU:HG3	2.01	0.43
1:D:660:ARG:HE	1:D:731:LEU:HD23	1.84	0.43
1:F:692:LEU:O	1:F:696:ASN:ND2	2.52	0.43
1:E:531:ASN:HA	1:E:534:ARG:HE	1.83	0.43
1:F:361:CYS:HA	1:F:362:PRO:HD3	1.94	0.43
1:A:326:LEU:HA	1:A:329:LEU:HD12	2.00	0.42
1:A:668:PRO:HA	1:A:671:TRP:HB2	2.00	0.42
1:B:279:ALA:O	1:B:283:THR:OG1	2.34	0.42
1:E:123:PHE:HZ	1:E:294:PHE:HE1	1.66	0.42
1:B:142:ASP:O	1:B:151:ARG:NH2	2.49	0.42
1:D:373:PHE:HE2	1:D:377:ARG:HH21	1.67	0.42
1:D:420:GLN:HE21	1:D:472:TRP:H	1.66	0.42
1:A:509:ASP:OD1	1:A:509:ASP:N	2.53	0.42
1:B:130:ILE:HD13	1:B:130:ILE:HA	1.91	0.42
1:C:247:THR:HG22	1:C:248:THR:H	1.84	0.42
1:D:638:SER:OG	1:D:640:GLU:OE1	2.36	0.42
1:F:266:LYS:HB2	1:F:266:LYS:HE3	1.78	0.42
1:F:512:LEU:HB3	1:F:516:LYS:HE2	2.01	0.42
1:A:367:ASP:OD1	1:A:367:ASP:N	2.52	0.42
1:B:712:LEU:HA	1:B:715:GLN:HB2	2.00	0.42
1:E:464:VAL:HA	1:E:467:ALA:HB3	2.01	0.42
1:E:711:ASP:OD1	1:E:711:ASP:N	2.51	0.42
1:A:190:GLU:HG2	1:A:193:TRP:CD2	2.55	0.42
1:A:664:THR:HA	1:B:664:THR:HG23	2.02	0.42
1:B:254:GLU:HA	1:B:283:THR:HG21	2.01	0.42
1:C:699:SER:OG	1:C:700:THR:N	2.52	0.42

Continued on next page...

For Manuscript Review

Continued from previous page...

Atom-1	Atom-2	Interatomic distance (Å)	Clash overlap (Å)
1:D:605:GLU:HA	1:D:625:TRP:HE1	1.84	0.42
1:B:684:CYS:O	1:B:688:SER:OG	2.27	0.42
1:C:447:SER:OG	1:C:449:ARG:NH1	2.53	0.42
1:E:591:PHE:HB3	1:E:596:GLY:HA2	2.02	0.42
1:F:88:ARG:HG2	1:F:92:ARG:NE	2.34	0.42
1:D:345:TYR:HA	1:D:348:ARG:HB2	2.01	0.42
1:D:533:LEU:HD12	1:D:533:LEU:HA	1.85	0.42
1:D:653:ALA:HA	1:D:656:ILE:HD12	2.01	0.42
1:E:598:LYS:HB3	1:E:598:LYS:HE3	1.79	0.42
1:A:323:VAL:O	1:A:327:THR:OG1	2.32	0.42
1:A:523:GLN:HB3	1:A:527:ARG:HH12	1.85	0.42
1:B:114:ARG:HB2	1:B:118:GLY:HA3	2.01	0.42
1:B:284:ALA:O	1:B:288:THR:OG1	2.34	0.42
1:C:700:THR:HA	1:C:703:VAL:HG12	2.01	0.42
1:E:530:LEU:HD23	1:E:530:LEU:HA	1.90	0.42
1:D:220:GLU:HB3	1:D:223:LEU:HB3	2.02	0.42
1:F:524:LYS:HD3	1:F:524:LYS:HA	1.94	0.42
1:B:730:ARG:HD2	1:B:733:ARG:HH12	1.84	0.42
1:F:485:LEU:HD22	1:F:516:LYS:HG3	2.02	0.42
1:A:533:LEU:HD12	1:A:533:LEU:HA	1.90	0.41
1:B:151:ARG:O	1:B:155:THR:N	2.52	0.41
1:B:458:LYS:HD3	1:B:487:GLN:HA	2.02	0.41
1:B:676:LEU:HD23	1:B:676:LEU:HA	1.91	0.41
1:C:571:ALA:HA	1:C:574:ARG:HB3	2.02	0.41
1:D:759:ASP:OD2	1:D:764:GLN:NE2	2.53	0.41
1:B:230:ARG:HE	1:B:234:LEU:HD22	1.85	0.41
1:C:450:MET:HG3	1:C:461:PRO:HB3	2.02	0.41
1:D:296:TYR:HD2	1:D:297:LEU:HG	1.85	0.41
1:B:639:GLN:NE2	1:B:640:GLU:OE2	2.53	0.41
1:C:400:LEU:HD23	1:C:403:GLU:HB3	2.03	0.41
1:F:353:ASP:OD1	1:F:354:GLY:N	2.54	0.41
1:F:364:LYS:HB2	1:F:364:LYS:HE2	1.88	0.41
1:C:300:ILE:HG21	1:C:308:VAL:HG23	2.02	0.41
1:D:471:PRO:HD2	1:D:474:ALA:HB3	2.01	0.41
1:F:444:ARG:HE	1:F:449:ARG:HD2	1.85	0.41
1:F:707:ASP:HA	1:F:710:VAL:HG12	2.02	0.41
1:C:199:GLU:HB2	1:C:233:LYS:HD2	2.03	0.41
1:F:503:MET:HB3	1:F:505:LEU:HD12	2.03	0.41
1:E:573:GLU:HG3	1:E:657:PHE:HB3	2.03	0.41
1:F:506:PHE:HA	1:F:511:TYR:HE2	1.85	0.41
1:B:730:ARG:HD2	1:B:730:ARG:HA	1.83	0.41

Continued on next page...

For Manuscript Review

Continued from previous page...

Atom-1	Atom-2	Interatomic distance (Å)	Clash overlap (Å)
1:C:136:PRO:O	1:C:174:ARG:NH1	2.49	0.41
1:C:163:LYS:HE3	1:C:169:THR:HG23	2.03	0.41
1:D:377:ARG:HA	1:D:381:TYR:H	1.86	0.41
1:D:539:ARG:O	1:D:541:ASN:ND2	2.54	0.41
1:E:350:TRP:HZ2	1:E:387:VAL:HG11	1.86	0.41
1:E:644:GLY:HA2	1:E:684:CYS:HB3	2.03	0.41
1:E:663:LEU:HD23	1:E:663:LEU:HA	1.88	0.41
1:C:184:TRP:O	1:C:188:HIS:NE2	2.54	0.41
1:C:224:LYS:HD2	1:C:224:LYS:HA	1.94	0.41
1:C:667:ARG:NH1	1:D:655:GLU:OE2	2.54	0.41
1:E:94:ILE:HD12	1:E:94:ILE:HA	1.98	0.41
1:F:656:ILE:HD12	1:F:656:ILE:HA	1.89	0.41
1:A:292:LYS:HE2	1:A:292:LYS:HB2	1.79	0.40
1:B:181:GLU:O	1:B:184:TRP:NE1	2.54	0.40
1:B:709:GLN:NE2	1:B:709:GLN:O	2.54	0.40
1:B:720:ARG:HA	1:B:723:GLN:HG2	2.03	0.40
1:C:229:GLU:HG3	1:C:232:ARG:HG3	2.03	0.40
1:D:345:TYR:HA	1:D:348:ARG:HD3	2.03	0.40
1:D:721:VAL:HG23	1:D:733:ARG:HB3	2.03	0.40
1:F:731:LEU:HD23	1:F:731:LEU:HA	1.96	0.40
1:A:415:THR:OG1	1:A:437:CYS:SG	2.73	0.40
1:A:519:PHE:HA	1:A:522:PHE:HB3	2.04	0.40
1:D:268:ARG:HD2	1:D:272:GLY:HA2	2.02	0.40
1:E:224:LYS:HD3	1:E:224:LYS:HA	1.79	0.40
1:F:587:VAL:HG23	1:F:604:LEU:HD13	2.03	0.40
1:B:111:LEU:HA	1:B:165:TRP:HH2	1.87	0.40
1:B:232:ARG:HH22	1:B:233:LYS:HD2	1.86	0.40
1:A:560:LEU:HD11	1:A:746:VAL:HG11	2.03	0.40
1:C:102:SER:O	1:C:106:THR:OG1	2.38	0.40
1:C:114:ARG:HB3	1:C:117:GLY:H	1.87	0.40
1:C:270:LEU:HD12	1:C:360:ASN:HA	2.03	0.40
1:C:571:ALA:HB2	1:C:574:ARG:HH21	1.87	0.40
1:E:181:GLU:O	1:E:184:TRP:NE1	2.55	0.40
1:E:629:LEU:HD23	1:E:629:LEU:HA	1.82	0.40
1:F:718:THR:HA	1:F:721:VAL:HG12	2.03	0.40
1:C:180:GLN:HE22	1:C:217:PRO:HD3	1.86	0.40
1:C:296:TYR:OH	1:C:312:TYR:O	2.36	0.40
1:D:259:LEU:HD12	1:D:261:TRP:NE1	2.36	0.40
1:D:312:TYR:HA	1:D:313:PRO:HA	1.82	0.40

There are no symmetry-related clashes.

For Manuscript Review

5.3 Torsion angles (i)

5.3.1 Protein backbone (i)

In the following table, the Percentiles column shows the percent Ramachandran outliers of the chain as a percentile score with respect to all PDB entries followed by that with respect to all EM entries.

The Analysed column shows the number of residues for which the backbone conformation was analysed, and the total number of residues.

Mol	Chain	Analysed	Favoured	Allowed	Outliers	Percentiles	
1	A	686/775 (88%)	638 (93%)	48 (7%)	0	100	100
1	B	686/775 (88%)	645 (94%)	41 (6%)	0	100	100
1	C	686/775 (88%)	646 (94%)	40 (6%)	0	100	100
1	D	686/775 (88%)	627 (91%)	59 (9%)	0	100	100
1	E	686/775 (88%)	654 (95%)	32 (5%)	0	100	100
1	F	678/775 (88%)	637 (94%)	41 (6%)	0	100	100
All	All	4108/4650 (88%)	3847 (94%)	261 (6%)	0	100	100

There are no Ramachandran outliers to report.

5.3.2 Protein sidechains (i)

In the following table, the Percentiles column shows the percent sidechain outliers of the chain as a percentile score with respect to all PDB entries followed by that with respect to all EM entries.

The Analysed column shows the number of residues for which the sidechain conformation was analysed, and the total number of residues.

Mol	Chain	Analysed	Rotameric	Outliers	Percentiles	
1	A	597/672 (89%)	592 (99%)	5 (1%)	81	89
1	B	597/672 (89%)	593 (99%)	4 (1%)	84	91
1	C	597/672 (89%)	594 (100%)	3 (0%)	88	94
1	D	597/672 (89%)	595 (100%)	2 (0%)	92	96
1	E	597/672 (89%)	592 (99%)	5 (1%)	81	89
1	F	593/672 (88%)	589 (99%)	4 (1%)	84	91
All	All	3578/4032 (89%)	3555 (99%)	23 (1%)	86	93

For Manuscript Review

All (23) residues with a non-rotameric sidechain are listed below:

Mol	Chain	Res	Type
1	A	200	ILE
1	A	593	ARG
1	A	669	ASP
1	A	670	LEU
1	A	687	LEU
1	B	174	ARG
1	B	432	ARG
1	B	645	ASP
1	B	690	ARG
1	C	156	LEU
1	C	360	ASN
1	C	450	MET
1	D	522	PHE
1	D	592	ARG
1	E	303	LYS
1	E	358	THR
1	E	432	ARG
1	E	642	ILE
1	E	690	ARG
1	F	92	ARG
1	F	116	ASP
1	F	527	ARG
1	F	614	ASP

Sometimes sidechains can be flipped to improve hydrogen bonding and reduce clashes. All (37) such sidechains are listed below:

Mol	Chain	Res	Type
1	A	401	HIS
1	A	521	ASN
1	A	590	HIS
1	A	639	GLN
1	A	666	GLN
1	A	694	GLN
1	B	286	GLN
1	B	414	ASN
1	B	442	GLN
1	B	694	GLN
1	C	168	HIS
1	C	180	GLN
1	C	243	HIS
1	C	275	HIS

Continued on next page...

For Manuscript Review

Continued from previous page...

Mol	Chain	Res	Type
1	C	414	ASN
1	C	448	ASN
1	C	521	ASN
1	D	134	GLN
1	D	420	GLN
1	D	487	GLN
1	D	541	ASN
1	D	661	HIS
1	D	715	GLN
1	E	182	ASN
1	E	251	HIS
1	E	357	HIS
1	E	564	ASN
1	E	666	GLN
1	E	715	GLN
1	F	180	GLN
1	F	360	ASN
1	F	405	ASN
1	F	414	ASN
1	F	442	GLN
1	F	675	GLN
1	F	694	GLN
1	F	696	ASN

5.3.3 RNA (i)

There are no RNA molecules in this entry.

5.4 Non-standard residues in protein, DNA, RNA chains (i)

There are no non-standard protein/DNA/RNA residues in this entry.

5.5 Carbohydrates (i)

There are no monosaccharides in this entry.

5.6 Ligand geometry (i)

There are no ligands in this entry.

For Manuscript Review

5.7 Other polymers ⓘ

There are no such residues in this entry.

5.8 Polymer linkage issues ⓘ

There are no chain breaks in this entry.

For Manuscript Review

6 Map visualisation ⓘ

This section contains visualisations of the EMDB entry EMD-35206. These allow visual inspection of the internal detail of the map and identification of artifacts.

No raw map or half-maps were deposited for this entry and therefore no images, graphs, etc. pertaining to the raw map can be shown.

6.1 Orthogonal projections ⓘ

6.1.1 Primary map

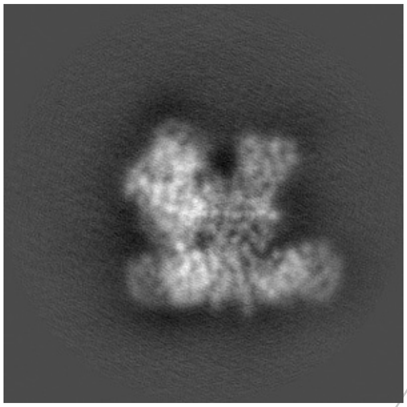
X

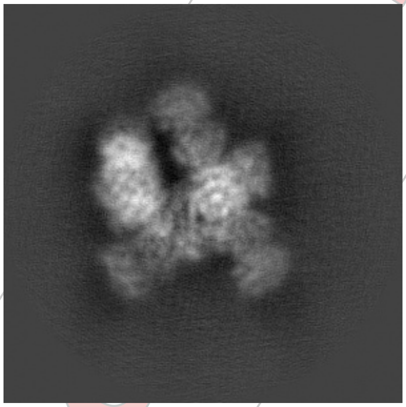
Y

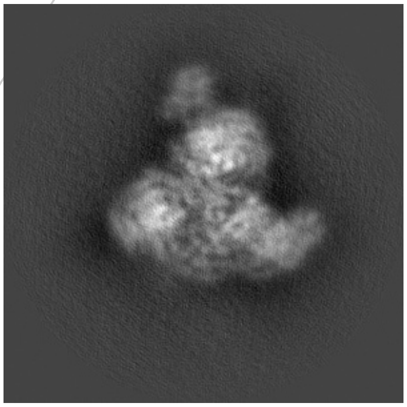
Z

The images above show the map projected in three orthogonal directions.

6.2 Central slices ⓘ

6.2.1 Primary map

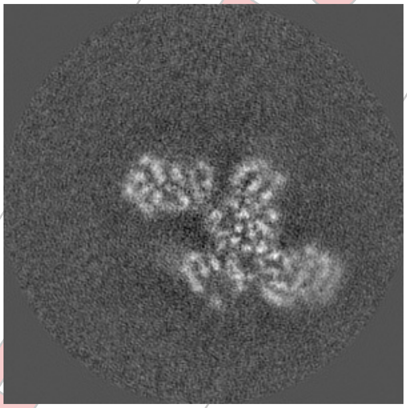
X Index: 140

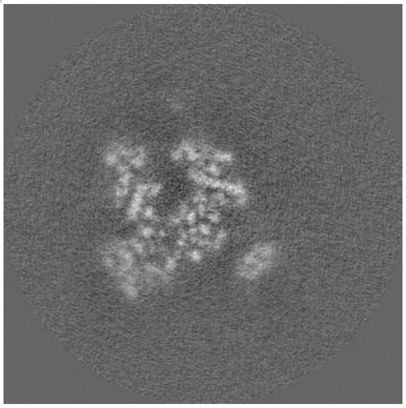
Y Index: 140

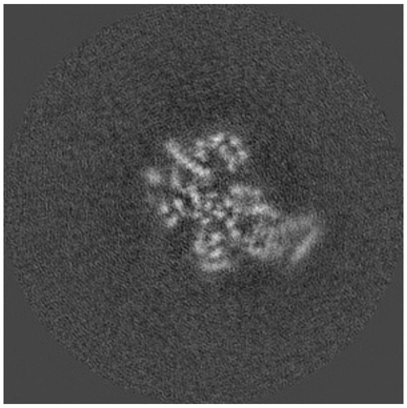
Z Index: 140

For Manuscript Review

The images above show central slices of the map in three orthogonal directions.

6.3 Largest variance slices (i)

6.3.1 Primary map

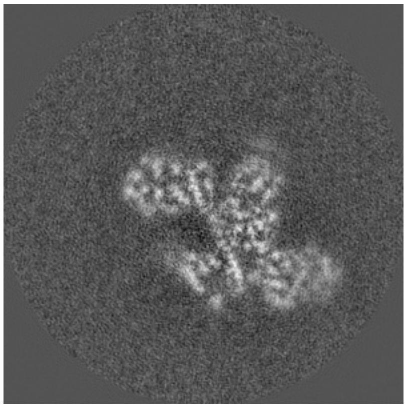

X Index: 142

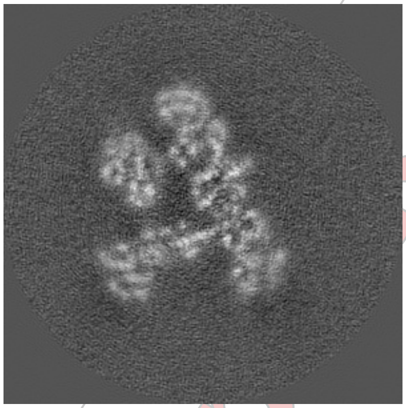

Y Index: 127

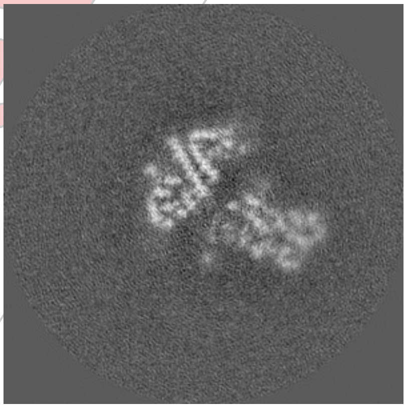

Z Index: 132

The images above show the largest variance slices of the map in three orthogonal directions.

6.4 Orthogonal surface views (i)

6.4.1 Primary map

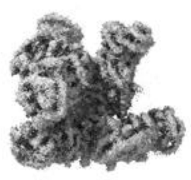

X

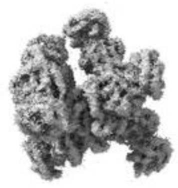

Y

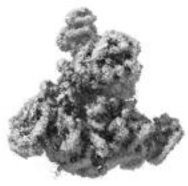

Z

The images above show the 3D surface view of the map at the recommended contour level 0.0176. These images, in conjunction with the slice images, may facilitate assessment of whether an appropriate contour level has been provided.

For Manuscript Review

6.5 Mask visualisation (i)

This section was not generated. No masks/segmentation were deposited.

For Manuscript Review

7 Map analysis (i)

This section contains the results of statistical analysis of the map.

7.1 Map-value distribution (i)

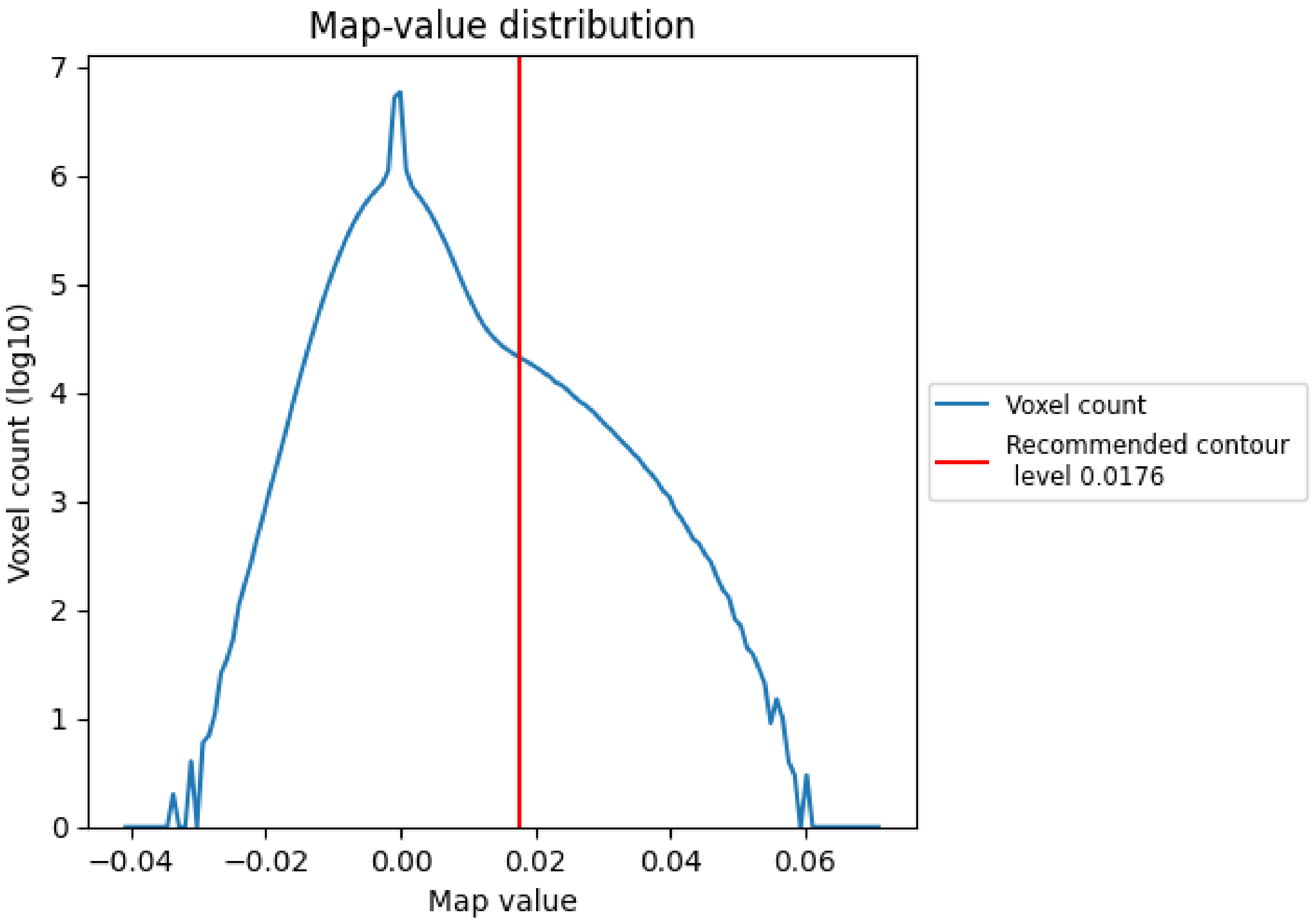

The map-value distribution is plotted in 128 intervals along the x-axis. The y-axis is logarithmic. A spike in this graph at zero usually indicates that the volume has been masked.

For Manuscript Review

7.2 Volume estimate (i)

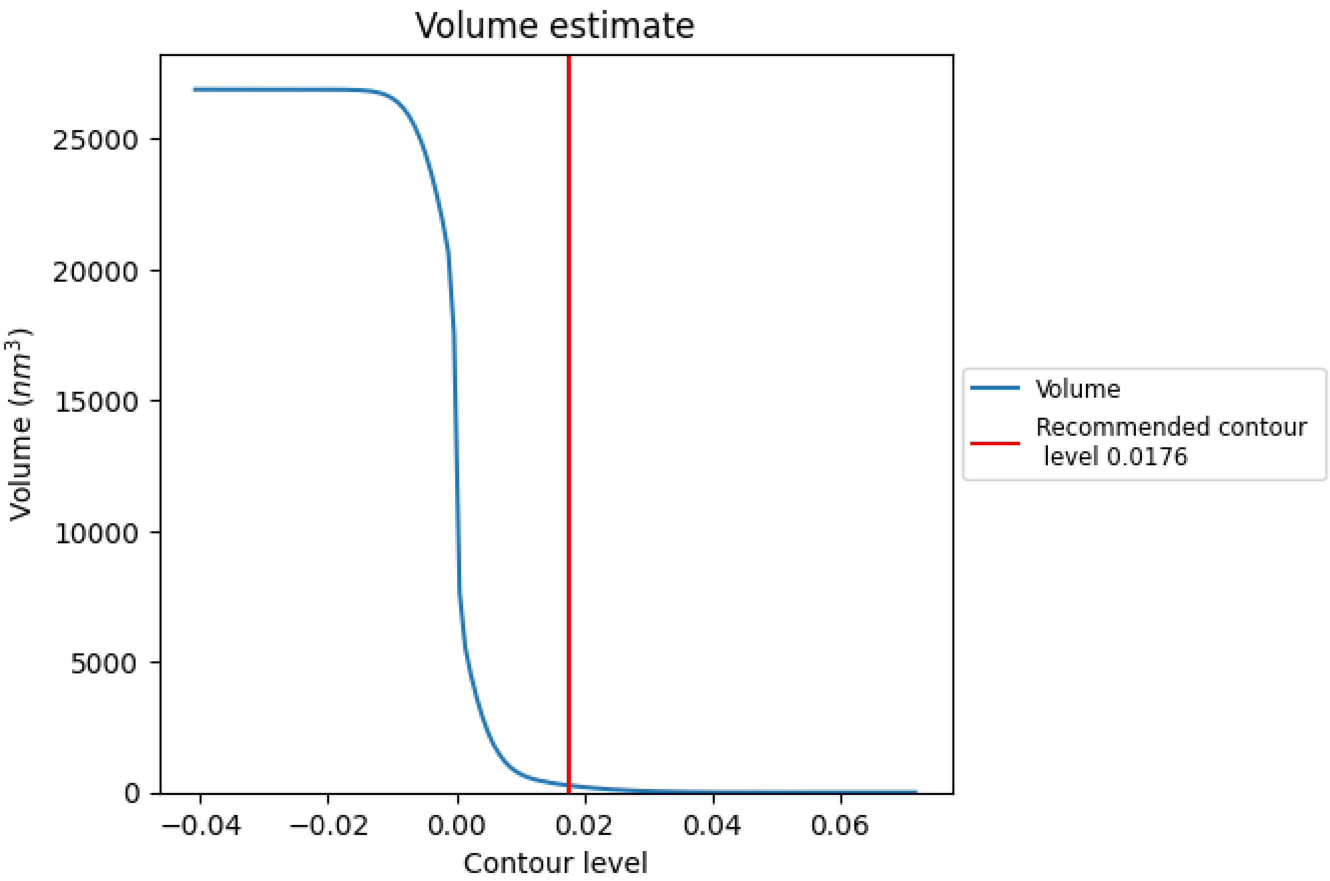

The volume at the recommended contour level is 268 nm^3; this corresponds to an approximate mass of 242 kDa.

The volume estimate graph shows how the enclosed volume varies with the contour level. The recommended contour level is shown as a vertical line and the intersection between the line and the curve gives the volume of the enclosed surface at the given level.

For Manuscript Review

7.3 Rotationally averaged power spectrum (i)

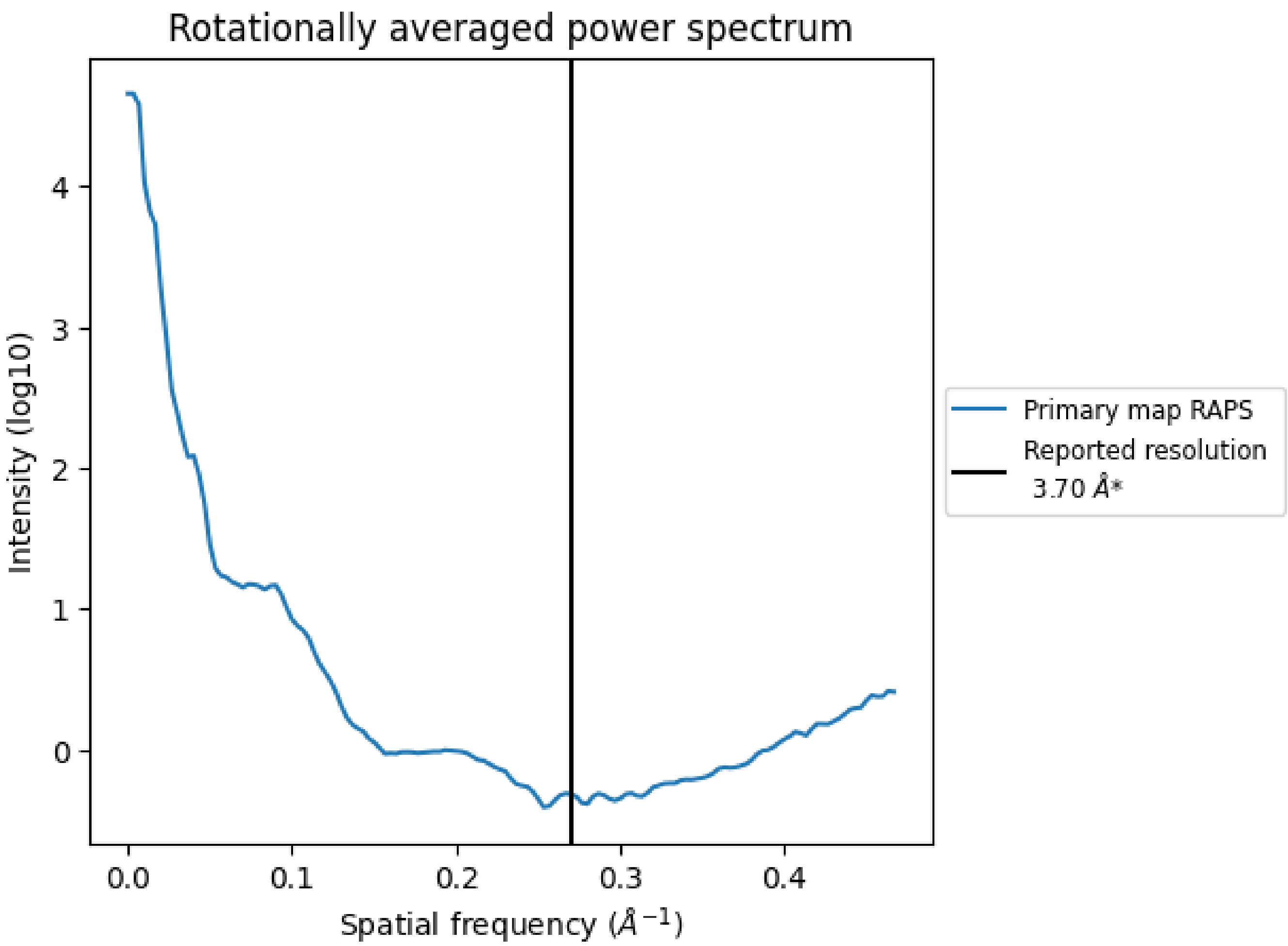

*Reported resolution corresponds to spatial frequency of 0.270 Å^{-1}

For Manuscript Review

8 Fourier-Shell correlation (i)

This section was not generated. No FSC curve or half-maps provided.

For Manuscript Review

9 Map-model fit ⓘ

This section contains information regarding the fit between EMDB map EMD-35206 and PDB model 8I6T. Per-residue inclusion information can be found in section 3 on page 5.

9.1 Map-model overlay ⓘ

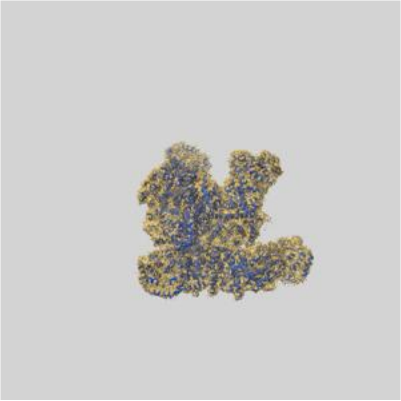

X

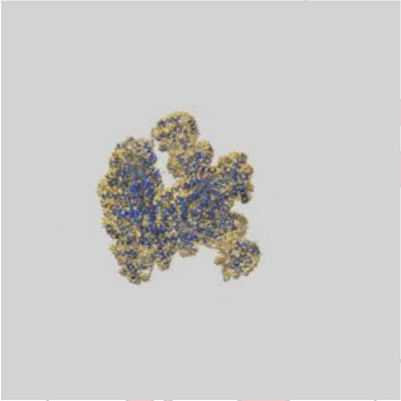

Y

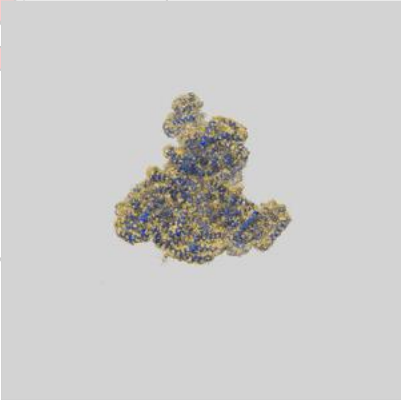

Z

The images above show the 3D surface view of the map at the recommended contour level 0.0176 at 50% transparency in yellow overlaid with a ribbon representation of the model coloured in blue. These images allow for the visual assessment of the quality of fit between the atomic model and the map.

For Manuscript Review

9.2 Q-score mapped to coordinate model (i)

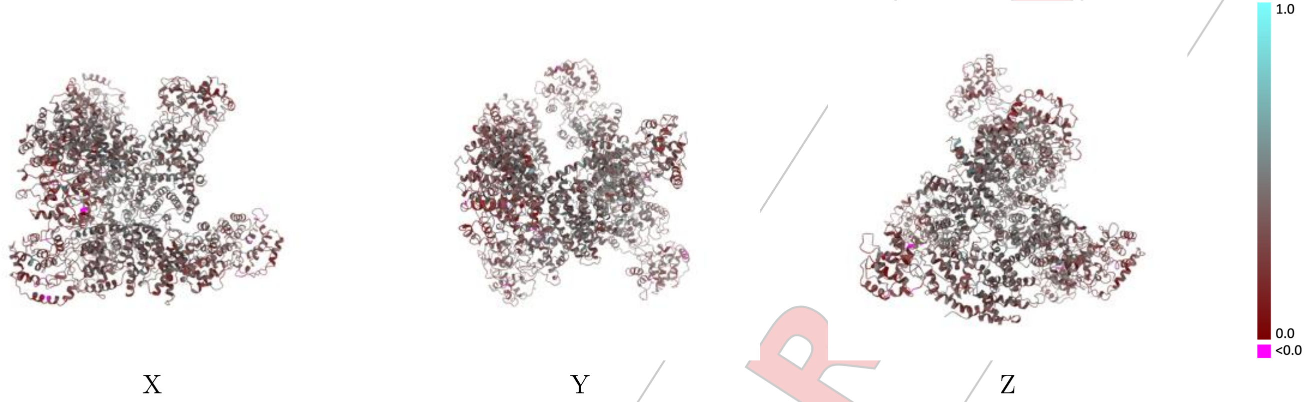

The images above show the model with each residue coloured according its Q-score. This shows their resolvability in the map with higher Q-score values reflecting better resolvability. Please note: Q-score is calculating the resolvability of atoms, and thus high values are only expected at resolutions at which atoms can be resolved. Low Q-score values may therefore be expected for many entries.

9.3 Atom inclusion mapped to coordinate model (i)

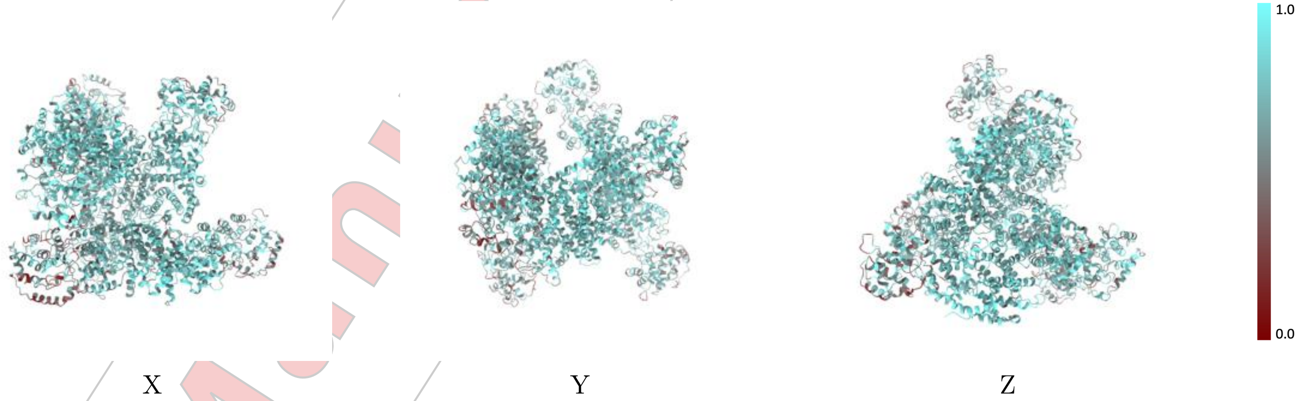

The images above show the model with each residue coloured according to its atom inclusion. This shows to what extent they are inside the map at the recommended contour level (0.0176).

For Manuscript Review

9.4 Atom inclusion (i)

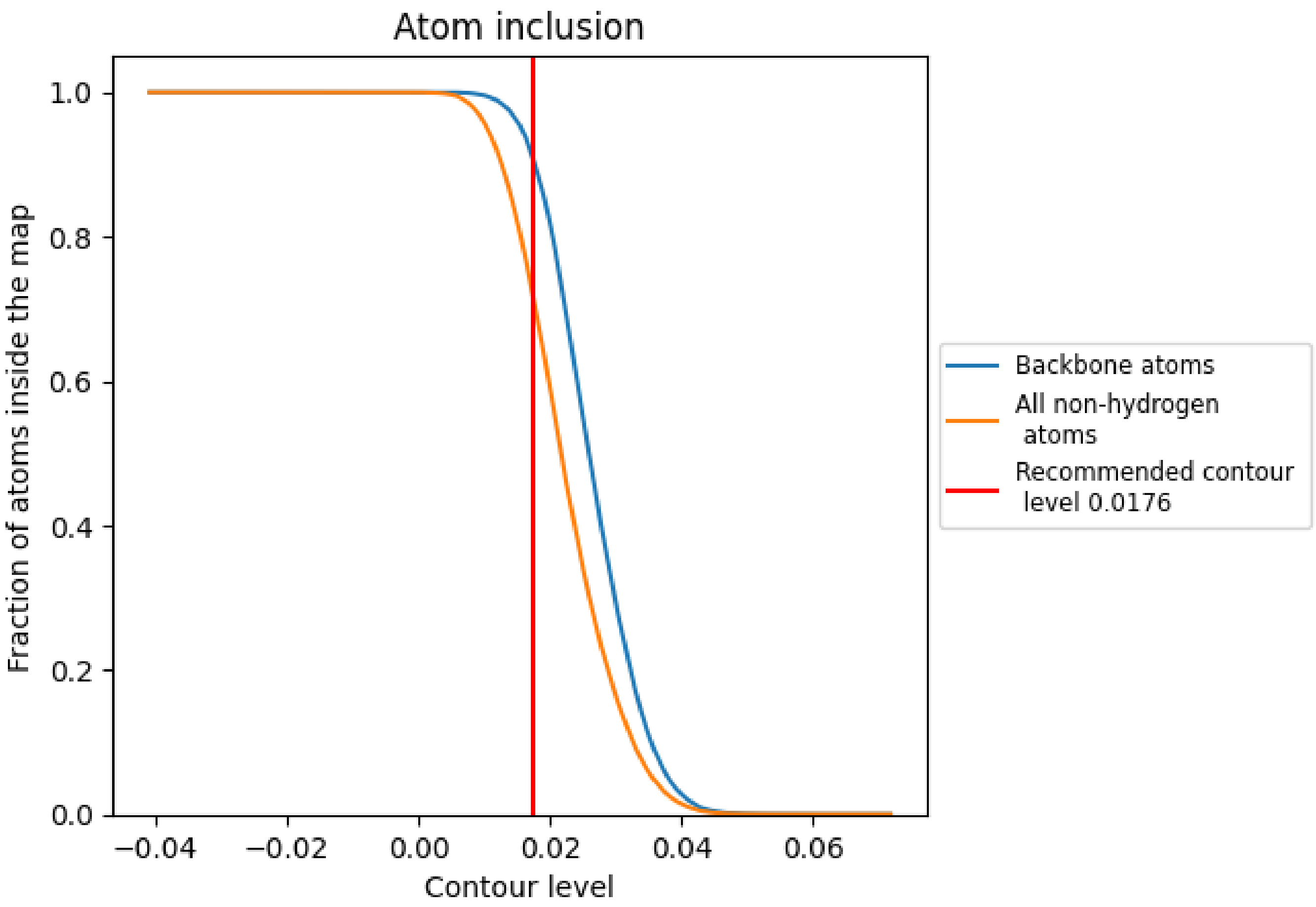

At the recommended contour level, 90% of all backbone atoms, 71% of all non-hydrogen atoms, are inside the map.

For Manuscript Review

9.5 Map-model fit summary (i)

The table lists the average atom inclusion at the recommended contour level (0.0176) and Q-score for the entire model and for each chain.

Chain	Atom inclusion	Q-score
All	0.7109	0.3600
A	0.7161	0.3490
B	0.7477	0.3590
C	0.6927	0.3810
D	0.7624	0.3860
E	0.6607	0.3510
F	0.6856	0.3310

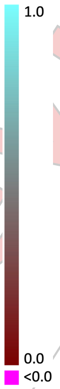

For Manuscript Review
